# Amine‐Catalyzed Copper‐Mediated C−H Sulfonylation of Benzaldehydes via a Transient Imine Directing Group**

**DOI:** 10.1002/anie.202202933

**Published:** 2022-05-05

**Authors:** Joe I. Higham, James A. Bull

**Affiliations:** ^1^ Department of Chemistry Imperial College London Molecular Sciences Research Hub, White City Campus Wood Lane London W12 0BZ UK

**Keywords:** C−H Functionalization, Copper, Directing Groups, Organocatalysis, Sulfones

## Abstract

Transient directing groups (TDGs) can provide a powerful means for C−H functionalization without requiring additional steps for directing group introduction and removal. We report the first use of a TDG in combination with copper to effect C−H functionalization. The regioselective copper mediated β−C(sp^2^)−H sulfonylation of aldehydes with sulfinate salts is accomplished using catalytic β‐alanine to form a transient imine. A broad range of sulfonylated benzaldehydes are prepared using copper fluoride as both copper source and oxidant, involving a [5,6] cupracyclic intermediate. γ‐(*peri*)‐Sulfonylation of napthyl and phenanthrenyl carboxaldehydes is achieved through [6,6] cupracyclic intermediates. Further derivatisation of the aldehyde products is demonstrated. Kinetic experiments and Hammett analysis suggest the turnover limiting step to be a concerted asynchronous C−H cleavage via a dearomative Wheland‐type transition state.

## Graphical abstract

Transient C−H sulfonylation of benzaldehydes is achieved using copper and catalytic β‐alanine to form a directing group in situ. Aldehydes and sulfinate salts are coupled in a single step in high yields. Kinetic and mechanistic investigations are presented, with insights to the C−H activation step.

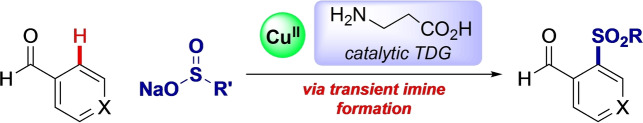

## Introduction

**Figure 1:**
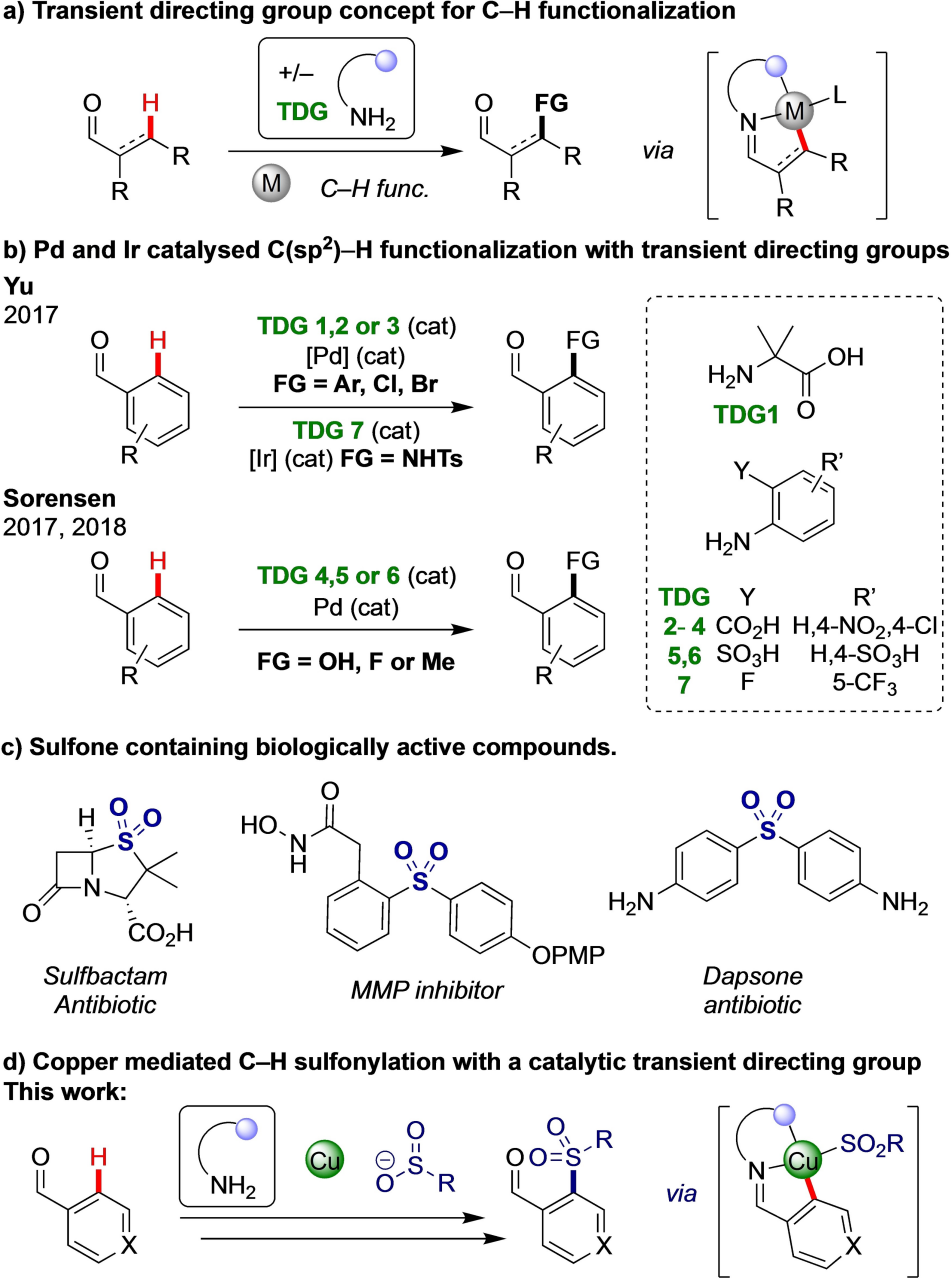
a) Transient C−H functionalisation concept. b) Leading examples of C(sp^2^)−H functionalization with transient directing groups using precious metals. c) Biologically important sulfones. d) Copper mediated sulfonylation of aldehydes controlled by a transient directing group catalytic in amine.

C−H Functionalization offers the potential for streamlined and improved processes in the synthesis of valuable organic compounds.^[1]^ Directing groups have provided an effective means to control site‐selectivity between numerous similar C−H bonds in C−H functionalization, by correctly locating a transition metal and promoting proximity driven C−H activation.^[2]^ However, the requirement for amide‐bound directing groups continues to present an intrinsic efficiency limitation, due to the additional discrete steps required to install and remove these groups. Recently transient directing groups (TDGs) have been developed, notably for arylation processes, whereby the directing group is formed and removed within the C−H functionalisation reaction by taking advantage of common valuable functional groups such as aldehydes (Figure 1a).^[3]^ Transient directing groups formed as imines have been used in particular to selectively functionalize aldehyde or amine components, though in a limited range of transformations to date. A transient directing group approach was first described by Jun for the alkylation of aldehydic C−H bonds.^[4]^ In 2016, Yu reignited interest in this field by demonstrating powerful benzylic and sp^3^ C−H functionalization of aldehydes and ketones respectively using co‐catalytic palladium acetate and glycine to form a transient coordinating imine.^[5]^ In 2017, Yu reported the palladium catalyzed transient *ortho*‐C−H functionalization of benzaldehydes, achieving arylation, bromination and chlorination with imines derived from anilines (Figure 1b).^[6]^ Contemporaneously, Sorensen developed C(sp^2^)−H hydroxylation, fluorination and methylation processes.^[7]^ Noticeably, there are no examples of C−S bond formation using transient C−H functionalization, which may reflect sulfur functionalities being poisons for precious metal catalysts,^[8]^ and which is especially problematic in transient C−H activation systems where the concentration of the active directing group is low. Furthermore, the majority of transient directing groups use precious metal catalysis, mainly palladium, but also rhodium, ruthenium and iridium.[^3^, ^9^, ^10^]

The move towards more sustainable, earth abundant metals presents a notable challenge in C−H functionalization, due in part to the poor mechanistic understanding of base metal mediated processes making rational reaction optimization particularly challenging.[^11^, ^12^] To date there are only two reports of 3d metals being used for C−H functionalisation in combination with TDGs, both in cobalt catalysed amidation.^[13]^ Specifically, there are no examples of copper mediated C−H functionalization with TDGs to date.

Sulfones feature heavily in active pharmaceutical ingredients and biologically active compounds (Figure 1c),[^14^, ^15^] and recent years has seen extensive investigation into their synthesis. Copper and palladium catalysed cross‐coupling methods have allowed the introduction of the sulfone motif without relying on preinstalled sulfur moieties.^[16]^ However, these methods rely on the manipulation of a pre‐installed functionality, often in the form of an aryl iodide. Strategies that can directly replace C−H bonds with sulfonyl functionality are highly sought after as they allow the generation of sulfones in a step efficient manner and can allow late‐stage diversification.[^17^, ^18^] In 2015, Tan,^[17a]^ Shi,^[17b]^ and Manolikakes^[17c]^ demonstrated copper mediated oxidative *ortho*‐sulfonylation of benzoic acid derived compounds using amide bound directing groups.

Here we report the first application of a transient directing group in copper enabled C−H functionalization, which is catalytic in amine. This also represents the first C−S bond forming transient C−H functionalization process, enabling the efficient construction of sulfones (Figure 1d). The aldehyde can be directly used for further derivatisation. Kinetics and deuteration studies have provided insight into the reaction mechanism of this C−H functionalization process.

## Results and Discussion

**Scheme 1:**
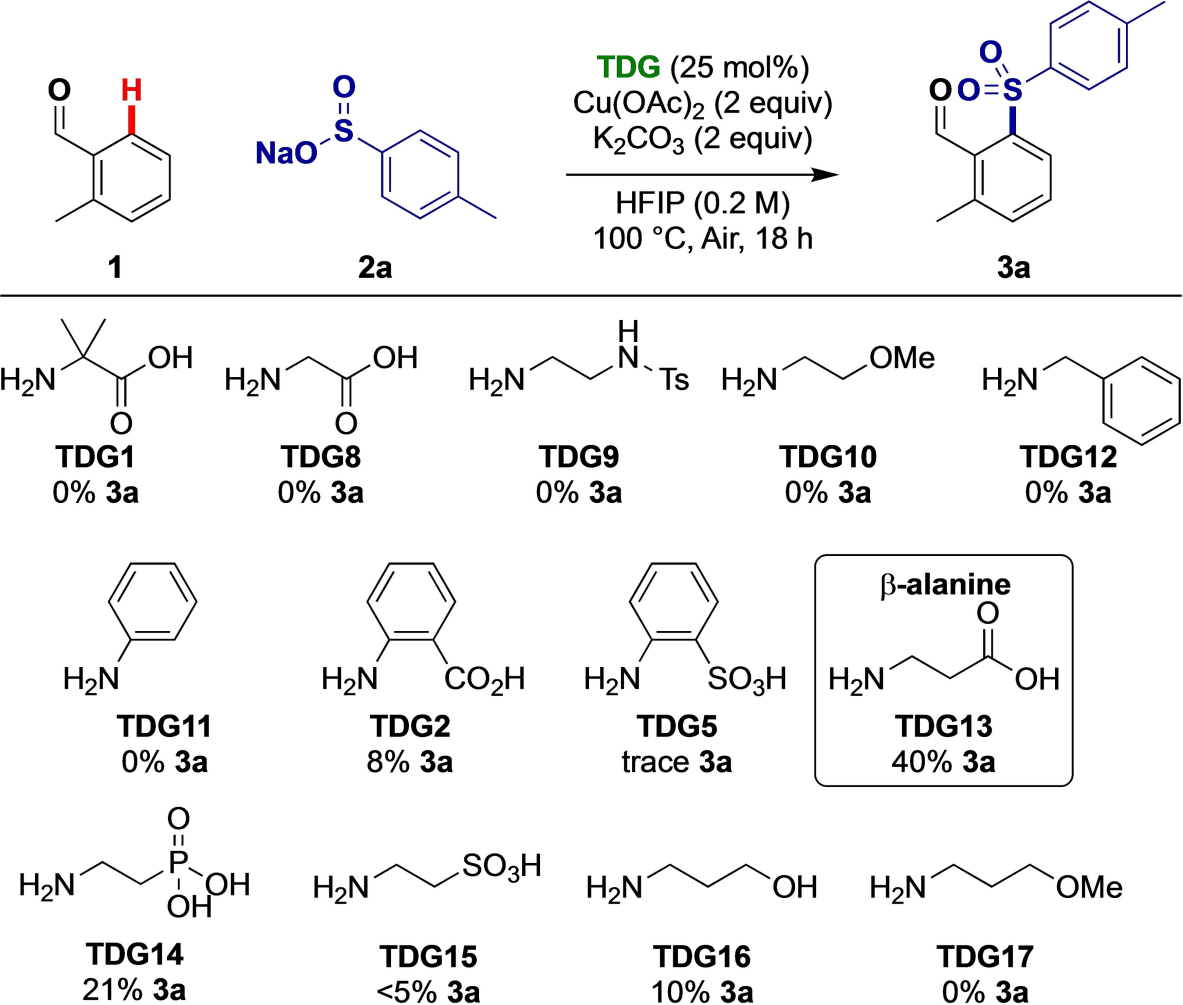
Assessing transient directing groups in copper mediated C(sp^2^)−H sulfonylation. Yield determined by ^1^H‐NMR using 1,3,5‐trimethoxybenzene as an internal standard.

Initially, we aimed to establish whether copper mediated C−H functionalization reactions were feasible with a catalytic TDG. Common bidentate groups that function as transient directing groups also chelate copper, and indeed find application as ligands in copper mediated reactions. Our studies focussed on the C−H sulfonylation of benzaldehyde and *o*‐tolualdehyde by assessing the potential of different classes of amines as TDGs. Copper (II) acetate was selected for early studies to effect both a putative concerted metalation deprotonation (CMD) process and subsequent functionalization steps, as well as to provide an inexpensive and non‐toxic stoichiometric oxidant. Potassium carbonate base and HFIP,^[19]^ used previously in Pd‐mediated transient processes, were employed to screen for successful *ortho*‐sulfonylation, using 25 mol % of the transient directing groups (Scheme 1).

Bidentate transient directing groups **TDG1**, and **TDG8**–**TDG10** which had previously reported in C(sp^2^)−H functionalization with Pd were ineffective, as were monodentate aniline and benzylamine. Pleasingly, reactivity was observed when using anthranilic acid (**TDG2**), forming a presumed [5,6] cupracyclic intermediate, but the related orthanilic acid was ineffective. β‐Alanine (**TDG13**) was more effective, giving 40 % yield of **3** **a**. Changing the acidic secondary binding site (P(O)(OH)_2_, SO_3_H, OH or OMe; **TDG14**–**TDG17**) gave lower yields, hence **TDG13** was selected for further study.

We next investigated reaction variables including copper sources, solvents, bases, and other additives.^[20]^ CuF_2_ was the most effective copper source, and the yield could be further improved with carboxylate additives. Using 0.5 equiv of Cu(OAc)_2_ along with 1.5 equiv CuF_2_ was most effective. These copper sources both provide the necessary stoichiometric oxidant and effect the C−H functionalization. Further investigation confirmed K_2_CO_3_ and HFIP as the most effective base and solvent respectively. We observed that 25 mol % of the TDG was optimal, and variation in the loadings of β‐alanine (5–100 mol %) resulted in lower yields of the sulfonyl aldehyde. A subsequent design of experiment (DoE) optimization focused on CuF_2_ equivalents, aldehyde equivalents and reaction concentration identified a key positive interaction between increasing equivalents of aldehyde and CuF_2_ (see Supporting Information for further details).^[20a]^

Using a catalytic quantity of the TDG, the aldehyde in excess and 2 equiv. of readily available CuF_2_, aldehyde **3** **a** was isolated in 86 % yield (Table 1, entry 1). Importantly, no reaction was observed in the absence of the TDG (Entry 2). Reduced reactivity was observed when either of the copper salts were omitted (Entries 3 and 4), and when no copper salts were present there was no observable reaction (Entry 5). Using only Cu(OAc)_2_ (2.5 equiv) in place of CuF_2_ remained effective but with reduced yield (Entry 6). A low yield of **3** **a** was observed in the absence of K_2_CO_3_ (Entry 7). It was also possible to use the aldehyde as the limiting reagent to give 63 % yield of **3** **a** (Entry 8). In the absence of the sulfinate salt, we observed the coupling of HFIP to give HFIP ether **4**, whereby the solvent acts as a nucleophile in the reaction (Entry 9).^[21]^ We observed complete suppression of sulfone formation on addition of the radical trap TEMPO (Entry 10) indicating the potential for the involvement of radical species. The reaction was broadly tolerant to changes in concentration and increased temperature as assessed by the Glorius protocol, though sensitivity to increased water and oxygen was observed.[^20b^, ^22^]

**Table 1:** Control reactions describing deviation from the optimized conditions.

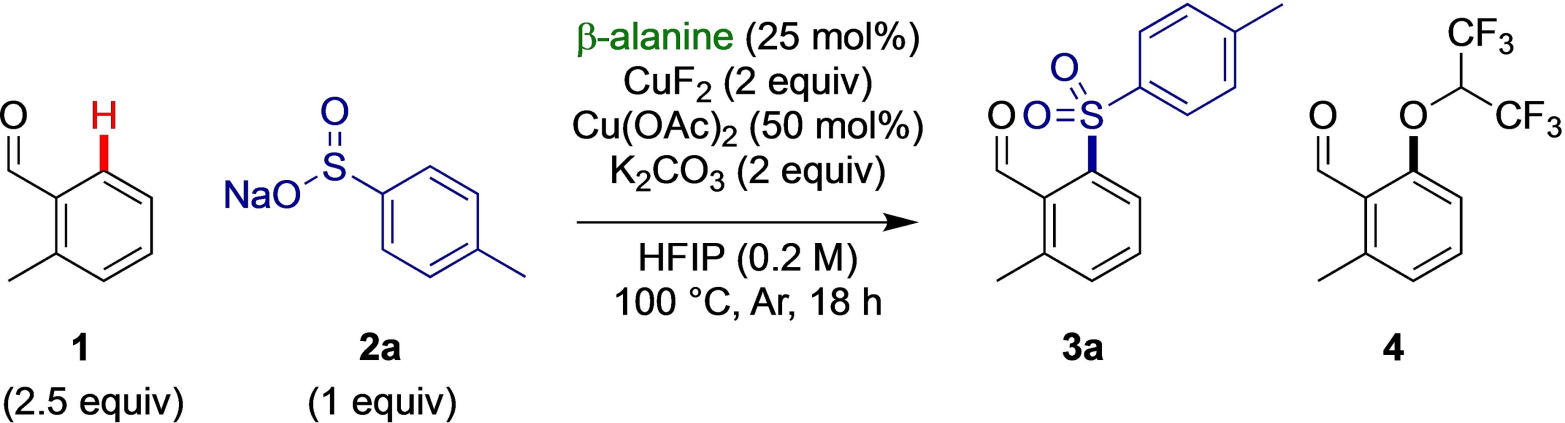				
		
Entry	Deviation from standard conditions	Yield [%]^[a]^		
		**3** **a**	**4**	Recovered **1**
1	None	93 (86)	0	153
2	No TDG	0	0	102
3	No CuF_2_	20	0	227
4	No Cu(OAc)_2_	82	0	165
5	No CuF_2_ or Cu(OAc)_2_	0	0	250
6	Cu(OAc)_2_ (2.5 equiv)	75	0	150
7	No K_2_CO_3_	18	0	202
8	Using aldehyde as the limiting reagent	63	0	30
9	No Sulfinate	0	33^[b]^ (19 %)^[c]^	61^[b]^
10	+TEMPO (1 equiv)	0	trace	178

Reactions performed using 0.2 mmol sulfinate salt. [a] Yield determined by ^1^H‐NMR using 1,3,5‐trimethoxybenzene as an internal standard. Isolated yield in parenthesis. Recovered **1** refers to recovered excess aldehyde from a maximum of 250 %. [b] Yield based on aldehyde as limiting reagent. [c] Yield on 0.5 mmol scale of aldehyde. Note that **4** is volatile.

**Scheme 2:**
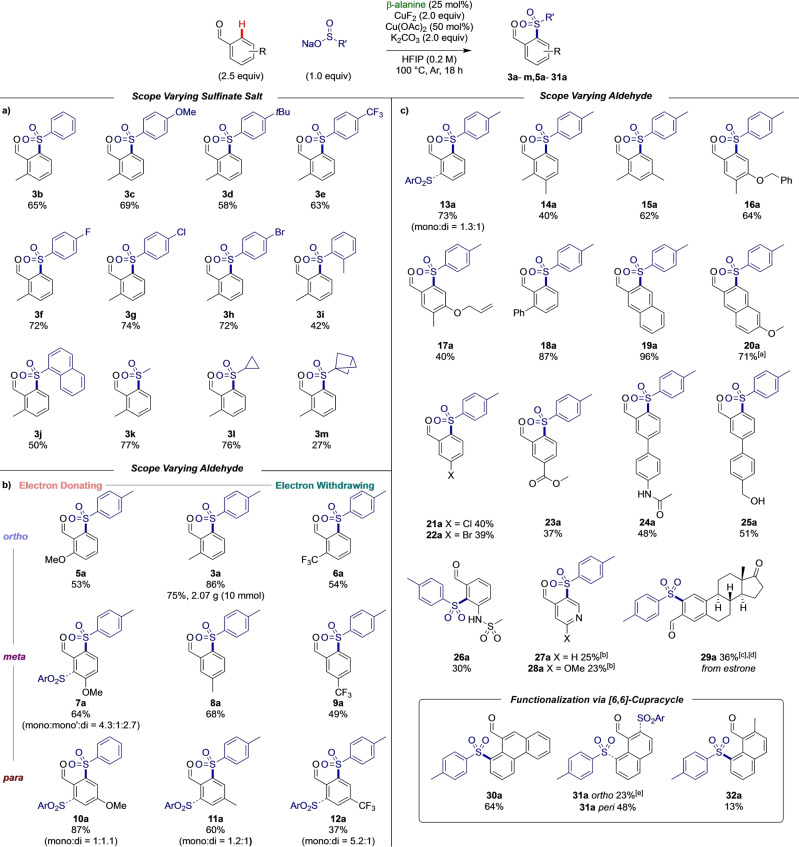
Reaction scope varying the sulfinate salt and aldehyde. Reactions performed on 0.2 mmol scale. Isolated yields reported. [a] 22 h reaction time. [b] NaHCO_3_ (sat. aq) workup was used. [c] 1 equiv aldehyde and 1.25 equiv sulfinate salt. [d] 91 % purity. [e] 87 % purity.

With these optimized conditions we investigated the substrate scope with changes in sulfinate salt and aldehyde. A wide range of electronically and sterically diverse sulfinate salts were effectively coupled to give sulfones **3** **a**–**3** **m** in good to excellent yields (Scheme 2a). Aryl sulfinate salts with electronically neutral (H) or electron donating (OMe, *t*Bu) substituents were well tolerated (**3** **b**–**3** **d**). Sulfinate salts bearing arenes with inductively electron withdrawing groups (*p*‐CF_3_, *p*‐halides) were particularly effective giving yields of 63–74 % without any unwanted cross coupling observed (**3** **e**–**3** **h**). More sterically encumbered *ortho*‐substituted sulfinates such as 1‐napthyl and *o*‐tolyl derivatives successfully gave sulfones **3** **i** and **3** **j**. Methyl and cyclopropyl sulfinate salts were both effective in this oxidative coupling reacting in 77 % and 76 % yield respectively (**3** **k** and **3** **l**). Additionally, bicyclo[1.1.1]pentane (BCP) sulfinate was used to introduce a BCP moiety which is increasingly of interest as a phenyl isostere. No obvious trend between the oxidation potential of the sulfinate salts and the yield of the coupling was observed,^[23]^ indicating direct oxidation of the salt to a sulfonyl radical may not be involved in the rate determining step of the reaction.

Next the scope of the aldehyde component was investigated. Electron donating (OMe), neutral (Me) and withdrawing (CF_3_) substituents were probed at *ortho‐, meta‐* and *para‐*positions in a 3×3 array (Scheme 2b), revealing electron‐rich substrates tended to be most reactive. Symmetrical 4‐methoxybenzaldehyde gave an 87 % combined yield of mono and di‐functionalization (1 : 1.1 mono : di). Substrates with the methoxy group in the *ortho‐* and *meta*‐positions gave slightly reduced yields. This contrasts with the Me and CF_3_ substituted systems, where increased yield was observed with *ortho*‐substitution. Electron poor derivatives gave lower overall yields and reduced di‐functionalization. The reaction was successfully scaled for the multigram synthesis of **3** **a** in excellent yield (2.07 g **3** **a**, 75 %).

A broader range of aldehydes was then investigated (Scheme 2c). Benzaldehyde itself gave good yield of combined mono and di‐functionalized products (73 % **13** **a**, mono:di 1.3 : 1). 2,3‐Dimethyl and 2,4‐dimethyl benzaldehydes reacted to give **14** **a** and **15** **a** in moderate to good yields. Benzyl protected phenol was tolerated, as was an O‐allyl substituent, a challenging substrate in a palladium mediated process due to allylic activation. 2‐Phenylbenzaldehyde, 2‐napthaldehyde and 6‐methoxy‐2‐napthaldehyde were particularly effective, giving **18** **a**, **19** **a** and **20** **a** in 87 %, 96 % and 71 % yield respectively. Halogenated examples were tolerated without cross‐coupling occurring. In substrates possessing additional potential directing sites (**23** **a**–**28** **a**), C−H sulfonylation was only observed at the position *ortho* to the aldehyde due to the superior directing ability of the transient directing group. Ester **23** **a** was tolerated without any observed hydrolysis. Amide‐containing **24** **a** and benzyl alcohol containing **25** **a** were prepared in 48 % and 51 % yield respectively. The presence of a coordinating sulfonamide group at the 3‐position gave exclusive sulfonylation at the most hindered 2‐position (**26** **a**) indicating a tertiary directing effect of the NHSO_2_Me group. The Cu‐mediated conditions also gave sulfonylated pyridine‐4‐carboxaldehyde and 2‐methoxypyridine‐4‐carboxaldehyde (**27** **a**, **28** **a**). A substrate derived from estrone underwent preferential sulfonylation reaction adjacent to the aldehyde functional group in the presence of the ketone (**29** **a**), using the aldehyde as the limiting reagent. The reaction of 9‐phenanthene carboxaldehyde gave *peri*‐sulfonylated product **30** **a** exclusively, favoring the required [6,6]‐cupracyclic intermediate over the potential *ortho*‐sulfonylation. In the case of 1‐napthaldehyde, *peri*‐functionalization was again the major product in a 2 : 1 ratio with the less hindered *ortho*‐product. On blocking the *ortho*‐position with a methyl group, reduced *peri*‐reactivity was observed.

**Scheme 3:**
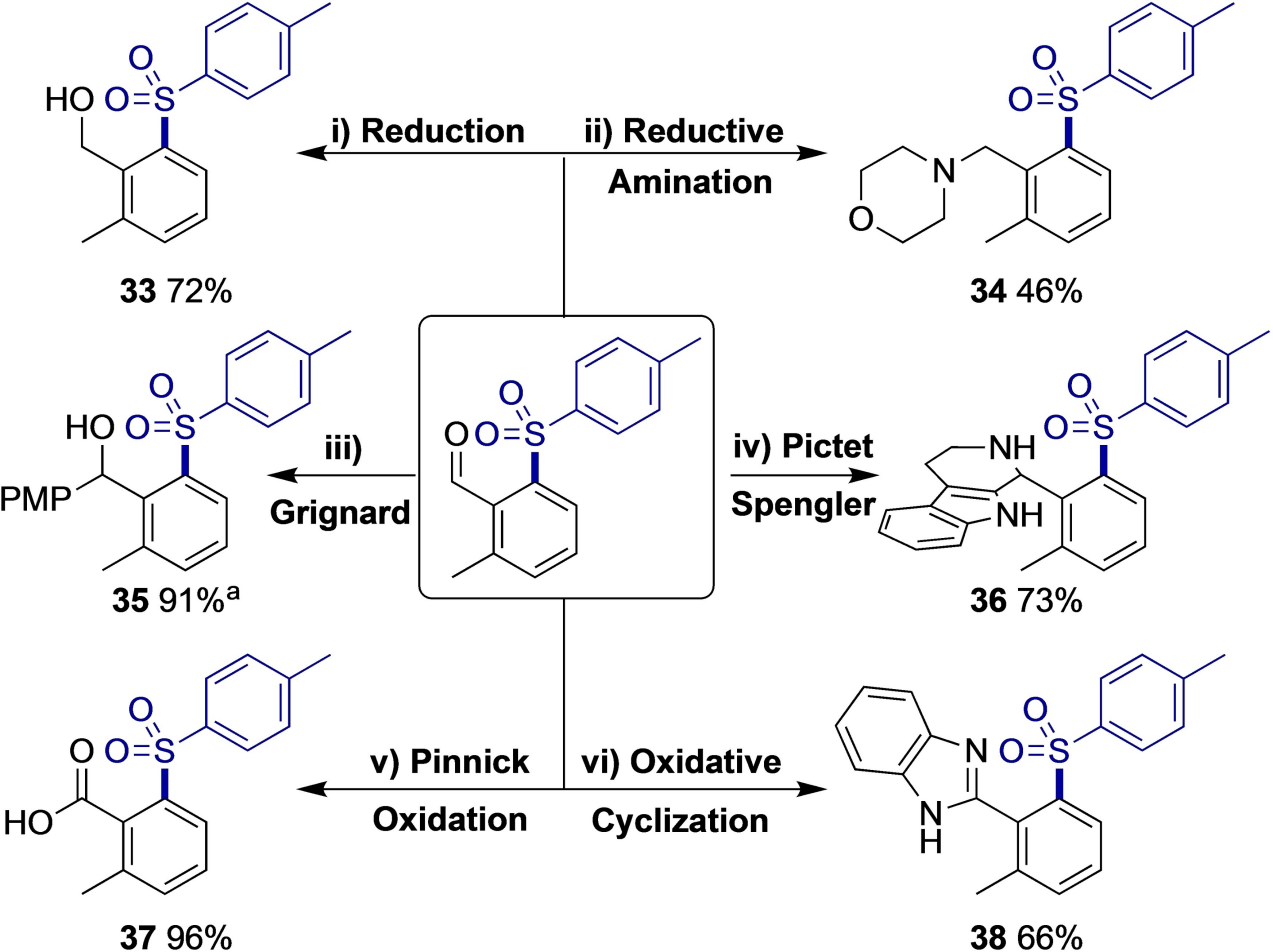
Derivatisation of aldehyde product. Reactions performed on 0.2 mmol scale. Isolated yield reported. i) LiBH_4_, THF, 65 °C, 15 min. ii) NaHB(OAc)_3_, morpholine, CH_2_Cl_2_, rt, overnight. iii) 4‐MeOC_6_H_4_MgBr, THF, 0 °C to rt, overnight. iv) Tryptamine, HFIP, 60 °C, 48 h. v) NaClO_2_, H_2_O_2_ (30 % in H_2_O), NaH_2_PO_4_, MeCN : H_2_O, 0 °C to rt, 3 h. vi) *o*‐Phenylenediamine, CAN (10 mol %), H_2_O_2_ (30 % in H_2_O), MeCN, 50 °C, overnight. [a] Reaction performed on 0.1 mmol scale.

The transient C−H functionalisation strategy provides a reactive handle that is immediately available for further diversification reactions without requiring unmasking of an amide directing group. This was demonstrated by reaction of these sulfonyl aldehydes to provide broader sulfone containing derivatives with motifs of value in medicinal chemistry (Scheme 3). Reduction of the aldehyde with LiBH_4_, gave alcohol **33** and morpholine containing **34** was prepared by reductive amination. The addition of a Grignard reagent furnished secondary alcohol **35** in 91 % yield. Piperidine **36** was synthesised by a Pictet Spengler reaction with tryptamine. Oxidative methods were also suitable to access sulfonyl acid **37** and benzimidazole **38** in excellent yield.

Detailed mechanistic understanding of the often complex copper mediated C−H activation reactions remains limited as cupracyclic intermediates are relatively unstable and only a handful of stable organocuprates have been reported to date.^[24]^ Our attempts at direct investigation of the potential imine coordinated organocuprate intermediate were unsuccessful. Despite these challenges, we wanted to gain insight into the role and performance of the catalytic transient directing groups and underlying mechanism of the C−H functionalization. Therefore, to interrogate the reaction mechanism kinetic and deuteration experiments were undertaken.

We investigated the catalytic performance of the transient directing group using visual comparison methods developed by Blackmond (RPKA)^[25]^ and Burés (VTNA)[^20c^, ^26^] Same‐excess kinetic experiments can determine if product inhibition or catalyst deactivation occur during a reaction.^[25]^ Pleasingly, deactivation of the amino acid catalyst was not occurring. Instead, an inhibitory effect of the sulfonyl aldehyde product was observed, which slowed reaction progress. Studying the imine formation by ^1^H‐NMR, in the absence of copper, indicated a preference for β‐alanine to form an imine with the starting aldehyde in competition with the sulfonylated product aldehyde (41 : 7 imine of **1** : **3** **a**).^[20d]^ The simple imine formation was unlikely to be the cause of inhibition, therefore, we propose that the inhibitory effect arises from the formation of a copper ligated imine intermediate involving the sulfonyl aldehyde product imine, temporarily removing the catalytic transient directing group from the cycle. This helps to explain why using the aldehyde substrate in excess gave an increased yield, which could help alleviate the inhibition effects by further favouring formation of the starting material imine hence shifting the equilibrium to favour the productive pathway.

**Figure 2:**
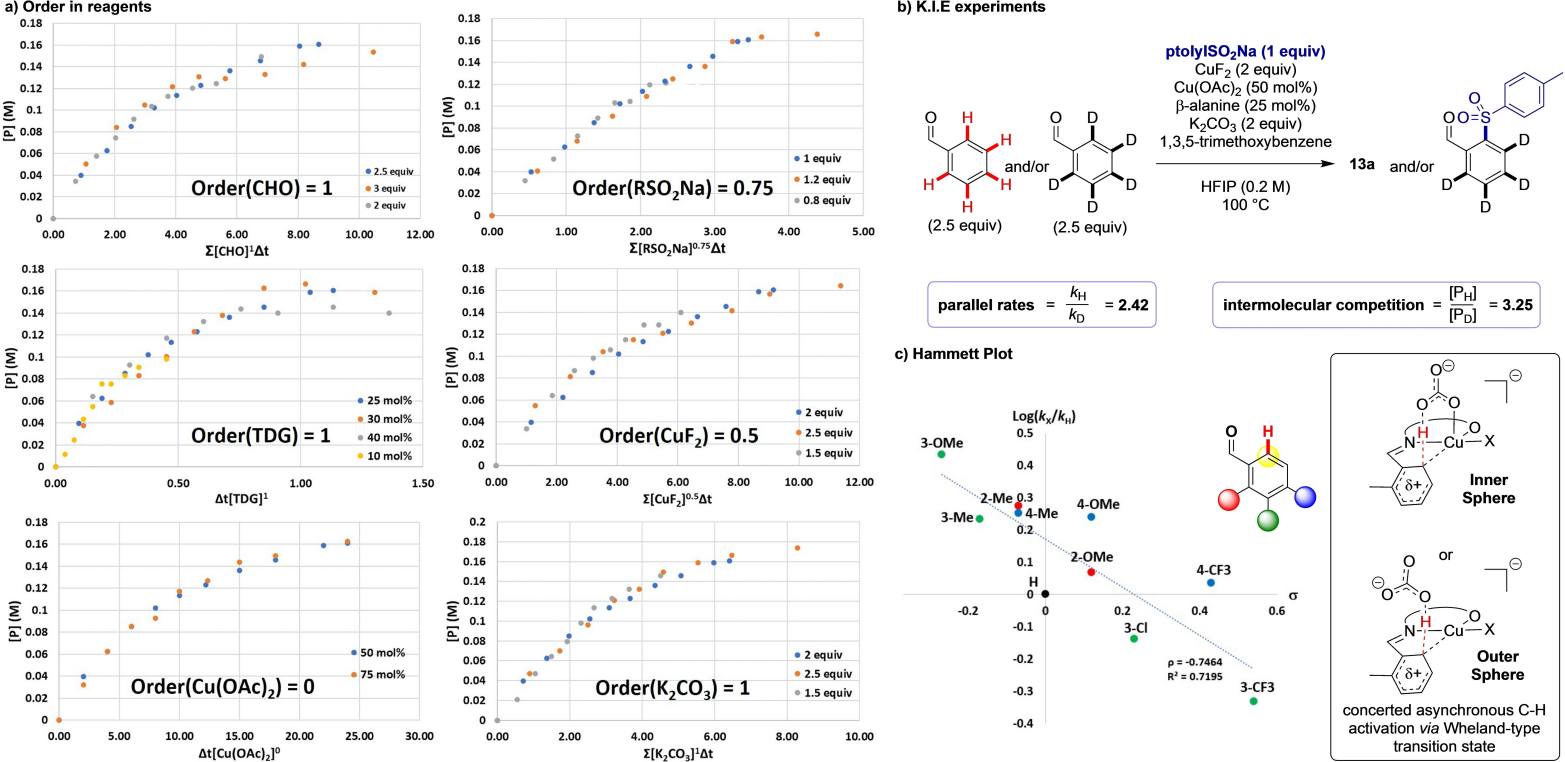
a) VTNA kinetic analysis, graphs of order in reagents. b) K.I.E. experiments using benzaldehyde and benzaldehyde‐d_5_ for parallel and competition reactions c) Hammett plot of varying substituents with proposed electrophilic mode of C−H activation.

Next, variable time normalization analysis (VTNA) was used to estimate the orders in each of the reaction components: the amine catalyst, each copper source, the base and the other reagents (Figure 2a). We identified an order of 1 in aldehyde, transient directing group and K_2_CO_3_. Fractional orders were observed for both the sulfinate and CuF_2_ (0.75 and 0.5 respectively), and a zero order in Cu(OAc)_2_. Together with the optimization studies was clear that the sulfinate has a competing inhibitory effect on the reaction, presumably by blocking copper coordination sites and thus leading to the complex order observed.^[28]^ We hypothesise the role of the Cu(OAc)_2_ is to form Cu(SO_2_R)_2_ in situ and act as a sulfinate reservoir to alleviate inhibition by free sulfinate.

In order to better understand the C−H activation step itself, we employed deuteration and kinetic isotope effect experiments. When performing the reaction in HFIP‐d_2_, no obvious deuteration was observed in either starting material or product, implying that both C−H activation is irreversible under the reaction conditions and there are likely no further cyclometallation events between copper and the product. A significant primary kinetic isotope effect was observed in both parallel (KIE_parallel_=2.42) and competition experiments (KIE_competition_=3.25) which indicates C−H bond cleavage is intimately linked to the turnover limiting step (Figure 2b).^[28]^ Further investigation into the electronics of this step by Hammett analysis revealed a negative correlation (Figure 2c). This implies a build‐up of positive charge on the arene ring in the turnover limiting step, therefore implying an unusual turnover limiting mechanism of C−H activation via a partially dearomatized transition state.^[29]^ Considering these findings together, we propose a concerted asynchronous cleavage of the C−H bond and formation of the C−Cu bond, which involves nucleophilic attack from the aryl π‐system to the copper centre leading to a partial Wheland‐type transition state followed by deprotonation by the carbonate base. This may occur through either an inner or outer sphere mechanism (Figure 2c boxed).

**Figure 3:**
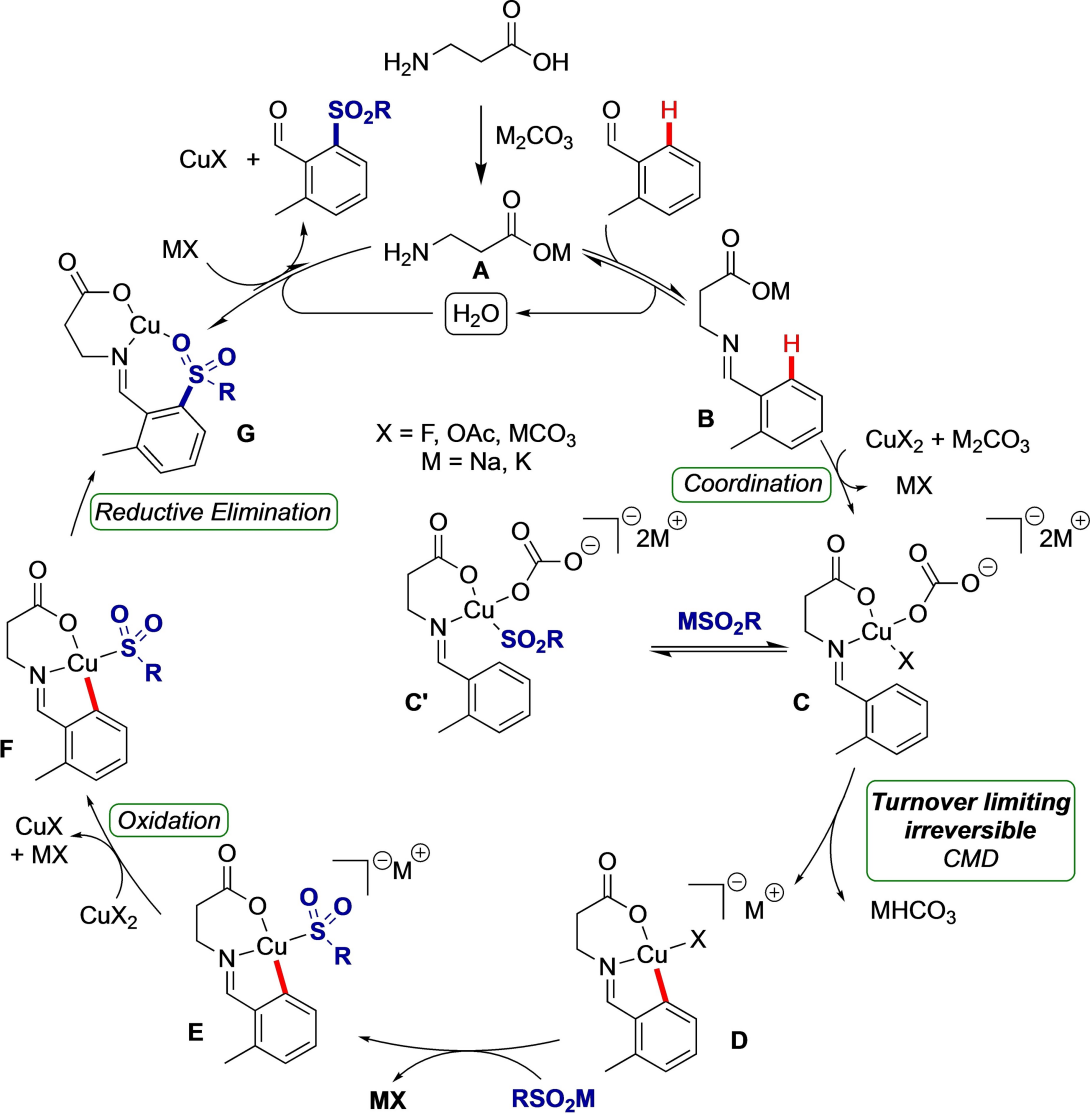
Proposed mechanism of transient C−H sulfonylation, catalytic in transient directing group.

Overall, we propose the following as a plausible mechanism for this transformation (Figure 3). Catalytic amino acid **A** condenses with the aldehyde to form imine **B**. Copper and carbonate coordinate to form intermediate **C**. Coordination of additional sulfinate (**C′**) may result in inhibition of the reaction. Otherwise, **C** undergoes a concerted irreversible and turnover‐limiting C−H activation involving a positive charge build‐up suggestive of an unusual Wheland‐type intermediate and carbonate deprotonation to form **D** with loss of bicarbonate. Ligand exchange with sulfinate would lead to the formation of intermediate **E**. High valent Cu metallocycle **F** would be formed by disproportionation of **E** with another equivalent of copper resulting in a neutral aryl copper species. Reductive elimination from **F** will give copper‐ligated product imine **G**, which must undergo hydrolysis to release the product aldehyde and regenerate the amino acid catalyst. The equilibrium favoring **G** is likely to be the origin of product inhibition trapping the catalytic directing group at high concentrations of product and Cu^I^, rather than the uncoordinated imine.

## Conclusion

In summary, the copper mediated C(sp^2^)‐H sulfonylation of aldehydes has been achieved using catalytic β‐alanine to form a transient directing group. This demonstrates for the first time the potential to use a transient directing group with copper to promote C−H functionalization. The conditions were applied to a wide range of sulfinate salts and aromatic aldehydes including heteroaromatic aldehydes to effect *ortho*‐ or *peri*‐functionalization. As such a broad range of sulfones are readily prepared. The transient directed processes provides new aldehyde derivatives that present useful substrates for further reactions, without requiring additional manipulation to remove directing groups. Kinetic and mechanistic investigations highlighted an unusual turnover‐limiting and partially cationic dearomative C−H activation mechanism. We expect these results will open further possibilities for the use of copper species for C−H functionalization, particularly in combination with transient directing groups, as well as contribute to improved understanding of copper mediated C−H functionalization processes.

## Conflict of interest

The authors declare no conflict of interest.

1

## Supporting information

As a service to our authors and readers, this journal provides supporting information supplied by the authors. Such materials are peer reviewed and may be re‐organized for online delivery, but are not copy‐edited or typeset. Technical support issues arising from supporting information (other than missing files) should be addressed to the authors.

Supporting Information

## Data Availability

The data that support the findings of this study are openly available in Imperial College London Research Data Repository at https://doi.org/10.14469/hpc/8810.
