# Supplementary material for: Amine‐Catalyzed Copper‐Mediated C−H Sulfonylation of Benzaldehydes via a Transient Imine Directing Group
Source: Angew Chem Int Ed Engl. 2022 May 5;61(27):e202202933. doi: 10.1002/anie.202202933 (PMC9321081; doi:10.1002/anie.202202933)
Supplement: Supplementary file 1 — Supporting Information [file ANIE-61-0-s001.pdf]

Angewandte Chemie
Eine Zeitschrift der Gesellschaft Deutscher Chemiker

Supporting Information

Amine-Catalyzed Copper-Mediated C–H Sulfonylation of Benzaldehydes via a Transient Imine Directing Group

*J. I. Higham, J. A. Bull**

Table of Contents

General Experimental S3
Comparison to Prior C(sp^2)–H Sulfonylation Reports S4
Optimisation of Reaction Conditions for Copper Mediated C(sp^2)–H Sulfonylation S5
Copper source S5
Base S6
Solvent S7
Transient directing group S8
Ligand additive S9
Interaction of copper and transient directing group loading S10
Effect of changing sulfinate salt loading S11
Attempts at achieving a protocol catalytic in copper S12
Optimisation using 2-methylbenzaldehyde as substrate S13
Copper source S14
Additional carboxylate additive S15
Aldehyde in excess S16
Design of Experiment (DoE) Optimisation S17
Mechanistic Investigation S23
Kinetics S23
Same Excess S24
Different Excess S27
Imine formation in proteo HFIP S34
Competition experiment S37
Synthesis of Sulfinate Salts S43
General Procedure A: Synthesis of Sulfinate Salts S43
Copper Mediated C(sp^2)–H Sulfonylation S44
General Procedure B: Lab humidity<75% S44
General Procedure C: Lab humidity>75% S44
Reaction Scope Varying the Sulfinate Salt S45
Reaction Scope Varying the Aldehyde S49
Estrone Derived S61
Procedure for Multi Gram Scale Synthesis of Sulfonyl Aldehyde 3a S68
Unsuccessful Substrates S69
^{1}H and ^{13}C Spectra of Selected Compounds S70

General Experimental

All reactions were run under an inert atmosphere (argon) with flame-dried glassware using standard techniques unless otherwise stated. Anhydrous solvents were obtained by filtration through drying columns (THF, diethyl ether, CH_2Cl_2, DMF). $Cu(OAc)_2$ (98%, product code: B23615) and anhydrous CuF_2 (99.5%, product code: 11489) were obtained from Alfa Aesar and used as provided. β-Alanine (99%, product code: 146064) was obtained from Sigma–Aldrich and used as provided. Potassium carbonate (99.5%, product code: 024862) was obtained from Fluorochem and used as provided. Liquid commercial aldehydes were distilled prior to use. Solid aldehydes with boiling points >300 °C were dissolved in CH_2Cl_2, washed with 1 M NaOH, dried over Na_2SO_4, filtered, then concentrated *in vacuo*. All other commercial reagents were used as supplied or purified by standard techniques where necessary. All C–H activation reactions were performed in microwave vials sealed with Fisherbrand™ 20 mm aluminium, plain, centre hole, molded septa butyl, dark grey, 55° shore A, 3.0 mm caps if using high boiling solvents (>100 °C). Fisherbrand™ 20mm Crimp Seal, Gold, Magnetic Cap, 8mm Center hole, assembled septum, molded septa butyl, dark grey, 55° shore A, 3.0 mm caps were used with lower boiling solvents (<100 °C) if heating significantly above their boiling point.

Flash column chromatography was performed using 230-400 mesh silica with the indicated solvent system according to standard techniques. Analytical thin-layer chromatography (TLC) was performed on precoated, glass-backed silica gel plates. Visualisation of the developed chromatogram was performed by UV absorbance (254 nm), aqueous potassium permanganate, *p*-anisaldehyde, phosphomolybdic acid or vanillin stains. Infrared spectra (ν_{max}, FTIR ATR) were recorded in reciprocal centimeters (cm^{-1}). Nuclear magnetic resonance (NMR) spectra were recorded on 400 MHz spectrometers. Chemical shifts for ^1H NMR spectra are recorded in parts per million from tetramethylsilane with the solvent resonance as the internal standard (chloroform δ = 7.27 ppm). Data is reported as follows: chemical shift [multiplicity (s = singlet, d = doublet, t = triplet, q = quartet, hept = heptet, m = multiplet and b = broad), coupling constant in Hz, integration, assignment]. ^{13}C NMR spectra were recorded with complete proton decoupling. Chemical shifts are reported in parts per million from tetramethylsilane with the solvent resonance as the internal standard (chloroform: δ = 77.00 ppm). *J* values are reported in Hz. Assignments of $^1H/^{13}C$ spectra were made by the analysis of δ/J values, and COSY, HSQC, and HMBC experiments as appropriate. ^{19}F NMR spectra were recorded without complete proton decoupling unless otherwise stated. ^{19}F NMR spectra are indirectly referenced to $CFCl_3$ automatically via direct measurement of the absolute frequency of the deuterium lock signal by the spectrometer hardware. Melting points are uncorrected. Due to the PTFE lining in the NMR probe, there can be artifacts observed in the baseline of ^{19}F NMR.

The high-resolution mass spectrometry (HRMS) analyses were performed using electrospray ion source (ESI) or pneumatically assisted atmospheric pressure chemical ionization (APCI) using an atmospheric solids analysis probe (ASAP). ESI was performed using a Waters LCT Premier equipped with an ESI source operated in positive or negative ion mode. The software used was MassLynx 4.1. This software does not account for the electron and all the calibrations/references are calculated accordingly, i.e. $[M+H]^+$ is detected and the mass is calibrated to output [M+H]. APCI was performed using an Orbitrap XL or Xevo G2S using an ASAP to insert samples into the APCI source. The sample was introduced at ambient temperature and the temperature increased until the sample vaporised.

All Data for this manuscript can be found at the Imperial College London Research Data Repository: DOI:10.14469/hpc/8810 (https://doi.org/10.14469/hpc/8810)

Comparison to Prior C(sp^2)–H Sulfonylation Reports

Representative prior examples of cross-coupling and amide directed C(sp^2)–H sulfonylation strategies for the synthesis of sulfones are summarised below **(Scheme S1)**. Cross-coupling strategies rely on pre-functionalised starting materials in the form of aryl iodides and boronic acids, detailed are two methods, one demonstrating the three component coupling of aryl iodides and boronic acids, and the other achieving the oxidative coupling of boronic acids and sulfinate salts **(Scheme S1a).**[1a,b] Previous amide directed C(sp^2)–H sulfonylation methods predominantly use stoichiometric copper salts as both metal salt and oxidant **(Scheme S1b).**[2a,c] It is possible to instead use superstoichiometric silver salts to render the reaction catalytic in copper, this is non ideal as copper is cheaper and more abundant than silver.[2b] This transient C(sp^2)–H sulfonylation method offers greater step efficiency (1 step vs 3 steps), is amenable to late stage functionalisation and by transforming the aldehyde moiety, complex derivatives are easily accessible **(Scheme S1c)**.

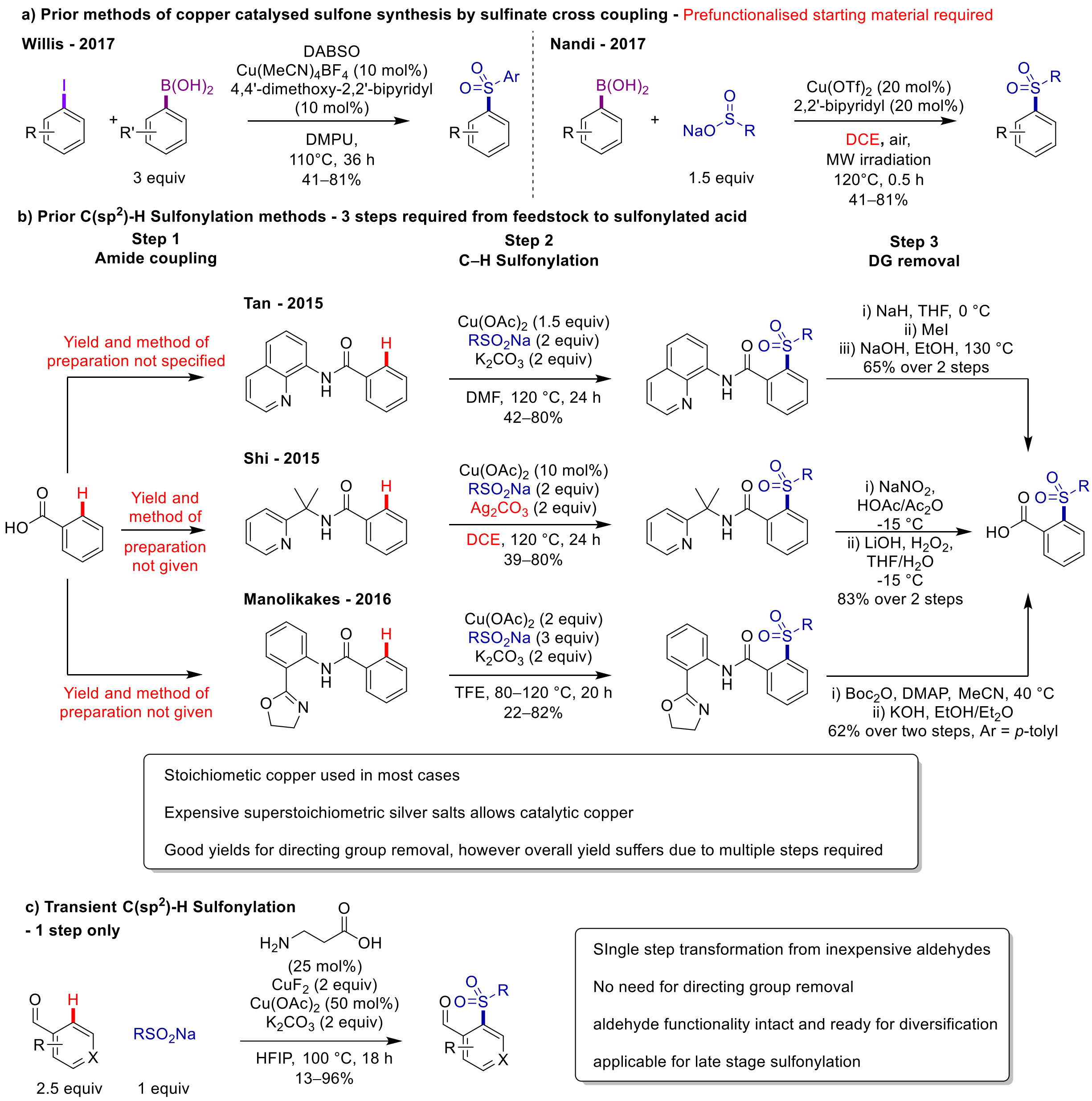

Scheme S1 – Comparison of prior methods of sulfone synthesis to this report of transient C(sp^2)–H Sulfonylation.

Optimisation of Reaction Conditions for Copper Mediated C(sp^2)–H Sulfonylation

Copper source

Initial studies were conducted using benzaldehyde as the substrate with reactions run under air **(Table S1)**. Initial optimisation found copper acetate was the most effective and economical copper source.

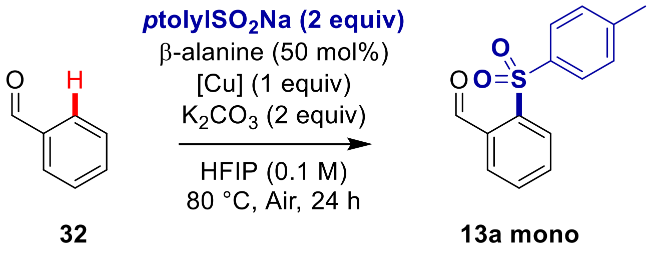

Entry	[Cu]	Sulfonylated Aldehyde (%)[a]
1	$Cu(OAc)_2$	11
2	$Cu(OTf)_2$	11
3	$CuBr_2$	0
4	$CuSO_4$	0
5	$Cu(MeCN)_4PF_6$	7

Table S1 – Effect of copper source. [a]Yields determined by ^{1}H NMR using 1,3,5-trimethoxybenzene as an internal standard.

Base

Several bases were screened, many appeared to be effective however none more so than K_2CO_3 thus this base was retained as the optimal base **(Table S2)**. The reduced efficacy of Cs_2CO_3 could due to the more hygroscopic carbonate drawing water into the reaction. We have observed this reaction is sensitive to water, thus this could be the reason for reduced reactivity with Cs_2CO_3 (Page S20, Table S16, Entry 4).

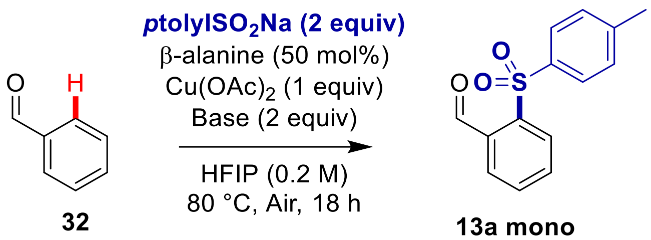

32 **13a mono**

Entry	Base	Sulfonylated Aldehyde (%)[a]
1	K_3PO_4	9
2	K_2CO_3	16
3	KOAc	9
4	KTFA	0
5	DBU	9
6	NEt_3	7
7	Pyridine	9
8	KF	12
9	Cs_2CO_3	trace

Table S2 – Effect of changing base. [a]Yields determined by ^1H NMR using 1,3,5-trimethoxybenzene as an internal standard.

Solvent
Other solvents and mixed solvent systems were trialled; however only HFIP was effective in promoting the reaction **(Table S3)**.

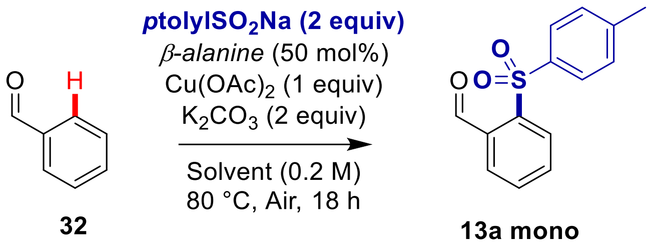

Entry	Solvent	Sulfonylated Aldehyde (%)[a]
1	DMSO	0
2	TFE	trace
3	MeCN	0
4	AcOH	0
5	CH_2Cl_2	0
6	MeOH	0
7	HFIP	15
8	HFIP:MeOH (1:1)	8
9	HFIP:EtOH (1:1)	Trace
10	HFIP:H_2O (1:1)	0
11	HFIP:MeCN (1:1)	0
12	HFIP:DMSO (1:1)	0

Table S3 – Effect of changing solvent. [a]Yields determined by ^{1}H NMR using 1,3,5-trimethoxybenzene as an internal standard.

Transient directing group

Initial investigation into TDG found b-alanine was effective. On testing more [5,6] chelating TDGs, β-alanine remained the most effective **(Scheme S2)**.

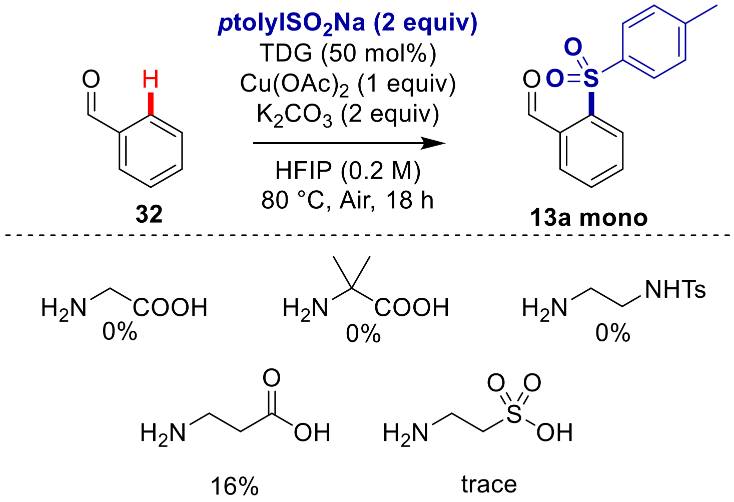

Scheme S2 – Effect of changing transient directing group. Yields determined by ^{1}H NMR using 1,3,5-trimethoxybenzene as an internal standard.

Further investigation into 5,6 chelating directing groups confirmed that β-alanine was the most effective. Additionally, a raised temperature led to a slight increase in yield **(Scheme S3)**.

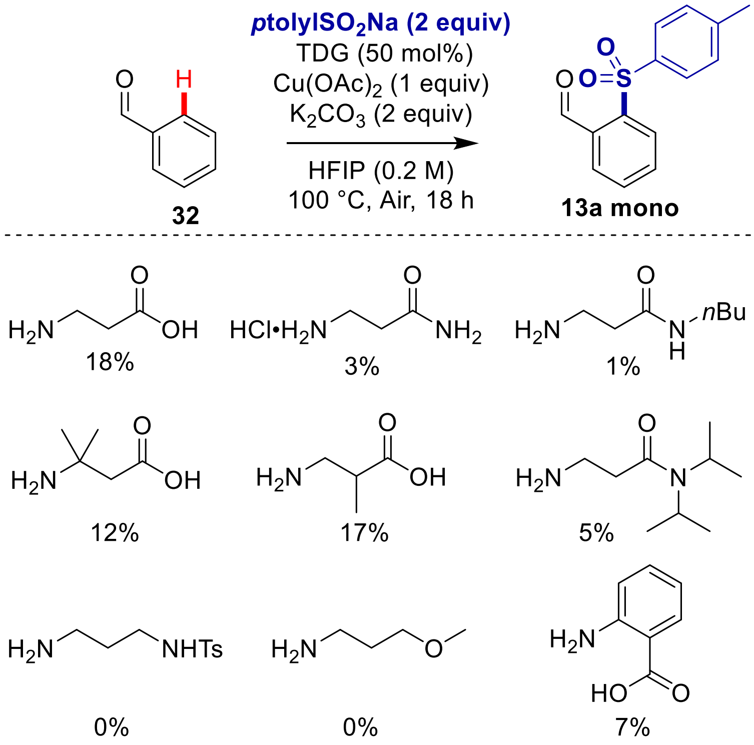

Scheme S3 – Further TDG investigation. Yields determined by ^{1}H NMR using 1,3,5-trimethoxybenzene as an internal standard.

Ligand additive

Additional additives were tested as possible ligands for copper, however we found additional ligand additives did not improve the reaction **(Scheme S4)**.

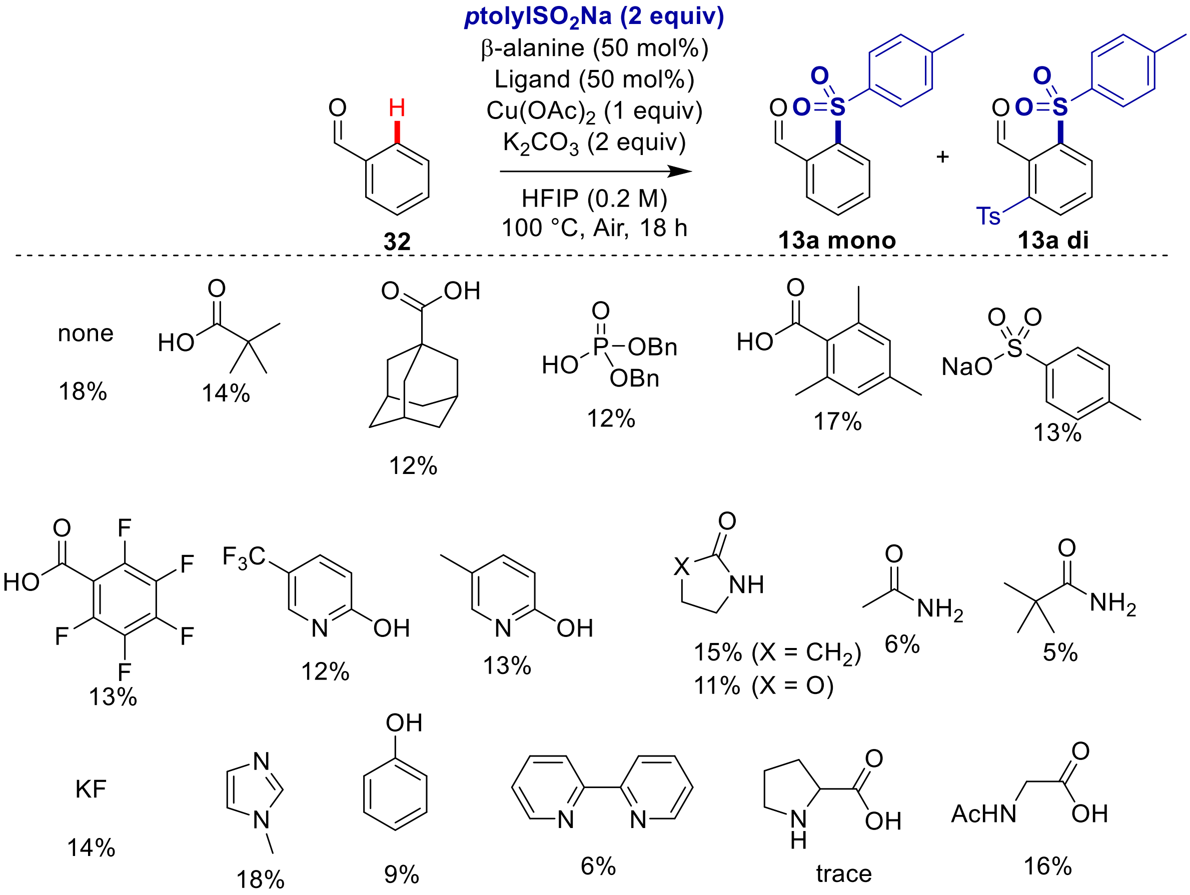

Scheme S4 – Effect of ligand additive. Yields shown are combined yield of Mono+Di. [a]Yields determined by ^{1}H NMR using 1,3,5-trimethoxybenzene as an internal standard.

Interaction of copper and transient directing group loading

We found that when investigating the loading of TDG and copper, highest yield was observed at 25 mol% TDG and with increased Cu loading (2 equiv) **(Scheme S5)**.

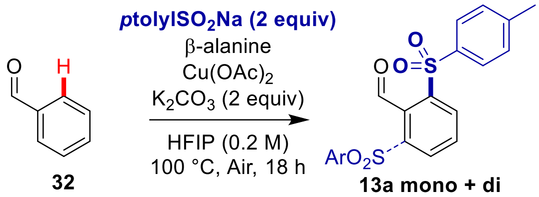

32 **13a mono + di**

equiv Cu \ β-alanine Loading (mol%)	5	10	25	50	100
0.25			5%	trace	0%
0.5	6%	8%	14%	12%	7%
1	8%	9%	17%	16%	12%
1.5	5%	8%	19%		
2	5%	7%	26%		
2.5			23%		

Mono+Di: 0-10% (red); 11-20% (orange); 21-30% (yellow); 31-40% (cyan)

Scheme S5 – Effect of changing both TDG and copper loading. Yields determined by ^{1}H NMR using 1,3,5-trimethoxybenzene as an internal standard.

Effect of changing sulfinate salt loading

The sulfinate salt loading was found to be important, with a lower loading of 1 equiv promoting the reaction **(Table S4)**.

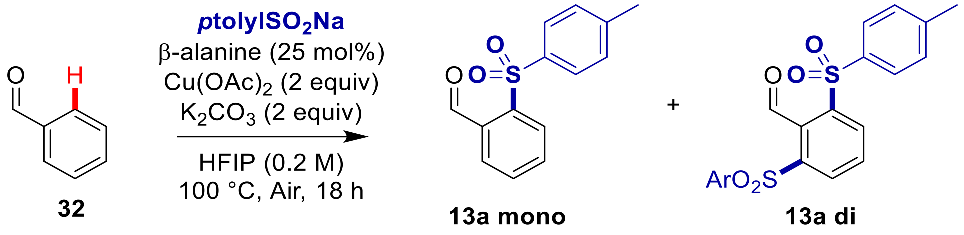

Entry	Equivalent of sulfinate salt	Yield sulfone (Mono+Di) (%)
1	2	22
2	3	23
3	4	22
4	1	30

Table S4 – Effect of sulfinate salt loading. [a]Yields determined by ^{1}H NMR using 1,3,5-trimethoxybenzene as an internal standard.

Attempts at achieving a protocol catalytic in copper

Attempts to achieve a protocol using catalytic copper were unsuccessful. The inclusion of additional oxidants was investigated, but these generally supressed the reaction **(Table S5)**.

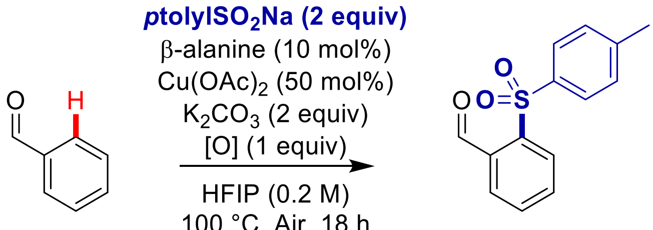

32 **13a mono**

Entry	[O]	Yield aldehyde (%)	RSM (%)
1	none	5	83
2	1-fluoro-2,4,6-trimethylpyridinium triflate	0	31
3	potassium persulfate	9	71
4	iodine	0	81
5	diacetoxyiodobenzene	0	64
6	MnO_2 + 2 equiv AcOH	0	81
7	O_2 atmosphere	0	-

Table S5 – Attempt at protocol catalytic in copper. [a]Yields determined by ^{1}H NMR using 1,3,5-trimethoxybenzene as an internal standard.

Optimisation using 2-methylbenzaldehyde as substrate

Further optimisation was conducted on 2-methylbenzaldehyde in order to simplify the product distribution to gain an easier understanding of the system reactivity.

Sulfinate Equivalents

The equivalent of sulfinate salt was re–investigated and a minor increase in yield was observed at 1.25 equiv of sulfinate salt **(Table S6)**.

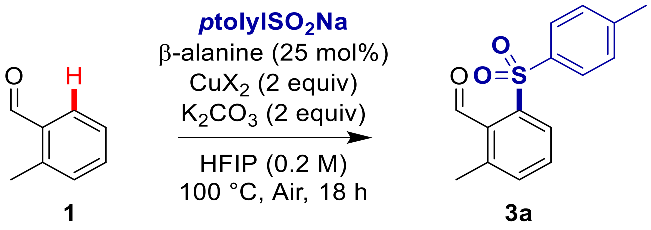

Entry	Equivalent of sulfinate	Yield aldehyde 3a (%)	RSM (%)
1	1.00	33	45
2	1.25	38	46
3	1.50	37	44

Table S6 – Re-investigation of sulfinate loading. Yields determined by ^{1}H NMR using 1,3,5-trimethoxybenzene as an internal standard.

Copper source

Further investigation into copper sources led to CuF_2 being identified as more effective **(Table S7)**. The higher hygroscopicity of this copper source, and variable lab humidity, led to a variation in yield correlating with varying humidity. We found that the reaction was more reliable when an argon atmosphere was used, and when the base and copper source were flame dried in the vial prior to other components being added. This copper source was used in further optimisation.

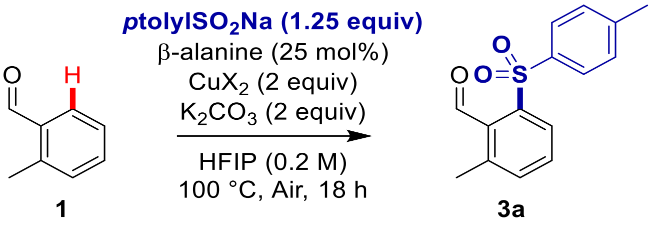

Entry	Cu	Yield aldehyde 3a (%)	RSM (%)
1	$Cu(OAc)_2$	37	60
2	$Cu_2CO_3(OH)_2$	trace	92
3	$Cu(acac)_2$	0	100
4	$Cu_3(PO_4)_2$	0	90
5	CuO	Trace	67
6	$CuSO_4 \cdot 5H_2O$	12	86
7	CuF_2	35–50	46–55
8[a]	CuF_2	51	41
9	$CuCl_2$	18	43
10	$Cu(OPiv)_2$	29	65
11	$Cu(OBz)_2$	18	74

Table S7 – Further of Copper source investigation. Yields determined by ^{1}H NMR using 1,3,5-trimethoxybenzene as an internal standard. [a] Reaction run under argon atmosphere, flame drying base and copper source.

Additional carboxylate additive

When using CuF_2, additional carboxylate ligand additives led to increased yield. This effect could be exploited by adding $Cu(OAc)_2$ to lower the loading of CuF_2 while maintaining the higher yield **(Table S8)**.

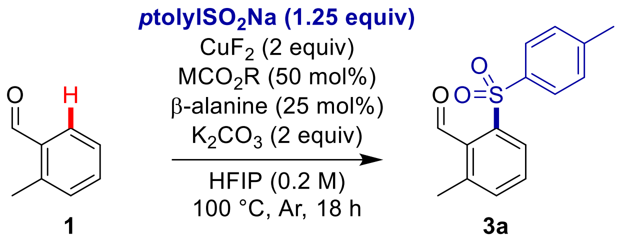

**Entry**	MCO_2R	**Yield aldehyde (%)**	**RSM (%)**
1	none	53	36
2	TBAOAc	63	41
3	KOAc	63	37
4	KOPiv	57	41
5	KTFA	62	24
6	CuF_2 (1.5 equiv),<br>$Cu(OAc)_2$ (50 mol%)	64	33

Table S8 – Effect of Carboxylate additives. [a]Yields determined by ^1H NMR using 1,3,5-trimethoxybenzene as an internal standard.

Aldehyde in excess

It was possible to further increase the yield by using the aldehyde in excess, with 78% yield accessible when using the aldehyde in 2 equiv **(Table S9)**.

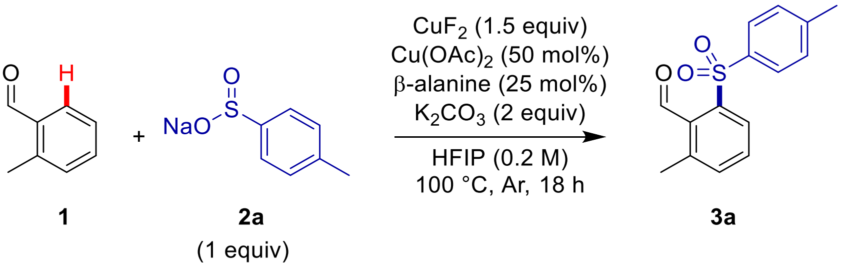

Entry	Equiv of aldehyde	Yield aldehyde (%)	RSM (%)
1	1 (1.25 equiv Sulfinate salt)	64	33
2	1.25	62	42
3	1.5	63	59
4	1.75	73	90
5	2	78	116

Table S9 – Aldehyde in excess. [a]Yields determined by ^1H NMR using 1,3,5-trimethoxybenzene as an internal standard.

Design of Experiment (DoE) Optimisation

We used a design of experiment (DoE) approach in order to evaluate the interactions and main factors influencing the yield. JMP pro 14 DoE software was used using a 'custom design', focusing on the following variables: aldehyde loading, CuF_2 loading and reaction concentration, in the constraints shown. All other variables were fixed. The DoE data is shown **(Table S10)**.

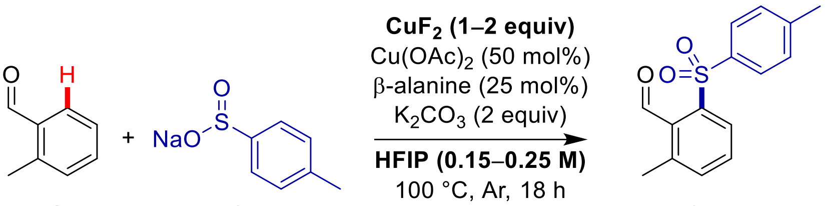

1 **1.5–2.5 equiv** **2a** (1 equiv) **3a**

Entry	CHO Loading (equiv)	CuF_2 Loading (equiv)	Conc. (M)	Yield (%)[a]
1	2.5	1.5	0.2	83
2	2	1	0.2	61
3	2	1.5	0.25	72
4	1.5	1	0.15	54
5	2.5	2	0.25	87
6	1.5	1	0.25	62
7	1.5	1.5	0.15	69
8	1.5	2	0.25	66
9	2.5	1	0.25	61
10	1.5	2	0.2	73
11	2.5	1	0.15	54
12	2	1.5	0.2	77
13	2	1.5	0.2	77
14	2	2	0.15	82
15	2.5	2	0.15	87
16	2	1	0.2	63
17	2	1.5	0.25	73

Table S10 – DoE data. [a]Yields determined by ^1H NMR using 1,3,5-trimethoxybenzene as an internal standard.

From these results, the JMP software generated a model to predict the changes in yield with changes in the above variables (and their interactions). The actual by predicted plot show the data matches well with the model generated, without any major outliers **(Figure S1)**. Additionally, the effect summary highlights CuF_2 loading, CHO loading, and their interaction and the interaction between CuF_2 loading and concentration as most significant **(Figure S1)**.

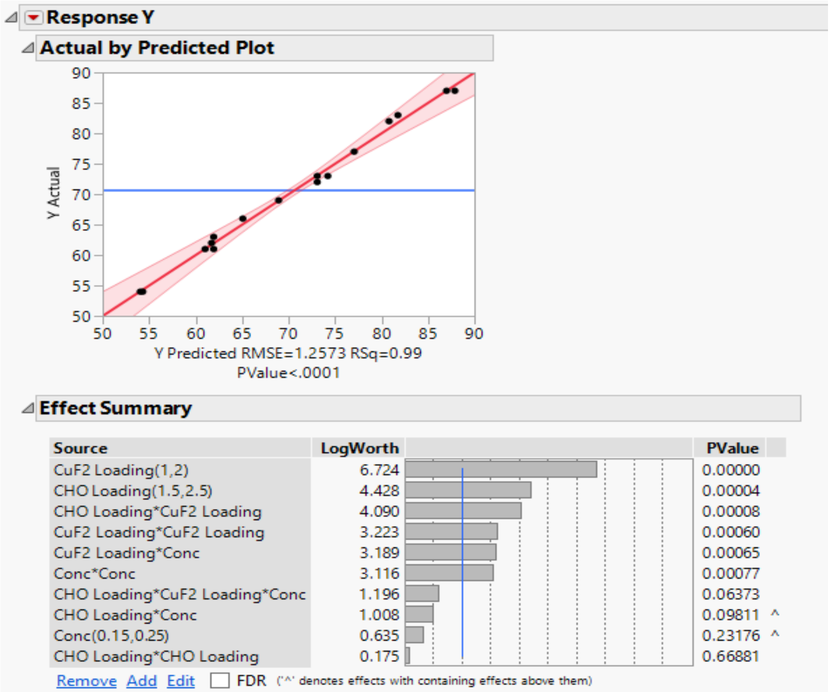

Figure S1 – Actual by predicted plot and effect summary.

The interactions shown in **figure S2** can be summarised as the following:

B1: Improved reactivity at high CuF_2 and CHO loading

C1: No strong interaction

A2: Increased reactivity when raising CHO loading only when CuF_2 loading is high

C2: Slight interaction, lower yield at lower loading of CuF_2

A3: No strong interaction

B3:Slight interaction, higher reactivity using more CuF_2 at lower conc.

Overall, the most interesting interaction is that of the CHO loading and CuF_2 loading, as higher yields are only observed when both are increased **(Figure S2, A2)**. If the CuF_2 loading is 1 equiv, the yield is independent of the aldehyde loading.

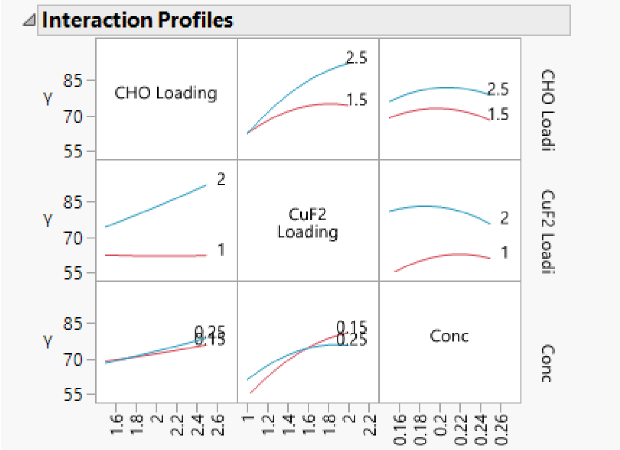

Figure S2 – Interaction plot

In addition, the DoE model prediction could be plotted to give the following surface with CuF_2 loading and CHO loading as the X and Y axis respectively as the most important variables (Conc is set to 0.2 M). Clearly there is a maximum at 2 equiv CuF_2 and 2.5 equiv aldehyde, and this is further backed up by the best conditions predicted by the model shown **(Figure S3, S4)**, which predict 92% if using those conditions.

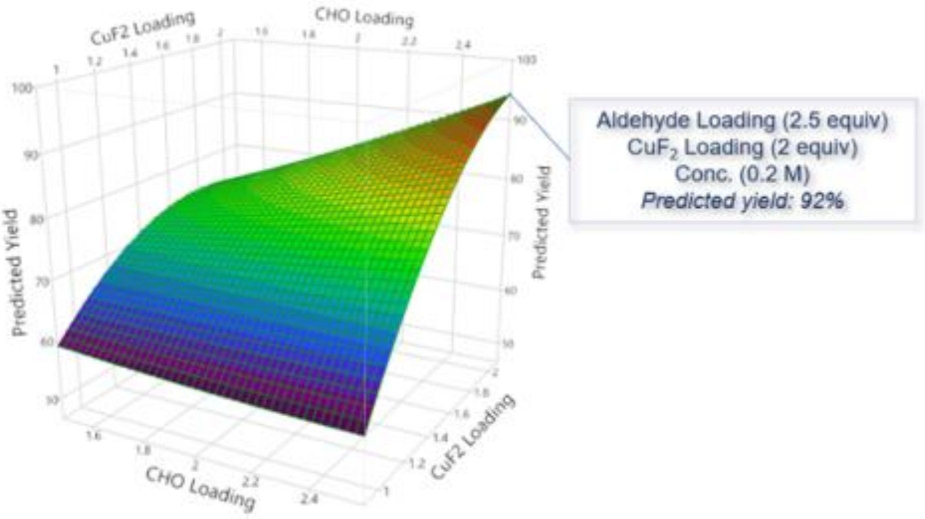

Figure S3 – Response surface, showing CuF_2 loading and CHO loading.

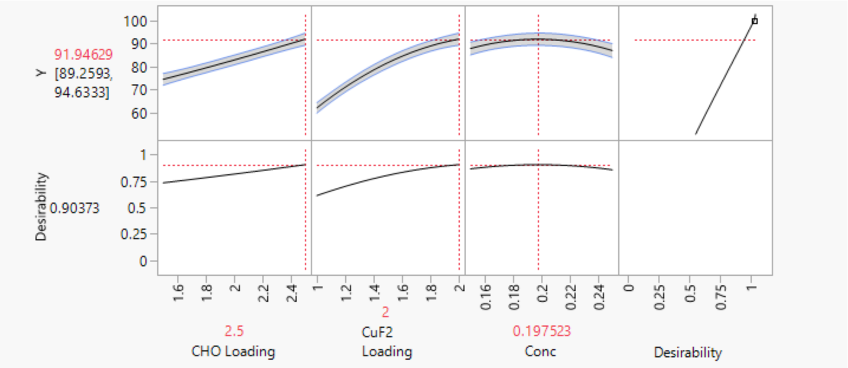

Figure S4 – Best conditions predicted by the DoE model.

The model was validated experimentally by running these 'best conditions' and pleasingly, the model correctly predicted a hotspot, and using the conditions suggested by the DoE gave 93% yield **(Table S11)**. It was also possible to obtain 75% yield using the less expensive copper acetate as the sole copper source in 2.5 equiv, which could be used as a more economical alternative to the standard conditions on a large scale.

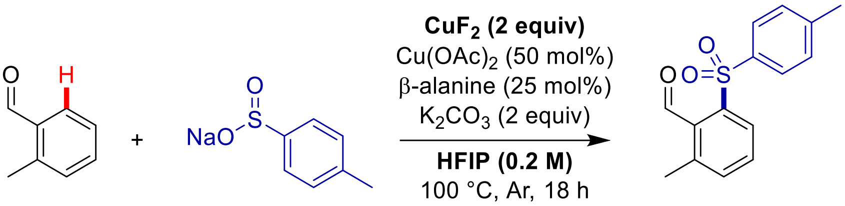

Entry	Change to Conditions	Yield Sulfone (%)	RSM (%)
1	None	93	151
2	None	93	153
3	$Cu(OAc)_2$ (2.5 equiv) instead of CuF_2	75	150

Table S11 – Model validation experiments. Yields determined *in situ* by ^{1}H NMR using 1,3,5-trimethoxybenzene and an internal standard.

Sensitivity Screen

The sensitivity of the reaction under the optimised conditions was evaluated using a method developed by Glorius.[3] We found a particular sensitivity to the presence of water and when the reaction was run under air **(Table S12)**.

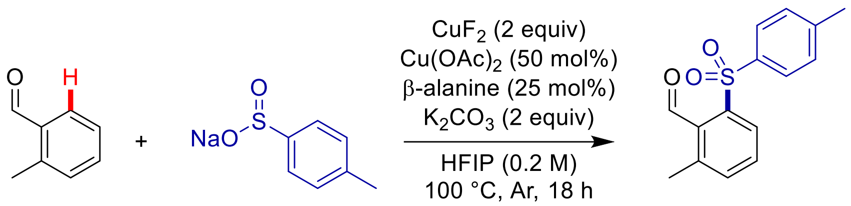

1 **2.5 equiv** **2a** **3a**

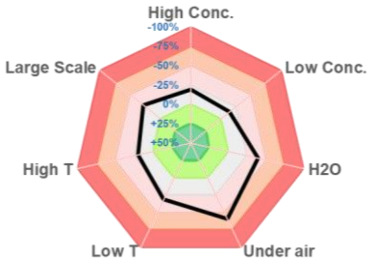

**Entry**	Change	Yield (%)	Deviation (%)
1	None	[a]90	0
2	High C (-10% vol)	73	-19
3	Low C (+10% vol)	77	-14
4	+10 mL H_2O	54	-40
5	high O2 (under air)	37[b]	-59
6	Low T (90 °C)	62	-31
7	High T (110 °C)	72	-20
8	Large scale (2 mmol)	67 (65)	-28

Table S12 – Reaction sensitivity screen. [a]Average of 5 reactions. Yields determined by ^1H NMR using 1,3,5-trimethoxybenzene as an internal standard. Isolated yield in parentheses. [b]11% of HFIP adduct **4** was observed.

HFIP Adduct Formation

In the absence of the sulfinate salt, under otherwise identical reaction conditions the solvent was observed to couple and formed HFIP adduct (**4**) in 33% yield *in situ* **(Scheme S6a)**. Due to the change in limiting reagent in the absence of the sulfinate salt, the equivalents of reagents and loading of catalyst are by definition different, despite the same number mmol of each component being employed. When reducing the amount of aldehyde to 0.2 mmol while keeping the amount of the other components the same (so effectively raising the equivalents of other components), a higher yield of 55% of the HFIP adduct was observed.

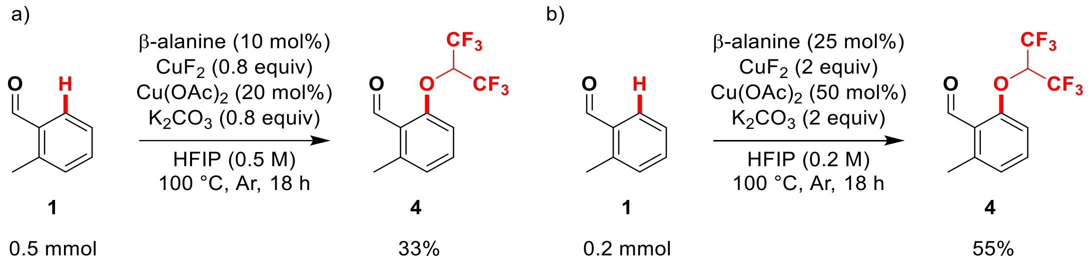

Scheme S6 – Investigation of HFIP adduct formation a) HFIP adduct formation under standard conditions in the absence of sulfinate salt. b) HFIP adduct formation lowering the amount of aldehyde while keeping amounts of other components identical. Yields determined by ^{1}H NMR using 1,3,5-trimethoxybenzene as an internal standard.

Mechanistic Investigation

Kinetics

Procedures for same excess and different excess experiments

Visual comparison methods developed by Blackmond,[4] and Burés,[5] were used to elucidate key mechanistic information from kinetics experiments. Same excess experiments were conducted to determine if catalyst deactivation or product inhibition were occuring in the reaction, and different excess experiments were performed to estimate the orders of each component of the reaction. In all cases, reaction profiles were constructed from the sampling of two 0.5 mmol scale reactions (0.1 mL per aliquot) at the indicated time with a known quantity of internal standard (1,3,5-trimethoxybenzene) added to the reaction. 2–10 h and 24 h timepoints were collected from one reaction, and 12–18 h timepoints were collected from a second reaction. Control experiments showed that the addition of the internal standard had an insignificant effect on the final yield and sampling had no effect on the yield observed under these conditions. Additionally, changing from sampling one reaction to another had no effect and profiles remained smooth. We found the most reproducible set up was using a 25 mL microwave vial submerged in an oil bath just above the solvent line of the reaction in the centre of the hotplate. The stirring rate was set to 440 rpm.

The reaction was less efficient on larger scale, and this was observed when comparing profiles from sampling vs profiles derived from individual experiments (0.2 mmol scale, 500 rpm stirring). However, the difference in yield was minimal, and the added convenience, reliability and reproducibility of the sampling method meant it was chosen for all following kinetics experiments. It was not possible to get reliable data from recovered starting material, as the starting material was found to be volatile enough to be removed when concentrating *in vacuo* so only product formation data was used in the analysis.

A general procedure used for same excess and different excess experiments are outlined below.

Potassium carbonate and copper(II) fluoride were added sequentially to a 25 mL microwave vial which was flame dried under argon until a blue colour just appeared (ca. 2–5 seconds). The microwave vial was allowed to cool to room temperature and copper(II) acetate, β-alanine, *p*-tolylsulfinic acid sodium salt, accurately weighed 1,3,5-trimethoxybenzene (0.3 equiv) and the aldehyde were added to a microwave vial sequentially under argon. The vial was sealed and HFIP (0.2 M) was added and the vial was submerged in an oil bath preheated to 100 °C [Stirring rate set to 440 rpm]. To take an aliquot: at the allotted time the vial was removed from the oil bath and 0.1 mL of solvent was removed by syringe while the reaction mixture was hot. [Caution: As the reaction is above the boiling point of the solvent, there is a small degree of back pressure. This was easily managed by holding the syringe plunger down gently while collecting the sample, and ensuring the liquid was taken up carefully and adequate inert atmosphere was taken up with the sample.] The aliquot was added to a small vial containing a mixture of saturated aqueous ammonium chloride and EtOAc (approx. 1:1). The vial was sealed and shaken until the aliquot was observed to change from brown to blue/green. The organic and aqueous layers were allowed to separate, and the organic layer was carefully removed by pipette then filtered through a small pad of Na_2SO_4 to remove any residual water, then the sample was concentrated *in vacuo*. The entire residue was dissolved $CDCl_3$ (<1 mL) and the yield of sulfonylated material was determined by ^{1}H NMR by comparison with the internal standard.

Timepoints for 2, 4, 6, 8, 10 and 24 h were collected from one reaction and 12, 15, and 18 h were collected from a second reaction. Concentrations were calculated from the total volumes of the parent liquids (aldehyde+HFIP): 2.5 equiv CHO – 2.6445 mL, 3 equiv CHO – 2.6734, 2 equiv CHO – 2.6156.

Control profile was constructed using the above method **(Scheme S7)**, and was used as a comparison for subsequent kinetic experiments.

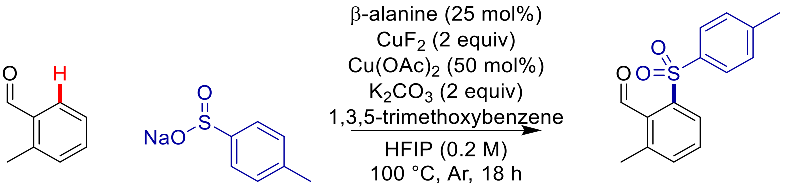

1 1.25 mmol 2.5 equiv

2a 0.5 mmol 1 equiv

3a Max yield 0.5 mmol 0.2 M

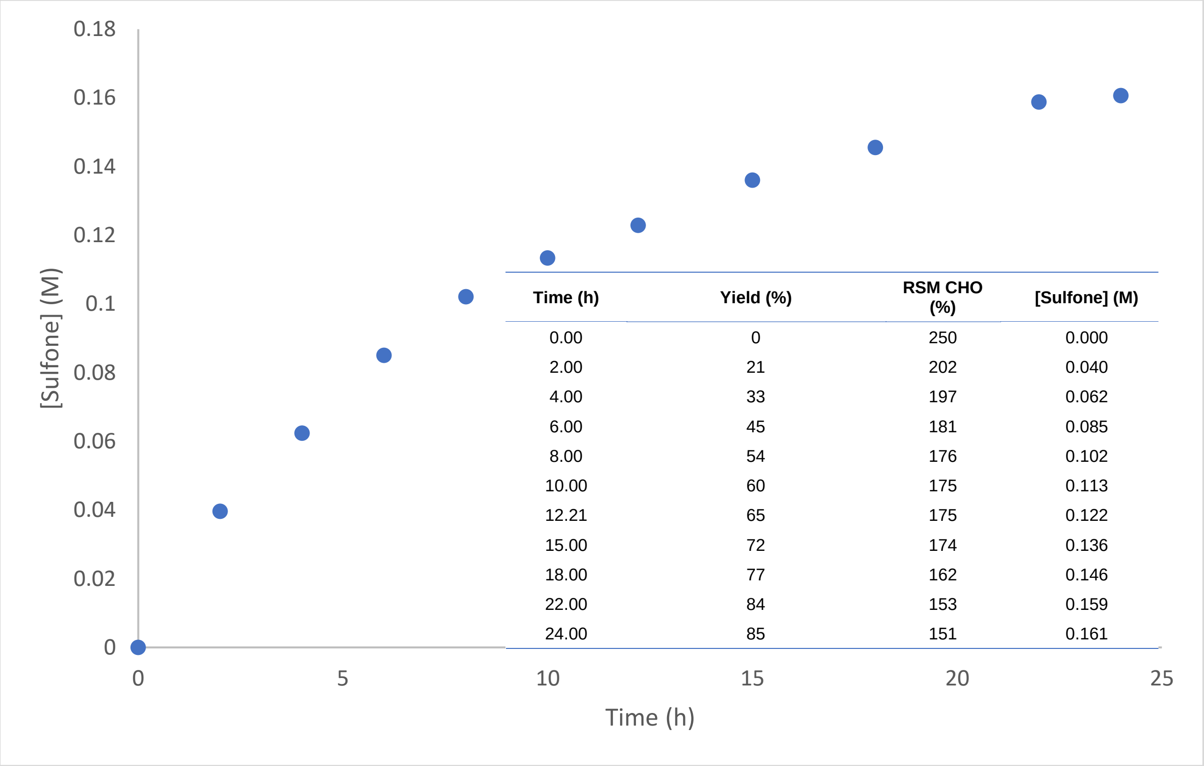

Time (h)	Yield (%)	RSM CHO (%)	[Sulfone] (M)
0.00	0	250	0.000
2.00	21	202	0.040
4.00	33	197	0.062
6.00	45	181	0.085
8.00	54	176	0.102
10.00	60	175	0.113
12.21	65	175	0.122
15.00	72	174	0.136
18.00	77	162	0.146
22.00	84	153	0.159
24.00	85	151	0.161

Scheme S7 – Control profile using sampling procedure.

Same Excess

Same excess experiments were used to determine if catalyst deactivation or product inhibition were occuring in this reaction **(Table S13)**. A 4 h timepoint was selected where the reaction had reached around 35% yield. Another experiment **(Entry B)** was performed in which each component was decreased by the amount that would have been consumed in the reaction until this point, assuming a 1:1 reaction stoichiometry in all cases except the CuF_2, where a 1:2 stoichiometry of sulfinate:CuF_2 is

assumed. Therefore, Experiments B was set up with the following quantities: aldehyde (125 μL, 1.08 mmol), sulfinate salt (59 mg, 0.33 mmol), CuF_2 (67 mg, 0.66 mmol) and K_2CO_3 (115 mg, 0.83 mmol). All catalytic components (TDG and $Cu(OAc)_2$) were assumed not to change in concentration.

To detect product inhibition, two further same experiments were conducted but with either the addition of all the assumed byproducts of the reaction + the sulfone product **(Entry C)** or the addition of only the aldehyde product **(Entry D)**. [note: it was not possible to add copper(I) fluoride in experiment C as this has never been isolated and is not possible to make.]

Entry	Experiment	[CHO] (M)	[Sulfinate] (M)	[CuF_2] (M)	[K_2CO_3] (M)	[P] added (M)	[$KHCO_3$] added (M)	[KF] added (M)
**A**	Control	0.473	0.189	0.378	0.378	0	0	0
**B**	Same excess t = 4 h	0.411	0.126	0.264	0.251	0	0	0
**C**	Same excess t = 4 h + product + $KHCO_3$ + 2KF	0.411	0.126	0.264	0.251	0.065	0.065	0.130
**D**	Same excess t = 4 h + product	0.411	0.126	0.264	0.251	0.065	0	0

Table S13 – Same excess experiments.

Time (h)	Adjusted time (h)	Experiment B [P] Same Excess t = 4 h (M)	[P] Same excess t = 4 h [P] adjusted (M)	Experiment C [P] Same excess t = 4 h + Product 3a + $KHCO_3$ + KF (M)	Experiment D [P] Same excess t = 4 h + Product 3a only
0.00	4.00	0	0.065	0.065	0.065
2.00	6.00	0.032	0.097	0.088	0.080
4.00	8.00	0.055	0.120	0.105	0.099
6.00	10.00	0.070	0.135	0.118	0.112
8.00	12.00	0.082	0.147	0.130	0.124
10.00	14.00	0.095	0.160	0.141	0.135
12.00	16.00	-	-	-	0.143
12.17	16.17	0.101	0.166	-	-
12.50	16.50	-	-	0.141	-
15.00	19.00	0.097	0.162	0.154	0.154
18.00	22.00	0.099	0.163	0.154	0.158
21.50	25.50	0.103	0.168	0.156	-

Table S14 – Data for same excess experiments B, C and D.

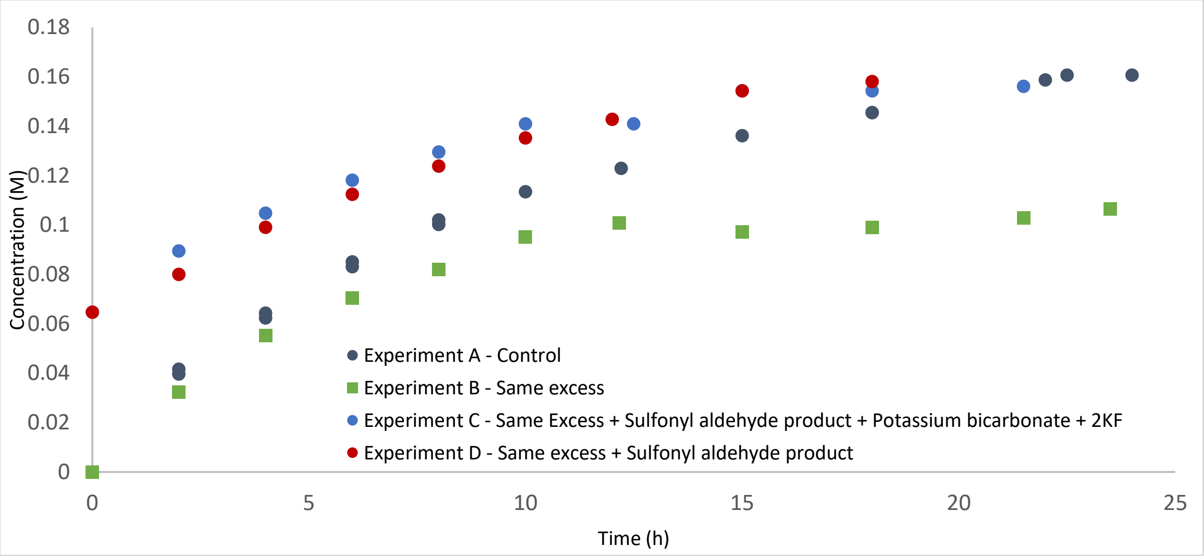

Graph S1 – Product concentration against time for experiments A to D before normalisation for time and product concentration.

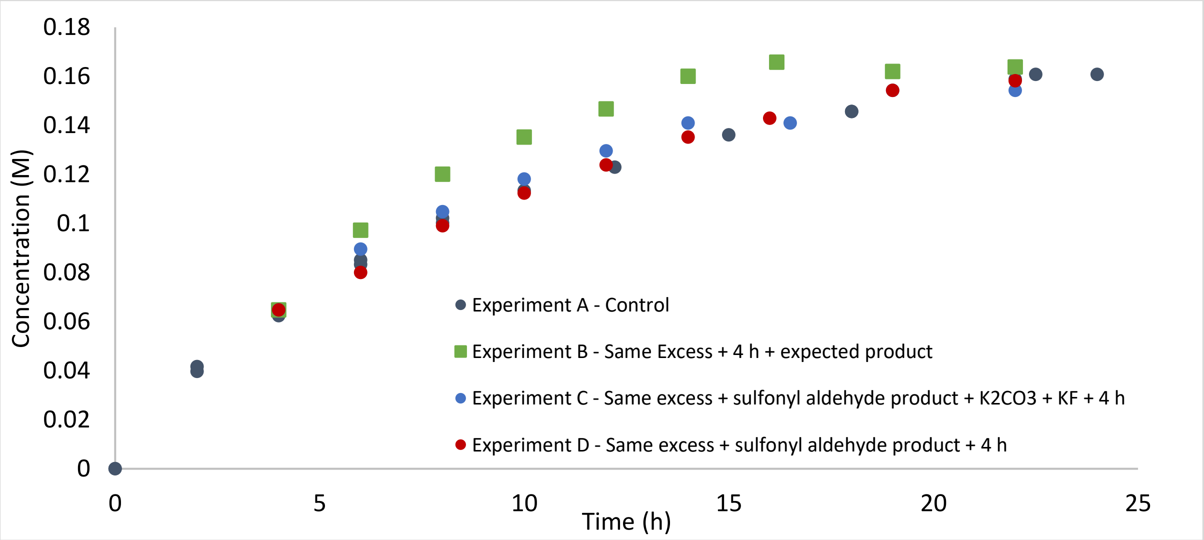

Graph S2 – Time and product normalised graph of product concentration against time for same excess experiments.

The data was normalised by the addition of 4 h to each point of the same excess experiments such that the profiles arising from B,C and D are all time shifted by 4 h. In addition to the + 4h timeshift, the profile arising from experiment B is adjusted to account for the expected [P] under standard conditions such that all points are shifted vertically (+ 0.065 M to each point of experiment B) **(Graph S1 and S2)**.

The normalised same excess trace (green squares) shows a faster rate than the control (dark blue circles). This implies either catalyst deactivation or product inhibition occurs in the reaction. Comparison of the same excess trace with additional product, $KHCO_3$ and KF (light blue circles) shows a better overlap with the control profile than the same excess profile, indicating that product inhibition is occurring in the reaction. Additionally, another same excess experiment in which only the sulfone product was added (dark red circles) showed excellent overlap with the control profile, indicating that product inhibition arises from the sulfone formed and not one of the other potential by-products **(Graph S2)**.

Different Excess

Visual comparisons were used to estimate the reaction order in each component using different excess experiments **(Table S15)** according to variable time normalisation analysis (VTNA) methods developed by Burés.[5]

**Experiment Profile**	[CHO] (M)	[Sulfinate] (M)	[TDG] (M)	[CuF_2] (M)	[$Cu(OAc)_2$] (M)	[K_2CO_3] (M)
A	0.473	0.189	0.0472	0.378	0.0945	0.378
B	0.567	0.189	0.0472	0.378	0.0945	0.378
B2	0.374	0.189	0.0472	0.378	0.0945	0.378
C	0.473	0.227	0.0472	0.378	0.0945	0.378
C2	0.473	0.151	0.0472	0.378	0.0945	0.378
D	0.473	0.189	0.0567	0.378	0.0945	0.378
D2	0.473	0.189	0.0756	0.378	0.0945	0.378
D3	0.473	0.189	0.0189	0.378	0.0945	0.378
E	0.473	0.189	0.0472	0.473	0.0945	0.378
E2	0.473	0.189	0.0472	0.284	0.0945	0.378
F	0.473	0.189	0.0472	0.378	0.142	0.378
G	0.473	0.189	0.0472	0.378	0.0945	0.473
G2	0.473	0.189	0.0472	0.378	0.0945	0.284

Table S15 – Different Excess experiments investigating 6 different components.

Order in Aldehyde (Expt A, B and B2)

a)

Time (h)	[P] for CHO loading (M)		
	3 equiv	2.5 equiv	2 equiv
0.00	0.000	0.000	0.000
2.00	0.051	0.039	0.034
4.00	0.084	0.062	0.057
6.00	0.105	0.085	0.075
8.00	0.122	0.102	0.092
10.00	0.131	0.113	0.103
12.00	0.129	-	0.113
12.21	-	0.122	-
15.00	0.133	0.136	0.120
18.00	0.142	0.146	0.124
22.00	-	0.159	-
23.50	0.153	-	-
24.00	-	0.161	0.1

b)

Time (h)	$\Sigma[CHO]^x\Delta t$								
	Order x = 0.0			Order x = 1.0			rder x = 2		
	3 equiv	2.5 equiv	2 equiv	3 equiv	2.5 equiv	2 equiv	3 equiv	2.5 equiv	2 equiv
0.00	0.00	0.00	0.00	0.00	0.00	0.00	0.00	0.00	0.00
2.00	2.00	2.00	2.00	1.07	0.91	0.73	0.57	0.41	0.27
4.00	4.00	4.00	4.00	2.06	1.75	1.40	1.06	0.77	0.49
6.00	6.00	6.00	6.00	2.99	2.55	2.04	1.50	1.08	0.69
8.00	8.00	8.00	8.00	3.89	3.30	2.63	1.90	1.37	0.87
10.00	10.00	10.00	10.00	4.76	4.03	3.20	2.28	1.64	1.03
12.00	12.00	-	12.00	5.61	-	3.75	2.65	-	1.18
12.21	-	12.21	-	-	4.82	-	-	1.92	-
15.00	15.00	15.00	15.00	6.91	5.78	4.55	3.20	2.24	1.40
18.00	18.00	18.00	18.00	8.18	6.77	5.33	3.74	2.57	1.60
22.00	-	22.00	-	-	8.05	-	-	2.98	-
23.50	23.50	-	-	10.45	-	-	4.68	-	-
24.00	-	24.00	24.00	-	8.68	6.80	-	3.18	1.96

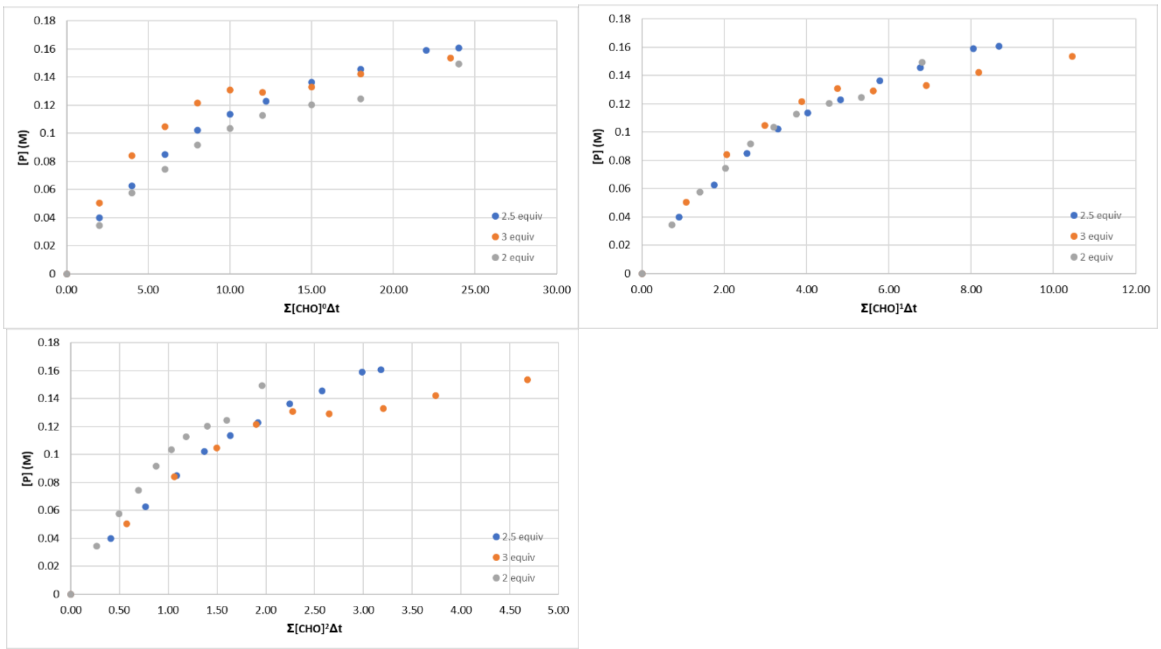

Figure S5 – a) Data for different CHO loadings. b) Normalised time data for different CHO loadings according to order x. Time normalised plots for CHO orders of c) x=0, d) x=1 and e) x=2.

Order in Sulfinate Salt (Expt A, C and C2)

a)	[P] for RSO_2Na loading (M)		
Time (h)	1.2 equiv	1 equiv	0.8 equiv
0.00	0.000	0	0.000
2.00	0.041	0.039	0.032
4.00	0.068	0.062	0.051
6.00	0.091	0.085	0.073
8.00	-	0.102	0.085
8.17	0.109	-	-
10.00	0.125	0.113	0.102
12.00	-	-	0.104
12.21	-	0.122	-
12.50	0.136	-	-
15.00	0.159	0.136	0.119
18.00	0.163	0.146	0.121
22.00	-	0.159	-
24.00	0.166	0.161	-

b)	$\Sigma[RSO_2Na]^x\Delta t$								
	x = 0.0			x = 0.75			x = 1.0		
Time (h)	1.2 equiv	1 equiv	0.8 equiv	1.2 equiv	1 equiv	0.8 equiv	1.2 equiv	1 equiv	0.8 equiv
0.00	0.00	0.00	0.00	0.00	0.00	0.00	0.00	0.00	0.00
2.00	2.00	2.00	2.00	0.61	0.53	0.45	0.41	0.34	0.27
4.00	4.00	4.00	4.00	1.15	0.98	0.83	0.76	0.61	0.49
6.00	6.00	6.00	6.00	1.62	1.38	1.15	1.05	0.85	0.67
8.00	-	8.00	8.00	-	1.72	1.43	-	1.04	0.81
8.17	8.17	-	-	2.08	-	-	1.33	-	-
10.00	10.00	10.00	10.00	2.44	2.02	1.65	1.53	1.20	0.92
12.00	-	-	12.00	-	-	1.86	-	-	1.01
12.21	-	12.21	-	-	2.33	-	-	1.36	-
12.50	12.50	-	-	2.87	-	-	1.77	-	-
15.00	15.00	15.00	12.00	3.24	2.66	2.12	1.97	1.52	1.13
18.00	18.00	18.00	15.00	3.63	2.97	2.35	2.17	1.67	1.23
22.00	-	22.00	-	-	3.31	-	-	1.81	-
24.00	24.00	24.00	-	4.38	3.45	-	2.54	1.87	-

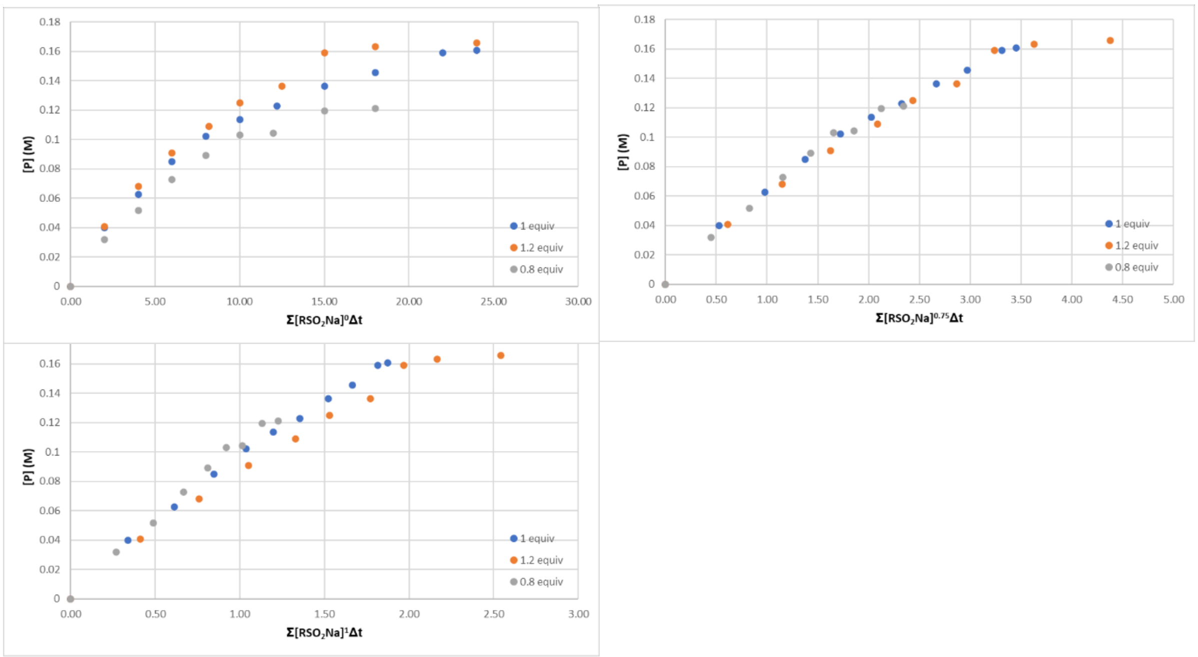

Figure S6 – a) Data for different RSO_2Na loadings. b) Normalised time data for different RSO_2Na loadings according to order x. Time normalised plots for RSO_2Na orders of c) x=0, d) x=0.75 and e) x=1.

Order in Transient Directing Group (Expt A, D, D2 and D3)

a)

Time (h)	[P] for TDG loading (M) 40 mol%	30 mol%	25 mol%	10 mol%
0.00	0.000	0.000	0.000	0.000
2.00	0.064	0.038	0.040	0.011
4.00	0.093	0.059	0.062	0.025
6.00	0.117	0.083	0.085	0.043
8.00	0.132	0.100	0.102	0.055
10.00	0.144	0.123	0.113	0.076
12.00	0.140	0.138	-	0.076
12.21	-	-	0.129	-
15.00	0.146	0.163	0.136	0.083
18.00	0.140	0.166	0.146	0.091
22.00	-	-	0.159	-
23.00	-	0.159	-	-
24.00	-	-	0.161	0.098

b)

	$\Delta t[TDG]^x$											
	X = 0.0				X = 1.0				X = 2.0			
Time (h)	40 mol%	30 mol%	25 mol%	10 mol%	40 mol%	30 mol%	25 mol%	10 mol%	40 mol%	30 mol %	25 mol%	10 mol%
0.00	0.00	0.00	0.00	0.00	0.00	0.00	0.00	0.00	0.00	0.00	0.00	0.00
2.00	2.00	2.00	2.00	2.00	0.15	0.11	0.09	0.04	0.01	0.01	0.00	0.00
4.00	4.00	4.00	4.00	4.00	0.30	0.23	0.19	0.08	0.02	0.01	0.01	0.00
6.00	6.00	6.00	6.00	6.00	0.45	0.34	0.28	0.11	0.03	0.02	0.01	0.00
8.00	8.00	8.00	8.00	8.00	0.61	0.45	0.38	0.15	0.05	0.03	0.02	0.00
10.00	10.00	10.00	10.00	10.00	0.76	0.57	0.47	0.19	0.06	0.03	0.02	0.00
12.00	12.00	12.00	-	12.00	0.91	0.68	-	0.23	0.07	0.04	-	0.00
12.21	-	-	12.21	-	-	-	0.58	-	-	-	0.03	-
15.00	15.00	15.00	15.00	15.00	1.13	0.85	0.71	0.28	0.09	0.05	0.03	0.01
18.00	18.00	18.00	18.00	18.00	1.36	1.02	0.85	0.34	0.10	0.06	0.04	0.01
22.00	-	-	22.00	-	-	-	1.04	-	-	-	0.05	-
23.00	-	23.00	-	-	-	1.30	-	-	-	0.07	-	-
24.00	-	-	24.00	24.00	-	-	1.13	0.45	-	-	0.05	0.01

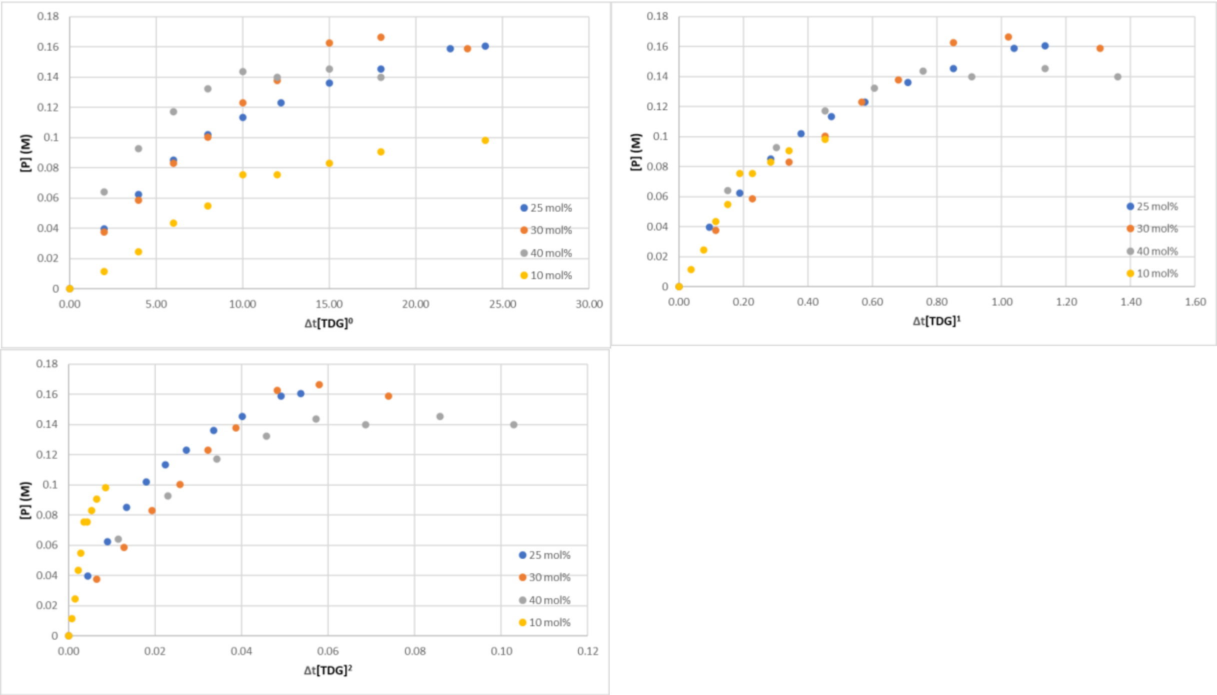

Figure S7 – a) Data for different TDG loadings. b) Normalised time data for different TDG loadings according to order x. Time normalised plots for TDG orders of c) x=0, d) x=1 and e) x=2

Order in CuF_2 (Expt A, E and E2)

a) Time (h)	[P] for CuF_2 loading (M) 2.5 equiv	2 equiv	1.5 equiv
0.00	0.000	0.000	0.000
2.00	0.055	0.040	0.034
4.00	0.081	0.062	0.064
6.00	0.104	0.085	0.087
8.00	0.115	0.102	0.098
10.00	0.121	0.113	0.106
12.00	0.130	-	0.115
12.21	-	0.123	-
15.00	0.142	0.136	0.129
18.00	0.157	0.146	0.129
22.00	-	0.159	-
24.00	0.164	0.160	0.140

b)	$\Sigma[CuF_2]^x\Delta t$								
	x = 0.0			x = 0.5			x = 1.0		
Time (h)	2.5 equiv	2 equiv	1.5 equiv	2.5 equiv	2 equiv	1.5 equiv	2.5 equiv	2 equiv	1.5 equiv
0.00	0.00	0.00	0.00	0.00	0.00	0.00	0.00	0.00	0.00
2.00	2.00	2.00	2.00	1.29	1.16	1.00	0.84	0.68	0.50
4.00	4.00	4.00	4.00	2.45	2.21	1.86	1.51	1.23	0.87
6.00	6.00	6.00	6.00	3.53	3.17	2.59	2.08	1.69	1.13
8.00	8.00	8.00	8.00	4.53	4.05	3.21	2.59	2.07	1.33
10.00	10.00	10.00	10.00	5.50	4.86	3.78	3.06	2.40	1.49
12.00	12.00	-	12.00	6.44	-	4.28	3.51	-	1.61
12.21	-	12.21	-	-	5.69	-	-	2.71	-
15.00	15.00	15.00	15.00	7.79	6.65	4.88	4.11	3.04	1.73
18.00	18.00	18.00	18.00	9.04	7.58	5.36	4.63	3.33	1.81
22.00	-	22.00	-	-	8.67	-	-	3.63	-
24.00	24.00	24.00	24.00	11.37	9.15	6.10	5.54	3.74	1.90

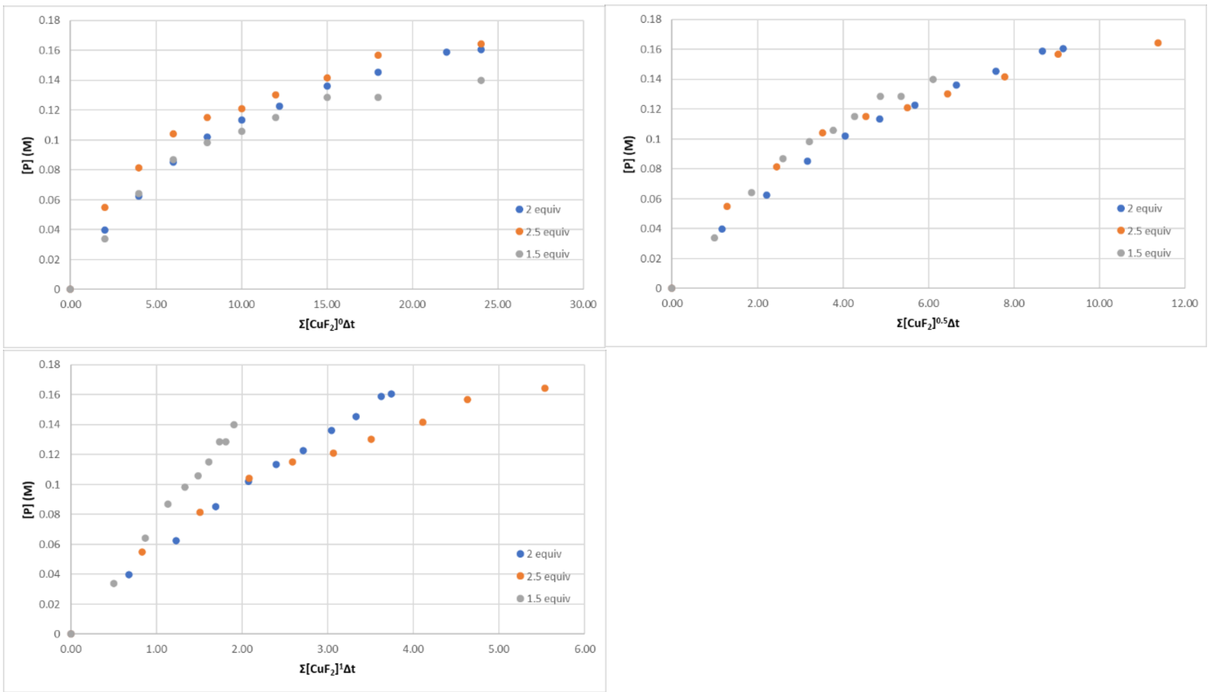

Figure S8 – a) Data for different CuF_2 loadings. b) Normalised time data for different CuF_2 loadings according to order x. Time normalised plots for CuF_2 orders of c) x=0, d) x=0.5 and e) x=1.

Order in $Cu(OAc)_2$

a)	[P] for $Cu(OAc)_2$ loading (M)	
Time (h)	75 mol%	50 mol%
0.00	0.000	0
2.00	0.032	0.040
4.00	0.062	0.062
6.00	0.085	0.085
8.00	0.093	0.102
10.00	0.117	0.113
12.21	-	0.123
12.33	0.127	-
15.00	0.144	0.136
18.00	0.149	0.146
22.00	-	0.159
24.00	0.163	0.161

b)	$\Delta t[Cu(OAc)_2]^x$			
	x = 0.0		x = 1.0	
Time (h)	75 mol%	50 mol%	75 mol%	50 mol%
0.00	0.00	0.00	0.00	0.00
2.00	2.00	2.00	0.28	0.19
4.00	4.00	4.00	0.57	0.38
6.00	6.00	6.00	0.85	0.57
8.00	8.00	8.00	1.13	0.76
10.00	10.00	10.00	1.42	0.95
12.21	-	12.21	-	1.15
12.33	12.33	-	1.75	-
15.00	15.00	15.00	2.13	1.42
18.00	18.00	18.00	2.55	1.70
22.00	-	22.00	-	2.08
24.00	24.00	24.00	3.40	2.27

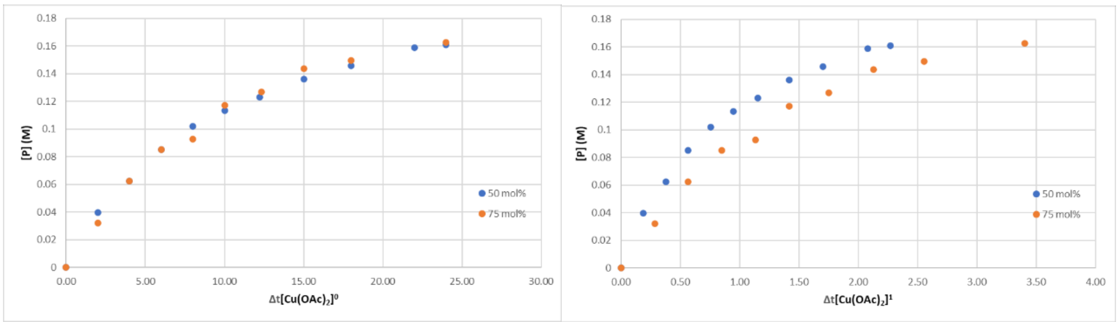

Figure S9 – a) Data for different $Cu(OAc)_2$ loadings. b) Normalised time data for different $Cu(OAc)_2$ loadings according to order x. Time normalised plots for CuF_2 orders of c) x=0, d) x=1.

Order in K_2CO_3

a)	[P] for K_2CO_3 loading (M)		
Time (h)	2.5 equiv	2 equiv	1.5 equiv
0.00	0.000	0.000	0.000
2.00	0.047	0.040	0.021
4.00	0.070	0.062	0.047
6.00	0.096	0.085	0.064
8.00	0.121	0.102	0.079
10.00	0.132	0.113	0.098
12.00	0.149	-	0.113
12.21	-	0.123	-
15.00	0.159	0.136	0.122
18.00	0.166	0.146	0.132
22.00	-	0.159	-
24.00	0.174	0.160	0.146

b)	$\Sigma[K_2CO_3]^x\Delta t$								
	x = 0.0			x = 1.0			x = 2.0		
Time (h)	2.5 equiv	2 equiv	1.5 equiv	2.5 equiv	2 equiv	1.5 equiv	2.5 equiv	2 equiv	1.5 equiv
0.00	0.00	0.00	0.00	0.00	0.00	0.00	0.00	0.00	0.00
2.00	2.00	2.00	2.00	0.90	0.72	0.55	0.40	0.26	0.15
4.00	4.00	4.00	4.00	1.73	1.37	1.05	0.75	0.47	0.27
6.00	6.00	6.00	6.00	2.51	1.98	1.50	1.05	0.66	0.38
8.00	8.00	8.00	8.00	3.23	2.55	1.92	1.31	0.82	0.47
10.00	10.00	10.00	10.00	3.93	3.09	2.31	1.55	0.96	0.54
12.00	12.00	-	12.00	4.59	-	2.67	1.77	-	0.61
12.21	-	12.21	-	-	3.66	-	-	1.11	-
15.00	15.00	15.00	15.00	5.54	4.36	3.17	2.08	1.29	0.69
18.00	18.00	18.00	18.00	6.47	5.07	3.63	2.37	1.45	0.76
22.00	-	22.00	-	-	5.97	-	-	1.66	-
24.00	24.00	24.00	24.00	8.29	6.41	4.50	2.92	1.75	0.89

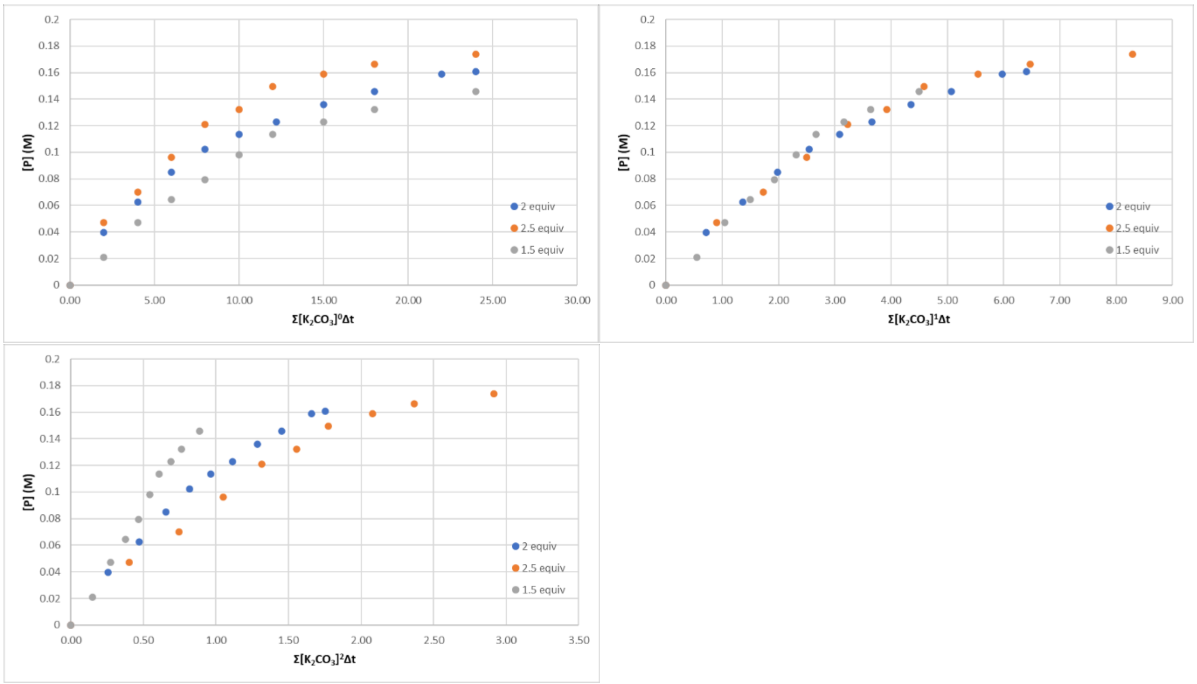

Figure S10 – a) Data for different K_2CO_3 loadings. b) Normalised time data for different K_2CO_3 loadings according to order x. Time normalised plots for K_2CO_3 orders of c) x=0, d) x=1 and e) x=2.

Imine formation in proteo HFIP

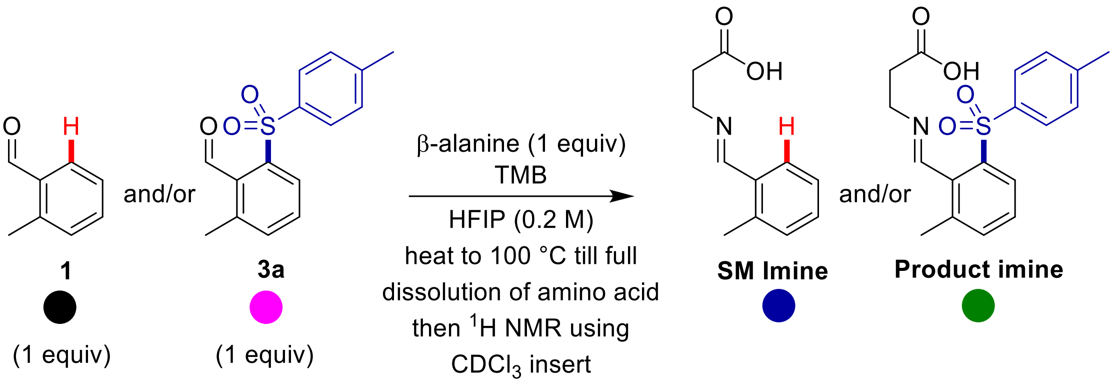

2-Methylbenzaldehyde **1** (23 µL, 0.2 mmol) and/or sulfonylated aldehyde **3a** (54.9 mg, 0.2 mmol), β-alanine (17.8 mg, 0.2 mmol) and accurately weighed 1,3,5-trimethoxybenzene (≈ 7 mg, 0.3 equiv) were heated to 100 °C in HFIP until dissolution of the amine. Each solution was added to separate NMR tubes with glass inserts containing $CDCl_3$. We observed an almost 1:1 ratio of SM(CHO):SM(imine) **(Table S16, Entry 1)**, whereas in we see only a small proportion of the product imine formed **(Table S16, Entry 2 or 3)**. This indicates product inhibition is unlike to result from **only** imine formation and instead could be due to the product imine being a better ligand for copper.

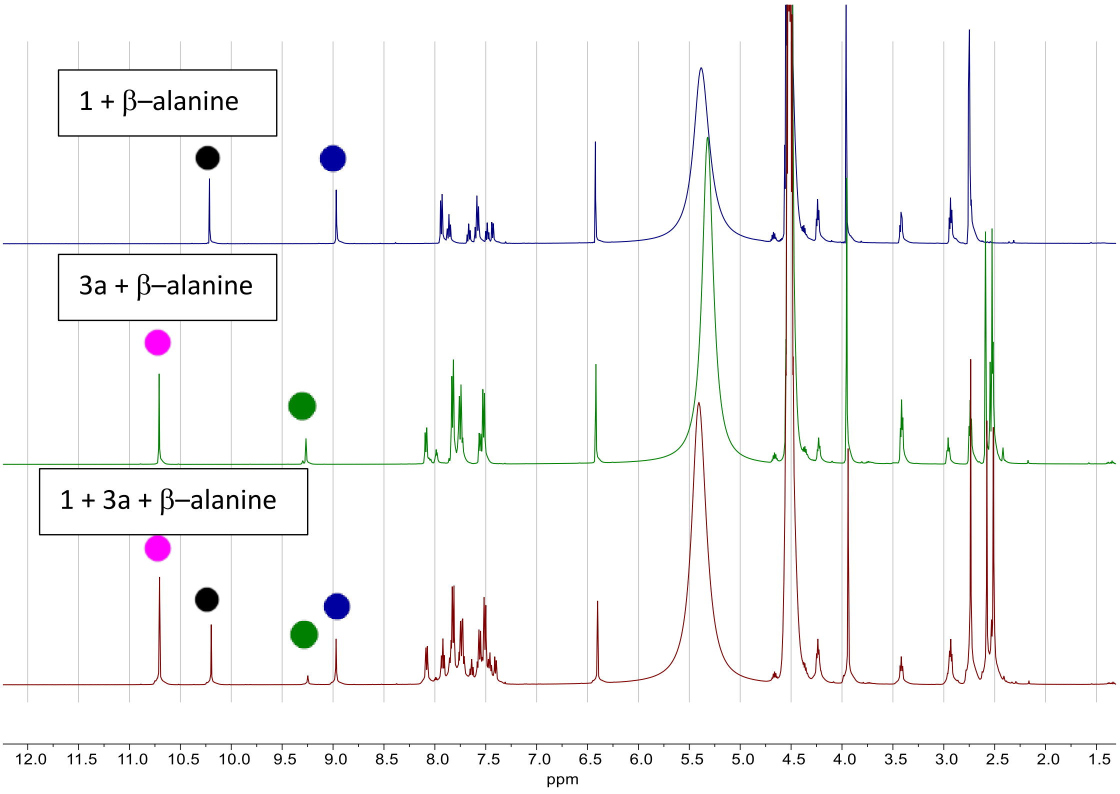

Entry	Description	SM Imine yield (%)	Product Imine yield (%)	SM CHO Yield (%)	Product CHO Yield (%)
1	Only SM aldehyde	54	-	40	-
2	Only product aldehyde	-	24	-	66
3	SM aldehyde + product aldehyde	41	7	42	79

Table S16 – Imine formation NMR experiments. Yields determined *in situ* by ^{1}H NMR by comparison with 1,3,5-trimethoxybenzene as an internal standard.

Deuteration Labelling Experiments

Reaction in HFIP-d$_2$

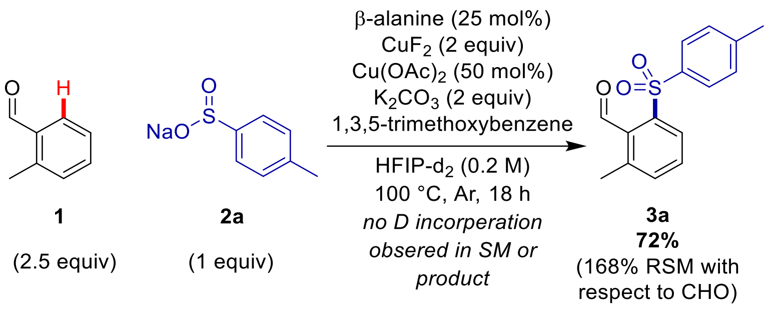

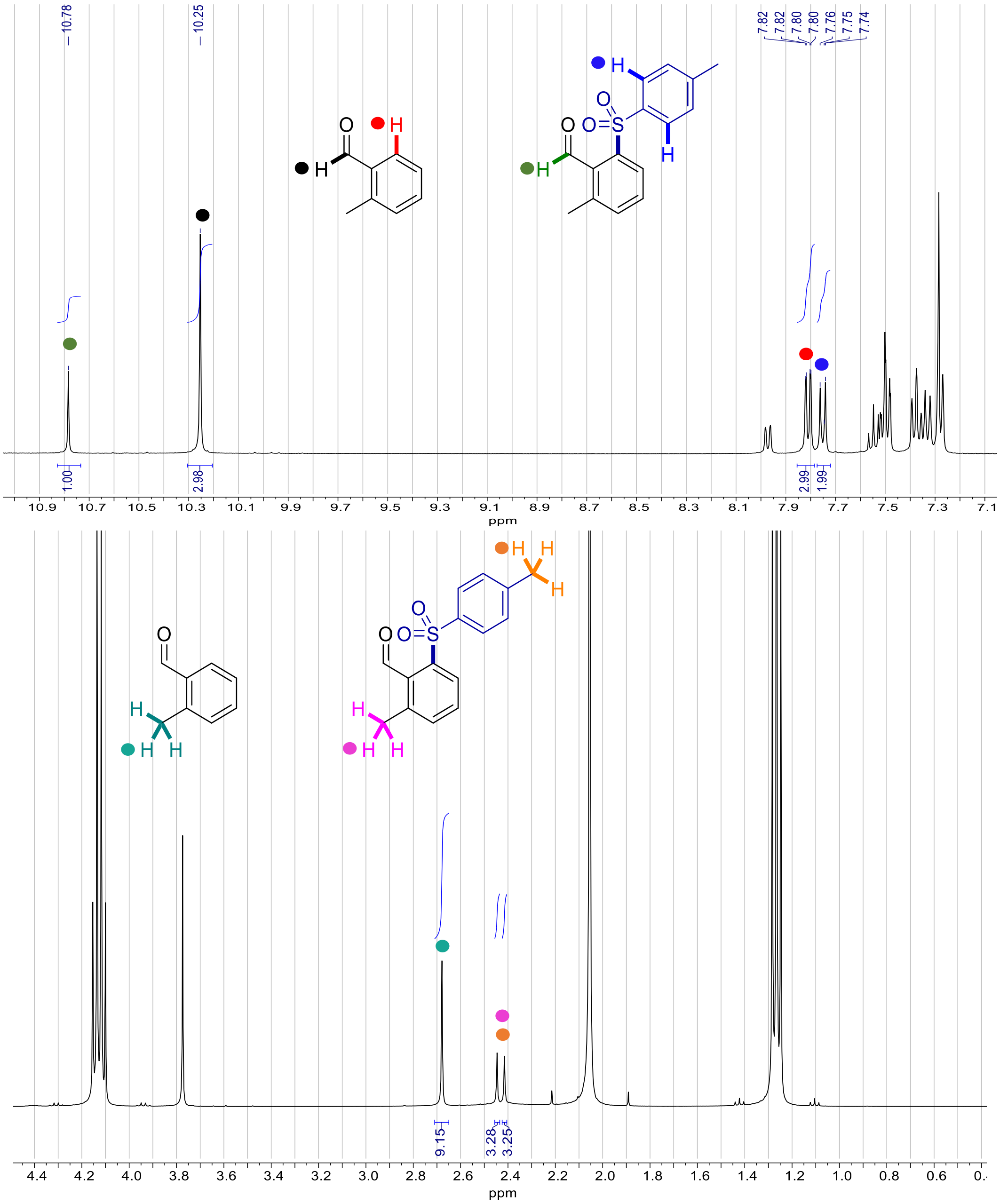

Scheme S8 – Deuteration experiment using HFIP-d$_2$ with regions of relevant protons shown. ^{1}H NMR was acquired with 30 s delay

KIE Experiments

Both parallel and competition experiments were carried out using benzaldehyde-d_5 as a substrate **(Scheme S9 and Scheme S10)**.

A procedure used for parallel KIE experiments comparing initial rates is outlined below.

Potassium carbonate (110 mg, 0.8 mmol) and copper(II) fluoride (81 mg, 0.8 mmol) were added sequentially to a 5 mL microwave vial which was flame dried under argon until a blue colour just appeared (ca. 2–5 seconds). The microwave vial was allowed to cool to room temperature and copper(II) acetate (36 mg, 0.2 mmol), β-alanine (8.9 mg, 0.1 mmol), *p*-tolylsulfinic acid sodium salt (71.2 mg, 0.4 mmol), accurately weighed 1,3,5-trimethoxybenzene (0.3 equiv) and the aldehyde were added to a microwave vial sequentially under argon. The vial was sealed and HFIP (0.2 M) was added and the vial was submerged in an oil bath preheated to 100 °C [Stirring rate set to 1000 rpm]. To take an aliquot: at the allotted time the vial was removed from the oil bath and 0.1 mL of solvent was removed by syringe while the reaction mixture is hot. [Caution: As the reaction is above the boiling point of the solvent, there is a small degree of back pressure. This was easily managed by holding the syringe plunger down gently while collecting the sample, and ensuring the liquid was taken up carefully and adequate inert atmosphere was taken up with the sample.] The aliquot was added to a vial containing a mixture of saturated aqueous ammonium chloride and EtOAc (approx. 1:1). This vial was sealed and shaken until the aliquot was observed to change from brown to blue/green. The organic and aqueous layers were allowed to separate, and the organic layer was carefully removed by pipette then filtered through a small pad of Na_2SO_4 and concentrated *in vacuo*. The entire residue was dissolved in $CDCl_3$ (<1 mL) and the yield of sulfonylated material was determined by ^{1}H NMR by comparison with the internal standard.

Timepoints at 0.25, 0.5, 0.75, 1 and 1.5 h were collected from one reaction and 2, 2.5 and 3 h were collected from a second reaction. Concentrations were calculated from the total volumes of the parent liquids (aldehyde+HFIP): 2.1015 mL.

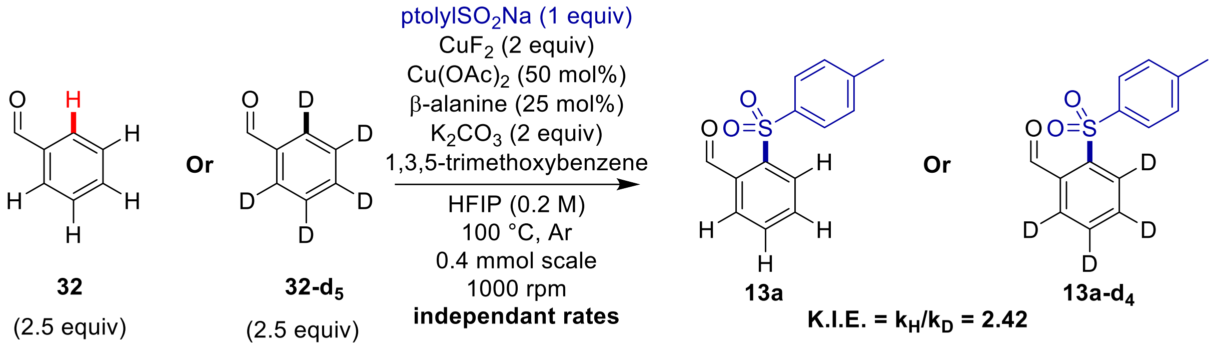

Proteo		
Time (h)	Yield (%)	[Sulfone] (mM)
0	0	0
0.25	2	3.81
0.5	3	5.71
0.75	4	7.61
1	6	11.42
1.5	8	15.23
2	10	19.03
2.5	11	20.94
3	13	24.74

Deutero		
Time (h)	Yield (%)	[Sulfone] (mM)
0	0	0
0.5	1	1.90
1	2	3.81
1.5	2	3.81
3	5	9.52
4.5	8	15.23

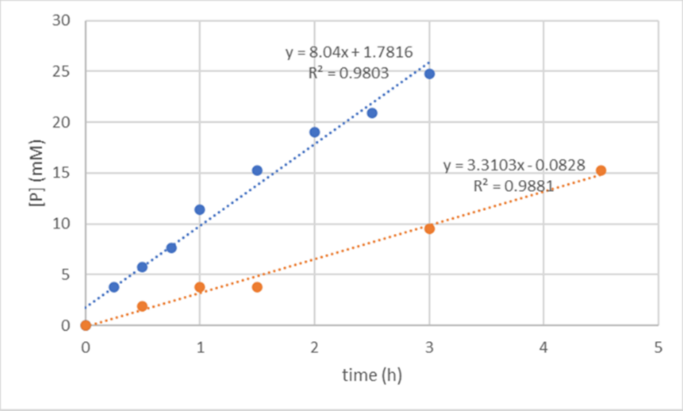

Scheme S9 – Parallel KIE experiment

Competition experiment

A procedure used for competition KIE experiments comparing initial rates is outlined below.

Potassium carbonate (27.5 mg, 0.2 mmol) and copper(II) fluoride (20 mg, 0.2 mmol) were added sequentially to a 5 mL microwave vial which was flame dried under argon until a blue colour just appeared (ca. 2–5 seconds). The microwave vial was allowed to cool to room temperature and copper(II) acetate (9 mg, 0.05 mmol), β-alanine (2.3 mg, 0.025 mmol), *p*-tolylsulfinic acid sodium salt (17.8 mg, 0.1 mmol), accurately weighed 1,3,5-trimethoxybenzene (4.5 mg, approx. 0.3 equiv) and benzaldehyde (25.4 μL, 0.25 mmol) were added to a microwave vial sequentially under argon. The vial was sealed and a solution of Benzaldehyde-d_5 in HFIP (25.4 μL in 0.5 mL HFIP) was added and the vial was submerged in an oil bath preheated to 100 °C for 3 h [stirring rate set to 500 rpm]. The reaction was allowed to cool to room temperature, diluted with EtOAc (5 mL) and the organic phase was washed with a saturated aqueous solution of ammonium chloride (5 mL), The product was extracted from the aqueous phase with EtOAc (2 × 5 mL) and the combined organic extracts were dried over Na_2SO_4, filtered, then concentrated *in vacuo* and analysed by ^{1}H NMR (with 30 s delay).

Due to volatility of starting material, accurate RSM values of proteo and deutero benzaldehydes were not possible to obtain, thus only product was considered. While it was not possible to directly analyse the quantity of the deutero benzaldehyde due to overlap of signals, it was possible to determine indirectly *via* comparison of the aldehyde signal at δ 10.86 ppm (which originates from both proteo and deutero product) and the signal at δ 8.03

ppm (which can only originate from the proteo product) Analysis by ^{1}H NMR (with 30 s delay) with comparison to an internal standard (1,3,5-trimethoxybenzene) allowed determination of the relative amounts of proteo and deutero products (13% and 4% respectively) which allowed a KIE of 3.25 to be determined. This is in agreement with the value derived from the parallel reactions, indicating a primary KIE and so a turnover limiting C–H activation.

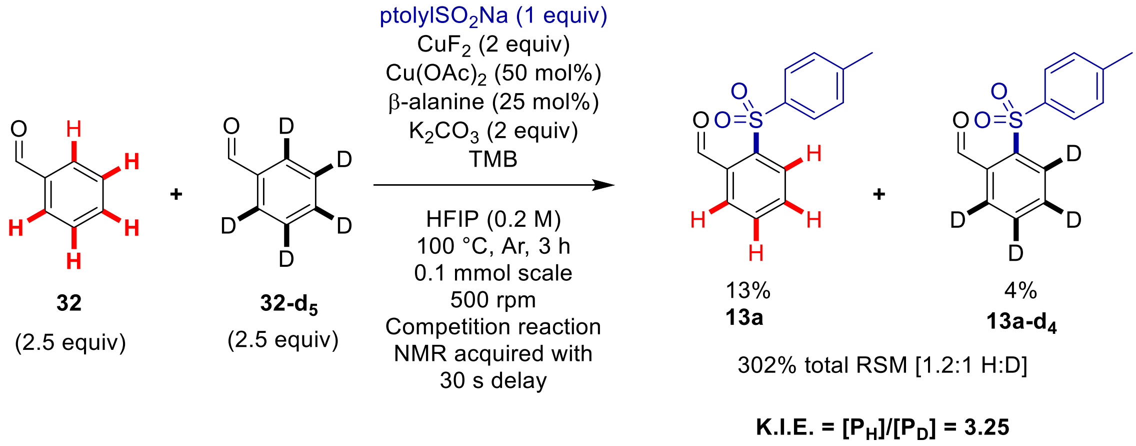

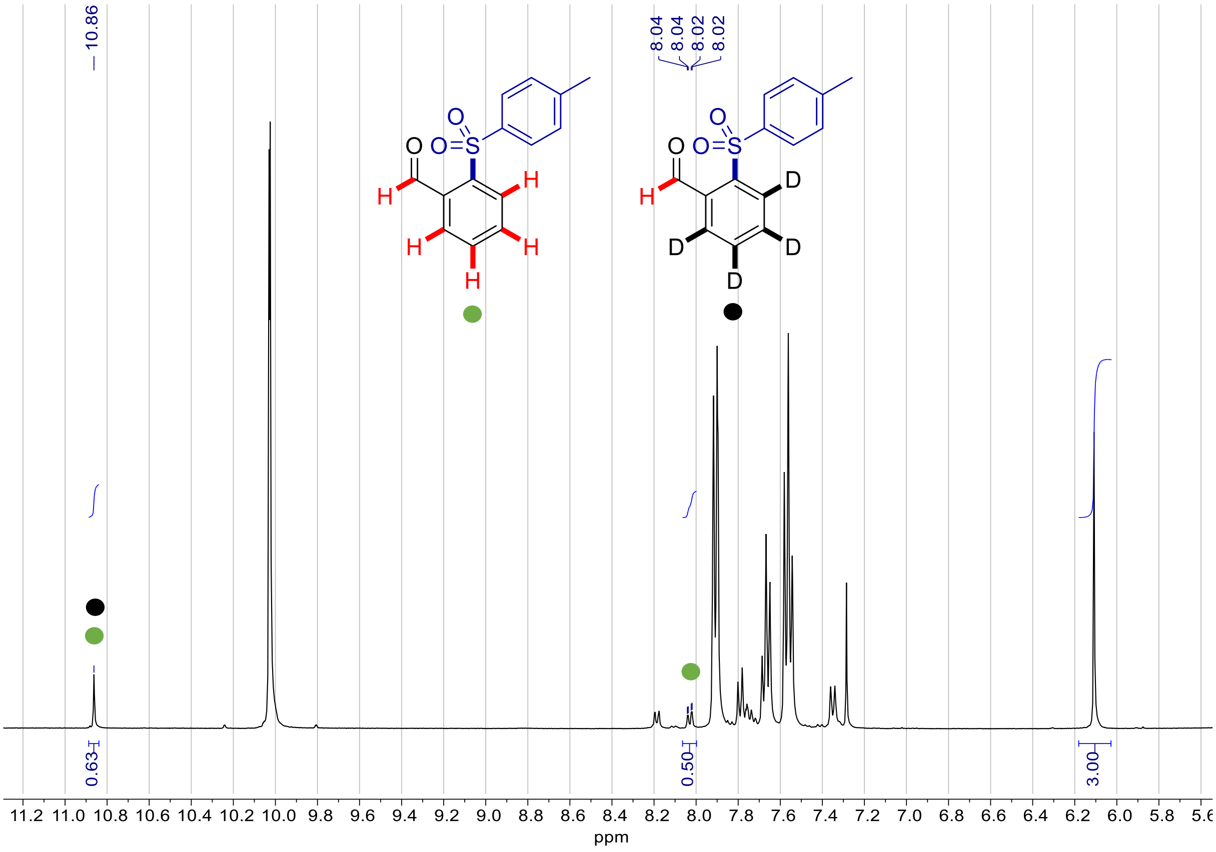

Scheme S10 – Competition K.I.E. experiment

Hammett Analysis

Standard procedure for Hammett analysis: Potassium carbonate (110 mg, 0.8 mmol) and copper(II) fluoride (81 mg, 0.8 mmol) were added sequentially to a 5 mL microwave vial which was flame dried under argon until a blue colour just appeared (ca. 2–5 seconds). The microwave vial was allowed to cool to room temperature and copper(II) acetate (36 mg, 0.2 mmol), β-alanine (8.9 mg, 0.1 mmol), *p*-tolylsulfinic acid sodium salt (71.2 mg, 0.4 mmol), accurately weighed 1,3,5-trimethoxybenzene (approx. 0.3 equiv) and the aldehyde (1 mmol) were added sequentially under argon. The vial was sealed and HFIP (0.2 M) was added, and the vial was submerged in an oil bath preheated to 100 °C [Stirring rate set to 1000 rpm]. To take an aliquot: at the allotted time the vial was removed from the oil bath and 0.2 mL of solvent was removed by syringe while the reaction mixture is hot. [Caution: As the reaction is above the boiling point of the solvent, there is a small degree of back pressure. This was easily managed by holding the syringe plunger down gently while collecting the sample, and ensuring the liquid is taken up carefully and adequate inert atmosphere was taken up with the sample.] The aliquot was added to a small vial containing a mixture of saturated aqueous ammonium chloride and EtOAc (approx. 1:1). The vial was sealed and shaken until the aliquot was observed to change from brown to blue/green. The organic layer was carefully removed by pipette then filtered through a small pad of Na_2SO_4 to remove any residual water, then the sample was concentrated *in vacuo*. The entire residue was dissolved in $CDCl_3$ (<1 mL) and the yield of sulfonylated material was determined by ^{1}H NMR by comparison with the internal standard. Concentrations were calculated from the total volumes of the parent liquids (aldehyde+HFIP): R = H (2.1015 mL), R = 2-Me (2.1156 mL), R = 2-OMe (2.1208 mL), R = 3-Me (2.1180 mL), R = 3-CF_3 (2.1340 mL), R = 3-Cl (2.1130 mL), 4-CF_3 (2.1370 mL), 4-Me (2.1180 mL), 4-OMe (2.1180 mL), 3-OMe (2.1220 mL).

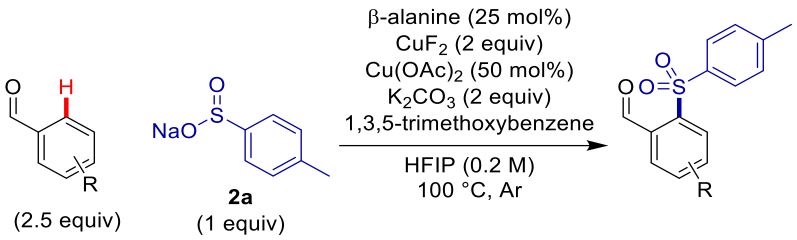

R = H		
Time (h)	Yield (%)	[Sulfone] (mM)
0	0	0
0.25	2	3.81
0.5	3	5.71
0.75	4	7.61
1	6	11.42
2	10	19.03
2.5	11	20.94
3	13	24.74

R = 2-Me		
Time (h)	Yield (%)	[Sulfone] (mM)
0	0	0
0.5	3	5.67
1	8	15.13
1.5	11	20.80
2	16	30.25

R = 2-OMe		
Time (h)	Yield (%)	[Sulfone] (mM)
0	0	0
0.5	4	7.54
1	6	11.32
1.5	9	16.97
2	12	22.63
3	15	28.29

R = 3-Me		
Time (h)	Yield (%)	[Sulfone] (mM)
0	0	0
0.5	4	7.55
1	8	15.11
1.5	11	20.77
2	15	28.33
3	22	41.55

R = 3-CF_3		
Time (h)	Yield (%)	[Sulfone] (mM)
0	0	0
0.5	1	1.87
1	2	3.75
1.5	3	5.62
2	4	7.50
3	6	11.25

R = 3-Cl		
Time (h)	Yield (%)	[Sulfone] (mM)
0	0	0
0.5	1	1.89
1	3	5.68
1.5	5	9.47
2	6	11.36
3	9	17.04

R = 4-CF_3		
Time (h)	Yield (%)	[Sulfone] (mM)
0	0	0
0.5	2	3.74
1	5	9.36
1.5	7	13.10
2	9	16.85
3	14	26.20

R = 4-Me		
Time (h)	Yield (%)	[Sulfone] (mM)
0	0	0
0.5	4	7.55
1	9	17.00
1.5	12	22.66
2	15	28.33

R = 4-OMe		
Time (h)	Yield (%)	[Sulfone] (mM)
0	0	0
0.5	4	7.54
1	7	13.20
1.5	11	20.74
2	15	28.28

R = 3-OMe					
Time (h)	Mono (%)	[mono] (mM)	Mono’ (%)	[Mono’] (mM)	[Total] (mM)
0	0	0	0	0	0
0.5	5	9.425071	3	5.655042	15.08011
1	9	16.96513	4	7.540057	24.50518
1.5	13	24.50518	5	9.425071	33.93025
2	17	32.04524	7	13.1951	45.24034

Table S17–Hammett analysis Data.

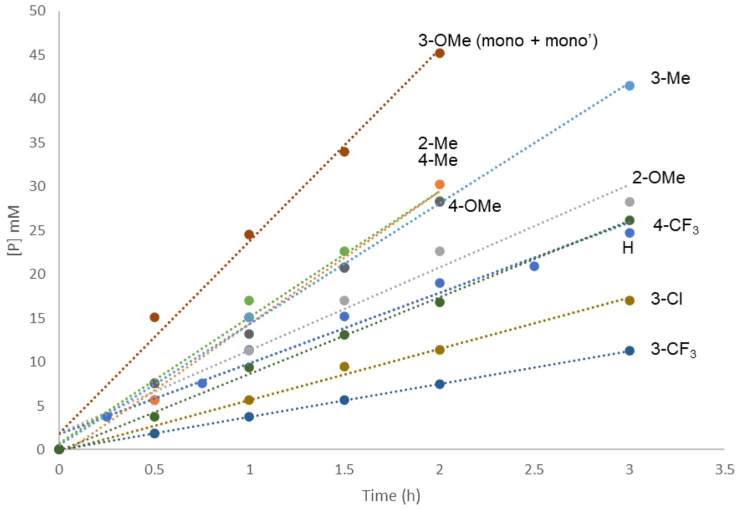

Graph S3 – Hammett analysis initial rates profiles

A negative correlation was observed when constructing the Hammett plot from the perspective of the C–H position highlighted. A poorer correlation was observed when instead using Hammett parameters from the ipso position. This indicates the change in electronics with varying substituents is more significant at the *ortho* C–H position rather than the ipso position, and so indicating a mechanism of C–H activation in which there is build up of positive charge which is stabilised by electron donating substituents.

Substituent	k_{obs} (mM/h)	Log(k_X/k_H)	Substituent	k_{obs} (mM/h)	Log(k_X/k_H)
H	8.04	0.00	3-Cl	5.84	-0.139
2-Me	15.13	0.274	3-CF_3	3.75	-0.331
2-OMe	9.43	0.069	4-Me	14.35	0.252
3-Me	13.76	0.233	4-OMe	13.95	0.239
3-OMe	21.87	0.435	4-CF_3	8.72	0.035

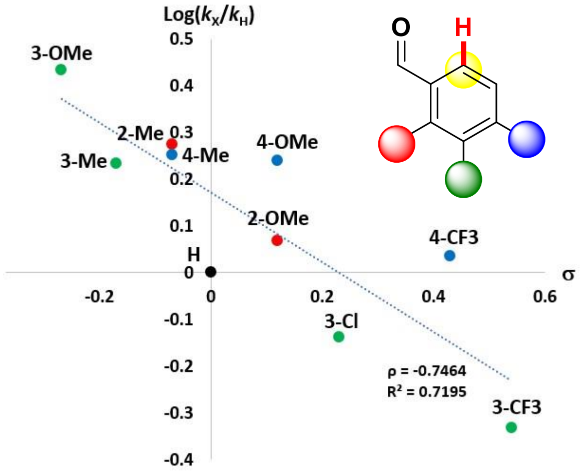

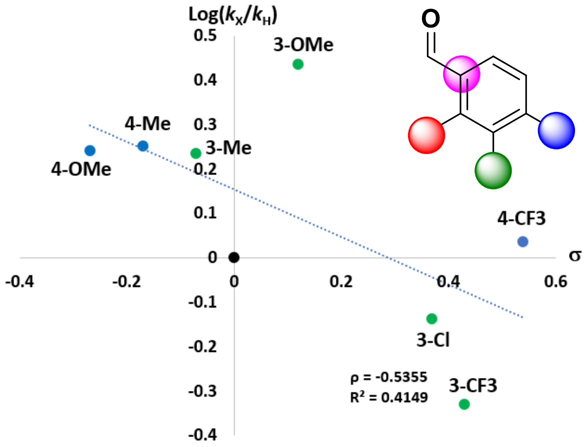

Figure S11 – a) Hammett analysis table. b) Hammett plot varying substitution on the aldehyde from the perspective of the C–H bond. c) Hammett plot varying substitution on the aldehyde from the perspective of the ipso position.

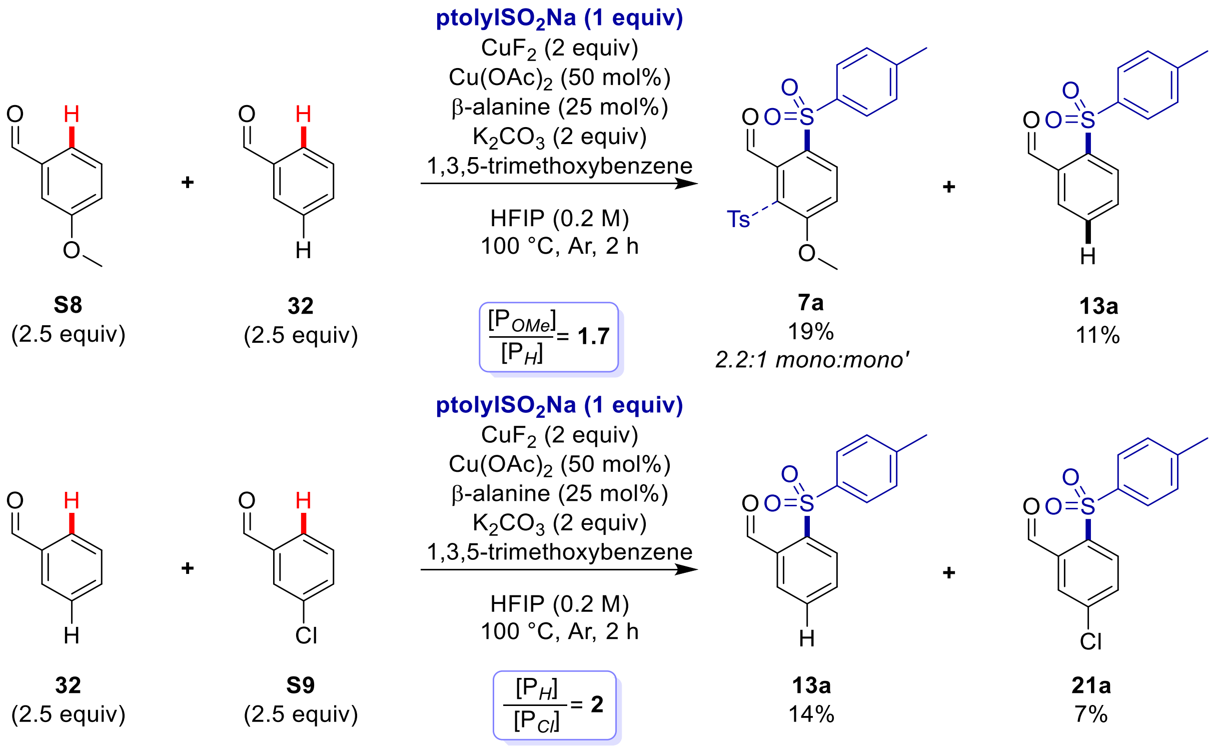

Scheme S11 – Competition experiments.

Synthesis of Sulfinate Salts

General Procedure A: Synthesis of Sulfinate Salts

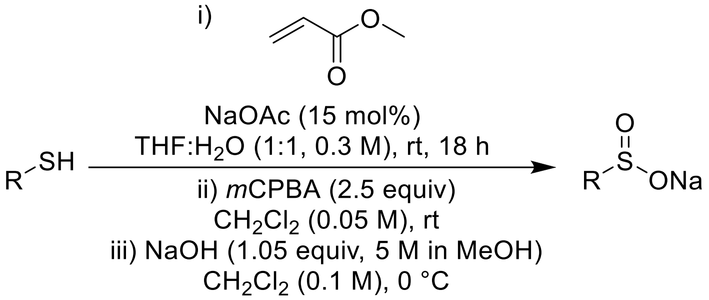

Thiol (1 equiv) was added to a stirring solution of methyl acrylate (1 equiv) in THF:H_2O (1:1, 0.3 M). Sodium acetate (0.15 equiv) was added, and the reaction stirred for 18 h. The reaction was concentrated *in vacuo*, then the product was extracted from the aqueous phase with ethyl acetate, dried over Na_2SO_4, filtered and concentrated *in vacuo.* The residue was dissolved in CH_2Cl_2 (0.05 M) and *m*CPBA (3 equiv) was added at rt. The reaction was stirred until completion, as determined by TLC. The reaction was quenched by the addition of 1 M $NaOH_{(aq)}$ then the product was extracted with CH_2Cl_2, dried over Na_2SO_4, filtered and concentrated *in vacuo* to afford the sulfone intermediate. The sulfone intermediate was then dissolved in CH_2Cl_2 and sodium hydroxide as a solution in MeOH (1.05 equiv, 5 M) was added to a stirring solution of sulfone in CH_2Cl_2 (0.17 M) at 0 °C. The precipitate was filtered off and washed with hexane to afford the sulfinate salt. Sulfinate salts were prepared as previously reported.[6]

Sodium bicyclo[1.1.1]pentane-1-sulfinate (S1)

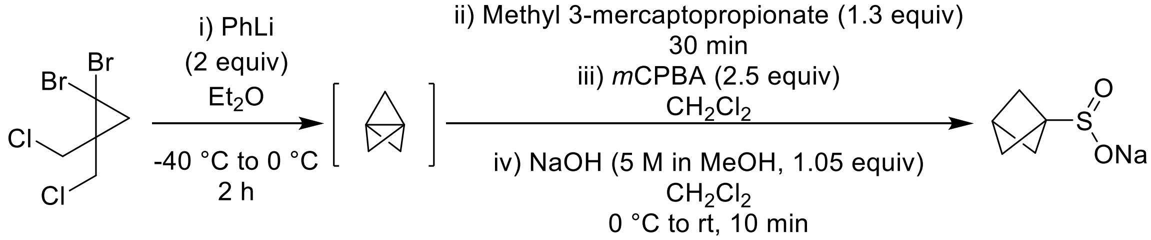

Using procedure modified from Bär *et al.*[7] PhLi (1.79 M in Bu_2O, 5.59 mL, 10 mmol) was added dropwise to a stirring solution of 1,1-dibromo-2,2-bis(chloromethyl)cyclopropane (1.484 g, 5 mmol) in Et_2O (7.2 mL) at -40 °C. After addition, the reaction was warmed to 0 °C then stirred for 2 h at 0 °C. Flame dried distillation apparatus purged with argon was attached to the reaction flask and the receiving flask was cooled to -78 °C. The reaction was placed in water bath and warmed to 20 °C and the intermediary propellane was co distilled with Et_2O under reduced pressure (slowly from 500 to 20 mbar). Methyl 3-mercaptopropionate (719 μL, 6.5 mmol) was added to the solution of propellane in Et_2O at rt and the reaction was stirred for 30 min at this temperature. The reaction mixture was washed with 1 M $NaOH_{(aq)}$ (10 mL), dried over Na_2SO_4, filtered and concentrated *in vacuo*. The crude sulfide was dissolved in CH_2Cl_2 (100 mL) and *m*CPBA (2.9 g, 12.5 mmol) was added at rt and the solution was stirred for 1 h. 1 M NaOH (100 mL) was added and the product extracted with CH_2Cl_2 (3 × 50 mL). the combined organic extracts were dried over Na_2SO_4, filtered, and concentrated *in vacuo*. The crude sulfone was dissolved in CH_2Cl_2 (45 mL) and sodium hydroxide as a solution in MeOH (948 μL, 5 M) was added. The precipitate was filtered off and washed with hexane to

afford sodium bicyclo[1.1.1]pentane-1-sulfinate **S1** as a white powder (355.3 mg, 46% over 4 steps). m.p. = >300 °C. IR (film)/cm^{-1} 2974, 2955, 2900, 2869, 1553, 1449, 1203, 1015, 990, 931, 897, 858, 661, 607, 582, 542, 475. ^{1}H NMR (400 MHz, D_2O) δ 2.67 (s, 1H, CH), 1.87 (s, 6H, $(CH_2)_3$). ^{13}C NMR (101 MHz, D_2O) δ 57.2 (C_q), 47.2 ($(CH_2)_3$), 25.8 (CH). Analytical data (^{1}H, ^{13}C NMR) are in agreement with the reported literature.[7]

Copper Mediated C(sp^2)–H Sulfonylation

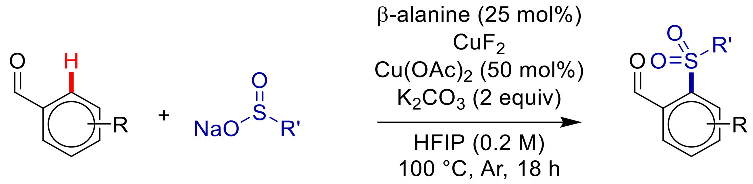

General Procedure B: Lab humidity<75%

Potassium carbonate (55 mg, 0.4 mmol) and copper(II) fluoride (40 mg, 0.4 mmol) were added sequentially to a microwave vial which was then flame dried under argon until a blue colour just appeared (ca. 2–5 seconds). The microwave vial was allowed to cool to room temperature and copper(II) acetate (18 mg, 0.1 mmol), β-alanine (4.5 mg, 0.05 mmol), sulfinic acid sodium salt (0.2 mmol) and the aldehyde (0.5 mmol) were added to a microwave vial sequentially under argon, sealed and HFIP (1 mL, 0.2 M) was added and the vial was submerged in a preheated oil bath to 100 °C for 18 h [Stirring rate set to 500 rpm]. The reaction was allowed to cool to room temperature, diluted with EtOAc (5 mL) and the organic phase was washed with a saturated aqueous solution of ammonium chloride (10 mL), [Note: The crude should be shaken until a change from orange/brown to blue/green is observed. Should the resulting solution emulsify brine can be added. Occasionally a brown precipitate can remain which obscures the phase boundary, this is collected with the aqueous phase for the first two extractions then with the organic phase on the final extraction]. The product was extracted from the aqueous phase with EtOAc (2 × 10 mL) and the combined organic extracts were dried over Na_2SO_4, filtered and concentrated *in vacuo*.

[Note: We found that the presence of water (from lab humidity) can negatively impact the yield and reproducibility, therefore if lab humidity is >75% following general procedure **C** can be used to mitigate this effect.]

General Procedure C: Lab humidity>75%

Potassium carbonate (55 mg, 0.4 mmol) and copper(II) fluoride (40 mg, 0.4 mmol) were added sequentially to a microwave vial which was then flame dried under argon until a blue colour just appeared (ca. 2–5 seconds). The microwave vial was allowed to cool to room temperature and copper(II) acetate (18 mg, 0.1 mmol), β-alanine (4.5 mg, 0.05 mmol), acid sodium salt (0.2 mmol) were added sequentially. The vial was sealed then purged and then backfilled with argon (3 times, purge for 30 s each time). To a separate flame dried vial under argon, aldehyde (0.5 mmol) was added then diluted with HFIP (1 mL, 0.2 M). The solution of aldehyde in HFIP was transferred to the reaction vial then submerged in a oil bath preheated to 100 °C for 18 h [Stirring rate set to 500 rpm]. The reaction was allowed to cool to room temperature, diluted with EtOAc (5 mL) and the organic phase was washed with a saturated aqueous solution of ammonium chloride (10 mL). [Note: The crude should be shaken until a change from orange/brown to blue/green is observed, should the resulting solution emulsify brine can be

added. Occasionally a brown precipitate can remain which obscures the phase boundary, this was collected with the aqueous phase for the first two extractions then with the organic phase on the final extraction] The product was extracted from the aqueous phase with EtOAc (2 × 10 mL) and the combined organic extracts were dried over Na_2SO_4, filtered and concentrated *in vacuo*.

General Note on Purification

Unless otherwise stated, sulfonylated aldehydes were purified by flash column chromatography using a short silica column (approx. 4 cm height in 2.5 cm diameter column). If the reaction gave 1 product: 10% Et_2O:pentane was used until the starting material came off the column, then 20% Et_2O:pentane was used until the product came off the column.
If the reaction gave >1 products: 10% Et_2O:pentane was used until the starting material came off the column, then 20% Et_2O:pentane was used until the first product came off. At this point the solvent system was changed to 20% EtOAc:pentane for the remaining products.

Reaction Scope Varying the Sulfinate Salt

2-(Phenylsulfonyl)-6-methylbenzaldehyde (3b)

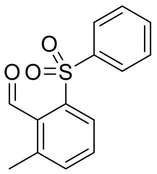

Synthesised according to general procedure **B** using 2-methylbenzaldehyde (58 μL, 0.5 mmol) and benzenesulfinic acid sodium salt (33.1 mg, 0.2 mmol). Purification by flash column chromatography (20% Et_2O:pentane) afforded sulfonyl aldehyde **3b** as a white solid (34.0 mg, 65%). m.p. = 105–107 °C. R_f 0.26 (20% Et_2O:pentane). IR (film)/cm^{-1} 3064, 2863, 2930, 2967, 1703 (C=O), 1588, 1446, 1387, 1312, 1163, 1141, 1085, 1025, 880, 790, 753, 723, 686. ^1H NMR (400 MHz, $CDCl_3$) δ 10.80 (s, 1H, CHO), 8.00 (d, *J* = 7.7 Hz, 1H, Ar–CH), 7.90–7.87 (m, 2H, 2 × Ar–CH), 7.63–7.49 (m, 5H, 5 × Ar–CH), 2.45 (s, 3H, CH_3). ^{13}C NMR (101 MHz, $CDCl_3$) δ 193.3 (CHO), 141.54 (Ar–C_q), 141.46 (Ar–C_q), 139.7 (Ar–C_q), 136.9 (Ar–CH), 134.7 (Ar–C_q), 133.6 (Ar–CH), 131.3 (Ar–CH), 129.4 (2 × Ar–CH), 127.5 (2 × Ar–CH), 127.4 (Ar–CH), 20.6 (CH_3). HRMS (TOF–ESI^+) m/z calcd. For $C_{14}H_{13}O_3S$ [M+H]: 261.0585; found: 261.0593.

2-((4-Methoxyphenyl)sulfonyl)-6-methylbenzaldehyde (3c)

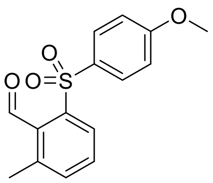

Synthesised according to general procedure **B** using 2-methylbenzaldehyde (58 μL, 0.5 mmol) and 4-methoxybenzenesulfinic acid sodium salt (39.0 mg, 0.2 mmol). Purification by flash column chromatography (20% Et_2O:pentane) afforded sulfonyl aldehyde **3c** as a white solid (40.1 mg, 69%). m.p. = 129–131 °C. R_f 0.06 (20% Et_2O:pentane). IR (film)/cm^{-1} 2930, 2952, 2848, 2728, 1703 (C=O), 1595, 1498, 1498, 1446, 1379, 1308, 1267, 1155, 1084, 857, 835, 787. ^1H NMR (400 MHz, $CDCl_3$) δ 10.83 (s, 1H, CHO), 7.95 (dd, *J* = 7.7, 0.7 Hz, 1H, Ar–CH), 7.83–7.79 (m, 2H, 2 × Ar–CH), 7.52 (dd, *J* = 7.7, 7.7 Hz, 1H, Ar–CH), 7.47 (dt, *J* = 7.7, 0.7 Hz, 1H, Ar–CH), 7.01–6.97 (m, 2H, 2 × Ar–CH), 3.86 (s, 3H, OCH_3), 2.45 (s, 3H, $ArCH_3$). ^{13}C NMR (101 MHz, $CDCl_3$) δ 193.7 (CHO), 163.7 (Ar–C_q), 142.4 (Ar–C_q), 139.5 (Ar–C_q), 136.5 (Ar–CH), 134.6 (Ar–C_q), 132.8 (Ar–C_q), 131.2 (Ar–CH), 129.9 (2 × Ar–CH), 127.0 (Ar–CH), 114.6 (2 × Ar–CH), 55.7 (OCH_3), 20.6 ($ArCH_3$). HRMS (TOF–ESI^+) m/z calcd. For $C_{15}H_{15}O_4S$ [M+H]: 291.0691; found: 291.0686.

2-((4-*tert*-Butylphenyl)sulfonyl)-6-methylbenzaldehyde (3d)

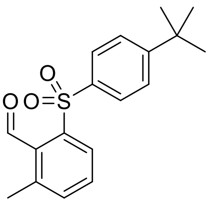

Synthesised according to general procedure **B** using 2-methylbenzaldehyde (58 μL, 0.5 mmol) and 4-(*tert*butyl)benzenesulfinic acid sodium salt (44.1 mg, 0.2 mmol). Purification by flash column chromatography (10%–20% Et_2O:pentane) afforded sulfonyl aldehyde **3d** as a white solid (36.8 mg, 58%). m.p. = 126–127 °C. R_f 0.33 (20% Et_2O:pentane). IR (film)/cm^{-1} 2963, 2870, 1703 (C=O), 1591, 1562, 1454, 1398, 1316, 1141, 1107, 1014, 913, 880, 839, 753, 675. ^1H NMR (400 MHz, $CDCl_3$) δ 10.82 (s, 1H, CHO), 8.00 (ddd, *J* = 7.7, 1.3, 0.6 Hz, 1H, Ar–CH), 7.82–7.78 (m, 2H, 2 × Ar–CH), 7.57–7.48 (m, 4H, 4 × Ar–CH), 2.46 (s, 3H, CH_3), 1.32 (s, 9H, $C(CH_3)_3$). ^{13}C NMR (101 MHz, $CDCl_3$) δ 193.4 (CHO), 157.6 (Ar–C_q), 142.0 (Ar–C_q), 139.6 (Ar–C_q), 138.4 (Ar–C_q), 136.8 (Ar–CH), 134.6 (Ar–C_q), 131.2 (Ar–CH), 127.4 (2 × Ar–CH), 127.3 (Ar–CH), 126.5 (2 × Ar–CH), 35.2 ($C_q(CH_3)_3$), 31.0 ($C(CH_3)_3$), 20.7 ($ArCH_3$). HRMS (TOF–ESI$^+$) m/z calcd. For $C_{18}H_{21}O_3S$ [M+H]: 317.1211; found: 317.1208.

2-((4-Trifluoromethylphenyl)sulfonyl)-6-methylbenzaldehyde (3e)

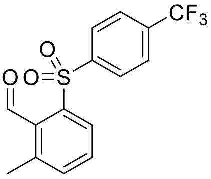

Synthesised according to general procedure **B** using 2-methylbenzaldehyde (58 μL, 0.5 mmol) and 4-trifluoromethylbenzenesulfinic acid sodium salt (46.4 mg, 0.2 mmol). Purification by flash column chromatography (10% EtOAc:pentane) afforded sulfonyl aldehyde **3e** as a white solid (41.5 mg, 63%). m.p. = 93–94 °C. R_f 0.16 (20% Et_2O:pentane). IR (film)/cm^{-1} 2920, 2854, 1704 (C=O), 1586, 1558, 1401, 1320, 1165, 1134, 1134, 1060, 1014, 878, 842, 787, 739, 617, 556, 423. ^1H NMR (400 MHz, $CDCl_3$) δ 10.77 (s, 1H, CHO), 8.04–8.00 (m, 3H, 3 × Ar–CH), 7.80 (d, *J* = 8.3 Hz, 2H, 2 × Ar–CH), 7.59 (dd, *J* = 7.7, 7.7 Hz, 1H, Ar–CH), 7.54 (d, *J* = 7.4 Hz, 1H, 1 × Ar–CH), 2.46 (s, 3H, CH_3). ^{13}C NMR (101 MHz, $CDCl_3$) δ 193.5 (CHO), 145.1 (Ar–C_q), 140.1 (Ar–C_q), 139.6 (Ar–C_q), 137.3 (Ar–CH), 135.7 (Ar–C_q), 135.2 (q, $^2J_{C\text{–}F}$ = 38.0 Hz, Ar–C_q), 131.4 (Ar–CH), 128.2 (2 × Ar–CH), 127.7 (Ar–CH), 126.5 (q, $^3J_{C\text{–}F}$ = 3.8 Hz, 2 × Ar–CH), 122.9 (q, $^1J_{C\text{–}F}$ = 274.2 Hz, C_qF_3), 20.3 (CH_3). $^{19}F\{^1H\}$ NMR (377 MHz, $CDCl_3$) δ –63.24. HRMS (TOF–ESI$^+$) m/z calcd. For $C_{15}H_{12}O_3SF_3$ [M+H]: 329.0459; found: 329.0466.

2-((4-Fluorophenyl)sulfonyl)-6-methylbenzaldehyde (3f)

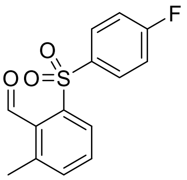

Synthesised according to general procedure **B** using 2-methylbenzaldehyde (58 μL, 0.5 mmol) and 4-fluorobenzenesulfinic acid sodium salt (34.6 mg, 0.2 mmol). Purification by flash column chromatography (10–20% Et_2O:pentane) afforded sulfonyl aldehyde **3f** as a white solid (40.0 mg, 72%). m.p. = 97–100 °C. R_f 0.17 (20% Et_2O:pentane). IR (film)/cm^{-1} 3105, 3075, 2982, 2930,2863, 1707 (C=O), 1592, 1491, 1454, 1405, 1320, 1293, 1238, 1137, 880, 839, 790, 746, 686, 593, 563, 500. ^1H NMR (400 MHz, $CDCl_3$) δ 10.80 (s, 1H, CHO), 7.98–7.96 (m, 1H, Ar–CH), 7.93–7.88 (m, 2H, 2 × Ar–CH), 7.56 (t, *J* = 7.7 Hz, 1H, Ar–CH), 7.54–7.50 (m, 1H, Ar–CH), 7.24–7.18 (m, 2H, 2 × Ar–CH), 2.45 (s, 3H, CH_3). ^{13}C NMR (101 MHz, $CDCl_3$) δ 193.7 (CHO), 165.6 (d, $^1J_{C\text{–}F}$ = 257.0 Hz, Ar–C_q), 141.2 (Ar–C_q), 139.4 (Ar–C_q), 137.5 (d, $^4J_{C\text{–}F}$ = 3.0 Hz, Ar–C_q), 136.9 (Ar–CH), 135.1 (Ar–C_q), 131.2 (Ar–CH), 130.6 (d, $^3J_{C\text{–}F}$ = 9.6 Hz, 2 × Ar–CH), 127.2 (Ar–CH), 116.8 (d, $^2J_{C\text{–}F}$ = 22.9 Hz, 2 × Ar–CH), 20.4 (CH_3). $^{19}F\{^1H\}$ NMR (377 MHz, $CDCl_3$) δ –103.16. HRMS (TOF–ESI$^+$) m/z calcd. For $C_{14}H_{12}O_3SF$ [M+H]: 279.0491; found: 279.0492.

2-((4-Chlorophenyl)sulfonyl)-6-methylbenzaldehyde (3g)

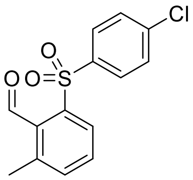

Synthesised according to general procedure **B** using 2-methylbenzaldehyde (58 μL, 0.5 mmol) and 4-chlorobenzenesulfinic acid sodium salt (39.7 mg, 0.2 mmol). Purification by flash column chromatography (20% Et_2O:pentane) afforded sulfonyl aldehyde **3g** as a white solid (43.7 mg, 74%). m.p. = 95–98 °C. R_f 0.19 (20% Et_2O:pentane). IR (film)/cm^{-1} 3090, 2960, 2862, 2930, 1707 (C=O), 1580, 1476, 1394, 1320, 1282, 1163, 1088, 1014, 880, 828, 794, 757, 708, 678. ^{1}H NMR (400 MHz, $CDCl_3$) δ 10.78 (s, 1H, CHO), 7.99–7.97 (m, 1H, Ar–CH), 7.83–7.80 (m, 2H, 2 × Ar–CH), 7.56 (t, *J* = 7.7 Hz, 1H, Ar–CH), 7.54–7.49 (m, 3H, 3 × Ar–CH), 2.45 (s, 3H). ^{13}C NMR (101 MHz, $CDCl_3$) δ 193.6 (CHO), 140.9 (Ar–C_q), 140.4 (Ar–C_q), 140.0 (Ar–C_q), 139.5 (Ar–C_q), 137.0 (Ar–CH), 135.2 (Ar–C_q), 131.3 (Ar–CH), 129.8 (2 × Ar–CH), 129.1 (2 × Ar–CH), 127.4 (Ar–CH), 20.4 (CH_3). HRMS (FTMS+pAPCI) m/z calcd. For $C_{14}H_{12}O_3S^{35}Cl$ [M+H]: 295.0190; found: 295.0183.

2-((4-Bromophenyl)sulfonyl)-6-methylbenzaldehyde (3h)

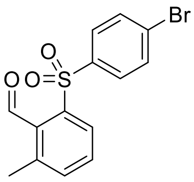

Synthesised according to general procedure **B** using 2-methylbenzaldehyde (58 μL, 0.5 mmol) and 4-bromobenzenesulfinic acid sodium salt (48.6 mg, 0.2 mmol). Purification by flash column chromatography (20% Et_2O:pentane) afforded sulfonyl aldehyde **3h** as a white solid (48.6 mg, 72%). m.p. = 97–98 °C. R_f 0.17 (20% Et_2O:pentane). IR (film)/cm^{-1} 3086, 2930, 2863, 1703 (C=O), 1573, 1472, 1386, 1320, 1163, 1164, 1070, 1010, 880, 824, 794, 742, 701, 675. ^{1}H NMR (400 MHz, $CDCl_3$) δ 10.77 (s, 1H, CHO), 7.98 (d, *J* = 7.7 Hz, 1H, Ar–CH), 7.74 (d, *J* = 8.9 Hz, 2H, 2 × Ar–CH), 7.67 (d, *J* = 8.9 Hz, 2H, 2 × Ar–CH), 7.56 (dd, *J* = 7.7, 7.7 Hz, 1H, Ar–CH), 7.51 (d, *J* = 7.7 Hz, 1H, Ar–CH), 2.45 (s, 3H, CH_3). ^{13}C NMR (101 MHz, $CDCl_3$) δ 193.5 (CHO), 140.8 (Ar–C_q), 140.5 (Ar–C_q), 139.5 (Ar–C_q), 137.0 (Ar–CH), 135.1 (Ar–C_q), 132.7 (2 × Ar–CH), 131.3 (Ar–CH), 129.1 (2 × Ar–CH), 129.0 (Ar–C_q), 127.4 (Ar–CH), 20.4 (CH_3).HRMS (TOF–ESI$^+$) m/z calcd. For $C_{14}H_{12}O_3S^{79}Br$ [M+H]: 338.9691; found: 338.9678.

2-((2-Methylphenyl)sulfonyl)-6-methylbenzaldehyde (3i)

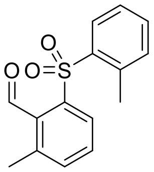

Synthesised according to general procedure **B** using 2-methylbenzaldehyde (58 μL, 0.5 mmol) and 2-methylbenzenesulfinic acid sodium salt (35.7 mg, 0.2 mmol). Purification by flash column chromatography (10–20% EtOAc:hexane) afforded sulfonyl aldehyde **3i** as a yellow oil (23.0 mg, 42%). R_f 0.20 (20% EtOAc:hexane). IR (film)/cm^{-1} 3059, 2921, 2854, 1701 (C=O), 1587, 1454, 1382, 1308, 1183, 1144, 1123, 1059, 1038, 878, 794, 762, 698, 609, 588, 565. ^{1}H NMR (400 MHz, $CDCl_3$) δ 10.73 (s, 1H, CHO), 8.12 (dd, *J* = 7.7, 1.8 Hz, 1H, Ar–CH), 7.98 (dd, *J* = 7.7, 1.8 Hz, 1H, Ar–CH), 7.59–7.51 (m, 3H, 3 × Ar–CH), 7.43 (ddd, *J* = 8.0, 8.0, 2.0 Hz, 1H, Ar–CH), *7.27–7.25 (m, 1H, Ar–CH), 2.50 (s, 3H, CH_3), 2.39 (s, 3H, CH_3). ^{13}C NMR (101 MHz, $CDCl_3$) δ 192.4 (CHO), 142.0 (Ar–C_q), 140.5 (Ar–C_q), 139.4 (Ar–C_q), 138.0 (Ar–C_q), 137.0 (Ar–CH), 134.1 (Ar–CH), 133.8 (Ar–C_q), 132.9 (Ar–CH), 131.1 (Ar–CH), 128.9 (Ar–CH), 127.4 (Ar–CH), 126.7 (Ar–CH), 21.0 (CH_3), 20.1 (CH_3). HRMS (TOF–ESI$^+$) m/z calcd. For $C_{15}H_{15}O_3S$ [M+H]: 275.0742; found: 275.0735. *signal overlaps with $CDCl_3$ signal, partly obscuring signal

2-(Naphthalen-1-ylsulfonyl)-6-methylbenzaldehyde (3j)

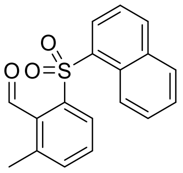

Synthesised according to general procedure **B** using 2-methylbenzaldehyde (58 μL, 0.5 mmol) and naphthalene-1-sulfinic acid sodium salt (42.4 mg, 0.2 mmol). Purification by flash column chromatography (10–20% EtOAc:hexane) afforded sulfonyl aldehyde **3j** as an white solid (30.7 mg, 50%). m.p. = 136–137 °C. R_f 0.19 (20% EtOAc:hexane). IR (film)/cm^{-1} 3059,

2947, 2919, 2851, 1700 (C=O), 1588, 1560, 1505, 1452, 1343, 1309, 1281, 1263, 1183, 1158, 1118, 974, 878, 826, 801, 770, 605, 587, 511, 454. ^{1}H NMR (400 MHz, $CDCl_3$) δ 10.86 (s, 1H, CHO), 8.46–8.41 (m, 2H, 2 × Ar–CH), 8.15 (dt, *J* = 8.3, 1.2 Hz, 1H, Ar–CH), 8.06 (d, *J* = 7.7 Hz, 1H, Ar–CH), 7.96–7.92 (m, 1H, Ar–CH), 7.65 (dd, *J* = 8.3, 7.5 Hz, 1H, Ar–CH), 7.59–7.53 (m, 3H, 3 × Ar–CH), 7.48 (d, *J* = 7.7 Hz, 1H, Ar–CH), 2.46 (s, 3H, CH_3). ^{13}C NMR (101 MHz, $CDCl_3$) δ 192.5 (CHO), 142.6 (Ar–C_q), 140.4 (Ar–C_q), 137.0 (Ar–CH), 136.1 (Ar–C_q), 135.7 (Ar–CH), 134.2 (Ar–C_q), 134.0 (Ar–C_q), 131.2 (Ar–CH), 129.9 (Ar–CH), 129.2 (Ar–CH), 128.6 (Ar–CH), 128.3 (Ar–C_q), 127.1 (Ar–CH), 126.9 (Ar–CH), 124.3 (Ar–CH), 124.0 (Ar–CH), 20.9 (CH_3). HRMS (TOF–ESI$^+$) m/z calcd. For $C_{18}H_{15}O_3S$ [M+H]: 311.0742; found: 311.0737.

2-Methyl-6-(methylsulfonyl)benzaldehyde (3k)

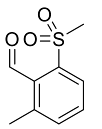

Synthesised according to general procedure **B** using 2-methylbenzaldehyde (58 μL, 0.5 mmol) and methylsulfinic acid sodium salt (20.1 mg, 0.20 mmol). Purification by flash column chromatography (30% Et_2O:pentane) afforded sulfonyl aldehyde **3k** as a white solid (30.3 mg, 77%). m.p. = 98–99 °C. R_f 0.05 (30% Et_2O:pentane). IR (film)/cm^{-1} 3071, 3012, 2930, 1696 (C=O), 1587, 1561, 1439, 1402, 1290, 1182, 1118, 1014, 928, 876, 787, 753, 731, 675. ^{1}H NMR (400 MHz, $CDCl_3$) δ 10.76 (s, 1H, CHO), 7.96 (dd, *J* = 7.3, 1.7 Hz, 1H, Ar–CH), 7.60–7.54 (m, 2H, 2 × Ar–CH), 3.22 (s, 3H, CH_3), 2.52 (s, 3H, CH_3). ^{13}C NMR (101 MHz, $CDCl_3$) δ 194.4 (CHO), 140.1 (Ar–C_q), 139.1 (Ar–C_q), 136.7 (Ar–CH), 136.0 (Ar–C_q), 131.2 (Ar–CH), 127.5 (Ar–CH), 46.3 (CH_3), 19.7 (CH_3). HRMS (FTMS+pAPCI) m/z calcd. For $C_9H_{11}O_3S$ [M+H]: 199.0423; found: 199.0421.

2-Methyl-6-(cyclopropylsulfonyl)benzaldehyde (3l)

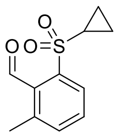

Synthesised according to general procedure **B** using 2-methylbenzaldehyde (58 μL, 0.5 mmol) and cyclopropylsulfinic acid sodium salt (25.5 mg, 0.20 mmol). Purification by flash column chromatography (20% Et_2O:pentane) afforded sulfonyl aldehyde **3l** as a white solid (34.1 mg, 76%). m.p. = 96–98 °C. R_f 0.25 (20% Et_2O:pentane). IR (film)/cm^{-1} 3053, 3019, 2967, 2863, 1703 (C=O), 1588, 1562, 1454, 1423, 1315, 1185, 1159, 1125, 1040, 887, 705, 667. ^{1}H NMR (400 MHz, $CDCl_3$) δ 10.82 (s, 1H, CHO), 7.83 (dd, *J* = 7.1, 2.0 Hz, 1H, Ar–CH), 7.57–7.51 (m, 2H, 2 × Ar–CH), 2.70 (tt, *J* = 8.0, 4.8 Hz, 1H, SO_2CH), 2.51 (s, 3H, CH_3), 1.37–1.32 (m, 2H, CH(C*H*H)$_2$), 1.10–1.05 (m, 2H, CH(CH*H*)$_2$). ^{13}C NMR (101 MHz, $CDCl_3$) δ 194.6 (CHO), 140.4 (Ar–C_q), 139.1 (Ar–C_q), 136.4 (Ar–CH), 135.8 (Ar–C_q), 131.1 (Ar–CH), 127.2 (Ar–CH), 34.2 (SO_2CH), 20.1 (CH_3), 6.4 ($SO_2CH(CH_2)_2$). HRMS (TOF–ESI$^+$) m/z calcd. For $C_9H_{11}O_3S$ [M+H]: 199.0423; found: 199.0421.

2-(Bicyclo[1.1.1]pentan-1-ylsulfonyl)-6-methylbenzaldehyde (3m)

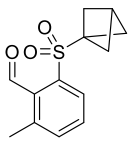

Synthesised according to general procedure **B** using 2-methylbenzaldehyde (58 μL, 0.5 mmol) and bicyclo[1.1.1]pentan-1-ylsulfinic acid sodium salt (30.8 mg, 0.20 mmol). Purification by flash column chromatography (10% EtOAc:pentane) afforded sulfonyl aldehyde **3m** as a white solid (13.5 mg, 27%). m.p. = 100–103 °C. R_f 0.08 (10% EtOAc:pentane). IR (film)/cm^{-1} 2969, 2918, 2882, 1703 (C=O), 1451, 1424, 1381, 1306, 1206, 1183, 1104, 876, 794, 681, 629, 560, 535. ^{1}H NMR (400 MHz, $CDCl_3$) δ 10.77 (s, 1H, CHO), 7.87–7.84 (m, 1H, Ar–CH), 7.60–7.55 (m, 2H, 2 × Ar–CH), 2.77 (s, 1H, $SO_2C(CH_2)_3C$*H*), 2.53 (s, 3H, CH_3), 2.12 (s, 6H,$SO_2C(C$*H*$_2)_3CH$). ^{13}C NMR (101 MHz, $CDCl_3$) δ 193.9 (CHO), 139.5 (Ar–C_q), 137.6 (Ar–C_q), 137.2 (Ar–CH), 136.2 (Ar–C_q), 131.2 (Ar–CH), 128.6(Ar–CH), 55.9 (SO_2C_q), 50.6 (3 × CH_2), 26.6 (CH), 20.8 (CH_3). HRMS (FTMS+pAPCI) m/z calcd. For $C_{13}H_{15}O_3S$ [M+H]: 251.0736; found: 251.0742.

Reaction Scope Varying the Aldehyde

2-Methoxy-6-(4-methylbenzenesulfonyl)benzaldehyde (5a)

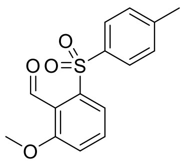

Synthesised according to general procedure **B** using 2-methoxybenzaldehyde (60 μL, 0.5 mmol) and 4-methylbenzenesulfinic acid sodium salt (35.6 mg, 0.2 mmol). Purification by flash column chromatography (20% EtOAc:pentane) afforded sulfonyl aldehyde **5a** as a while solid (30.5 mg, 53%). m.p. = 116–119 °C. R_f 0.1 (20% EtOAc:pentane). IR (film)/cm^{-1} 3086, 2945, 2848, 2736, 1707 (C=O), 1588, 1461, 1491, 1461, 1435, 1402, 1297, 1267, 1163, 1141, 1088, 1036, 969, 813, 790, 738. ^{1}H NMR (400 MHz, $CDCl_3$) δ 10.59 (s, 1H, CHO), 7.80–7.77 (m, 2H, 2 × Ar–CH), 7.71 (dd, *J* = 8.0, 0.9 Hz, 1H, Ar–CH), 7.58 (dd, *J* = 8.3, 8.3 Hz, 1H, Ar–CH), 7.31 (d, *J* = 7.7 Hz, 2H, 2 × Ar–CH), 7.19 (d, *J* = 8.3 Hz, 1H, Ar–CH), 3.86 (s, 3H, OCH_3), 2.40 (s, 3H, CH_3). ^{13}C NMR (101 MHz, $CDCl_3$) δ 191.8 (CHO), 158.8 (Ar–C_q), 144.5 (Ar–C_q), 141.6 (Ar–C_q), 138.4 (Ar–C_q), 132.5 (Ar–CH), 129.8 (2 × Ar–CH), 127.9 (2 × Ar–CH), 126.2 (Ar–C_q), 121.3 (Ar–CH), 116.5 (Ar–CH), 56.4 (OCH_3), 21.6 (CH_3). HRMS (TOF–ESI$^+$) m/z calcd. For $C_{15}H_{15}O_4S$ [M+H]: 291.0691; found: 291.0692.

2-Methyl-6-(4-methylbenzenesulfonyl)benzaldehyde (3a)

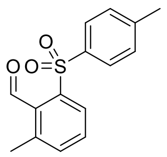

Synthesised according to general procedure **B** using 2-methylbenzaldehyde (58 μL, 0.5 mmol) and 4-methylbenzenesulfinic acid sodium salt (35.6 mg, 0.2 mmol). Purification by flash column chromatography (10–20% Et_2O:pentane) afforded sulfonyl aldehyde **3a** as an off while solid (47.1 mg, 86%). m.p. = 112–114 °C. R_f 0.21 (20% Et_2O:pentane). IR (film)/cm^{-1} 3064, 2967, 1703 (C=O), 1595, 1453, 1382, 1301, 1136, 1081, 1040, 880, 816. ^{1}H NMR (400 MHz, $CDCl_3$) δ 10.81 (s, 1H, CHO), 7.98 (d, *J* = 7.7 Hz, 1H, Ar–CH), 7.76 (d, *J* = 8.3 Hz, 2H, 2 × Ar–CH), 7.54 (t, *J* = 7.7, 7.7 Hz, 1H, Ar–CH), 7.48 (d, *J* = 7.7 Hz, 1H, Ar–CH), 7.32 (d, *J* = 8.3 Hz, 2H, 2 × Ar–CH), 2.45 (s, 3H, CH_3), 2.41 (s, 3H, CH_3). ^{13}C NMR (101 MHz, $CDCl_3$) δ 193.5 (CHO), 144.7 (Ar–C_q), 142.0 (Ar–C_q), 139.6 (Ar–C_q), 138.5 (Ar–C_q), 136.7 (Ar–CH), 134.6 (Ar–C_q), 131.2 (Ar–CH), 130.1 (2 × Ar–CH), 127.6 (2 × Ar–CH), 127.2 (Ar–CH), 21.6 (CH_3), 20.6 (CH_3). HRMS (TOF–ESI$^+$) m/z calcd. For $C_{15}H_{15}O_3S$ [M+H]: 275.0742; found: 275.0739.

Also prepared on 0.4 mmol scale which afforded sulfonyl aldehyde **3a** (89.1 mg, 81%) [note: on larger scale, a higher stirring rate (750 rpm) was necessary.]

Also prepared on 2 mmol scale which afforded sulfonyl aldehyde **3a** (356 mg, 65%)

Also prepared on 10 mmol scale which afforded sulfonyl aldehyde **3a** (2.07 g, 75%) For details of multigram scale synthesis, see page **S68.**

2-Trifluoromethyl-6-(4-methylbenzenesulfonyl)benzaldehyde (6a)

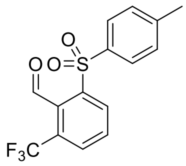

Synthesised according to general procedure **B** using 2-trifluoromethylbenzaldehyde (66 μL, 0.5 mmol) and 4-methylbenzenesulfinic acid sodium salt (35.6 mg, 0.2 mmol). Purification by flash column chromatography (10–20% Et_2O:pentane) afforded sulfonyl aldehyde **6a** as a while solid (35.5 mg, 54%). m.p. = 138–139 °C. R_f 0.09 (20% Et_2O:pentane). IR (film)/cm^{-1} 3075, 2924, 1710 (C=O), 1592, 1567, 1315, 1210, 1159, 1138, 1117, 1093, 1069, 1043, 1017, 846, 808, 754, 710, 664, 644, 579, 542. ^{1}H NMR (400 MHz, $CDCl_3$) δ 10.77 (q, $^5J_{C\text{-}F}$ = 2.9 Hz, 1H, CHO), 8.25 (d, *J* = 8.0 Hz, 1H, Ar–CH), 7.92 (d, *J* = 7.8 Hz, 1H, Ar–CH), 7.78–7.72 (m, 3H, 3 × Ar–CH), 7.35 (d, *J* = 8.2 Hz, 2H, 2 × Ar–CH), 2.42 (s, 3H, CH_3). ^{13}C NMR (101 MHz, $CDCl_3$) δ 191.9 (CHO), 145.4 (Ar–C_q), 142.2 (Ar–C_q),

137.5 (Ar–C_q), 137.4 (Ar–C_q), 132.9 (Ar–CH), 131.0 (q, $^3J_{C-F}$ = 4.8 Hz, Ar–CH), 130.9 (Ar–CH), 130.2 (2 × Ar–CH), 129.3 (q, $^2J_{C-F}$ = 31.0 Hz, Ar–C_q), 128.1 (2 × Ar–CH), 122.8 (q, $^1J_{C-F}$ = 275.5 Hz, C_qF_3), 21.6 (CH_3). $^{19}F\{^1H\}$ NMR (377 MHz, $CDCl_3$) δ –57.25. HRMS (TOF–ESI^+) m/z calcd. For $C_{15}H_{12}O_3SF_3$ [M+H]: 329.0459; found: 329.0459.

5-Methoxy-2-(4-methylbenzenesulfonyl)benzaldehyde (7a mono), 3-methoxy-2-(4-methylbenzenesulfonyl)benzaldehyde (7a mono') and 3-methoxy-2,6-di(4-methylbenzenesulfonyl)benzaldehyde (7a di)

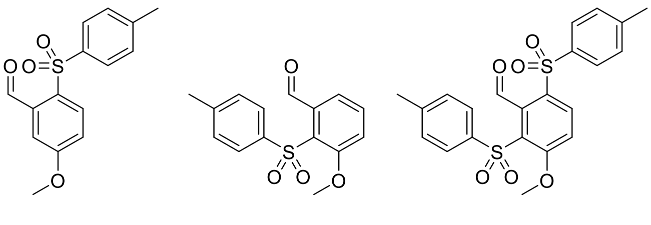

mono mono' di

Synthesised according to general procedure **B** using 3-methoxybenzaldehyde (61 µL, 0.5 mmol) and 4-methylbenzenesulfinic acid sodium salt (35.6 mg, 0.2 mmol). Purification by flash column chromatography (10–20% Et_2O:pentane to 20% EtOAc:pentane) afforded sulfonyl aldehyde **7a mono** as a while solid (19.5 mg, 34%), followed by sulfonyl aldehyde **7a mono'** as a white solid (4.9 mg, 8%), followed by **7a di** as a white solid (9.6 mg, 22%).

7a mono: m.p. = 85–87 °C. R_f 0.18 (20% Et_2O:pentane). IR (film)/cm^{-1} 3094, 2941, 1692 (C=O), 1588, 1480, 1394, 1323, 1286, 1156, 1129, 1088, 1058, 1025, 932, 816, 764, 682. ^1H NMR (400 MHz, $CDCl_3$) δ 10.81 (s, 1H, CHO), 8.14 (d, *J* = 8.5 Hz, 1H, Ar–CH), 7.75 (d, *J* = 8.5 Hz, 2H, 2 × Ar–CH), 7.48 (d, *J* = 2.8 Hz, 1H, Ar–CH), 7.31 (d, *J* = 8.5 Hz, 2H, 2 × Ar–CH), 7.21 (dd, *J* = 8.5, 2.8 Hz, 1H, Ar–CH), 3.91 (s, 3H, OCH_3), 2.41 (s, 3H, CH_3). ^{13}C NMR (101 MHz, $CDCl_3$) δ 189.4 (CHO), 163.5 (Ar–C_q), 144.5 (Ar–C_q), 139.3 (Ar–C_q), 135.7 (Ar–C_q), 134.4 (Ar–C_q), 131.9 (Ar–CH), 130.2 (2 × Ar–CH), 127.2 (2 × Ar–CH), 119.2 (Ar–CH), 113.5 (Ar–CH), 56.0 (OCH_3), 21.6 (CH_3). HRMS (TOF–ESI^+) m/z calcd. For $C_{15}H_{15}O_4S$ [M+H]: 291.0691; found: 291.0696.

7a mono': m.p. = 156–158 °C. R_f 0.06 (20% Et_2O:pentane). IR (film)/cm^{-1} 3086, 2930, 1670 (C=O), 1573, 1469, 1316, 1282, 1152, 1085, 910, 813, 775, 738. ^1H NMR (400 MHz, $CDCl_3$) δ 10.98 (d, *J* = 0.7 Hz, 1H, CHO), 7.87–7.85 (m, 2H, 2 × Ar–CH), 7.61 (ddd, *J* = 8.4, 7.6, 0.7 Hz, 1H, Ar–CH), 7.33–7.29 (m, 3H, 3 × Ar–CH), 7.09 (dd, *J* = 8.4, 1.2 Hz, 1H, Ar–CH), 3.77 (s, 3H, OCH_3), 2.44 (s, 3H, CH_3). ^{13}C NMR (101 MHz, $CDCl_3$) δ 191.7 (CHO), 157.2 (Ar–C_q), 144.4 (Ar–C_q), 139.8 (Ar–C_q), 138.8 (Ar–C_q), 135.2 (Ar–CH), 129.2 (2 × Ar–CH + Ar–C_q), 128.3 (2 × Ar–CH), 120.8 (Ar–CH), 116.4 (Ar–CH), 56.4 (OCH_3), 21.6 (CH_3). HRMS (TOF–ESI^+) m/z calcd. For $C_{15}H_{15}O_4S$ [M+H]: 291.0691; found: 291.0702.

7a di: m.p. = 200–201 °C. R_f 0.01 (20% Et_2O:pentane). IR (film)/cm^{-1} 3068, 3027, 2986, 2945, 1703 (C=O), 1595, 1494, 1319, 1185, 1156, 1107, 768, 921, 813, 768, 708. ^1H NMR (400 MHz, $CDCl_3$) δ 10.89 (s, 1H, CHO), 8.18 (d, *J* = 8.9 Hz, 1H, Ar–CH), 7.79 (d, *J* = 8.4 Hz, 2H, 2 × Ar–CH), 7.74 (d, *J* = 8.4 Hz, 2H, 2 × Ar–CH), 7.34–7.29 (m, 4H, 4 × Ar–CH), 7.04 (d, *J* = 8.9 Hz, 1H, Ar–CH), 3.87 (s, 3H, OCH_3), 2.41 (s, 6H, 2 × CH_3). ^{13}C NMR (101 MHz, $CDCl_3$) δ 191.6 (CHO), 160.7 (Ar–C_q), 145.0 (Ar–C_q), 144.8 (Ar–C_q), 142.1 (Ar–C_q), 138.3 (Ar–C_q), 136.9 (Ar–C_q), 136.8 (Ar–CH), 133.3 (Ar–C_q), 129.9 (2 × Ar–CH), 129.6 (Ar–C_q), 129.4 (2 × Ar–CH), 128.9 (2 × Ar–CH), 128.1 (2 × Ar–CH), 113.3 (Ar–CH), 56.8 (OCH_3), 21.7 (CH_3), 21.6 (CH_3). HRMS (TOF–ESI^+) m/z calcd. For $C_{22}H_{21}O_6S_2$ [M+H]: 445.0773; found: 445.0780.

5-Methyl-2-(4-methylbenzenesulfonyl)benzaldehyde (8a)

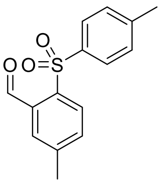

Synthesised according to general procedure **B** using 3-methylbenzaldehyde (59 μL, 0.5 mmol) and 4-methylbenzenesulfinic acid sodium salt (35.6 mg, 0.2 mmol). Purification by flash column chromatography (10–20% Et_2O:pentane) afforded sulfonyl aldehyde **8a** as a colourless oil (37.3 mg, 68%). R_f 0.23 (20% Et_2O:pentane). IR (film)/cm^{-1} 3034, 2922, 1684 (C=O), 1592, 1390, 1446, 1315, 1152, 1088, 1040, 910, 813, 727, 678. ^{1}H NMR (400 MHz, $CDCl_3$) δ 10.83 (s, 1H, CHO), 8.08 (d, *J* = 8.0 Hz, 1H, Ar–CH), 7.81 (d, *J* = 2.0 Hz, 1H, Ar–CH), 7.76 (d, *J* = 8.2 Hz, 2H, 2 × Ar–CH), 7.55 (dd, *J* = 8.0, 2.0 Hz, 1H, Ar–CH), 7.32 (d, *J* = 8.2 Hz, 2H, 2 × Ar–CH), 2.47 (s, 3H, CH_3), 2.41 (s, 3H, CH_3). ^{13}C NMR (101 MHz, $CDCl_3$) δ 189.8 (CHO), 144.9 (Ar–C_q), 144.7 (Ar–C_q), 139.8 (Ar–C_q), 138.9 (Ar–C_q), 134.1 (Ar–CH), 133.7 (Ar–C_q), 130.2 (2 × Ar–CH), 129.9 (Ar–CH), 129.7 (Ar–CH), 127.4 (2 × Ar–CH), 21.6 (CH_3), 21.4 (CH_3). HRMS (FTMS+pAPCI) m/z calcd. For $C_{15}H_{15}O_3S$ [M+H]: 275.0736; found: 275.0733

5-Trifluoromethyl-2-(4-methylbenzenesulfonyl)benzaldehyde (9a)

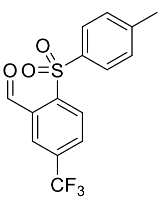

Synthesised according to general procedure **B** using 3-trifluoromethylbenzaldehyde (67 μL, 0.5 mmol) and 4-methylbenzenesulfinic acid sodium salt (35.6 mg, 0.2 mmol). Purification by flash column chromatography (10% acetone:pentane) afforded sulfonyl aldehyde **9a** as a colourless oil (32.1 mg, 49%) [note: a longer column (approx. 10 cm height, 2.5 cm width) was required]. R_f 0.19 (10% acetone:pentane). IR (film)/cm^{-1} 2919, 1698 (C=O), 1598, 1403, 1326, 1255, 1175, 1156, 1134, 1077, 909, 848, 814, 718, 661, 588, 545, 415. ^{1}H NMR (400 MHz, $CDCl_3$) δ 10.90 (s, 1H, CHO), 8.30–8.27 (m, 2H, 2 × Ar–CH), 7.99 (dd, *J* = 8.2, 1.3 Hz, 1H, Ar–CH), 7.80 (d, *J* = 8.3 Hz, 2H, 2 × Ar–CH), 7.38 (d, *J* = 8.3 Hz, 2H, 2 × Ar–CH), 2.44 (s, 3H, CH_3). ^{13}C NMR (101 MHz, $CDCl_3$) δ 188.0 (CHO), 146.0 (Ar–C_q), 145.7 (Ar–C_q), 137.4 (Ar–C_q), 135.5 (q, $^2J_{C\text{–}F}$ = 33.9 Hz, Ar–C_q), 134.4 (Ar–C_q), 130.5 (2 × Ar–CH), 130.2* (m, Ar–CH), 130.1 (Ar–CH), 127.8 (2 × Ar–CH), 126.6 (q, $^3J_{C\text{–}F}$ = 3.6 Hz, Ar–CH), 21.7 (CH_3). ^{19}F{^{1}H} NMR (377 MHz, $CDCl_3$) δ –63.42. HRMS (FTMS+pAPCI) m/z calcd. For $C_{15}H_{12}F_3O_3S$ [M+H]: 329.0454; found: 329.0453.*peak is partially obscured by adjacent singlet at δ 130.1 ppm. It was not possible to observe the quaternary *C*F_3 due to C–F coupling.

4-Methoxy-2-(4-methylbenzenesulfonyl)benzaldehyde (10a mono) and 4-methoxy-2,6-di(4-methylbenzenesulfonyl)benzaldehyde (10a di)

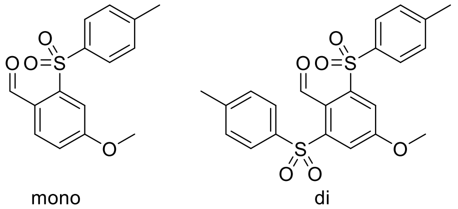

mono di

Synthesised according to general procedure **B** using 4-methoxybenzaldehyde (61 μL, 0.5 mmol) and 4-methylbenzenesulfinic acid sodium salt (35.6 mg, 0.2 mmol). Purification by flash column chromatography (10–20% Et_2O:pentane to 20% EtOAc:pentane) afforded sulfonyl aldehyde **10a mono** as a white solid (24.2 mg, 42%) followed by sulfonyl aldehyde **10a di** as a white solid (20.2 mg, 45%).

10a mono: m.p. = 158–160 °C. R_f 0.14 (20% Et_2O:pentane). IR (film)/cm^{-1} 3086, 2810, 2844, 1685 (C=O), 1592, 1491, 1310, 1238, 1200, 1152, 1028, 880, 708. ^{1}H NMR (400 MHz, $CDCl_3$) δ 10.71 (d, *J* = 0.8 Hz, 1H, CHO), 8.04 (d, *J* = 8.7 Hz, 1H, Ar–CH), 7.78 (d, *J* = 8.4 Hz, 2H, 2

× Ar–CH), 7.70 (d, *J* = 2.6 Hz, 1H, Ar–CH), 7.33 (dd, *J* = 8.7, 0.8 Hz, 2H, 2 × Ar–CH), 7.16 (ddd, *J* = 8.7, 2.6, 0.8 Hz, 1H, Ar–CH), 3.97 (s, 3H, OCH_3), 2.42 (s, 3H, CH_3). ^{13}C NMR (101 MHz, $CDCl_3$) δ 188.2 (CHO), 163.6 (Ar–C_q), 144.9 (Ar–C_q), 144.7 (Ar–C_q), 138.5 (Ar–C_q), 131.8 (Ar–CH), 130.2 (2 × Ar–CH), 127.4 (2 × Ar–CH), 126.5 (Ar–C_q), 118.5 (Ar–CH), 114.8 (Ar–CH), 56.2 (OCH_3), 21.6 (CH_3). HRMS (TOF–ESI$^+$) m/z calcd. For $C_{15}H_{15}O_4S$ [M+H]: 291.0691; found: 291.0698.

10a di: m.p. = 218–219 °C. R_f 0.03 (20% Et_2O:pentane). IR (film)/cm^{-1} 3071, 2922, 2885, 1710 (C=O), 1595, 1323, 1163, 1115, 1081, 813, 779, 667.^{1}H NMR (400 MHz, $CDCl_3$) δ 10.76 (s, 1H, CHO), 7.73 (d, *J* = 8.4 Hz, 4H, 4 × Ar–CH), 7.68 (s, 2H, 2 × Ar–CH), 7.32 (d, *J* = 8.1 Hz, 4H, 4 × Ar–CH), 3.93 (s, 3H, OCH_3), 2.41 (s, 6H, 2 × CH_3). ^{13}C NMR (101 MHz, $CDCl_3$) δ 192.3 (CHO), 160.6 (Ar–C_q), 145.3 (2 × Ar–C_q), 143.7 (2 × Ar–C_q), 137.3 (2 × Ar–C_q), 130.4 (Ar–C_q), 130.1 (4 × Ar–CH), 128.3 (4 × Ar–CH), 119.2 (2 × Ar–CH), 56.5 (OCH_3), 21.6 (2 × CH_3). HRMS (TOF–ESI$^+$) m/z calcd. For $C_{23}H_{21}NO_5NaS_2$ [M+Na+MeCN]: 478.0759; found: 478.0777.

4-Methyl-2-(4-methylbenzenesulfonyl)benzaldehyde (11a mono) and 4-methyl-2,6-di(4-methylbenzenesulfonyl)benzaldehyde (11a di)

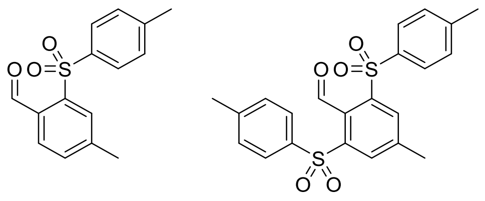

mono di

Synthesised according to general procedure **B** using 4-methylbenzaldehyde (59 μL, 0.5 mmol) and 4-methylbenzenesulfinic acid sodium salt (35.6 mg, 0.2 mmol). Purification by flash column chromatography (10–20% Et_2O:pentane then 20% EtOAc:pentane) afforded sulfonyl aldehyde **11a mono** as a white solid (17.9 mg, 33%) followed by sulfonyl aldehyde **11a di** as a white solid (11.4 mg, 27%)

11a mono: m.p. = 138–140 °C. R_f 0.19 (20% Et_2O:pentane). IR (film)/cm^{-1} 2951, 2919, 1691 (C=O), 1595, 1449, 1400, 1316, 1197, 1151, 1090, 1054, 882, 814, 707, 655, 578, 512. ^{1}H NMR (400 MHz, $CDCl_3$) δ 10.80 (d, *J* = 0.8 Hz, 1H, CHO), 8.02 (d, *J* = 0.8 Hz, 1H, Ar–CH), 7.94 (d, *J* = 7.8 Hz, 1H, Ar–CH), 7.79–7.76 (m, 2H, 2 × Ar–CH), 7.51 (ddd, *J* = 7.8, 0.8, 0.8 Hz, 1H, Ar–CH), 7.35–7.31 (m, 2H, 2 × Ar–CH), 2.53 (s, 3H, CH_3), 2.42 (s, 3H, CH_3). ^{13}C NMR (101 MHz, $CDCl_3$) δ 189.2 (CHO), 145.3 (Ar–C_q), 144.8 (Ar–C_q), 142.5 (Ar–C_q), 138.7 (Ar–C_q), 134.3 (Ar–CH), 131.4 (Ar–C_q), 130.2 (2 × Ar–CH), 129.9 (Ar–CH), 129.6 (Ar–CH), 127.4 (2 × Ar–CH), 21.8 (CH_3), 21.6 (CH_3). HRMS (FTMS+pAPCI) m/z calcd. For $C_{15}H_{15}O_3S$ [M+H]: 275.0736; found: 275.0735.

11a di: m.p. = 233–234 °C. R_f 0.04 (20% Et_2O:pentane). IR (film)/cm^{-1} 3059, 3087, 2952, 2920, 1706 (C=O), 1594, 1446, 1377, 1321, 1187, 1150, 1082, 912, 810, 664, 601, 579, 499. ^{1}H NMR (400 MHz, $CDCl_3$) δ 10.85 (s, 1H, CHO), 7.97 (s, 2H, 2 × Ar–CH), 7.74 (d, *J* = 8.2 Hz, 4H, 4 × Ar–CH), 7.32 (d, *J* = 8.2 Hz, 4H, 4 × Ar–CH), 2.48 (s, 3H, CH_3), 2.40 (s, 6H, 2 × CH_3). ^{13}C NMR (101 MHz, $CDCl_3$) δ 192.9 (CHO), 145.2 (2 × Ar–C_q), 142.1 (2 × Ar–C_q), 137.4 (2 × Ar–C_q), 136.0 (Ar–C_q), 134.4 (2 × Ar–CH), 130.1 (4 × Ar–CH + Ar–C_q), 128.3 (4 × Ar–CH), 21.6 (2 × CH_3), 21.3 (CH_3). HRMS (FTMS+pAPCI) m/z calcd. For $C_{22}H_{21}O_5S_2$ [M+H]: 429.0825; found: 429.0821.

4-(Trifluoromethyl)-2-(4-methylbenzenesulfonyl)benzaldehyde (12a mono) and 4-(trifluoromethyl)-2,6-di(4-methylbenzenesulfonyl)benzaldehyde (12a di)

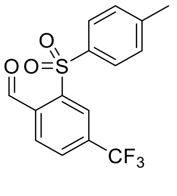

mono

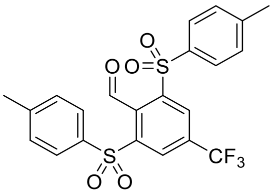

di

Synthesised according to general procedure **B** using 4-(trifluoromethyl)benzaldehyde (68 μL, 0.5 mmol) and 4-methylbenzenesulfinic acid sodium salt (35.6 mg, 0.2 mmol). Purification by flash column chromatography (10–20% EtOAc:pentane) afforded sulfonyl aldehyde **12a mono** as a white solid (20.3 mg, 31%) followed by sulfonyl aldehyde **12a di** as a white solid (2.8 mg, 6%) [note: a longer column (approx. 10 cm height, 2.5 cm width) was required].

12a mono: m.p. = 139–141 °C. R_f 0.44 (20% EtOAc:pentane). IR (film)/cm^{-1} 2923, 1698 (C=O), 1594, 1384, 1322, 1180, 1155, 1134, 1079, 853, 814, 779, 721, 578, 545, 506. ^{1}H NMR (400 MHz, $CDCl_3$) δ 10.89 (d, *J* = 0.9 Hz, 1H, CHO), 8.43 (d, *J* = 0.9 Hz, 1H, Ar–CH), 8.12 (d, *J* = 8.0 Hz, 1H, Ar–CH), 7.97 (d, *J* = 8.0 Hz, 1H, Ar–CH), 7.80 (d, *J* = 8.4 Hz, 2H, 2 × Ar–CH), 7.38 (d, *J* = 8.4 Hz, 2H, 2 × Ar–CH), 2.44 (s, 3H, CH_3). ^{13}C NMR (101 MHz, $CDCl_3$) δ 188.3 (CHO), 145.7 (Ar–C_q), 143.9 (Ar–C_q), 137.6 (Ar–C_q), 136.3 (Ar–C_q), 135.3 (q, $^2J_{C-F}$ = 34.1 Hz, Ar–C_q), 130.5 (2 × Ar–CH), 130.4 (q, $^3J_{C-F}$ = 3.3 Hz, Ar–CH), 130.3 (Ar–CH), 127.7 (2 × Ar–CH), 126.5 (q, $^3J_{C-F}$ = 3.3 Hz, Ar–CH), 122.6 (q, $^1J_{C-F}$ = 273.5 Hz, C_qF_3), 21.7 (CH_3). ^{19}F{^{1}H} NMR (377 MHz, $CDCl_3$) δ –63.16. HRMS (FTMS+pAPCI) m/z calcd. For $C_{15}H_{12}F_3O_3S$ [M+H]: 329.0454; found: 329.0461.

12a di: m.p. = 210–212 °C. R_f 0.26 (20% EtOAc:pentane). IR (film)/cm^{-1} 3068, 2919, 1707 (C=O), 1593, 1446, 1398, 1318, 1179, 1156, 1083, 910, 811, 729, 661, 574, 534. ^{1}H NMR (400 MHz, $CDCl_3$) δ 10.85 (s, 1H, CHO), 8.40 (s, 2H, , 2 × Ar–CH), 7.74 (d, *J* = 8.4 Hz, 4H, 4 × Ar–CH), 7.35 (d, *J* = 8.4 Hz, 4H, 4 × Ar–CH), 2.42 (s, 6H, 2 × CH_3). ^{19}F{^{1}H} NMR (377 MHz, $CDCl_3$) δ –62.90. HRMS (FTMS+pAPCI) m/z calcd. For $C_{22}H_{18}F_3O_5S_2$ [M+H]: 483.0542; found: 483.0563. Due to small quantity of material, it was not possible to obtain a clear ^{13}C NMR.

2-(4-Methylbenzenesulfonyl)benzaldehyde (13a mono) and 2,6-di(4-methylbenzenesulfonyl)benzaldehyde (13a di)

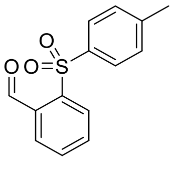

mono

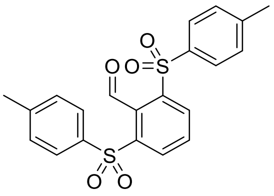

di

Synthesised according to general procedure **B** using benzaldehyde (51 μL, 0.5 mmol) and 4-methylbenzenesulfinic acid sodium salt (35.6 mg, 0.2 mmol). Purification by flash column chromatography (10–20% Et_2O:pentane) afforded sulfonyl aldehyde **13a mono** as a white solid (21.3 mg, 41%) followed by sulfonyl aldehyde **13a di** as a white solid (13.0 mg, 32%)

13a mono: m.p. = 75–76 °C [lit. m.p. = 72–73 °C]. R_f 0.18 (20% Et_2O:pentane). IR (film)/cm^{-1} 3090, 2922, 1696, 1595, 1320, 1193, 1156, 1088, 816, 768, 731.^{1}H NMR (400 MHz, $CDCl_3$)

δ 10.87 (d, J = 0.8 Hz, 1H, CHO), 8.18 (dd, J = 7.7, 1.5 Hz, 1H, Ar–CH), 8.02 (dd, J = 7.1, 2.0 Hz, 1H, Ar–CH), 7.80–7.70 (m, 4H, 4 × Ar–CH), 7.34 (d, J = 8.1 Hz, 2H, 2 × Ar–CH), 2.42 (s, 3H, CH_3). ^{13}C NMR (101 MHz, $CDCl_3$) δ 189.5 (CHO), 145.0 (Ar–C_q), 142.7 (Ar–C_q), 138.5 (Ar–C_q), 133.8 (Ar–C_q), 133.7 (2 × Ar–CH), 130.2 (2 × Ar–CH), 129.41 (Ar–CH), 129.37 (Ar–CH), 127.5 (2 × Ar–CH), 21.6 (CH_3). Analytical data (^{1}H, ^{13}C, IR, m.p.) is in agreement with the reported literature.[1b]

13a di: m.p. = 184–186 °C. R_f 0.04 (20% Et_2O:pentane). IR (film)/cm^{-1} 3071, 2960, 2922, 2885, 1710 (C=O), 1595, 1323, 1163, 1115, 1081, 779, 749, 105, 667. ^{1}H NMR (400 MHz, $CDCl_3$) δ 10.89 (s, 1H, CHO), 8.19 (d, J = 7.9 Hz, 2H, 2 × Ar–CH), 7.75–7.68 (m, 5H, 5 × Ar–CH), 7.32 (d, J = 7.7 Hz, 4H, 4 × Ar–CH), 2.40 (s, 6H, 2 × CH_3). ^{13}C NMR (101 MHz, $CDCl_3$) δ 192.7 (C=O), 145.3 (2 × Ar–C_q), 142.4 (2 × Ar–C_q), 138.6 (Ar–C_q), 137.3 (2 × Ar–C_q), 134.1 (2 × Ar–CH), 130.9 (Ar–CH), 130.1 (4 × Ar–CH), 128.4 (4 × Ar–CH), 21.7 (CH_3). HRMS (TOF–ESI$^+$) m/z calcd. For $C_{23}H_{21}NO_5NaS_2^+$ [M+Na+MeCN]: 478.0759; found: 478.0777.

2,3-Dimethyl-6-(4-methylbenzenesulfonyl)benzaldehyde (14a)

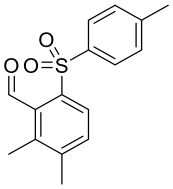

Synthesised according to general procedure **B** using 2,3-dimethylbenzaldehyde (65.2 μL, 0.5 mmol) and 4-methylbenzenesulfinic acid sodium salt (35.6 mg, 0.2 mmol). Purification by flash column chromatography (10–20% Et_2O:pentane) afforded sulfonyl aldehyde **14a** as a yellow solid (23.1 mg, 40%). m.p. = 126–127 °C. R_f 0.10 (20% Et_2O:pentane). IR (film)/cm^{-1} 3056, 2948, 2973, 2919, 1700 (C=O), 1593, 1573, 1449, 1391, 1311, 1289, 1203, 1156, 1134, 1081, 1017, 876, 814, 703, 686, 651, 596, 508, 477. ^{1}H NMR (400 MHz, $CDCl_3$) δ 10.78 (s, 1H, CHO), 7.84 (d, J = 8.1 Hz, 1H, Ar–CH), 7.73 (d, J = 8.4 Hz, 2H, 2 × Ar–CH), 7.40 (d, J = 8.1 Hz, 1H, Ar–CH), 7.31–7.28 (m, 2H, 2 × Ar–CH), 2.39 (s, 3H, CH_3), 2.35 (s, 3H, CH_3), 2.26 (s, 3H, CH_3). ^{13}C NMR (101 MHz, $CDCl_3$) δ 195.3 (CHO), 144.6 (Ar–C_q), 144.5 (Ar–C_q), 138.8 (Ar–C_q), 138.7 (Ar–C_q), 136.7 (Ar–C_q), 136.3 (Ar–C_q), 132.1 (Ar–CH), 129.9 (2 × Ar–CH), 127.6 (2 × Ar–CH), 126.8 (Ar–CH), 21.6 (CH_3), 20.7 (CH_3), 16.2 (CH_3). HRMS (TOF–ESI$^+$) m/z calcd. For $C_{16}H_{17}O_3S$ [M+H]: 289.0898; found: 289.0891.

2,4-Dimethyl-6-(4-methylbenzenesulfonyl)benzaldehyde (15a)

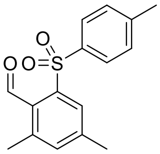

Synthesised according to general procedure **B** using 2,4-dimethylbenzaldehyde (65.2 μL, 0.5 mmol) and 4-methylbenzenesulfinic acid sodium salt (35.6 mg, 0.2 mmol). Purification by flash column chromatography (10–20% Et_2O:pentane) afforded sulfonyl aldehyde **15a** as a yellow solid (35.7 mg, 62%). m.p. = 152–156 °C. R_f 0.25 (20% Et_2O:pentane). IR (film)/cm^{-1} 2960, 2922, 2857, 1698 (C=O), 1597, 1493, 1437, 1316, 1191, 1150, 1083, 1038, 1038, 855, 814, 792, 706, 664. ^{1}H NMR (400 MHz, $CDCl_3$) δ 10.77 (s, 1H, CHO), 7.86 (s, 1H, Ar–CH), 7.75 (d, J = 8.4 Hz, 2H, 2 × Ar–CH), 7.33–7.29 (m, 3H, 3 × Ar–CH), 2.45 (s, 3H, CH_3), 2.44 (s, 3H, CH_3), 2.41 (s, 3H, CH_3). ^{13}C NMR (101 MHz, $CDCl_3$) δ 192.8 (CHO), 144.6 (Ar–C_q), 142.5 (Ar–C_q), 142.4 (Ar–C_q), 140.4 (Ar–C_q), 138.8 (Ar–C_q), 137.6 (Ar–CH), 131.4 (Ar–C_q), 130.0 (2 × Ar–CH), 127.8 (Ar–CH), 127.5 (2 × Ar–CH), 21.6 (CH_3), 21.5 (CH_3), 20.9 (CH_3). HRMS (FTMS+pAPCI) m/z calcd. For $C_{16}H_{17}O_3S$ [M+H]: 289.0893; found: 289.0894.

4-(Benzyloxy)-3-methylbenzaldehyde (S2)

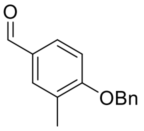

Synthesised using a modified procedure by Sparks *et al.*[8] Benzyl bromide (262 μL, 2.2 mmol) was added to a stirring solution of 4-hydroxy-3-methylbenzaldehyde (272.3 mg, 2 mmol) and potassium carbonate (552 mg, 4 mmol) in DMF (2.7 mL), then the reaction was heated to 55 °C for 3

h. The reaction was diluted with H_2O (10 mL) and the product extracted with EtOAc (3 × 10 mL). the combined organic extracts were washed with 1M $NaOH_{(aq.)}$ (30 mL) then the combined organic extracts were dried over Na_2SO_4, filtered then concentrated *in vacuo.* Azetropic removal of DMF with heptane (3 × 5 mL) afforded sulfonyl aldehyde **S2** as a white solid (406.2 mg, 90%). m.p. = 49–50 °C [lit. 51–53 °C]. IR (film)/cm^{-1} 3063, 3030, 2940, 2900, 2804, 2717, 1675 (C=O), 1593, 1495, 1418, 1384, 1326, 1260, 1229, 1160, 1117, 987, 903, 811, 732, 552, 503, 450, 425. ^{1}H NMR (400 MHz, CDCl3) δ 9.87 (s, 1H, CHO), 7.73–7.69 (m, 2H, 2 × Ar–CH), 7.47–7.36 (m, 5H, 5 × Ar–CH), 7.00 (d, *J* = 8.3 Hz, 1H, Ar–CH), 5.19 (s, 2H, CH_2), 2.34 (s, 3H, CH_3) ^{13}C NMR (101 MHz, $CDCl_3$) δ 191.1 (CHO), 161.9 (Ar–C_q), 136.3 (Ar–C_q), 131.6 (Ar–CH), 130.5 (Ar–CH), 129.6 (Ar–C_q), 128.6 (2 × Ar–CH), 128.1 (Ar–CH), 128.0 (Ar–C_q), 127.1 (2 × Ar–CH), 110.9 (Ar–CH), 70.0 (CH_2), 16.4 (CH_3). Analytical data (^{1}H, ^{13}C, m.p.) are in agreement with the reported literature.[9]

4-(Benzyloxy)-5-methyl-2-(4-methylbenzenesulfonyl)benzaldehyde (16a)

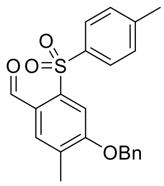

Synthesised according to general procedure **B** using 4-(benzyloxy)-3-methylbenzaldehyde (113 mg, 0.5 mmol) and 4-methylbenzenesulfinic acid sodium salt (35.6 mg, 0.2 mmol). Purification by flash column chromatography (10–20% Et_2O:pentane) afforded sulfonyl aldehyde **16a** as a yellow solid (48.5 mg, 64%). m.p. = 139–141 °C. R_f 0.20 (20% Et_2O:pentane). IR (film)/cm^{-1} 3058, 3028, 2947, 2917, 1680 (C=O), 1585, 1487, 1452, 1405, 1383, 1310, 1264, 1161, 1137, 1017, 907, 813, 732, 677, 636, 559, 530, 474. ^{1}H NMR (400 MHz, $CDCl_3$) δ 10.70 (s, 1H, CHO), 7.87 (s, 1H, Ar–CH), 7.70 (s, 1H, Ar–CH), 7.64 (d, *J* = 8.3 Hz, 2H, 2 × Ar–CH), 7.48–7.38 (m, 5H, 5 × Ar–CH), 7.27 (d, *J* = 8.3 Hz, 2H, 2 × Ar–CH), 5.31 (s, 2H, CH_2), 2.40 (s, 3H, CH_3), 2.34 (s, 3H, CH_3).^{13}C NMR (101 MHz, $CDCl_3$) δ 188.6 (CHO), 160.5 (Ar–C_q), 144.6 (Ar–C_q), 142.0 (Ar–C_q), 139.0 (Ar–C_q), 135.5 (Ar–C_q), 133.4 (Ar–C_q), 131.8 (Ar–CH), 130.1 (2 × Ar–CH), 128.8 (2 × Ar–CH), 128.4 (Ar–CH), 127.3 (2 × Ar–CH), 127.2 (2 × Ar–CH), 126.2 (Ar–C_q), 111.8 (Ar–CH), 70.7 (CH_2), 21.6 (CH_3), 16.4 (CH_3). HRMS (TOF–ESI$^+$) m/z calcd. For $C_{22}H_{21}O_4S$ [M+H]: 381.1161; found: 381.1154.

4-(Allyloxy)-3-methylbenzaldehyde (S3)

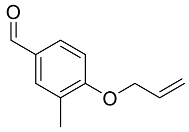

Synthesised according to a modified procedure from Sarkar *et. al*.[10] Allyl bromide (208 μL, 2.4 mmol) was added to a stirring solution of 4-hydroxy-3-methylbenzaldehyde (272 mg, 2 mmol) and potassium carbonate (386 mg, 2.8 mmol) in acetone (4 mL) at rt. The reaction mixture was heated to reflux for 4 h. The reaction mixture was diluted with water (5 mL) and the product extracted from the aquous layer with EtOAc (3 × 10 mL). The combine organic extracts were washed with 1 M $NaOH_{(aq.)}$ (30 mL), dried over Na_2SO_4, filtered then concentrated *in vacuo* to afford aldehyde **S3** as a yellow oil (310 mg, 88%). IR (film)/cm^{-1} 2919, 2718, 1679 (C=O), 1596, 1495, 1450, 1421, 1378, 1318, 1252, 1230, 1163, 1117, 991, 924, 808, 774, 654, 557, 500, 480, 443. ^{1}H NMR (400 MHz, $CDCl_3$) δ 9.86 (s, 1H, CHO), 7.71–7.68 (m, 2H, 2 × Ar–CH), 6.93–6.90 (m, 1H, Ar–CH), 6.08 (ddt, *J* = 17.3, 10.5, 5.0 Hz, 1H, CH_2CH), 5.46 (dd, *J* = 17.3, 1.7 Hz, 1H, $CH_2CH{=}C\mathit{H}H$), 5.33 (dd, *J* = 10.5, 1.7 Hz, 1H, $CH_2CH{=}CH\mathit{H}$), 4.66–4.64 (m, 2H, CH_2), 2.30 (s, 3H, CH_3). ^{13}C NMR (101 MHz, $CDCl_3$) δ 191.1 (CHO), 161.8 (Ar–C_q), 132.5 (Ar–CH), 131.6 (Ar–CH), 130.5 (Ar–CH), 129.5 (Ar–C_q), 127.8 (Ar–C_q), 117.7 ($CH_2\mathit{C}H{=}CHH$), 110.7 ($CH_2CH{=}\mathit{C}HH$), 68.8 (CH_2), 16.3 (CH_3). Analytical data (^{1}H, ^{13}C) is in agreement with the reported literature.[10]

4-(Allyloxy)-5-methyl-2-(4-methylbenzenesulfonyl)benzaldehyde (17a)

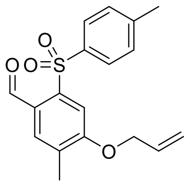

Potassium carbonate (55 mg, 0.4 mmol) and copper(II) fluoride (40 mg, 0.4 mmol) were added sequentially to a microwave vial which was then flame dried under argon until a blue colour just appears (ca. 2–5 seconds). The microwave vial was allowed to cool to room temperature and copper(II) acetate (18 mg, 0.1 mmol), β-alanine (4.5 mg, 0.05 mmol), 4-methylbenzenesulfinic acid sodium salt (35.6 mg, 0.2 mmol) and a solution of aldehyde **S3** in HFIP (88 mg, 0.5 mmol in 1 mL HFIP) were added sequentially under argon. The vial was sealed and submerged in an oil bath preheated to 100 °C for 18 h [Stirring rate set to 500 rpm]. The reaction was allowed to cool to room temperature, diluted with EtOAc (5 mL) and washed with a saturated aqueous solution of ammonium chloride (10 mL), The product was extracted from the aqueous phase with EtOAc (2 × 10 mL) and the combined organic extracts were dried over Na_2SO_4, filtered and concentrated *in vacuo*. Purification by flash column chromatography (10% EtOAc:pentane) afforded sulfonyl aldehyde **17a** as a white solid (26.5 mg, 40%) [note: a longer column was required (approx. 10 cm height, 2.5 cm width)]. m.p. = 119–121°C. R_f 0.22 (10% EtOAc:pentane). IR (film)/cm^{-1} 3068, 2980, 2919, 1681 (C=O), 1588, 1487, 1453, 1408, 1312, 1267, 1164, 1140, 1086, 1021, 931, 814, 748, 681, 586, 557, 531. ^{1}H NMR (400 MHz, $CDCl_3$) δ 10.69 (s, 1H, CHO), 7.86 (s, 1H, Ar–CH), 7.74 (d, *J* = 8.3 Hz, 2H, 2 × Ar–CH), 7.63 (s, 1H, Ar–CH), 7.31 (d, *J* = 8.3 Hz, 2H, 2 × Ar–CH), 6.08 (ddt, *J* = 17.3, 10.6, 5.2 Hz, 1H, CH_2CH=CHH), 5.49 (dd, *J* = 17.3, 1.5 Hz, 1H, CH_2CH=C*H*H), 5.39 (dd, *J* = 10.6, 1.5 Hz, 1H, CH_2CH=CH*H*), 4.76 (m, 2H) 2.41 (s, 3H), 2.31 (s, 3H). ^{13}C NMR (101 MHz, $CDCl_3$) δ 188.6 (CHO), 160.5 (Ar–C_q), 144.7 (Ar–C_q), 142.0 (Ar–C_q), 139.1 (Ar–C_q), 133.2 (Ar–C_q), 131.82 (Ar–CH), 131.77 (Ar–CH), 130.2 (2 × Ar–CH), 127.2 (2 × Ar–CH), 126.1 (Ar–C_q), 118.5 (CH_2CH=CHH), 111.4 (CH_2CH=*C*HH), 69.5 (CH_2), 21.6 (CH_3), 16.3 (CH_3). HRMS (TOF–ESI$^+$) m/z calcd. For $C_{18}H_{19}O_4S$ [M+H]: 331.1004; found: 331.1004.

3-(4-Methylbenzenesulfonyl)-[1,1'-biphenyl]-2-carboxaldehyde (18a)

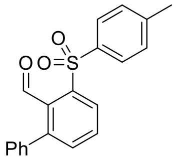

Synthesised according to general procedure **B** using biphenyl-2-carboxaldehyde (80.6 μL, 0.5 mmol) and 4-methylbenzenesulfinic acid sodium salt (35.6 mg, 0.2 mmol). Purification by flash column chromatography (10–20% Et_2O:pentane) afforded sulfonyl aldehyde **18a** as a colourless oil (58.4 mg, 87%). R_f 0.19 (20% Et_2O:pentane). IR (film)/cm^{-1} 3055, 3026, 2947, 2918, 2850, 2730, 1706 (C=O), 1592, 1492, 1445, 1381, 1315, 1202, 1182, 1167, 1158, 1084, 1019, 910, 811, 760, 732, 702, 680, 654, 578, 547. ^{1}H NMR (400 MHz, $CDCl_3$) δ 10.42 (s, 1H, CHO), 8.19 (dd, *J* = 7.9, 1.3 Hz, 1H, Ar–CH), 7.84 (d, *J* = 8.4 Hz, 2H, 2 × Ar–CH), 7.68 (dd, *J* = 7.8, 7.8 Hz, 1H, Ar–CH), 7.60 (dd, *J* = 7.7, 1.3 Hz, 1H, Ar–CH), 7.42–7.39 (m, 3H, 3 × Ar–CH), 7.35 (d, *J* = 8.6 Hz, 2H, 2 × Ar–CH), 7.25–7.23 (m, 2H, 2 × Ar–CH), 2.43 (s, 3H, CH_3). ^{13}C NMR (101 MHz, $CDCl_3$) δ 194.0 (CHO), 144.5 (Ar–C_q), 143.2 (Ar–C_q), 140.9 (Ar–C_q), 138.6 (Ar–C_q), 137.5 (Ar–C_q), 136.4 (Ar–C_q), 135.6 (Ar–CH), 130.7 (Ar–CH), 129.8 (2 × Ar–CH), 129.6 (2 × Ar–CH), 128.9 (Ar–CH), 128.6 (2 × Ar–CH), 128.4 (Ar–CH), 128.1 (2 × Ar–CH), 21.6 (CH_3). HRMS (TOF–ESI$^+$) m/z calcd. For $C_{20}H_{17}O_3S$ [M+H]: 337.0898; found: 337.0897.

3-(4-Methylbenzenesulfonyl)-2-naphthaldehyde (19a)

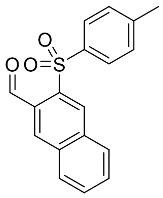

Synthesised according to general procedure **B** using 2-napthaldehyde (78.1 mg, 0.5 mmol) and 4-methylbenzenesulfinic acid sodium salt (35.6 mg, 0.2 mmol). Purification by flash column chromatography (10–20% Et_2O:pentane) afforded sulfonyl aldehyde **19a** as a white solid (59.8 mg, 96%). m.p. = 159–160 °C. R_f 0.13 (20% Et_2O:pentane). IR (film)/cm^{-1} 3054, 2946, 2916, 2950, 1689 (C=O), 1617, 1593, 1491, 1442, 1379, 1307, 1149, 1126,

1083, 1012, 908, 812, 751, 678, 613, 453. ^{1}H NMR (400 MHz, $CDCl_3$) δ 10.81 (s, 1H, CHO), 8.80 (s, 1H, Ar–CH), 8.55 (s, 1H, Ar–CH), 8.11–8.04 (m, 2H, 2 × Ar–CH), 7.83–7.73 (m, 4H, 4 × Ar–CH), 7.33–7.31 (m, 2H, 2 × Ar–CH), 2.41 (s, 3H, CH_3). ^{13}C NMR (101 MHz, $CDCl_3$) δ 189.4 (CHO), 144.8 (Ar–C_q), 138.7 (Ar–C_q), 137.5 (Ar–C_q), 134.2 (Ar–C_q), 133.9 (Ar–C_q), 132.1 (Ar–CH), 131.5 (Ar–CH), 130.3 (Ar–CH), 130.2 (2 × Ar–CH), 130.1 (Ar–CH), 129.9 (Ar–CH), 129.7 (Ar–C_q), 129.5 (Ar–CH), 127.5 (2 × Ar–CH), 21.6 (CH_3). HRMS (FTMS+pAPCI) m/z calcd. For $C_{18}H_{15}O_3S$ [M+H]: 311.0736; found: 311.0735.

6-Methoxy-3-(4-methylbenzenesulfonyl)-2-naphthaldehyde (20a)

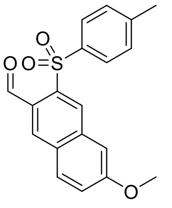

Synthesised according to general procedure **B** using 6-methoxy-2-napthaldehyde (93.0 mg, 0.5 mmol) and 4-methylbenzenesulfinic acid sodium salt (35.6 mg, 0.2 mmol) [note: reaction time = 22 h.]. Purification by flash column chromatography (20% EtOAc:hexane) afforded sulfonyl aldehyde **20a** as an off white solid (48.6 mg, 71%). m.p. = 231–232 °C. R_f 0.18 (20% Et_2O:hexane). IR (film)/cm^{-1} 3015, 2968, 2931, 2885, 2844, 1685 (C=O), 1611, 1491, 1413, 1383, 1299, 1243, 1173, 1142, 1121, 1081, 1016, 956, 907, 870, 819, 708, 676, 611, 491, 442. ^{1}H NMR (400 MHz, $CDCl_3$) δ 10.76 (s, 1H, CHO), 8.67 (s, 1H, Ar–CH), 8.49 (s, 1H, Ar–CH), 7.93 (d, *J* = 8.9 Hz, 1H, Ar–CH), 7.82–7.80 (m, 2H, 2 × Ar–CH), 7.39–7.36 (m, 2H, 2 × Ar–CH), 7.35–7.30 (m, 2H, 2 × Ar–CH), 4.01 (s, 3H, OCH_3), 2.41 (s, 3H, CH_3). ^{13}C NMR (101 MHz, $CDCl_3$) δ 189.2 (CHO), 161.1 (Ar–C_q), 144.7 (Ar–C_q), 138.9 (Ar–C_q), 138.2 (Ar–C_q), 136.0 (Ar–C_q), 131.8 (Ar–CH), 131.5 (Ar–CH), 130.1 (2 × Ar–CH), 129.9 (Ar–CH), 129.6 (Ar–C_q), 127.6 (Ar–C_q), 127.5 (2 × Ar–CH), 123.1 (Ar–CH), 107.2 (Ar–CH), 55.7 (OCH_3), 21.6 (CH_3). HRMS (TOF–ESI$^+$) m/z calcd. For $C_{19}H_{17}O_4S$ [M+H]: 341.0848; found: 341.0848.

5-Chloro-2-(4-methylbenzenesulfonyl)benzaldehyde (21a)

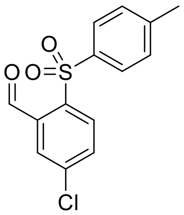

Synthesised according to general procedure **B** using 3-chlorobenzaldehyde (57 μL, 0.5 mmol) and 4-methylbenzenesulfinic acid sodium salt (35.6 mg, 0.2 mmol). Purification by flash column chromatography (10–20% Et_2O:pentane) afforded sulfonyl aldehyde **21a** as a colourless oil (23.2 mg, 40%). R_f 0.23 (20% Et_2O:pentane). IR (film)/cm^{-1} 3086, 2956, 2922, 1696 (C=O), 1595, 1322, 1185, 1156, 1129, 1084, 898, 746, 708. ^{1}H NMR (400 MHz, $CDCl_3$) δ 10.81 (s, 1H, CHO), 8.13 (d, *J* = 8.4 Hz, 1H, Ar–CH), 7.97 (d, *J* = 2.2 Hz, 1H, Ar–CH), 7.77 (d, *J* = 8.4 Hz, 2H, 2 × Ar–CH), 7.71 (dd, *J* = 8.4, 2.2 Hz, 1H, Ar–CH), 7.35 (d, *J* = 8.1 Hz, 2H, 2 × Ar–CH), 2.43 (s, 3H, CH_3). ^{13}C NMR (101 MHz, $CDCl_3$) δ 188.2 (CHO), 145.3 (Ar–C_q), 141.0 (Ar–C_q), 140.7 (Ar–C_q), 138.1 (Ar–C_q), 135.0 (Ar–C_q), 133.4 (Ar–CH), 131.0 (Ar–CH), 130.4 (2 × Ar–CH), 129.4 (Ar–CH), 127.5 (2 × Ar–CH), 21.6 (CH_3). HRMS (FTMS+pAPCI) m/z calcd. For $C_{14}H_{12}O_3S^{35}Cl$ [M+H]: 295.0190; found: 295.0187.

5-Bromo-2-(4-methylbenzenesulfonyl)benzaldehyde (22a)

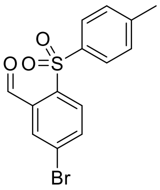

Synthesised according to general procedure **B** using 3-bromobenzaldehyde (58.0 μL, 0.5 mmol) and 4-methylbenzenesulfinic acid sodium salt (35.6 mg, 0.2 mmol). Purification by flash column chromatography (10–20% Et_2O:pentane) afforded sulfonyl aldehyde **22a** as a white solid (26.7 mg, 39%). m.p. = 91–92 °C. R_f 0.30 (20% Et_2O:pentane). IR (film)/cm^{-1} 3082, 2948, 2917, 1692 (C=O), 1593, 1570, 1552,1454, 1398, 1320, 1288, 1182, 1155, 1080, 875, 813, 738, 656, 588, 553, 404. ^{1}H NMR (400 MHz, $CDCl_3$) δ 10.81 (s, 1H, CHO), 8.12 (d, *J* = 2.2 Hz, 1H, Ar–CH), 8.04 (d, *J* = 8.4 Hz, 1H, Ar–CH), 7.88 (dd, *J* = 8.4, 2.2 Hz, 1H, Ar–CH), 7.77 (d, *J* = 8.4 Hz, 2H, 2 × Ar–CH), 7.35 (d, *J* = 8.4 Hz, 2H, 2 × Ar–CH), 2.43 (s, 3H, CH_3). ^{13}C NMR (101 MHz, $CDCl_3$) δ 188.1 (CHO), 145.3 (Ar–C_q), 141.6 (Ar–C_q), 138.1 (Ar–C_q), 136.5 (Ar–CH), 134.8 (Ar–C_q), 132.4 (Ar–CH), 130.9 (Ar–CH), 130.4 (2 × Ar–CH), 129.0 (Ar–C_q),

127.5 (2 × Ar–CH), 21.6 (CH_3). HRMS (FTMS+pAPCI) m/z calcd. For $C_{14}H_{12}O_3S^{79}Br$ [M+H]: 338.9865; found: 338.9692.

Methyl 3-formyl-4-(4-methylbenzenesulfonyl)benzoate (23a)

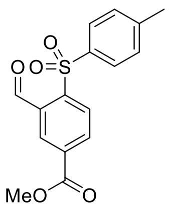

Synthesised according to general procedure **B** using Methyl 3-formylbenzoate (82.1 mg, 0.5 mmol) and 4-methylbenzenesulfinic acid sodium salt (35.6 mg, 0.2 mmol). Purification by flash column chromatography (10–20% Et_2O:pentane) afforded sulfonyl aldehyde **23a** as a white solid (23.8 mg, 37%). m.p. = 124–126 °C. R_f 0.13 (20% Et_2O:pentane). IR (film)/cm^{-1} 2919, 2950, 1729 (C=O aldehyde), 1693 (C=O ester), 1594, 1436, 1322, 1291, 1243, 1196, 1177, 1155, 1129, 1086, 814, 737, 660, 590, 552, 530.^{1}H NMR (400 MHz, $CDCl_3$) δ 10.88 (s, 1H, CHO), 8.62 (d, *J* = 1.8 Hz, 1H, Ar–CH), 8.37 (dd, *J* = 8.2, 1.8 Hz, 1H, Ar–CH), 8.23 (d, *J* = 8.2 Hz, 1H, Ar–CH), 7.79 (d, *J* = 8.2 Hz, 2H, 2 × Ar–CH), 7.36 (d, *J* = 8.2 Hz, 2H, 2 × Ar–CH), 3.97 (s, 3H, OCH_3), 2.43 (s, 3H, CH_3). ^{13}C NMR (101 MHz, $CDCl_3$) δ 188.6 (CHO), 164.7 (Ar–C_q), 146.1 (Ar–C_q), 145.5 (Ar–C_q), 137.7 (Ar–C_q), 135.0 (Ar–C_q), 134.2 (Ar–CH), 134.1 (Ar–C_q), 130.5 (Ar–CH), 130.4 Ar–C_q (2 × Ar–CH), 129.6 (Ar–CH), 127.7 (2 × Ar–CH), 52.9 (OCH_3), 21.7 (CH_3).HRMS (TOF–ESI$^+$) m/z calcd. For $C_{16}H_{15}O_5S$ [M+H]: 319.0640; found: 319.0637.

***N*-(3'-Formyl-[1,1'-biphenyl]-4-yl)acetamide (S4)**

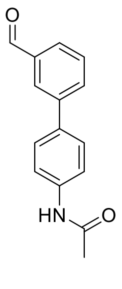

3-Formylphenylboronic acid (300 mg, 2 mmol), N-(4-iodophenyl)acetamide (553 mg, 2.12 mmol) and palladium acetate (22.4 mg, 0.1 mmol), Isopropanol (1.3 mL), water (1.3 mL), and potassium carbonate (552 mg, 4 mmol) were added sequentially to a round bottom flask and stirred at rt for 18 h. The reaction was concentrated *in vacuo* and purification by flash column chromatography (30% acetone;pentane) afforded aldehyde **S4** as a while solid (188.7 mg, 39%). m.p. = 164–165 °C. R_f 0.25 (30% acetone;pentane). IR (film)/cm^{-1} 3298 (N–H), 3186, 3112, 2360, 1694 (C=O aldehyde), 1670 (C=O amide), 1531, 1371, 1320, 1183, 794, 689. ^{1}H NMR (400 MHz, $CDCl_3$) δ 10.09 (s, 1H, CHO), 8.08 (dd, *J* = 1.9,1.9 Hz, 1H, Ar–CH), 7.84 (dd, *J* = 7.9, 1.9 Hz, 2H, 2 × Ar–CH), 7.68–7.52 (m, 5H, 5 × Ar–CH), 7.45 (s, 1H, N–H), 2.23 (s, 3H, CH_3). ^{13}C NMR (101 MHz, $CDCl_3$) δ 192.4 (CHO), 168.4 (C=O amide), 141.4 (Ar–C_q), 137.8 (Ar–C_q), 136.9 (Ar–C_q), 135.5 (Ar–C_q), 132.7 (Ar–CH), 129.5 (Ar–CH), 128.5 (Ar–CH), 127.71 (Ar–CH), 127.65 (2 × Ar–CH), 120.2 (2 × Ar–CH), 24.7 (CH_3). HRMS (TOF–ESI$^+$) m/z calcd. For $C_{15}H_{14}NO_2$ [M+H]: 240.1025; found: 240.1029

***N*-(3'-Formyl-4'-(4-methylbenzenesulfonyl)-[1,1'-biphenyl]-4-yl)acetamide (24a)**

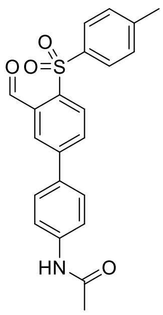

Synthesised according to general procedure B using *N*-(3'-Formyl-[1,1]' biphenyl]-4-yl)acetamide **S4** (119.6 mg, 0.5 mmol) and 4-methylbenzenesulfinic acid sodium salt (35.6 mg, 0.2 mmol). Purification by flash column chromatography (40% acetone:pentane) afforded sulfonyl aldehyde **24a** as an off white solid (37.8 mg, 48%). R_f 0.23 (40% acetone:pentane). IR (film)/cm^{-1} 3369 (N–H), 3278, 3056, 2362, 2336, 1693 (C=O), 1594, 1525, 1372, 1320, 1300, 1156, 826, 752, 670, 659, 559, 539, 404. ^{1}H NMR (400 MHz, $CDCl_3$) δ 10.89 (s, 1H, CHO), 8.21 (d, *J* = 8.2 Hz, 1H, Ar–CH), 8.18 (d, *J* = 2.1 Hz, 1H, Ar–CH), 7.91 (dd, *J* = 8.2, 2.1 Hz, 1H, Ar–CH), 7.83–7.77 (m, 2H, 2 × Ar–CH), 7.67–7.61 (m, 2H, 2 × Ar–CH), 7.60–7.49 (m, 3H, 2 × Ar–CH + N–H), 7.35 (dd, *J* = 8.6, 0.7 Hz, 2H, 2 × Ar–CH), 2.42 (s, 3H, $COCH_3$), 2.22 (s, 3H, $ArCH_3$). ^{13}C NMR (101 MHz, $CDCl_3$) δ 189.6 (CHO), 168.5 (C=O amide), 145.8 (Ar–C_q), 144.9 (Ar–C_q), 140.6 (Ar–C_q), 139.0 (Ar–C_q), 138.6 (Ar–C_q), 134.2 (Ar–C_q), 133.4 (Ar–C_q), 131.2 (Ar–CH), 130.3 (2 × Ar–CH), 130.2 (Ar–CH), 127.9 (2 × Ar–CH), 127.5 (2 × Ar–CH),

127.3 (Ar–CH), 120.2 (2 × Ar–CH), 24.7 (COCH3), 21.6 (ArCH3). HRMS (TOF–ESI+) m/z calcd. For $C_{24}H_{23}N_2O_4S$ [M+H+MeCN]: 435.1397; found: 435.1397.

4'-(Hydroxymethyl)-[1,1'-biphenyl]-3-carbaldehyde (S5)

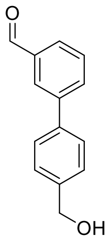

3-Formylphenylboronic acid (300 mg, 2 mmol), 4-iodobenzyl alcohol (496 mg, 2.12 mmol) and palladium acetate (22.4 mg, 0.1 mmol), Isopropanol (1.3 mL), water (1.3 mL), and potassium carbonate (552 mg, 4 mmol) were added sequentially to a round bottom flask and stirred at rt for 18 h. The reaction was concentrated *in vacuo* and purification by flash column chromatography (20% acetone;pentane) afforded aldehyde **S5** as a while solid (212.8 mg, 50%). m.p. = 58–59 °C. R_f 0.17 (20% acetone;pentane. IR (film)/cm^{-1} 3369 (O–H), 2825, 2742, 1692 (C=O), 1595, 1443, 1385, 1180, 1161, 993, 898, 788, 689, 648, 570, 500, 431. ^{1}H NMR (400 MHz, $CDCl_3$) δ 10.10 (s, 1H, CHO), 8.11 (d, *J* = 1.8 Hz, 1H, Ar–CH), 7.88 (dd, *J* = 7.6, 1.8 Hz, 2H, 2 × Ar–CH), 7.74–7.58 (m, 3H, 3 × Ar–CH), 7.50 (d, *J* = 8.3 Hz, 2H, 2 × Ar–CH), 4.79 (d, *J* = 6.0 Hz, 2H, C*H2*OH), 1.76 (t, *J* = 6.0 Hz, 1H, OH). ^{13}C NMR (101 MHz, $CDCl_3$) δ 192.3 (CHO), 141.8 (Ar–C_q), 140.7 (Ar–C_q), 139.0 (Ar–C_q), 136.9 (Ar–C_q), 133.0 (Ar–CH), 129.5 (Ar–CH), 128.7 (Ar–CH), 128.0 (Ar–CH), 127.6 (2 × Ar–CH), 127.3 (2 × Ar–CH), 65.0 (CH_2). HRMS (EI$^+$) m/z calcd. For $C_{14}H_{12}O_2$ [M]: 212.0832; found: 212.0839.

4'-(Hydroxymethyl)-4-(4-methylbenzenesulfonyl)-[1,1'-biphenyl]-3-carbaldehyde (25a)

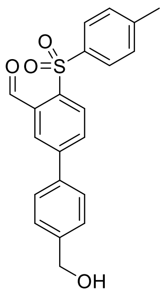

Synthesised according to general procedure B using 4'-(hydroxymethyl)-[1,1']-biphenyl]-3-carbaldehyde **S5** (106.1 mg, 0.5 mmol) and 4-methylbenzenesulfinic acid sodium salt (35.6 mg, 0.2 mmol). Purification by flash column chromatography (30% acetone:pentane) afforded sulfonyl aldehyde **25a** as a white solid (37.5 mg, 51%). R_f 0.25 (30% acetone:pentane). IR (film)/cm^{-1} 3521 (OH), 2919, 1692 (C=O), 1592, 1401, 1316, 1299, 1180, 1156, 1136, 1091, 814, 747, 665, 582, 541. ^{1}H NMR (400 MHz, $CDCl_3$) δ 10.91 (s, 1H, CHO), 8.31–8.15 (m, 2H, 2 × Ar–CH), 7.95 (dd, *J* = 8.2, 2.1 Hz, 1H, Ar–CH), 7.85–7.78 (m, 2H, 2 × Ar–CH), 7.62 (d, *J* = 8.2 Hz, 2H, 2 × Ar–CH), 7.49 (d, *J* = 8.2 Hz, 2H, 2 × Ar–CH), 7.35 (d, *J* = 8.4 Hz, 2H, 2 × Ar–CH), 4.78 (d, *J* = 5.7 Hz, 2H, CH_2), 2.43 (s, 3H, CH_3), 1.84 (s, 1H, O–H). ^{13}C NMR (101 MHz, $CDCl_3$) δ 189.6 (CHO), 146.3 (Ar–C_q), 145.0 (Ar–C_q), 142.0 (Ar–C_q), 141.0 (Ar–C_q), 138.7 (Ar–C_q), 137.2 (Ar–C_q), 134.3 (Ar–C_q), 131.6 (Ar–CH), 130.3 (Ar–CH), 130.2 (2 × Ar–CH), 127.73 (Ar–CH), 127.67 (2 × Ar–CH), 127.53 (2 × Ar–CH), 127.46 (2 × Ar–CH), 64.8 (CH_2), 21.7 (CH_3). HRMS (TOF–ESI$^+$) m/z calcd. For $C_{21}H_{19}O_4S$ [M+H]: 367.1004; found: 367.1002.

***N*-(3-Formyl-2-(4-methylbenzenesulfonyl)phenyl)methanesulfonamide (26a)**

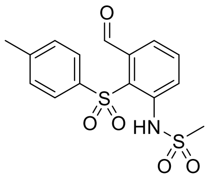

Synthesised according to general procedure **B** using N-(3-formylphenyl)methanesulfonamide (99.6 mg, 0.5 mmol) and 4-methylbenzenesulfinic acid sodium salt (35.6 mg, 0.2 mmol). Purification by flash column chromatography (100% CH_2Cl_2) afforded sulfonyl aldehyde **26a** as a yellow amorphous gum (21.2 mg, 30%). R_f 0.51 (100% CH_2Cl_2). IR (film)/cm^{-1} 3253 (N–H), 2932, 1695 (C=O), 1580, 1457, 1374, 1331, 1306, 1269, 1149, 971, 857, 811, 727, 654, 554, 464. ^{1}H NMR (400 MHz, $CDCl_3$) δ 10.74 (s, 1H, CHO), 9.51 (s, 1H, NH), 7.93 (dd, *J* = 8.2, 1.3 Hz, 1H, Ar–CH), 7.80 (d, *J* = 8.6 Hz, 2H, 2 × Ar–CH), 7.68 (dd, *J* = 8.2,8.2 Hz, 1H, Ar–CH), 7.50 (dd, *J* = 8.2, 1.3 Hz, 1H, Ar–CH), 7.39 (d, *J* = 8.6 Hz, 2H, 2 × Ar–CH), 3.01 (s, 3H, SO_2CH_3), 2.44 (s, 3H, $ArCH_3$). ^{13}C NMR (101 MHz, $CDCl_3$) δ 190.3 (CHO), 146.0 (Ar–C_q), 138.6 (Ar–C_q), 137.8 (Ar–C_q), 137.5 (Ar–C_q), 135.2 (Ar–CH), 130.5 (2 × Ar–CH), 127.2 (Ar–C_q), 126.8 (2 × Ar–CH), 124.6 (Ar–CH), 123.5 (Ar–CH),

40.6 (SO_2CH_3), 21.7 ($ArCH_3$). HRMS (FTMS+pAPCI) m/z calcd. For $C_{15}H_{16}NO_5S_2$ [M+H]: 354.0464; found: 354.458

3-(4-Methylbenzenesulfonyl)-4-pyridinecarboxaldehyde (27a)

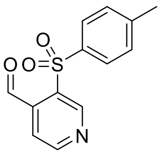

Potassium carbonate (55 mg, 0.4 mmol) and copper(II) fluoride (40 mg, 0.4 mmol) were added sequentially to a microwave vial which was then flame dried under argon until a blue colour just appeared (ca. 2–5 seconds). The microwave vial was allowed to cool to room temperature and copper(II) acetate (18 mg, 0.1 mmol), β-alanine (4.5 mg, 0.05 mmol), 4-methylbenzenesulfinic acid sodium salt (35.6 mg, 0.2 mmol), 4-pyridinecarboxaldehyde (58.0 μL, 0.5 mmol) and HFIP (1 mL) were added to a microwave vial sequentially under argon. The vial was sealed and submerged in an oil bath preheated to 100 °C for 18 h [Stirring rate set to 500 rpm]. The reaction was allowed to cool to room temperature, diluted with EtOAc (5 mL) and the organic phase was washed with a saturated aqueous solution of sodium bicarbonate (10 mL). The product was extracted from the aqueous phase with EtOAc (2 × 10 mL) and the combined organic extracts were dried over Na_2SO_4, filtered and concentrated *in vacuo*. Purification by flash column chromatography (10% Et_2O:CH_2Cl_2 then 100% EtOAc) afforded sulfonyl aldehyde **27a** as a white solid (13.0 mg, 25%). m.p. = 125–127 °C. R_f 0.37 (EtOAc). IR (film)/cm^{-1} 3083, 3052, 2918, 1703 (C=O), 1593, 1541, 1398, 1319, 1204, 1153, 1108, 1076, 818, 746, 720, 657, 581, 554, 500. ^{1}H NMR (400 MHz, $CDCl_3$) δ 10.90 (s, 1H, CHO), 9.26 (brs, 2H, 2 × Ar–CH), 7.85–7.82 (m, 3H, 3 × Ar–CH), 7.39 (d, *J* = 8.2 Hz, 2H, 2 × Ar–CH), 2.45 (s, 3H, CH_3).^{1}H NMR (400 MHz, DMSO) δ 10.64 (s, 1H, CHO), 9.32 (s, 1H, Ar–CH), 9.09 (d, *J* = 5.0 Hz, 1H, Ar–CH), 8.02–7.99 (m, 2H, 2 × Ar–CH), 7.73 (d, *J* = 5.0 Hz, 1H, Ar–CH), 7.49 (m, 2H, 2 × Ar–CH), 2.39 (s, 3H, CH_3). ^{13}C NMR (101 MHz, $CDCl_3$)[a] δ 188.7 (CHO), 155.3 (Ar–CH), 150.2 (Ar–CH), 145.8 (Ar–C_q), 139.7 (Ar–C_q), 137.5 (Ar–C_q), 130.6 (2 × Ar–CH), 127.8 (2 × Ar–CH), 121.4 (Ar–CH), 21.7 (CH_3). ^{13}C NMR (101 MHz, DMSO) δ 190.1 (CHO), 156.0 (Ar–CH), 149.8 (Ar–CH), 145.6 (Ar–C_q), 140.8 (Ar–C_q), 137.2 (Ar–C_q), 135.6[b] (Ar–C_q), 130.6 (2 × Ar–CH), 127.9 (2 × Ar–CH), 122.0 (Ar–CH), 21.1 (CH_3). HRMS (FTMS+pAPCI) m/z calcd. For $C_{13}H_{21}NO_3S$ [M+H]: 262.0532; found: 262.0539. [note significant broadening of pyridyl NMR signals was observed in $CDCl_3$, such broadening was not observed in DMSO-d_6 [a]Quaternary ^{13}C signal on pyridyl moiety bound to SO_2 was not possible to observe in $CDCl_3$ due to signal broadening [b]signal identified by HMBC correlation.

3-(4-Methylbenzenesulfonyl)-4-pyridinecarboxaldehyde (28a)

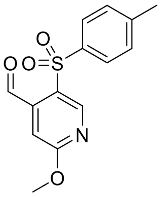

Potassium carbonate (55 mg, 0.4 mmol) and copper(II) fluoride (40 mg, 0.4 mmol) were added sequentially to a microwave vial which was then flame dried under argon until a blue colour just appeared (ca. 2–5 seconds). The microwave vial was allowed to cool to room temperature and copper(II) acetate (18 mg, 0.1 mmol), β-alanine (4.5 mg, 0.05 mmol), 4-methylbenzenesulfinic acid sodium salt (35.6 mg, 0.2 mmol), 2-methoxy-4-pyridinecarboxaldehyde (69 mg, 0.5 mmol) and HFIP (1 mL) were added to a microwave vial sequentially under argon. The vial was sealed, submerged in an oil bath preheated to 100 °C for 18 h [Stirring rate set to 500 rpm]. The reaction was allowed to cool to room temperature, diluted with EtOAc (5 mL) and the organic phase was washed with a saturated aqueous solution of sodium bicarbonate (10 mL), The product was extracted from the aqueous phase with EtOAc (2 × 10 mL) and the combined organic extracts were dried over Na_2SO_4, filtered and concentrated *in vacuo*. Purification by flash column chromatography (10–50% EtOAc:hexane) afforded sulfonyl aldehyde **28a** as a white solid (13.5 mg, 23%). m.p. = 145–148 °C. R_f 0.30 (20% EtOAc:hexane). IR (film)/cm^{-1} 3062, 2988, 2947, 2907, 2861, 1706 (C=O), 1591, 1542, 1473, 1369, 1323, 1284, 1161, 1142, 1094, 1019, 925, 814, 789, 575, 536. ^{1}H NMR (400 MHz, $CDCl_3$) δ 10.71 (s, 1H, CHO), 8.95 (s, 1H, Ar–CH), 7.80–7.77 (m, 2H, 2 × Ar–CH), 7.35 (dd, *J* = 8.5, 0.6 Hz, 2H, 2 × Ar–CH), 7.14 (d, *J* = 0.6 Hz, 1H, Ar–CH), 4.04 (s, 3H, OCH_3), 2.43 (s,

3H, CH_3). ^{13}C NMR (101 MHz, $CDCl_3$) δ 188.5 (CHO), 167.8 (Ar–C_q), 150.2 (Ar–CH), 145.0 (Ar–C_q), 142.7 (Ar–C_q), 138.6 (Ar–C_q), 130.3 (2 × Ar–CH), 129.5 (Ar–C_q), 127.4 (2 × Ar–CH), 110.3 (Ar–CH), 54.9 (OCH_3), 21.6 (CH_3). HRMS (FTMS+pAPCI) m/z calcd. For $C_{14}H_{14}NO_4S$ [M+H]: 292.0644; found: 292.0650.

Estrone Derived (8*R*,9*S*,13*S*,14*S*)-13-Methyl-17-oxo-7,8,9,11,12,13,14,15,16,17-decahydro-6H-cyclopenta[a]phenanthrene-3-carbaldehyde (S6)

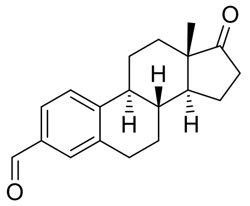

Estrone (541 mg, 2 mmol) was dissolved in CH_2Cl_2 (10 mL), cooled to 0 °C, then pyridine (324 μL, 4 mmol) and Tf_2O (405 μL, 2.4 mmol) were added sequentially under argon. The reaction was warmed to rt and stirred for 1.5 h. The reaction was quenched by the addition of water (10 mL) and the triflated intermediate was extracted from the aqueous phase with CH_2Cl_2 (3 × 10 mL), dried over Na_2SO_4, filtered, then concentrated *in vacuo*. In a separate flame dried microwave vial, palladium acetate (22.4 mg, 0.1 mmol), dppf (83 mg, 0.15 mmol) and *N*-formylsaccharin (633 mg, 3 mmol) were added sequentially followed by MeCN (10 mL). The vial was purged then backfilled with argon three times then estrone triflate, triethylsilane (479 μL, 3 mmol) and sodium carbonate (318 mg, 3 mmol) were added sequentially. The vial sealed then submerged in an oil bath preheated to 80 °C for 16 h. The reaction was then removed from the oil bath, allowed to cool to rt then pierced with a needle to release any internal pressure. The reaction was diluted with water (10 mL) and the product was extracted with CH_2Cl_2 (3 × 10 mL), dried over Na_2SO_4, filtered, then concentrated *in vacuo.* Purification by flash column chromatography (20% EtOAc:hexane) afforded aldehyde **S6** as a white solid (162 mg, 29%). $[\alpha]^{18}_D$ +130 (c 0.81, $CHCl_3$ (0.6% EtOH as stabliser)). m.p. = 190–191 °C. R_f 0.24 (20% EtOAc:hexane). IR (film)/cm^{-1} 2927, 2858, 2723, 1736 (C=O), 1693 (C=O), 1602, 1567, 1226, 1083, 1007, 822. ^{1}H NMR (400 MHz, $CDCl_3$) δ 9.95 (s, 1H, CHO), 7.66 (d, *J* = 8.1 Hz, 1H, Ar–CH), 7.62 (s, 1H, Ar–CH), 7.47 (d, *J* = 8.1 Hz, 1H, Ar–CH), 3.02–2.99 (m, 2H, C=OCH_2), 2.56–2.45 (m, 2H, 2 × CH), 2.37 (td, *J* = 10.8, 4.4 Hz, 1H, CH), 2.21–2.04 (m, 3H, 3 × CH), 2.00 (dt, *J* = 12.7, 2.8 Hz, 1H, CH), 1.67–1.49 (m, 6H, 6 × CH), 0.93 (s, 3H, CH_3). ^{13}C NMR (101 MHz, $CDCl_3$) δ 220.5 (C=O ketone), 192.3 (CHO), 147.0 (Ar–C_q), 137.5 (Ar–C_q), 134.2 (Ar–C_q), 130.2 (Ar–CH), 127.2 (Ar–CH), 126.0 (Ar–CH), 50.4 (C_q-CH), 47.8 (C_q), 44.8 (CH), 37.7(CH), 35.8 (CH_2), 31.5 (CH_2), 29.1 (CH_2), 26.1 (CH_2), 25.5 (CH_2), 21.5 (CH_2), 13.7 (CH_3). (^{1}H and ^{13}C) are consistent with previously reported literature.[11]

(8*R*,9*S*,13*S*,14*S*)-13-Methyl-17-oxo-2-(4-methylbenzenesulfonyl)-7,8,9,11,12,13,14,15,16,17-decahydro-6H-cyclopenta[a]phenanthrene-3-carbaldehyde (29a)

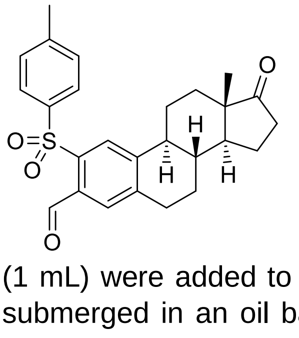

Potassium carbonate (55 mg, 0.4 mmol) and copper(II) fluoride (40 mg, 0.4 mmol) were added sequentially to a microwave vial which was then flame dried under argon until a blue colour just appears (ca. 2–5 seconds). The microwave vial was allowed to cool to room temperature and copper(II) acetate (18 mg, 0.1 mmol), β-alanine (4.5 mg, 0.05 mmol), 4-methylbenzenesulfinic acid sodium salt (44.5 mg, 0.25 mmol), aldehyde **S6** (79.9 mg, 0.201 mmol) and HFIP (1 mL) were added to a microwave vial sequentially under argon. The vial was sealed and submerged in an oil bath preheated to 100 °C for 18 h [Stirring rate set to 500 rpm]. The reaction was allowed to cool to room temperature, diluted with EtOAc (5 mL) and the organic phase was washed with a saturated aqueous solution of ammonium chloride (10 mL), The product was extracted from the aqueous phase with EtOAc (2 × 10 mL) and the combined

organic extracts were dried over Na_2SO_4, filtered and concentrated *in vacuo*. Purification by flash column chromatography (20–40% EtOAc:Hexane) afforded sulfonyl aldehyde **29a** as a white solid (31.2 mg, 36%[a]) $[\alpha]^{18}_{D}$ +72 (c 1.19, $CHCl_3$ (0.6% EtOH as stabliser)). m.p. = 140–141 °C. R_f 0.29 (40% EtOAc:hexane). IR (film)/cm^{-1} 2926, 2859, 2249, 1732 (C=O), 1686 (C=O), 1590, 1451, 1377, 1317, 1298, 1161, 1137, 1084, 1010, 910, 810, 727, 662, 614, 486. ^1H NMR (400 MHz, $CDCl_3$) δ 10.77 (s, 1H, CHO), 8.13 (s, 1H, Ar–CH), 7.77–7.74 (m, 3H, 3 × Ar–CH), 7.32 (dd, *J* = 8.3, 0.8 Hz, 2H, 2 × Ar–CH), 3.07–2.91 (m, 2H, $C{=}OCH_2$), 2.58–2.47 (m, 2H, 2 × CH), 2.41 (s, 3H, $ArCH_3$), 2.21–2.16 (m, 1H, CH), 2.12–2.04 (m, 3H, 3 × CH), 1.68–1.51[b] (m, 6H, 6 × CH), 0.94 (s, 3H, C_qCH_3). ^{13}C NMR (101 MHz, $CDCl_3$) δ 220.0 (C=O ketone), 189.6 (CHO), 146.6 (Ar–C_q), 144.6 (Ar–C_q), 143.4 (Ar–C_q), 139.7 (Ar–C_q), 139.1 (Ar–C_q), 131.1 (Ar–C_q), 130.2 (2 × Ar–CH), 130.1 (Ar–CH), 127.3 (2 × Ar–CH), 126.9 (Ar–CH), 50.3 (C_q-CH), 47.8 (C_q), 44.7 (CH), 37.4 (CH), 35.7 (CH_2), 31.3 (CH_2), 29.3 (CH_2), 25.7 (CH_2), 25.5 (CH_2), 21.6 (CH_2), 21.5 ($ArCH_3$), 13.8 (CH_3).HRMS (FTMS+pAPCI) m/z calcd. For $C_{26}H_{29}O_4S$ [M+H]: 437.1792; found: 437.1787. [a]91% purity, contains an inseparable impurity which we believe to be another regioisomer, where the position *alpha* to the carbonyl was sulfonylated, however due to the low intrinsic amount of the *alpha* functionalised regioisomer it was not possible to fully characterise this impurity. [b]signal overlapping with H_2O in $CDCl_3$

8-(4-Methylbenzenesulfonyl)phenanthrene-9-carbaldehyde (30a)

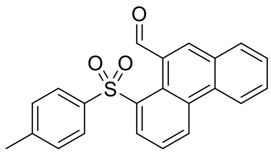

Synthesised according to general procedure **B** using phenanthrene-9-carbaldehyde (103 mg, 0.5 mmol) and 4-methylbenzenesulfinic acid sodium salt (35.6 mg, 0.2 mmol). Purification by flash column chromatography (20% EtOAc:pentane) afforded sulfonyl aldehyde **30a** as a white solid (46.4 mg, 64%). m.p. = 162–164 °C. R_f 0.04 (20% EtOAc:pentane). IR (film)/cm^{-1} 3056, 2948, 2919, 1851, 1690 (C=O), 1610, 1592, 1565, 1487, 1443, 1389, 1358, 1295, 1219, 1158, 1139, 1080, 1019, 910, 805, 759, 731, 664, 575, 528, 472. ^1H NMR (400 MHz, $CDCl_3$) δ 10.58 (s, 1H, CHO), 8.97 (d, *J* = 8.2 Hz, 1H, Ar–CH), 8.66 (d, *J* = 8.4 Hz, 1H, Ar–CH), 8.53 (s, 1H, Ar–CH), 8.12 (dd, *J* = 7.6, 1.2 Hz, 1H, Ar–CH), 8.08 (d, *J* = 7.9 Hz, 1H, Ar–CH), 7.85–7.71 (m, 5H, 5 × Ar–CH), 7.32 (d, *J* = 8.1 Hz, 2H, 2 ×Ar–CH), 2.43 (s, 3H, CH_3). ^{13}C NMR (101 MHz, $CDCl_3$) δ 189.3 (CHO), 144.5 (Ar–C_q), 138.8 (Ar–C_q), 138.1 (Ar–C_q), 133.5 (Ar–CH), 132.5 (Ar–C_q), 132.2 (Ar–C_q), 131.7 (Ar–CH), 131.5 (Ar–C_q), 130.8 (Ar–CH), 130.2 (Ar–C_q), 130.0 (Ar–CH), 129.9 (2 × Ar–CH), 128.4 (Ar–CH), 128.3 (Ar–CH), 127.6 (2 × Ar–CH), 125.9 (Ar–CH), 125.2 (Ar–C_q), 123.0 (Ar–CH), 21.6 (CH_3). HRMS (TOF–ESI$^+$) m/z calcd. For $C_{22}H_{17}O_3S$ [M+H]: 361.0898; found: 361.0896.

2-(4-Methylbenzenesulfonyl)-1-naphthaldehyde (31a *ortho*) and 8-(4-methylbenzenesulfonyl)-1-naphthaldehyde (31a *peri*)

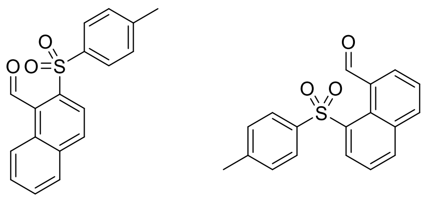

ortho *peri*

Synthesised according to general procedure **B** using 1-napthaldehyde (67.9 μL, 0.5 mmol) and 4-methylbenzenesulfinic acid sodium salt (35.6 mg, 0.2 mmol). Purification by flash column chromatography (10–20% EtOAc:pentane) afforded sulfonyl aldehyde **31a** ***ortho*** as a

white solid (14.2 mg, 23%*) followed by sulfonyl aldehyde **31a** ***peri*** as a white solid (29.5 mg, 48%)

31a ***ortho*****:** m.p. = 130–135 °C. R_f 0.36 (20% EtOAc:pentane). IR (film)/cm^{-1} 3058, 2919, 1699 (C=O), 1593, 1500, 1450, 1426, 1377, 1315, 1175, 1151, 1128, 1082, 1051, 892, 815, 706, 624, 683, 603, 542 493. ^{1}H NMR (400 MHz, $CDCl_3$) δ 11.16 (s, 1H, CHO), 8.27–8.24 (m, 1H, Ar–CH), 8.09 (d, *J* = 8.8 Hz, 1H, Ar–CH), 8.03 (d, *J* = 8.8 Hz, 1H, Ar–CH), 7.94–7.91 (m, 1H, Ar–CH), 7.83 (d, *J* = 8.4 Hz, 2H, 2 × Ar–CH), 7.70–7.63 (m, 2H, 2 × Ar–CH), 7.33 (d, *J* = 8.4 Hz, 2H, 2 × Ar–CH), 2.40 (s, 3H, CH_3). ^{13}C NMR (101 MHz, $CDCl_3$) δ 194.4 (CHO), 144.9 (Ar–C_q), 139.0 (Ar–C_q), 138.5 (Ar–C_q), 135.5 (Ar–C_q), 135.2 (Ar–C_q), 132.3 (Ar–CH), 130.1 (2 × Ar–CH), 129.3 (Ar–CH), 129.2 (Ar–C_q), 129.1 (Ar–CH), 128.5 (Ar–CH), 127.7 (2 × Ar–CH), 126.4 (Ar–CH), 123.5 (Ar–CH), 21.6 (CH_3). HRMS (TOF–ESI$^+$) m/z calcd. For $C_{20}H_{17}NO_3NaS$ [M+Na+MeCN]: 374.0827; found: 374.0833. *87% purity.

31a ***peri***: m.p. = 179–182 °C. R_f 0.12 (20% EtOAc:pentane). IR (film)/cm^{-1} 3062, 2922, 2878, 1690 (C=O), 1595, 1562, 1500, 1299, 1239, 1198, 1154, 1139, 1074, 803, 759, 714, 660. ^{1}H NMR (400 MHz, $CDCl_3$) δ 10.70 (s, 1H, CHO), 8.22 (dd, *J* = 7.2, 1.6 Hz, 1H, Ar–CH), 8.15 (d, *J* = 8.1 Hz, 1H, Ar–CH), 8.14–8.10 (m, 2H, 2 × Ar–CH), 7.81–7.79 (m, 2H, 2 × Ar–CH), 7.70 (dd, *J* = 7.6, 7.6 Hz, 1H, Ar–CH), 7.58 (dd, *J* = 7.8, 7.8 Hz, 1H, Ar–CH), 7.34 (d, *J* = 8.4 Hz, 2H, 2 × Ar–CH), 2.44 (s, 3H, CH_3). ^{13}C NMR (101 MHz, $CDCl_3$) δ 190.4 (CHO), 144.7 (Ar–C_q), 138.1 (Ar–C_q), 137.1 (Ar–C_q), 135.3 (Ar–CH), 134.8 (Ar–C_q), 134.4 (Ar–C_q), 134.1 (Ar–CH), 132.8 (Ar–CH), 131.1 (Ar–CH), 130.0 (2 × Ar–CH), 127.6 (2 × Ar–CH), 126.6 (Ar–CH), 126.4 (Ar–C_q), 124.8 (Ar–CH), 21.6 (CH_3). HRMS (FTMS+pAPCI) m/z calcd. For $C_{18}H_{15}O_3S$ [M+H]: 311.0736; found: 311.0735.

2-Methyl-8-(4-methylbenzenesulfonyl)-1-naphthaldehyde (32a)

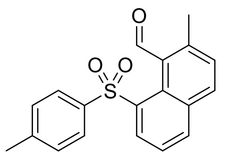

Synthesised according to general procedure **B** using 2-methyl-1-napthaldehyde (85 mg, 0.5 mmol) and 4-methylbenzenesulfinic acid sodium salt (35.6 mg, 0.2 mmol). Purification by flash column chromatography (20% EtOAc:pentane) afforded sulfonyl aldehyde **32a** as a white solid (8.6 mg, 13%). m.p. = 138–140 °C. R_f 0.16 (20% EtOAc:pentane). IR (film)/cm^{-1} 3056, 2922, 2363, 1700 (C=O), 1595, 1550, 1446, 1305, 1215, 1140, 1073, 924, 812, 731, 663, 589. ^{1}H NMR (400 MHz, $CDCl_3$) δ 10.63 (s, 1H, CHO), 8.24 (d, *J* = 7.9 Hz, 1H, Ar–CH), 8.07 (d, *J* = 7.9 Hz, 1H, Ar–CH), 7.92 (d, *J* = 8.3 Hz, 1H, Ar–CH), 7.77 (d, *J* = 8.3 Hz, 2H, 2 × Ar–CH), 7.55 (dd, *J* = 7.8, 7.8 Hz, 1H, Ar–CH), 7.45 (d, *J* = 8.3 Hz, 1H, Ar–CH), 7.32 (d, *J* = 8.3 Hz, 2H, 2 × Ar–CH), 2.73 (s, 3H, CH_3), 2.43 (s, 3H, CH_3). ^{13}C NMR (101 MHz, $CDCl_3$) δ 192.1 (CHO), 144.1 (Ar–C_q), 140.5 (Ar–C_q), 139.3 (Ar–C_q), 136.7 (Ar–C_q), 135.0 (Ar–CH), 133.8 (Ar–CH), 133.5 (Ar–C_q), 132.2 (Ar–CH), 131.8 (Ar–C_q), 131.1 (Ar–CH), 129.7 (2 × Ar–CH), 127.3 (Ar–C_q), 127.1 (2 × Ar–CH), 124.3 (Ar–CH), 21.6 (CH_3), 21.1 (CH_3). HRMS (TOF–ESI$^+$) m/z calcd. For $C_{19}H_{17}O_3S$ [M+H]: 325.0898; found: 325.0910.

Derivatisation

(2-Methyl-6-(4-methylbenzenesulfonyl)phenyl)methanol (33)

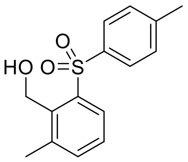

Lithium borohydride (22.8 mg, 1 mmol) was added to a stirring solution of sulfonyl aldehyde **3a** (54.5 mg, 0.2 mmol) in THF (0.34 mL) which was sealed and heated to 65 °C for 15 min. The reaction was quenched by the addition of MeOH, allowed to cool to rt then concentrated *in vacuo*. Water (10 mL) was added, and the product was extracted with CH_2Cl_2 (3 × 10 mL). The combined organic phases were dried over Na_2SO_4, filtered, then concentrated *in vacuo* to afford sulfonyl alcohol **33** as a colourless oil (39.5 mg, 72%). IR (film)/cm^{-1} 3532 (O–H), 2962, 2919, 1595, 1455, 1287, 1183, 1157, 1126, 1081, 1014, 814, 677, 582. ^{1}H NMR (400 MHz, $CDCl_3$) δ 7.96 (dd, *J* = 7.9, 1.6 Hz, 1H, Ar–CH), 7.79 (d, *J* = 8.2 Hz, 2H, 2 × Ar–CH), 7.47 (dd, *J* = 7.8, 1.4 Hz, 1H, Ar–CH), 7.39 (dd, *J* = 7.8,7.8 Hz, 1H, Ar–CH), 7.33 (d, *J* = 8.2 Hz, 2H, 2 × Ar–CH), 4.71 (d, *J* = 7.6 Hz, 2H, CH_2), 3.02 (t, *J* = 7.6 Hz, 1H, OH), 2.47 (s, 3H, CH_3), 2.43 (s, 3H, CH_3). ^{13}C NMR (101 MHz, $CDCl_3$) δ 144.4 (Ar–C_q), 140.5 (Ar–C_q), 140.4 (Ar–C_q), 138.3 (Ar–C_q), 137.6 (Ar–C_q), 136.3 (Ar–CH), 129.9 (2 × Ar–CH), 128.1 (Ar–CH), 127.5 (Ar–CH), 127.4 (2 × Ar–CH), 57.8 (CH_2), 21.6 (CH_3), 19.3 (CH_3). HRMS (TOF–ESI$^+$) m/z calcd. For $C_{15}H_{17}O_3S$ [M+H]: 277.0898; found: 277.0907.

4-(2-Methyl-6-(4-methylbenzenesulfonyl)benzyl)morpholine (34)

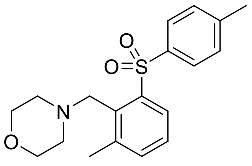

Sodium triacetoxyborohydride (51.0 mg, 0.24 mmol) was added to a solution of sulfonyl aldehyde **3a** (55.1 mg, 0.2 mmol) and morpholine (21.0 μL, 0.24 mmol) in CH_2Cl_2 (0.67 mL) at rt and was stirred overnight at rt. The reaction was quenched by the addition of 1 M aqueous NaOH (5 mL) and the product extracted from the aqueous phase with CH_2Cl_2 (3 × 10 mL). The combined organic extracts were dried over Na_2SO_4, filtered and concentrated *in vacuo*. The residue was acidified by the addition of 1 M aqueous HCl (7 mL), and washed with Et_2O. The organic phase was discarded, and the aqueous phase was basified by the addition of 1 M aqueous NaOH (14 mL) and the product extracted with CH_2Cl_2 (3 × 20 mL). The combined organic extracts were dried over Na_2SO_4, filtered, then concentrated *in vacuo* to afford sulfonyl amine **34** as an amorphous white solid (31.7 mg, 46%). IR (film)/cm^{-1} 2924, 2853, 2810, 1453, 1306, 1158, 1137, 1115, 1084, 10005, 664, 612, 680, 579. ^{1}H NMR (400 MHz, $CDCl_3$) δ 8.07 (d, *J* = 7.8 Hz, 1H, Ar–CH), 7.75 (d, *J* = 8.2 Hz, 2H, 2 × Ar–CH), 7.45–7.35 (m, 2H, 2 × Ar–CH), 7.31 (d, *J* = 8.2 Hz, 2H, 2 × Ar–CH), 3.87 (s, 2H, $ArCH_2$), 3.56 – 3.30 (brs, 4H, 2 × C*H*HC*H*H), 2.49 (s, 3H, CH_3), 2.43 (s, 3H, CH_3), 2.29 (brs, 4H, 2 × CH*H*CH*H*). ^{13}C NMR (101 MHz, $CDCl_3$) δ 143.6 (Ar–C_q), 141.8 (Ar–C_q), 140.4 (Ar–C_q), 140.2 (Ar–C_q), 136.0 (Ar–C_q + Ar–CH), 129.5 (2 × Ar–CH), 128.0 (Ar–CH), 127.2 (3 × Ar–CH), 66.9 (OCH_2), 54.9 ($ArCH_2$), 52.6 (NCH_2), 21.5 (CH_3), 20.7 (CH_3). HRMS (TOF–ESI$^+$) m/z calcd. For $C_{19}H_{24}NO_3S$ [M+H]: 346.1477; found: 346.1473.

(2-Methyl-6-(4-methylbenzenesulfonyl)phenyl)methanol (35)

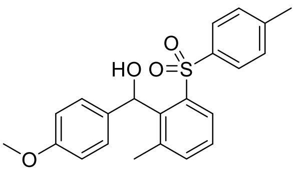

4-Methoxyphenylmagnesium bromide (0.23 mL, 0.5 M) was added to a stirring solution of sulfonyl aldehyde **3a** (27.6 mg, 0.1 mmol) in THF (0.2 mL) at 0°C then the reaction was allowed to warm to rt overnight. The reaction was quenched by the addition of saturated aqueous ammonium chloride (1 mL), diluted with water (10 mL) and the product extracted with CH_2Cl_2 (3 × 10 mL). The combined organic extracts were dried over Na_2SO_4, filtered then concentrated *in vacuo* to afford sulfonyl alcohol **35** as a pink solid (34.9 mg, 91%). m.p. = 149–150 °C. IR (film)/cm^{-1} 3493 (O–H), 2997, 2956, 2836, 1609, 1511, 1457,

1297, 1246, 1156, 1130, 1035, 829, 814, 683, 658, 574, 455. ^{1}H NMR (400 MHz, $CDCl_3$) δ 8.12 (dd, *J* = 7.3, 2.3 Hz, 1H, Ar–CH), 7.65 (d,*J* = 8.3 Hz, 2H, 2 × Ar–CH), 7.45–7.34 (m, 2H, 2 × Ar–CH), 7.21 (d, *J* = 8.3 Hz, 2H, 2 × Ar–CH), 6.86 (d, *J* = 8.8 Hz, 2H, 2 × Ar–CH), 6.80 (d, *J* = 4.6 Hz, 1H, ArC*H*OH), 6.68 (d, *J* = 8.8 Hz, 2H, 2 × Ar–CH), 3.76 (s, 3H, OCH_3), 2.88 (s, 1H, OH), 2.36 (s, 3H, CH_3), 2.07 (s, 3H, CH_3). ^{13}C NMR (101 MHz, $CDCl_3$) δ 158.2 (Ar–C_q), 144.0 (Ar–C_q), 141.5 (Ar–C_q), 140.5 (Ar–C_q), 140.4 (Ar–C_q), 138.8 (Ar–C_q), 137.7 (Ar–CH), 133.5 (Ar–C_q), 129.8 (2 × Ar–CH), 127.8 (Ar–CH), 127.3 (2 × Ar–CH), 127.0 (Ar–CH), 126.5 (2 × Ar–CH), 113.2 (2 × Ar–CH), 69.4 (CH), 55.1 (OCH_3), 21.5 (CH_3), 20.8 (CH_3). HRMS (TOF–ESI$^+$) m/z calcd. For $C_{22}H_{22}O_4SNa$ [M+Na]: 405.1137; found: 405.1127.

1-(2-Methyl-6-(4-methylbenzenesulfonyl)phenyl)-2,3,4,9-tetrahydro-1H-pyrido[3,4-b]indole (36)

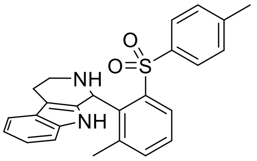

Sulfonyl aldehyde **3a** (55.0 mg, 0.2 mmol) and tryptamine (32.0 mg, 0.2 mmol) were added to a microwave vial. The vial was sealed then HFIP (0.32 mL) was added and the reaction was submerged in a preheated oil bath at 60 °C for 48 h. The reaction was concentrated *in vacuo* which afforded cyclised product **36** as an amorphous brown solid (60.4 mg, 73%). IR (film)/cm^{-1} 3401 (N–H), 3055, 2919, 2846, 1649, 1594, 1454, 1292, 1182, 1132, 1082, 908, 812, 730, 659, 582, 562, 499. ^{1}H NMR (400 MHz, $CDCl_3$) δ 8.84 (s, 1H, NH), 8.07 (s, 1H, NH), 8.02 (dd, *J* = 7.2, 2.2 Hz, 1H, Ar–CH), 7.68–7.61 (m, 3H, 3 × Ar–CH), 7.47–7.34 (m, 3H, 3 × Ar–CH), 7.25–7.13 (m, 4H, 4 × Ar–CH), 7.09–7.03 (m, 1H, ArC*H*NH), 3.97 (td, *J* = 7.7, 1.5 Hz, 2H, CH_2), 3.14 (t, *J* = 7.7 Hz, 2H,CH_2), 2.35 (s, 3H, CH_3), 2.23 (s, 3H, CH_3). ^{13}C NMR (101 MHz, $CDCl_3$) δ 160.2 (Ar–C_q), 144.1 (Ar–C_q), 139.9 (Ar–C_q), 138.9 (Ar–C_q), 138.8 (Ar–C_q), 136.3 (Ar–C_q), 136.0 (Ar–CH), 135.1 (Ar–C_q), 129.6 (2 × Ar–CH), 128.7 (Ar–CH), 127.6 (2 × Ar–CH), 127.4 (Ar–C_q), 126.8 (Ar–CH), 122.0 (Ar–CH), 121.8 (CH), 119.3 (Ar–CH), 118.8 (Ar–CH), 113.7 (Ar–C_q), 111.2 (Ar–CH), 62.3 (CH_2), 26.5 (CH_2), 21.5 (CH_3), 20.3 (CH_3). HRMS (TOF–ESI$^+$) m/z calcd. For $C_{25}H_{25}N_2O_2S$ [M+H]: 417.1637; found: 417.1635.

2-Methyl-6-(4-methylbenzenesulfonyl)benzoic acid (37)

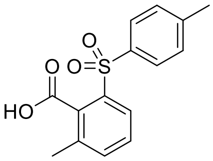

Hydrogen peroxide (30% in H_2O, 41 μL, 0.4 mmol) was added to a solution of sodium phosphate monobasic (105 mg, 0.88 mmol) and sulfonyl aldehyde **3a** in acetonitrile (1.6 mL). The reaction was cooled to 0 °C then a solution of sodium chlorite (72 mg, 0.8 mmol) in H_2O (0.64 mL) was added and the reaction was allowed to warm to rt. [NOTE: on addition of sodium chlorite, the reaction turned yellow and decolourised as the reaction progressed.] After 3 h, the reaction was quenched by the addition of sodium thiosulfate, acidified by the addition of 1 M aqueous HCl (5 mL) and the product was extracted with ethyl acetate (3 × 10 mL) The combined organic phases were dried over Na_2SO_4, filtered, then concentrated *in vacuo* to afford sulfonyl acid **37** as a white solid (55.5 mg, 96%). m.p. = 173–176 °C. IR (film)/cm^{-1} 3200 (O–H), 2924, 1738, 1712 (C=O), 1595, 1450, 1320, 1290, 1188, 1143, 1081, 876, 814, 708, 690, 657, 586. ^{1}H NMR (400 MHz, MeOD) δ 7.88–7.84 (m, 3H, 3 × Ar–CH), 7.50–7.43 (m, 2H, 2 × Ar–CH), 7.31 (d, *J* = 8.3 Hz, 2H, 2 × Ar–CH), 2.35 (s, 6H, 2 × CH_3). ^{13}C NMR (101 MHz, MeOD) δ 171.0 (C=O acid), 146.0 (Ar–C_q), 140.0 (Ar–C_q), 139.2 (Ar–C_q), 137.2 (Ar–C_q), 136.4 (Ar–CH), 135.7 (Ar–C_q), 130.7 (2 × Ar–CH), 130.6 (Ar–CH), 129.1 (2 × Ar–CH), 128.1 (Ar–CH), 21.5 (CH_3), 19.2 (CH_3). HRMS (TOF–ESI$^+$) m/z calcd. For $C_{15}H_{15}O_4S$ [M+H]: 291.0691; found: 291.0701.

2-(2-Methyl-6-(4-methylbenzenesulfonyl)phenyl)-1H-benzo[d]imidazole (38)

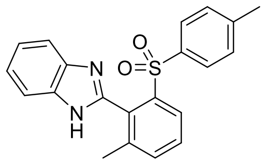

Sulfonyl aldehyde **3a** (55.1 mg, 0.2 mmol), *o*-phenylenediamine (21.6 mg, 0.2 mmol) and ceric ammonium nitrate (11 mg, 0.02 mmol) were added sequencially to microwave vial. The vial was sealed and acetonitrile (2 mL) and hydrogen peroxide (30 % in water, 0.2 mL) were added then the vial was submerged in an oil bath preheated to 50 °C overnight. The reaction was diluted with water (5 mL) and the product was extracted with CH_2Cl_2 (3 × 10 mL) The combined organic phases were dried over Na_2SO_4, filtered, then concentrated *in vacuo*. Purification by flash column chromatography (20% acetone:pentane) afforded sulfonyl benzimidazole **38** as a orange solid (48.3 mg, 66%). m.p. = 272–273 °C. IR (film)/cm^{-1} 3295 (N–H), 3103, 2363, 1668, 1639, 1594, 1443, 1321, 1205, 1162, 1141, 1083, 829, 531, 494m 457. ^{1}H NMR (400 MHz, $CDCl_3$) δ 9.83 (s, 1H, N–H), 8.26 (d, *J* = 7.5 Hz, 1H, Ar–CH), 7.70–7.49 (m, 4H, 4 × Ar–CH), 7.38–7.23 (m, 2H, 2 × Ar–CH), 7.15 (d, *J* = 8.2 Hz, 2H, 2 × Ar–CH), 6.84 (d, *J* = 8.1 Hz, 2H, 2 × Ar–CH), 2.24 (s, 3H, CH_3), 2.17 (s, 3H, CH_3). ^{13}C NMR (101 MHz, $CDCl_3$) δ 146.4 (Ar–C_q), 144.1 (Ar–C_q), 143.2 (Ar–C_q), 141.9 (Ar–C_q), 141.7 (Ar–C_q), 136.2 (Ar–C_q), 135.4 (Ar–CH), 133.3 (Ar–C_q), 129.7 (Ar–CH), 129.6 (Ar–C_q), 129.2 (2 × Ar–CH), 127.2 (2 × Ar–CH), 126.1 (Ar–CH), 123.5 (Ar–CH), 122.3 (Ar–CH), 119.5 (Ar–CH), 111.3 (Ar–CH), 21.5 (CH_3), 20.1 (CH_3). HRMS (TOF–ESI$^+$) m/z calcd. For $C_{21}H_{19}N_2O_2S$ [M+H]: 363.1167; found: 363.1155.

Other Starting Material and Product

Benzaldehyde-2,3,4,5,6-d$_5$ (13-d$_5$)

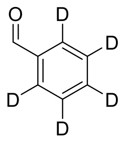

Dess martin periodinane (937 mg, 2.21 mmol) was added to a stirring solution of Benzyl-2,3,4,5,6-d$_5$ alcohol (228.5 μL, 2.21 mmol) in CH_2Cl_2 (44 mL) at 0 °C which was allowed to warm to rt and stirred for 4 h. The reaction was quenched by the addition of saturated aqueous $NaHCO_3$ (40 mL) and the product extracted with CH_2Cl_2 (2 × 40 mL). The combined organic phases were dried over Na_2SO_4, filtered then concentrated *in vacuo*. Purification by flash column chromatography (20% Et_2O:pentane) afforded deuterobenzaldehyde 13-d$_5$ as a colourless oil (157.2 mg, 64%). CAS no.: 14132-51-5. R_f 0.6 (20% Et_2O:pentane). ^{1}H NMR (400 MHz, $CDCl_3$) δ 10.0 (s, 1H). analytical data (^{1}H NMR) is in agreement with the reported literature.[12]

2-((1,1,1,3,3,3-Hexafluoropropan-2-yl)oxy)-6-methylbenzaldehyde (4)

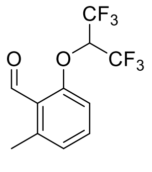

Potassium carbonate (138.2 mg, 1 mmol) and copper(II) fluoride (101.5 mg, 1 mmol) were added sequentially to a microwave vial which was then flame dried under argon until a blue colour just appeared (ca. 2–5 seconds). The microwave vial was allowed to cool to room temperature and copper(II) acetate (45 mg, 0.25 mmol), β-alanine (11.1 mg, 0.125 mmol), 2-methylbenzaldehyde (57.8 μL, 0.5 mmol) and HFIP (2.5 mL) were added to a microwave vial sequentially under argon. The vial was sealed and submerged in an oil bath preheated to 100 °C for 18 h [Stirring rate set to 500 rpm]. The reaction was allowed to cool to room temperature, diluted with EtOAc (5 mL) and the organic phase was washed with a saturated aqueous solution of ammonium chloride (10 mL), The product was extracted from the aqueous phase with EtOAc (2 × 10 mL) and the combined organic extracts were dried over Na_2SO_4, filtered and concentrated *in vacuo*. Purification by flash column chromatography (2.5% Et_2O:pentane) then concentration *in vacuo* [note: rotary evaporator bath set to 40 °C] to remove any remaining starting material afforded HFIP adduct **4** as a clear and colourless crystalline solid (26.7 mg, 19%[a]). m.p. = 52–53 °C. R_f 0.29 (2.5% EtOAc:hexane). IR (film)/cm^{-1} 2967, 2892, 1692 (C=O), 1573, 1469, 1372, 1252, 1200, 1111, 902, 828, 738. ^{1}H NMR (400 MHz, $CDCl_3$) δ 10.63 (s, 1H, CHO), 7.47 (dd, *J* = 8.3, 8.3 Hz, 1H, Ar–CH), 7.07–7.05 (m, 1H, Ar–CH), 6.95 (d, *J* = 8.3 Hz,

1H, Ar–CH), 5.02 (hept, *J* = 5.7 Hz, 1H, $CH(CF_3)_2$), 2.62 (s, 3H, CH_3). ^{13}C NMR (101 MHz, $CDCl_3$) δ 190.7 (CHO), 160.5 (Ar–C_q), 143.0 (Ar–C_q), 134.4 (Ar–CH), 128.0 (Ar–CH), 125.1 (Ar–C_q), 123.2–115.4[b] (m), 112.0 (Ar–CH), 76.6–75.3[b] (m, *C*H$(CF_3)_2$), 21.4 (CH_3). ^{19}F NMR (377 MHz, $CDCl_3$) δ –73.25 (d, *J* = 6.9 Hz, $CH(CF_3)_2$). HRMS (EI$^+$) m/z calcd. For $C_{11}H_8F_6O_2$ [M+H]: 286.0423; found: 286.0431. [a]93% purity, contains EtOAc. [Note: This material readily sublimes under reduced pressure at rt, so it was not possible to fully remove the EtOAc without evaporation of the product. Attempts to remove trace solvent under vacuum led to complete loss of product.] [b]Signal partly obscured. Analytical data (^{1}H, ^{13}C, ^{19}F NMR are in agreement with the reported literature.[13]

Procedure for Multi Gram Scale Synthesis of Sulfonyl Aldehyde 3a

Potassium carbonate (2.75 g, 20 mmol) and copper(II) fluoride (2.00 g, 20 mmol) were added sequentially to a schlenk tube which was then flame dried under argon until a blue colour just appeared (ca. 5–10 seconds). The Schlenk tube was allowed to cool to room temperature and copper(II) acetate (900 mg, 5 mmol), β-alanine (225 mg, 2.5 mmol), *p*-toluene sulfinic acid sodium salt (1.78 g, 10 mmol) and 2-methylbenzaldehyde (2.90 mL, 25 mmol) were added sequentially under argon, HFIP (50 mL, 0.2 M) was added then the tube sealed with a young's tap and was submerged in a preheated oil bath to 100 °C for 24 h [Stirring rate set to 500 rpm]. The reaction was allowed to cool to room temperature, diluted with EtOAc (50 mL) and the organic phase was washed with a saturated aqueous solution of ammonium chloride (150 mL) and brine (50 mL), [Note: The crude should be shaken until a change from orange/brown to blue/green is observed. Should the resulting solution emulsify brine can be added. Occasionally a brown precipitate can remain which obscures the phase boundary, this is collected with the aqueous phase for the first two extractions then with the organic phase on the final extraction]. The product was extracted from the aqueous phase with EtOAc (2 × 50 mL) and the combined organic extracts were dried over Na_2SO_4, filtered and concentrated *in vacuo*. Purification by flash column chromatography (10–20% EtOAc:hexane) afforded sulfonyl aldehyde **3a** as an off white solid (2.07 g, 75%).

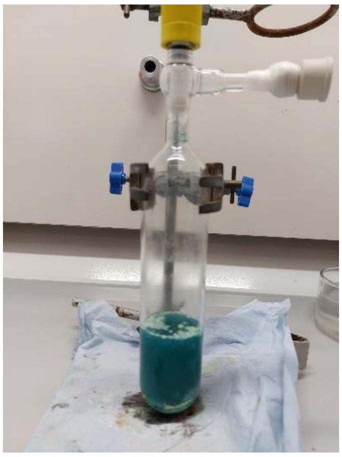

Reaction prior to heating

Blue to orange colour change observed. Colour change begins after approx. 30 min

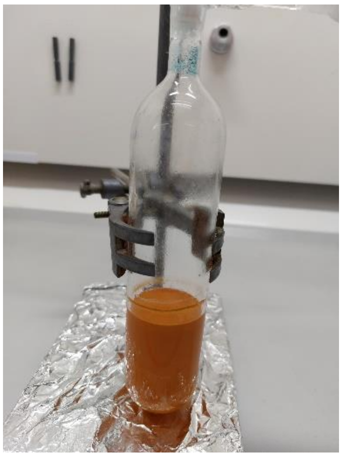

Reaction after 100 °C, 24 h

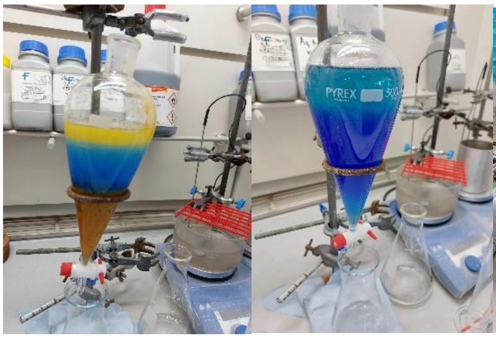

Aqueous sat NH_4Cl workup.
Brown/orange to blue colour change
Left: before shaking. Right: after shaking

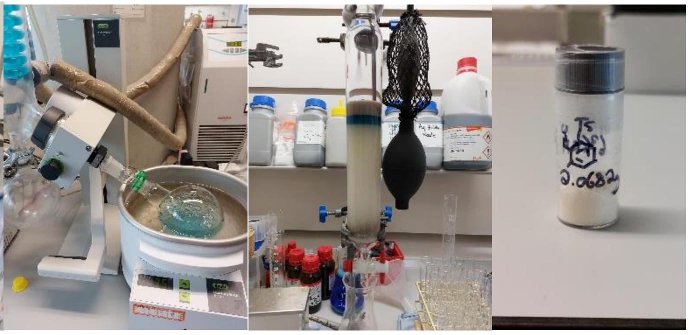

Concentration *in vacuo* then flash column chromatography affords **3a** as an off white powder

Unsuccessful Substrates

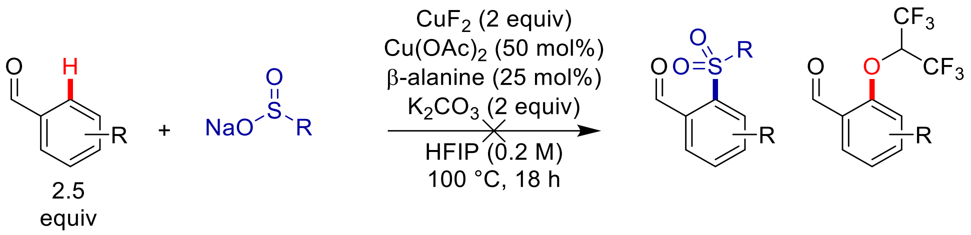

Aldehydes (using 4-methylbenzenesulfinic acid sodium salt)

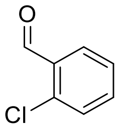

Complex Mixture
(Evidence of products of S_NAr)

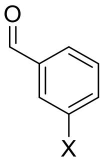

X = CN or SF_5

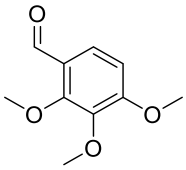

<10% yield

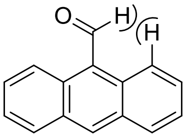

Unable to adopt reactive
confomer due to steric clash

Sulfinate salts (using 2-methylbenzaldehyde)

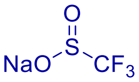

0%, gave exclusive formation
of HFIP adduct
insufficient nucleophillicity

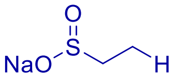

<10%,

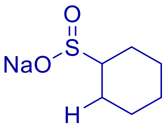

<5%

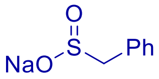

NR

Sulfinates possesing β-hydrogens
were not tolerated in the reaction

^{1}H and ^{13}C Spectra of Selected Compounds

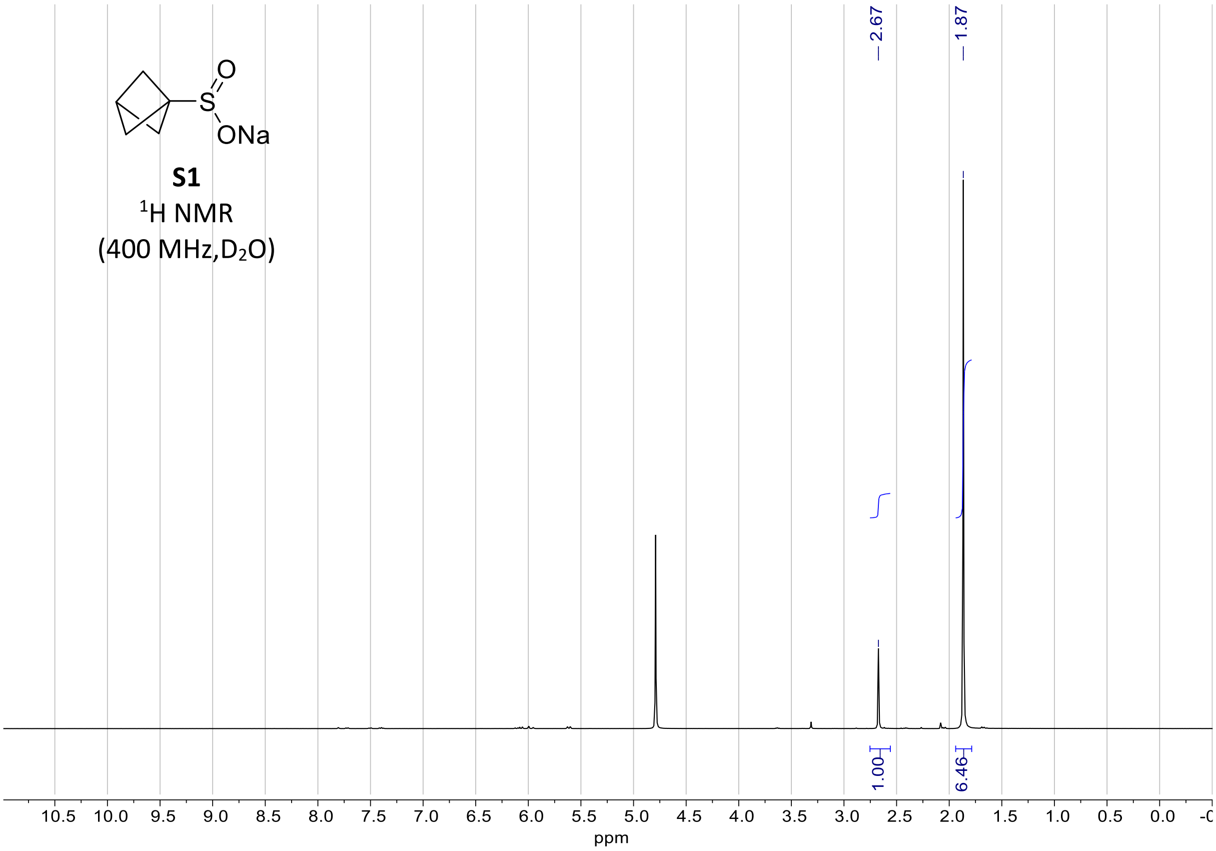
O
S
ONa
S1
1H NMR
(400 MHz,D2O)
2.67
1.87
1.00
6.46
10.5 10.0 9.5 9.0 8.5 8.0 7.5 7.0 6.5 6.0 5.5 5.0 4.5 4.0 3.5 3.0 2.5 2.0 1.5 1.0 0.5 0.0
ppm

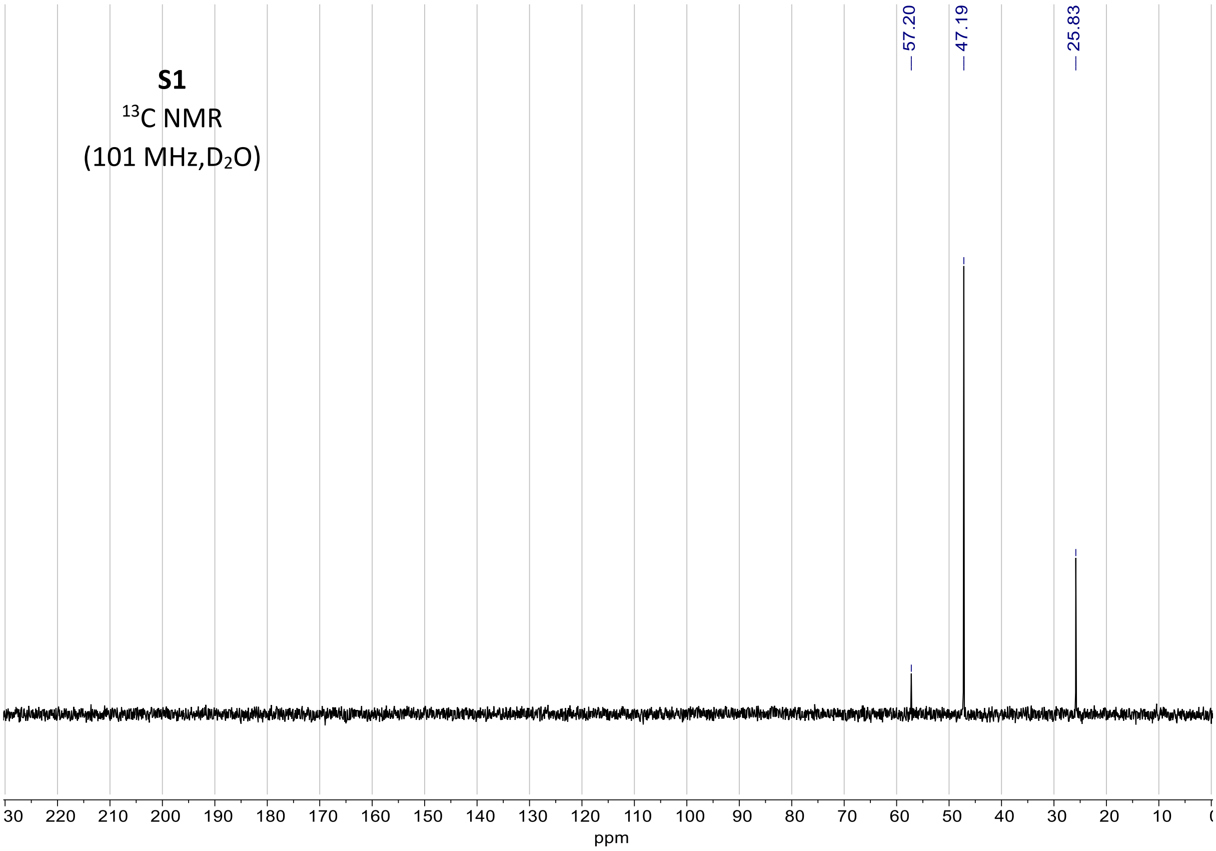
S1
13C NMR
(101 MHz,D2O)
57.20
47.19
25.83
220 210 200 190 180 170 160 150 140 130 120 110 100 90 80 70 60 50 40 30 20 10
ppm

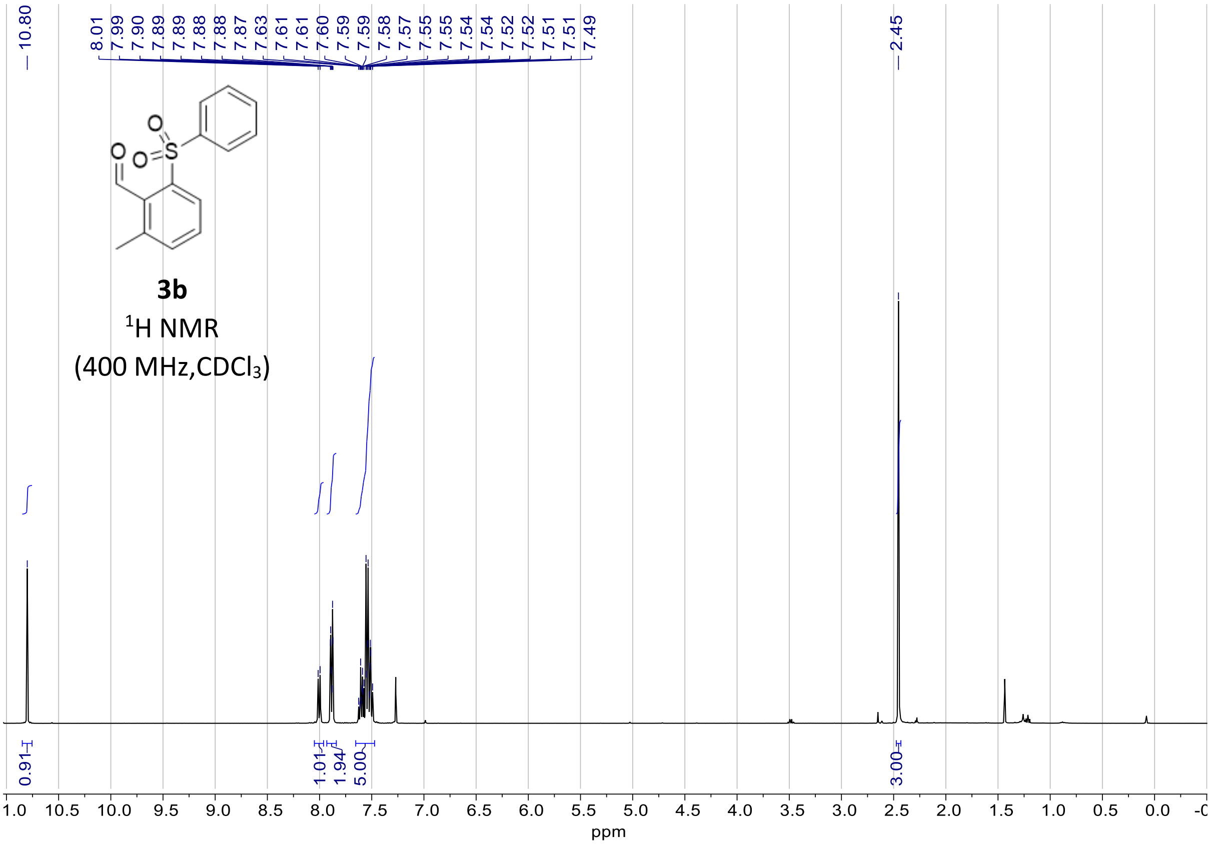
3b
^{1}H NMR
(400 MHz,$CDCl_3$)
ppm

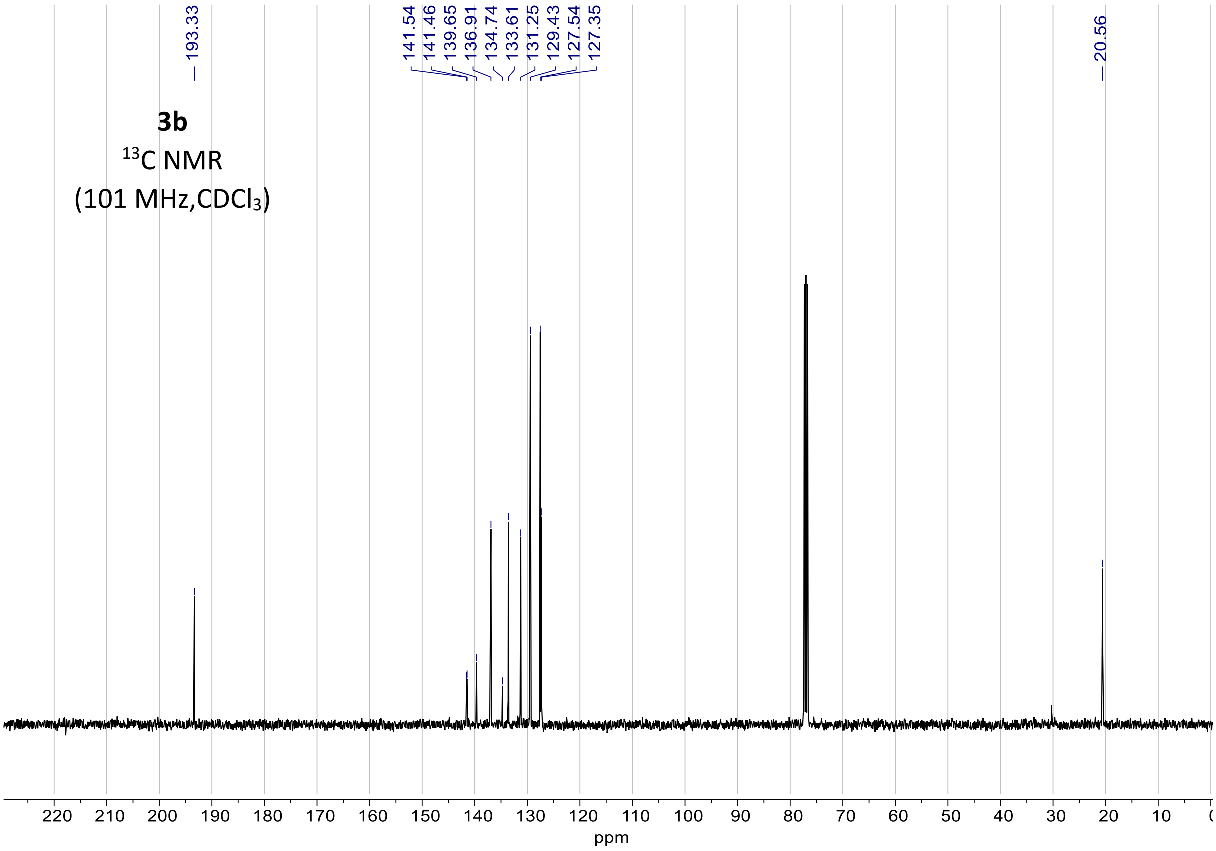
3b
^{13}C NMR
(101 MHz,$CDCl_3$)
ppm

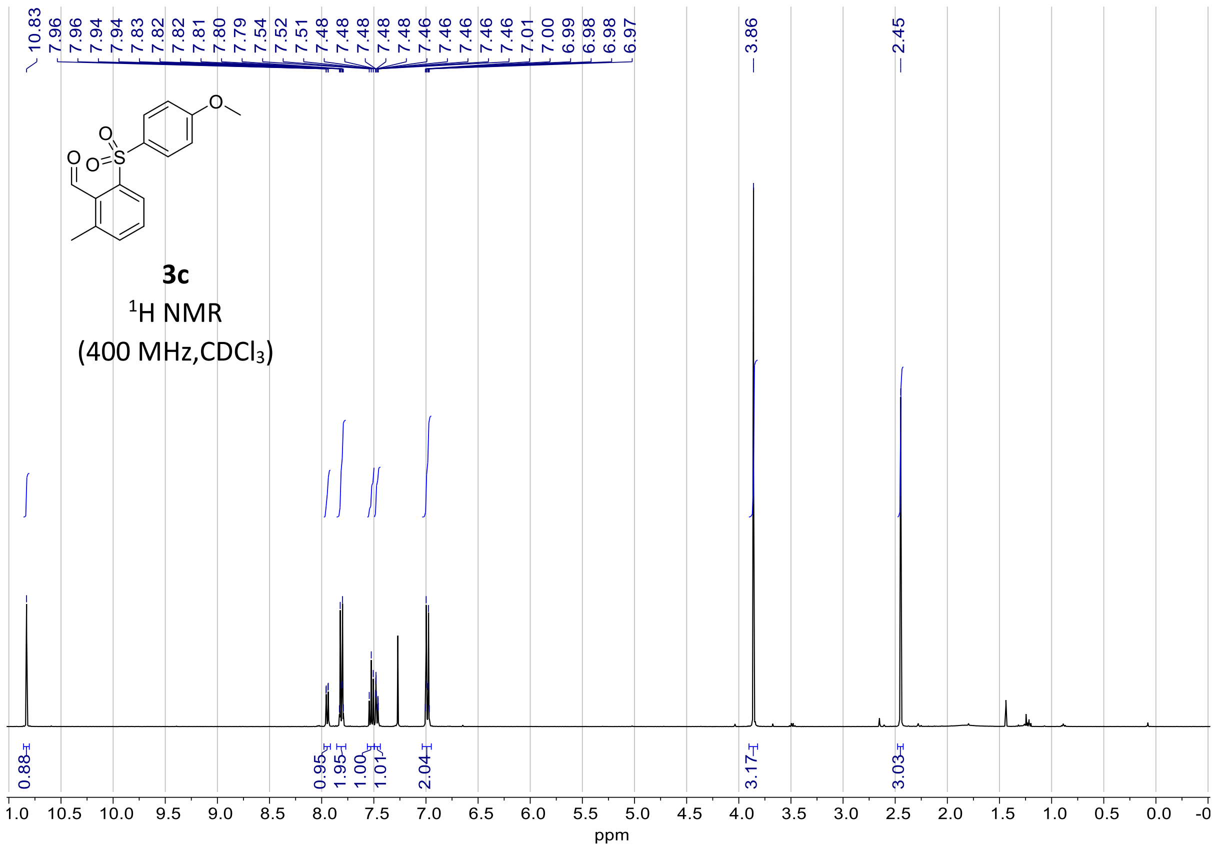
10.83
7.96
7.94
7.94
7.83
7.82
7.82
7.81
7.80
7.79
7.54
7.52
7.51
7.48
7.48
7.48
7.48
7.48
7.46
7.46
7.46
7.46
7.01
7.00
6.99
6.98
6.98
6.97
3.86
2.45
3c
^{1}H NMR
(400 MHz,CDCl$_3$)
0.88
0.95
1.95
1.00
1.01
2.04
3.17
3.03
ppm

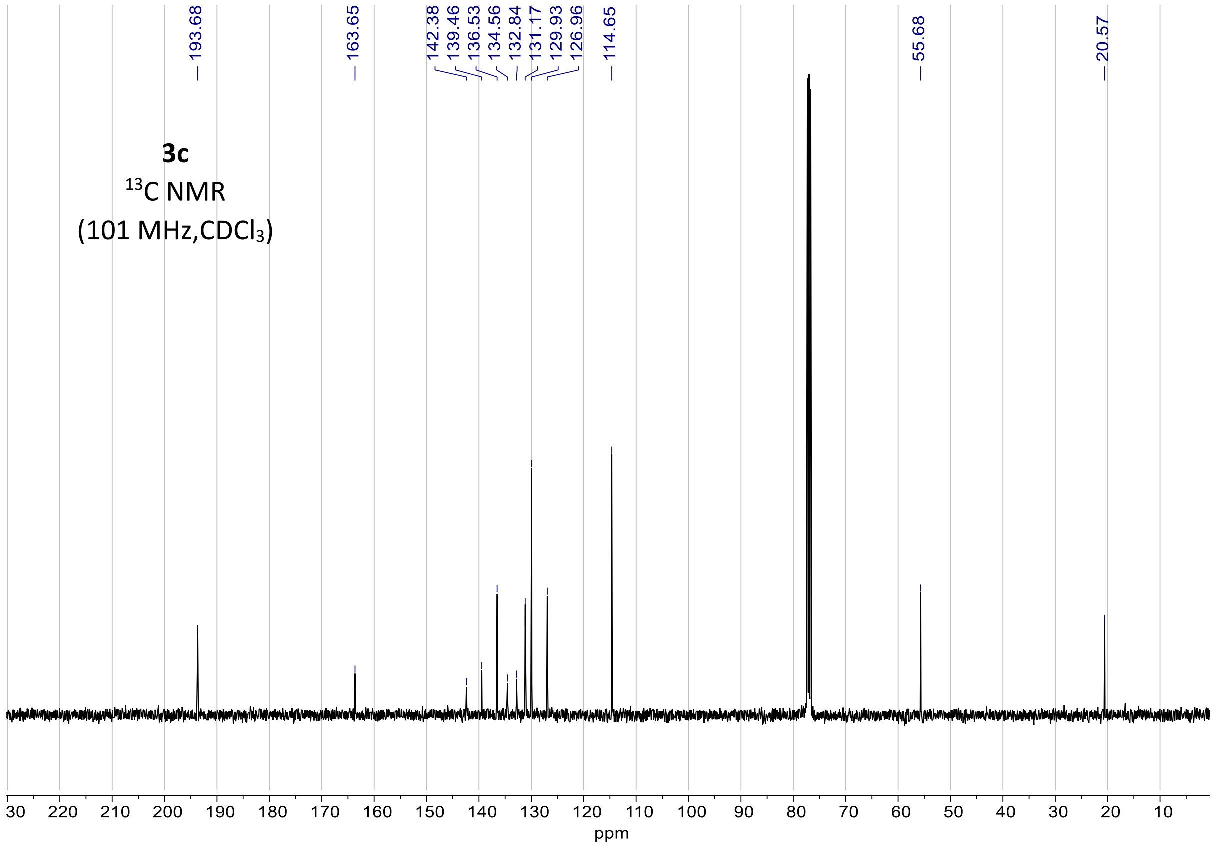
193.68
163.65
142.38
139.46
136.53
134.56
132.84
131.17
129.93
126.96
114.65
55.68
20.57
3c
^{13}C NMR
(101 MHz,CDCl$_3$)
ppm

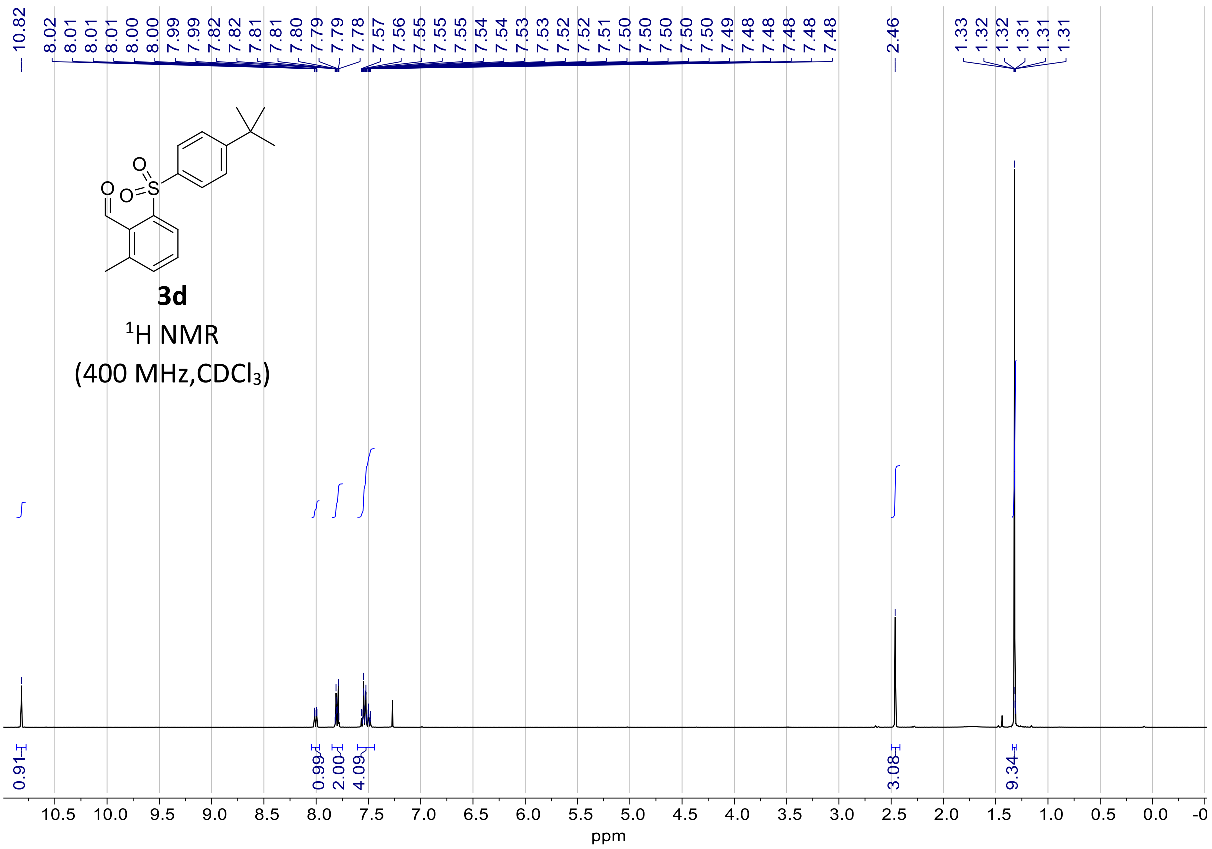
10.82
8.02 8.01 8.01 8.01 8.00 8.00 7.99 7.99 7.82 7.82 7.81 7.81 7.80 7.79 7.79 7.78 7.57 7.56 7.55 7.55 7.55 7.54 7.54 7.53 7.53 7.52 7.52 7.51 7.50 7.50 7.50 7.50 7.49 7.48 7.48 7.48 7.48 7.48
2.46
1.33 1.32 1.32 1.31 1.31 1.31
O
O
S
O
3d
^{1}H NMR
(400 MHz,CDCl$_3$)
0.91
0.99
2.00
4.09
3.08
9.34
ppm

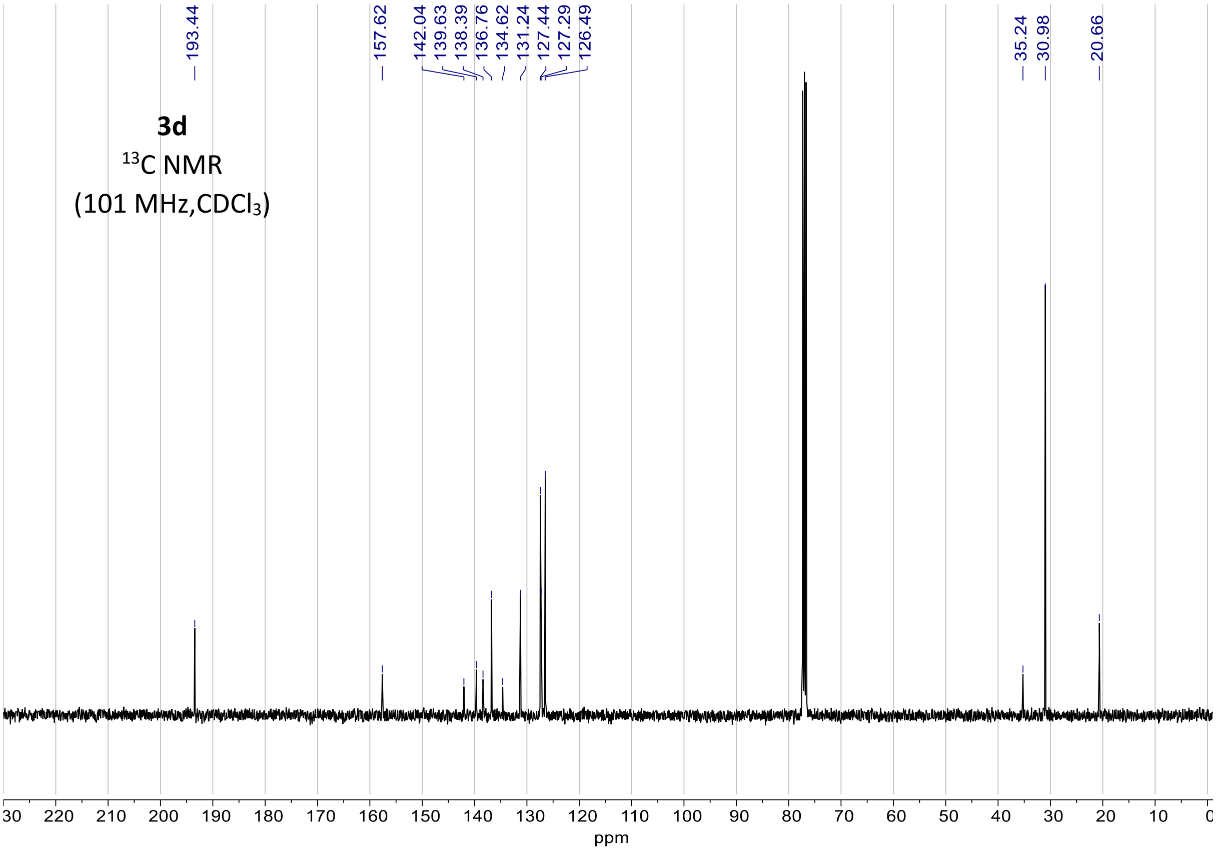
193.44
157.62
142.04 139.63 138.39 136.76 134.62 131.24 127.44 127.29 126.49
35.24
30.98
20.66
3d
^{13}C NMR
(101 MHz,CDCl$_3$)
ppm

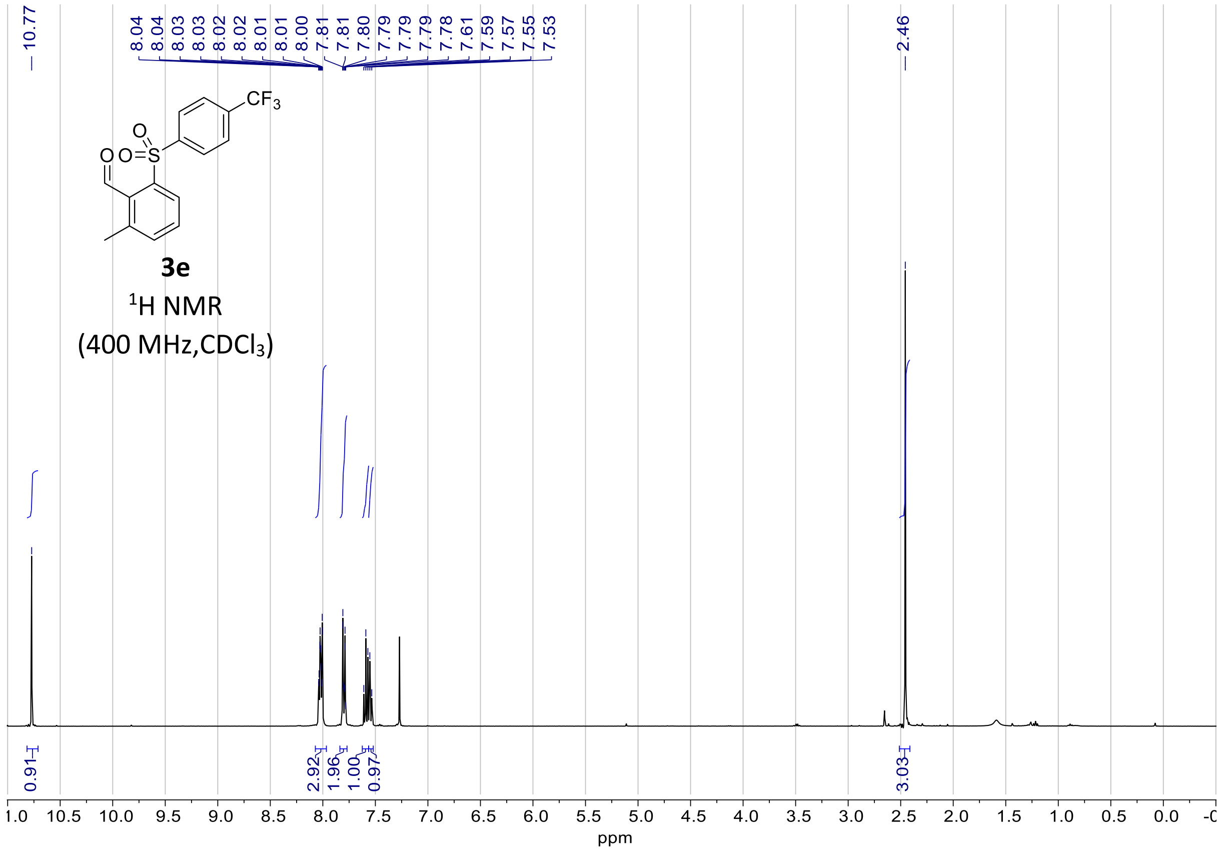
10.77
8.04 8.04 8.03 8.03 8.02 8.02 8.01 8.01 8.00 7.81 7.81 7.80 7.79 7.79 7.79 7.78 7.61 7.59 7.57 7.55 7.53
2.46
CF_3
3e
^{1}H NMR
(400 MHz,$CDCl_3$)
0.91
2.92
1.96
1.00
0.97
3.03
ppm

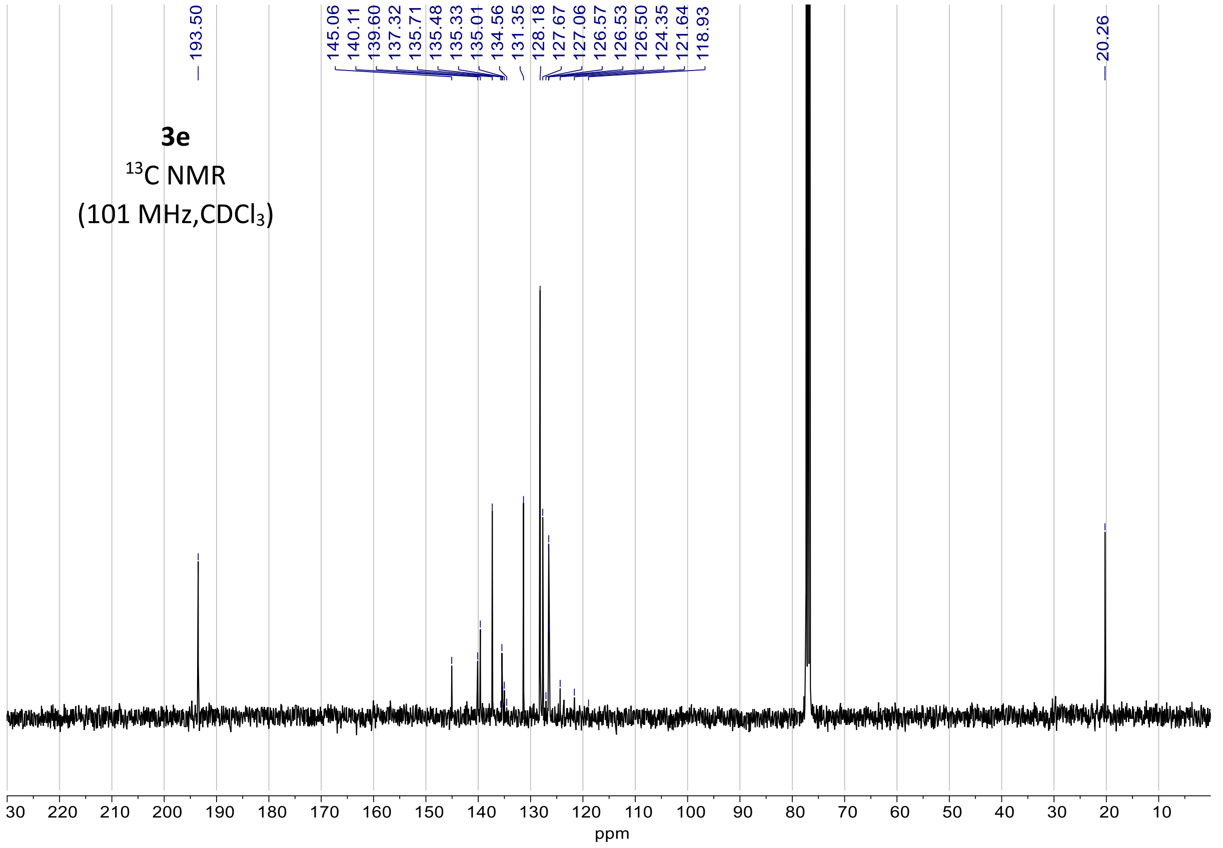
193.50
145.06 140.11 139.60 137.32 135.71 135.48 135.33 135.01 134.56 131.35 128.18 127.67 127.06 126.57 126.53 126.50 124.35 121.64 118.93
20.26
3e
^{13}C NMR
(101 MHz,$CDCl_3$)
ppm

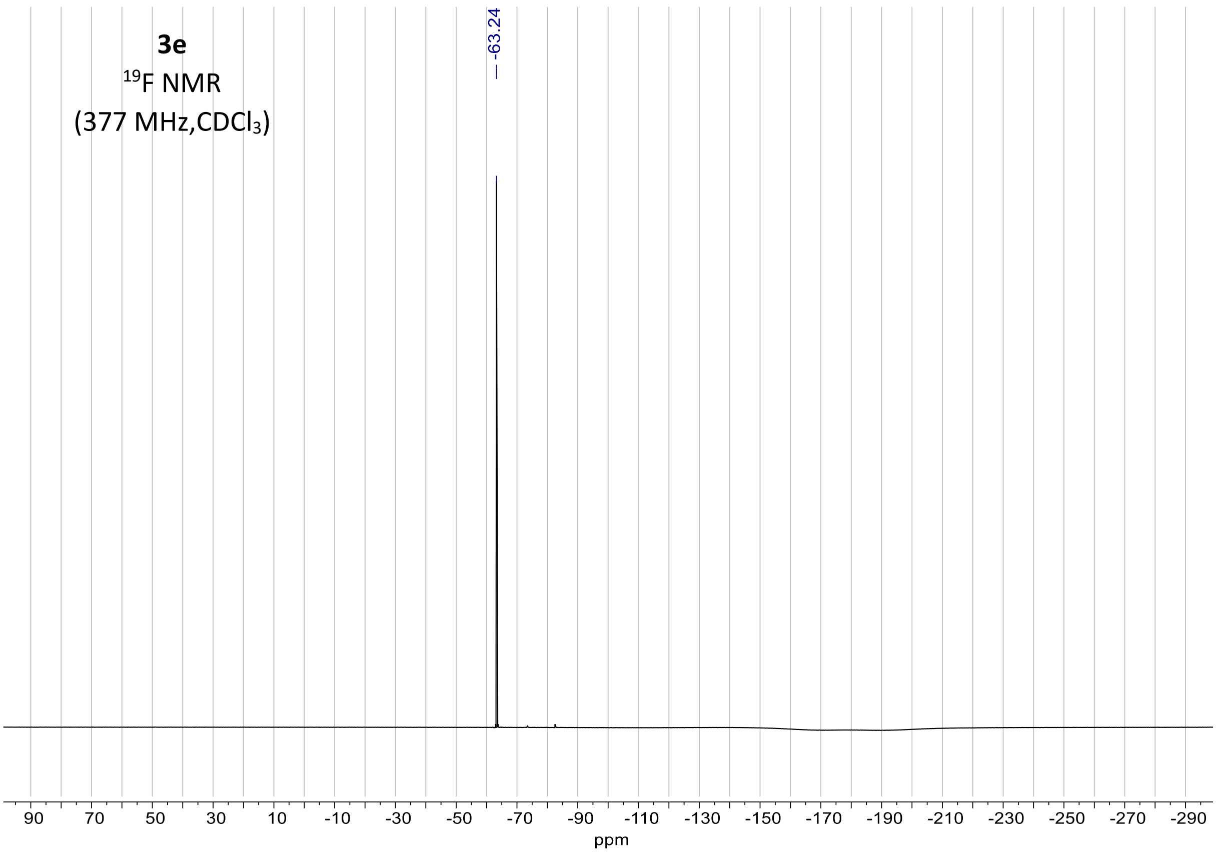
3e
^{19}F NMR
(377 MHz,CDCl$_3$)
-63.24
90 70 50 30 10 -10 -30 -50 -70 -90 -110 -130 -150 -170 -190 -210 -230 -250 -270 -290
ppm

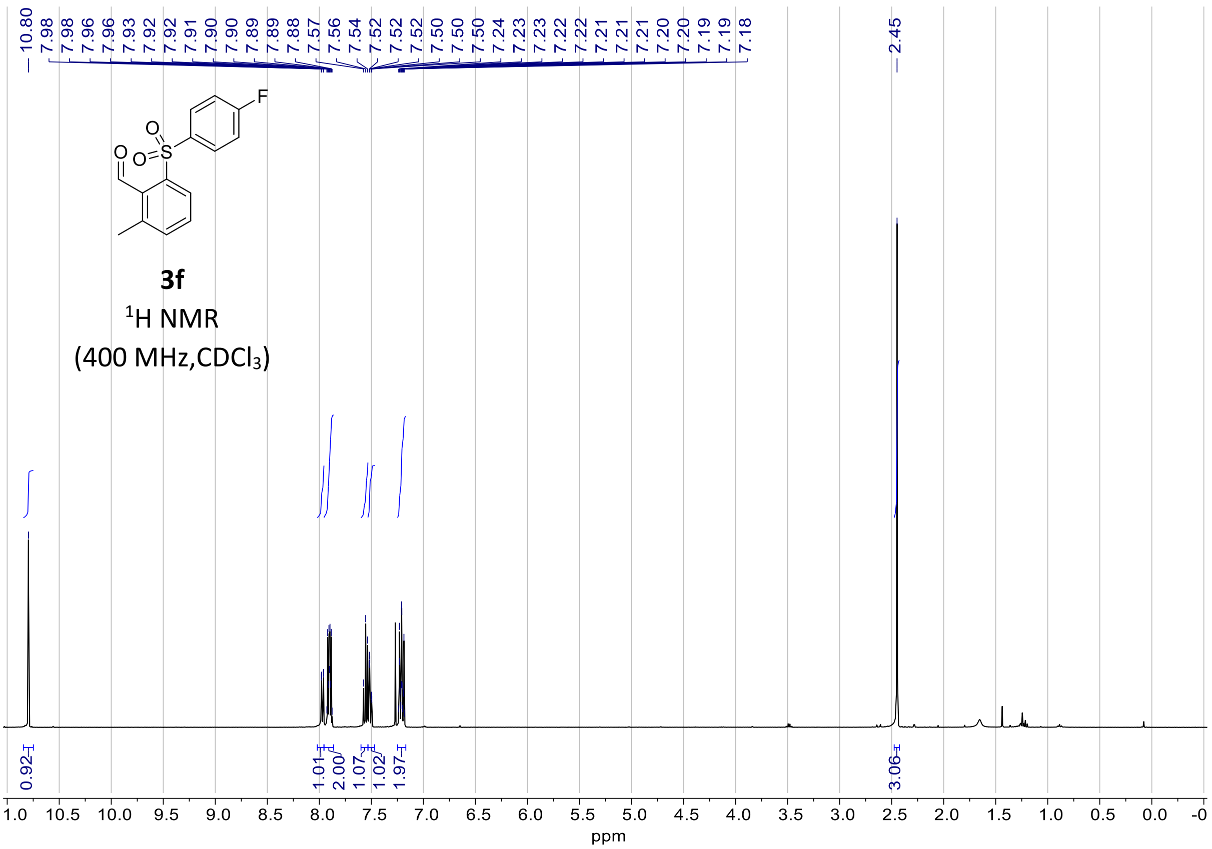
10.80 7.98 7.98 7.96 7.96 7.93 7.92 7.92 7.91 7.90 7.90 7.89 7.89 7.88 7.57 7.56 7.54 7.52 7.52 7.52 7.50 7.50 7.50 7.24 7.23 7.23 7.22 7.22 7.21 7.21 7.21 7.20 7.20 7.19 7.19 7.18
2.45
F
O
O
S
O
3f
1H NMR
(400 MHz,CDCl3)
0.92
1.01
2.00
1.07
1.02
1.97
3.06
ppm

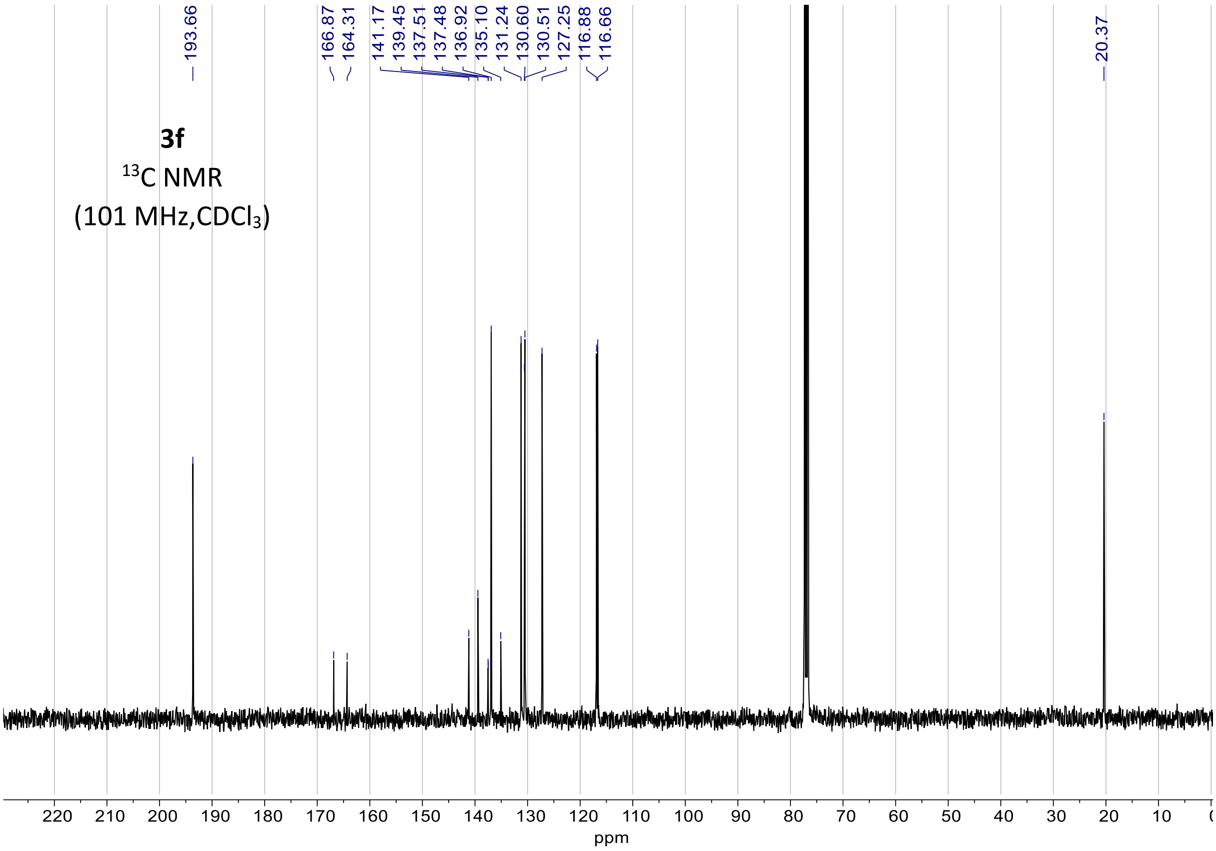
193.66
166.87 164.31
141.17 139.45 137.51 137.48 136.92 135.10 131.24 130.60 130.51 127.25 116.88 116.66
20.37
3f
13C NMR
(101 MHz,CDCl3)
ppm

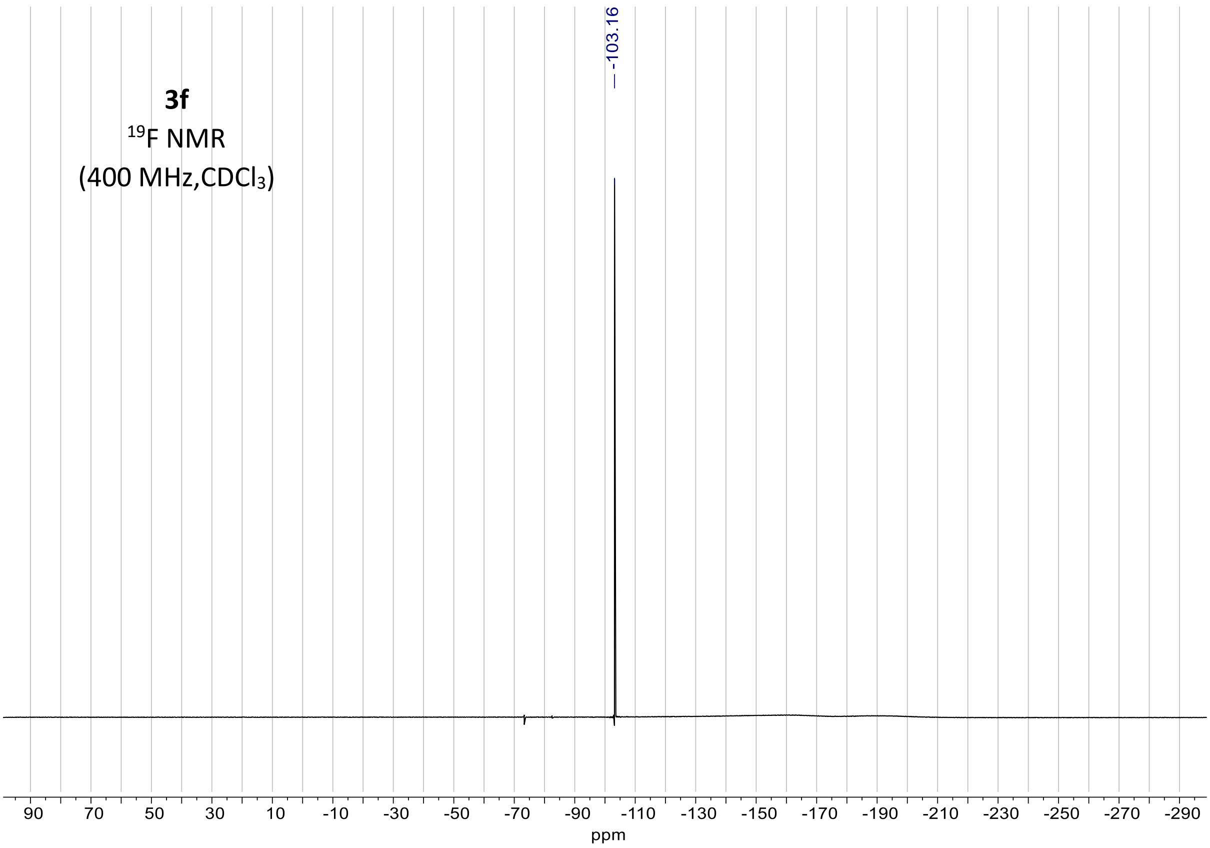
-103.16
3f
19F NMR
(400 MHz,CDCl3)
90 70 50 30 10 -10 -30 -50 -70 -90 -110 -130 -150 -170 -190 -210 -230 -250 -270 -290
ppm

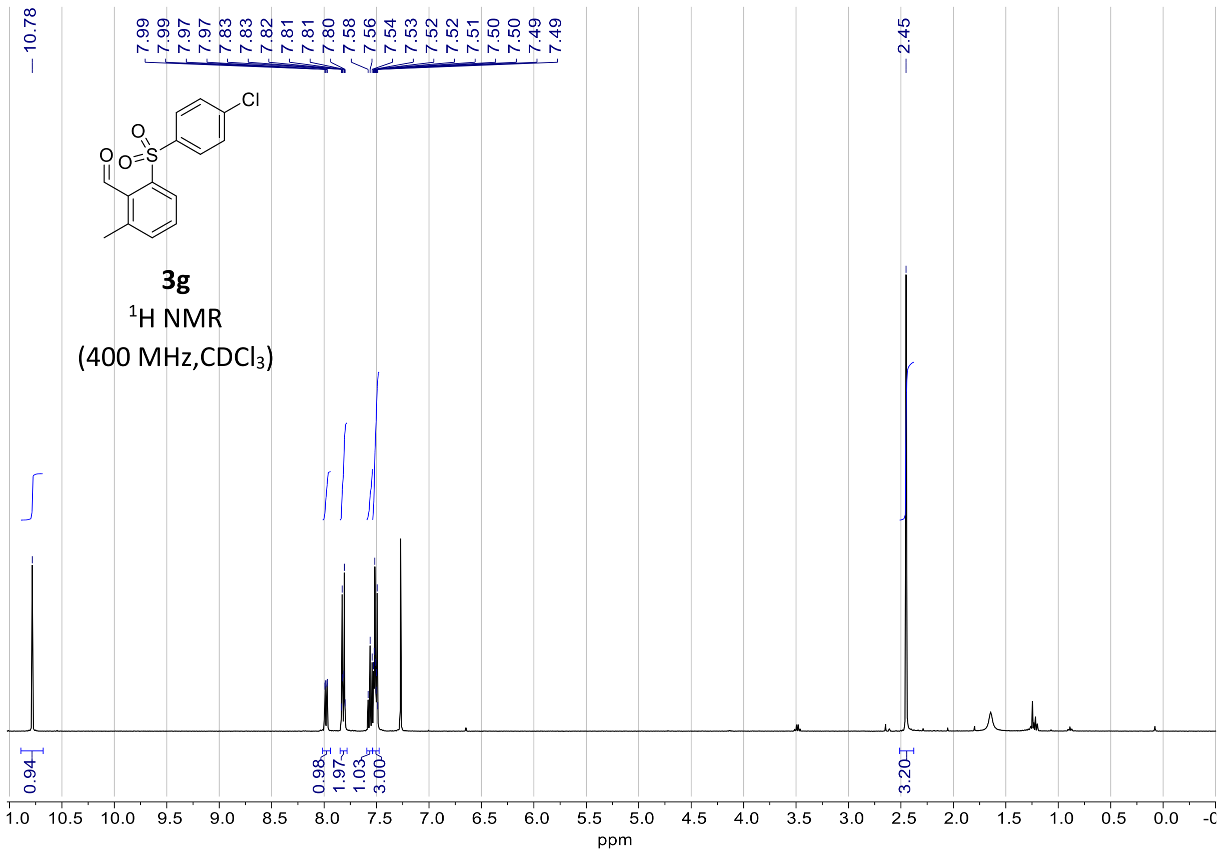
10.78
7.99 7.99 7.97 7.97 7.83 7.83 7.82 7.81 7.81 7.80 7.58 7.56 7.54 7.53 7.52 7.52 7.51 7.50 7.50 7.49 7.49
2.45
Cl
O
O
S
O
3g
1H NMR
(400 MHz,CDCl3)
0.94
0.98
1.97
1.03
3.00
3.20
1.0 10.5 10.0 9.5 9.0 8.5 8.0 7.5 7.0 6.5 6.0 5.5 5.0 4.5 4.0 3.5 3.0 2.5 2.0 1.5 1.0 0.5 0.0
ppm

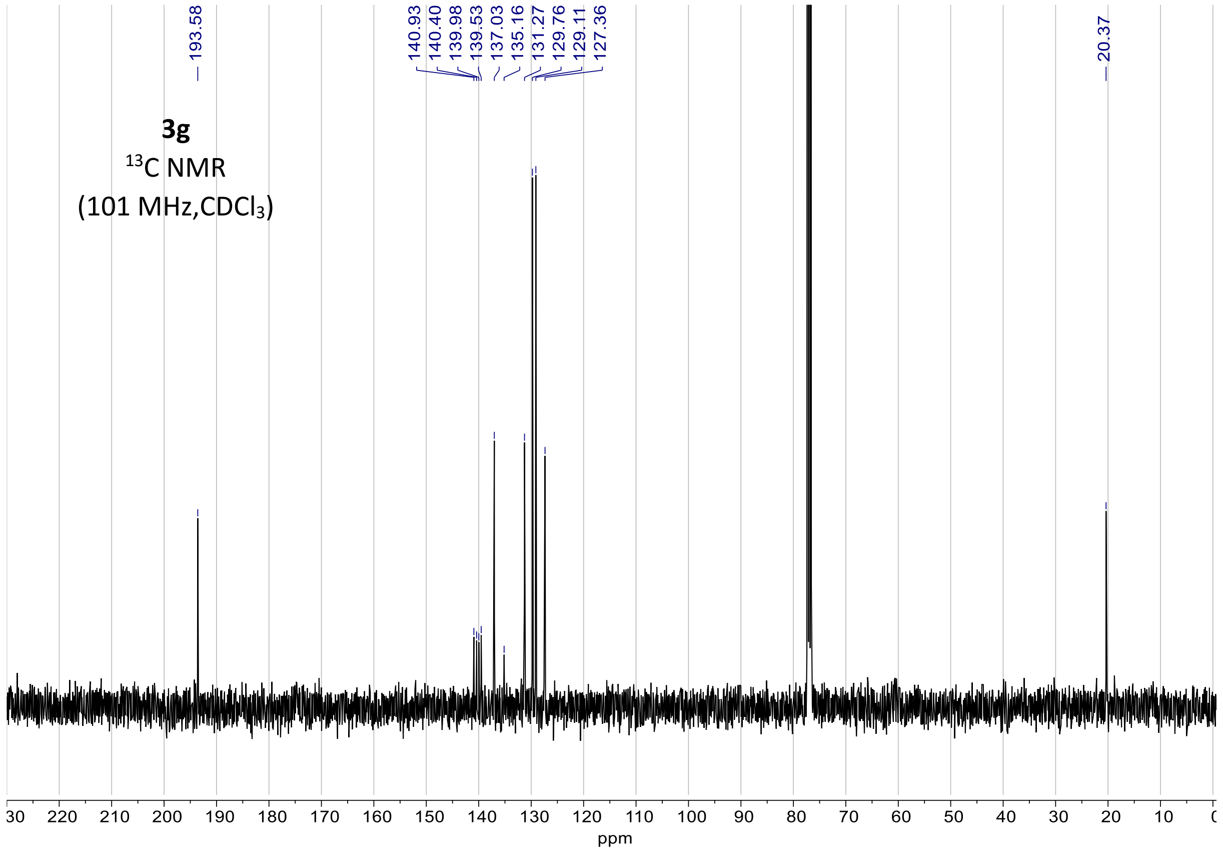
193.58
140.93 140.40 139.98 139.53 137.03 135.16 131.27 129.76 129.11 127.36
20.37
3g
13C NMR
(101 MHz,CDCl3)
30 220 210 200 190 180 170 160 150 140 130 120 110 100 90 80 70 60 50 40 30 20 10
ppm

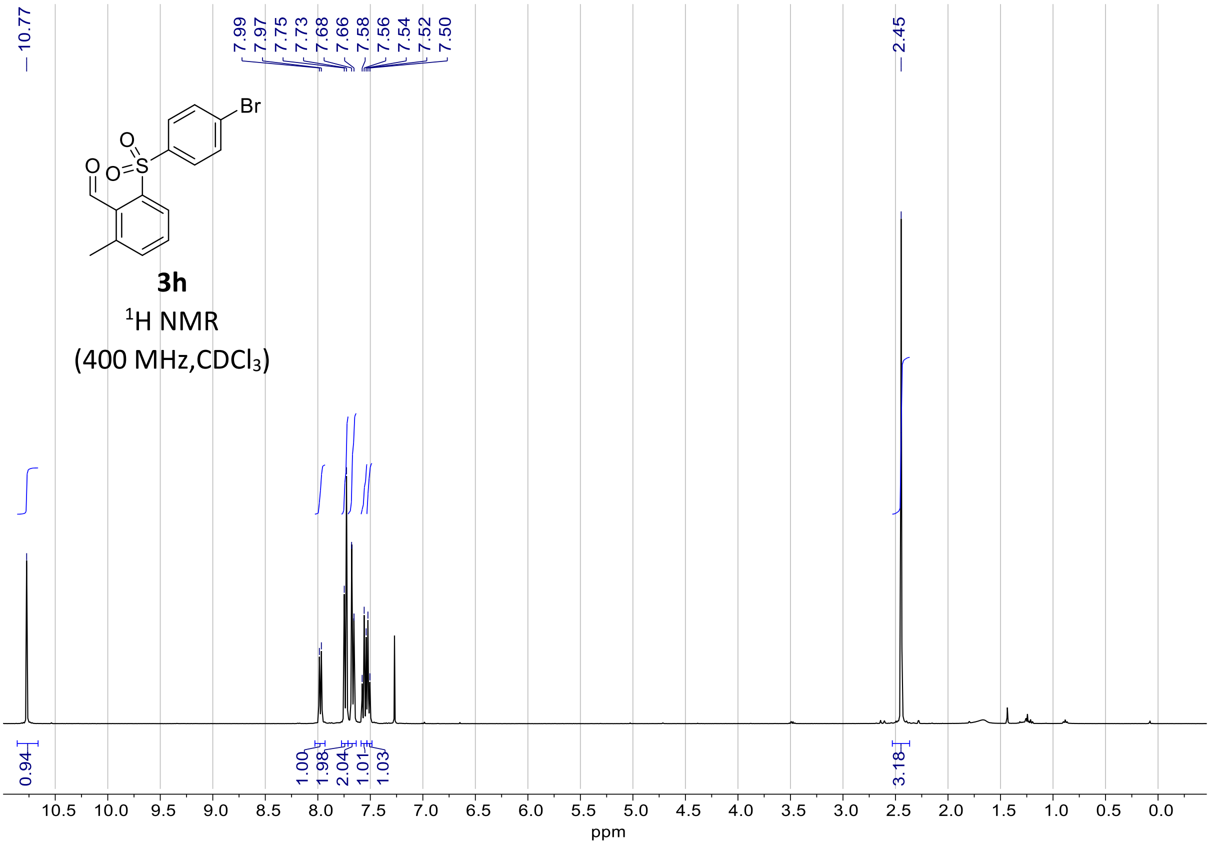
10.77
7.99
7.97
7.75
7.73
7.68
7.66
7.58
7.56
7.54
7.52
7.50
2.45
Br
O
O
O
S
3h
1H NMR
(400 MHz,CDCl3)
0.94
1.00
1.98
2.04
1.01
1.03
3.18
10.5 10.0 9.5 9.0 8.5 8.0 7.5 7.0 6.5 6.0 5.5 5.0 4.5 4.0 3.5 3.0 2.5 2.0 1.5 1.0 0.5 0.0
ppm

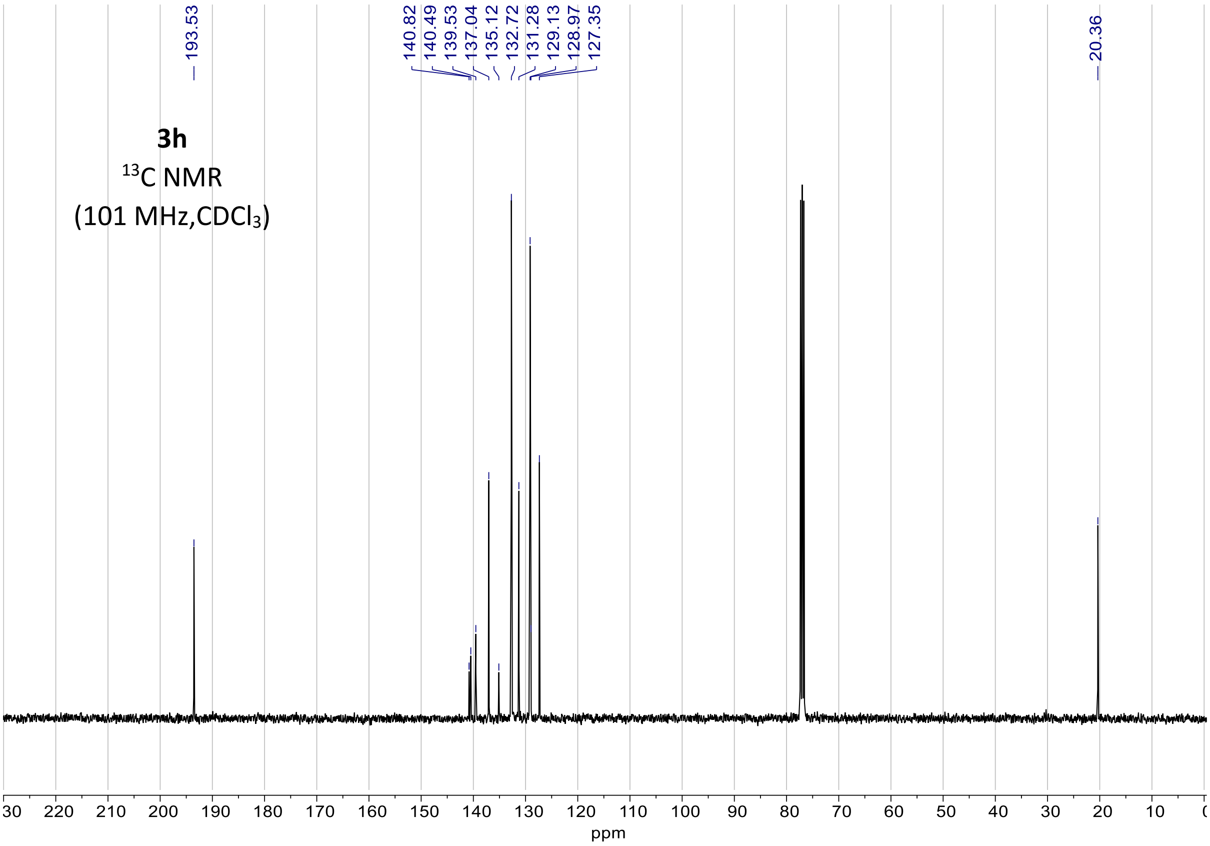
193.53
140.82
140.49
139.53
137.04
135.12
132.72
131.28
129.13
128.97
127.35
20.36
3h
13C NMR
(101 MHz,CDCl3)
30 220 210 200 190 180 170 160 150 140 130 120 110 100 90 80 70 60 50 40 30 20 10
ppm

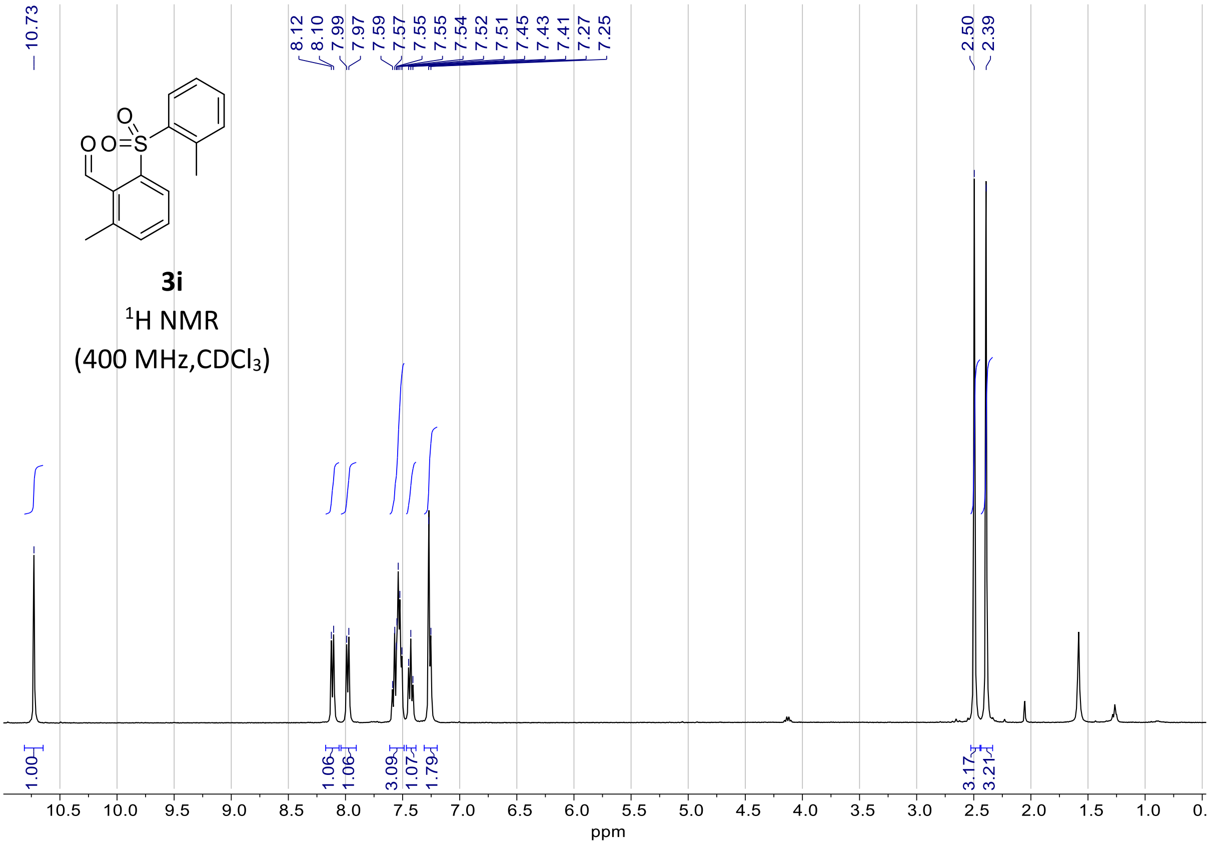

3i
^{1}H NMR
(400 MHz, $CDCl_3$)

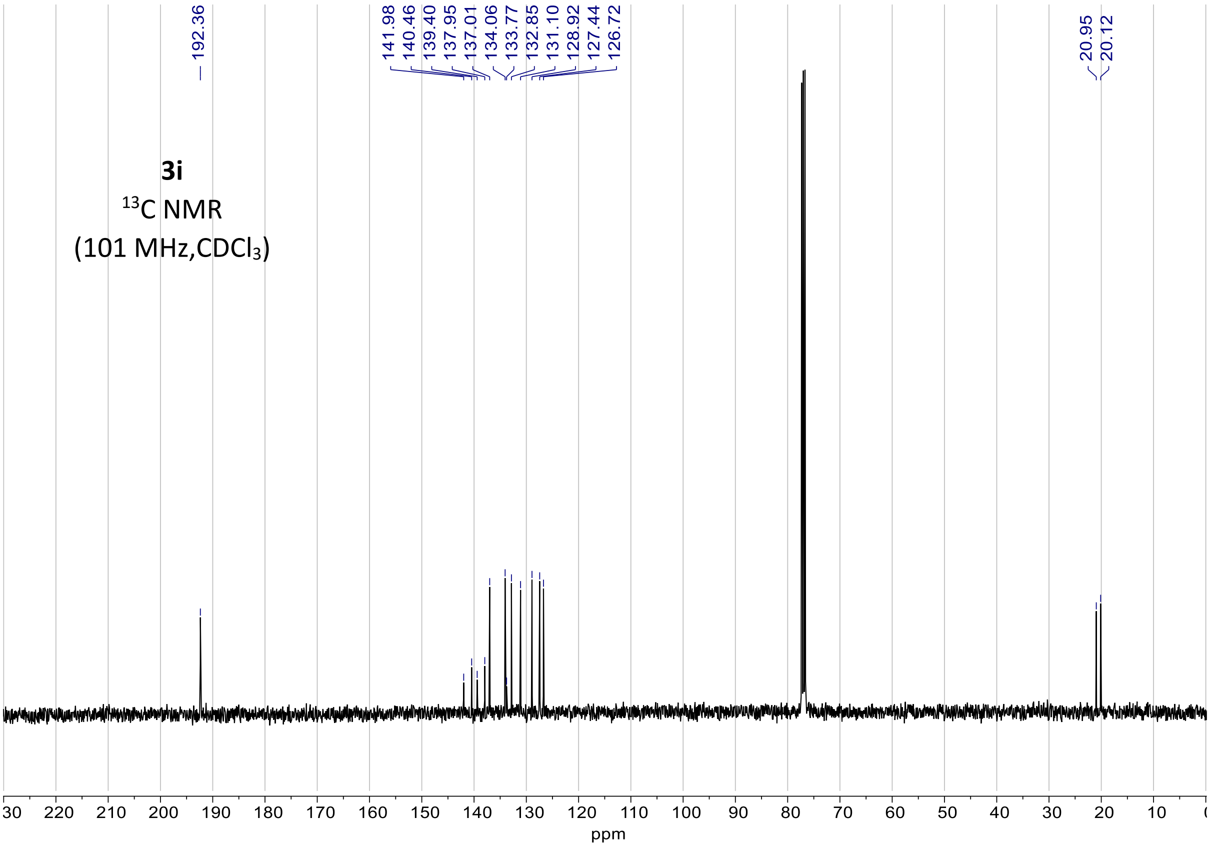

3i
^{13}C NMR
(101 MHz, $CDCl_3$)

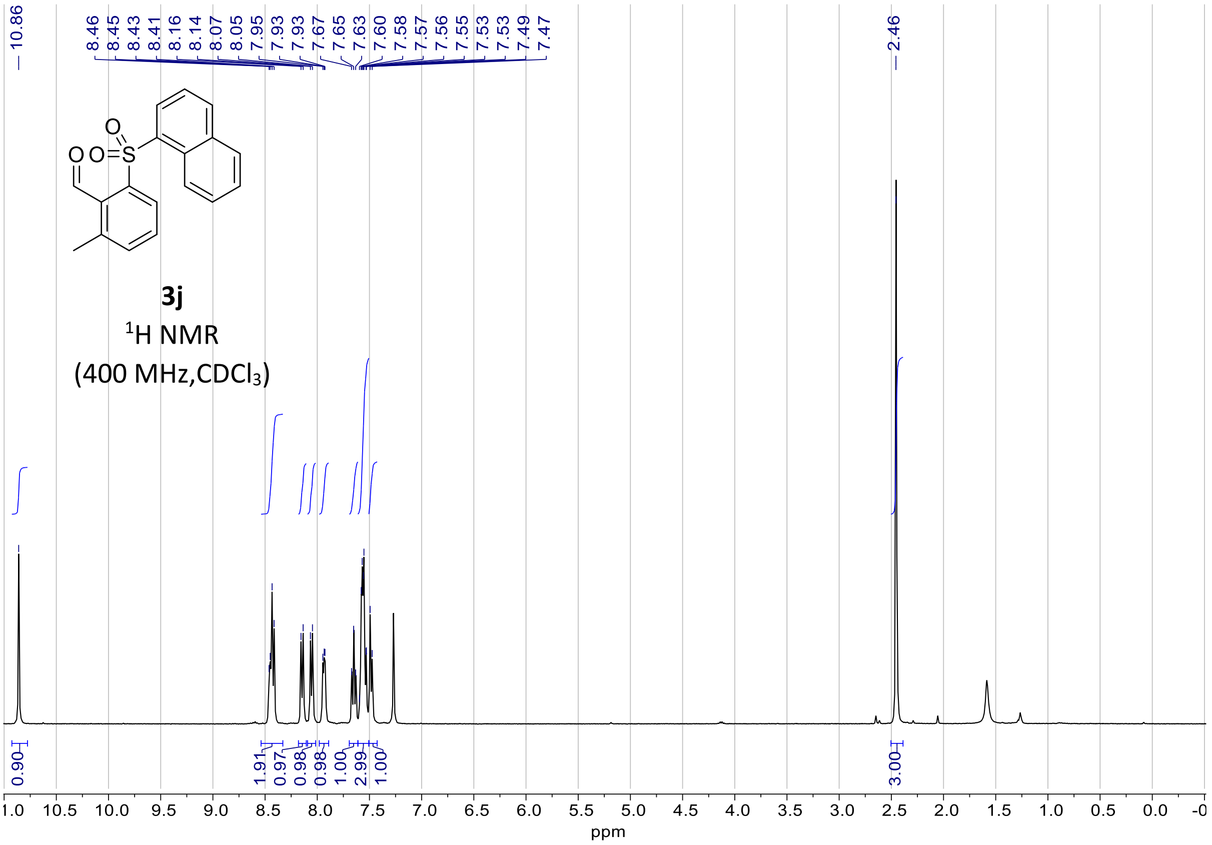
10.86
8.46 8.45 8.43 8.41 8.16 8.14 8.07 8.05 7.95 7.93 7.93 7.67 7.65 7.63 7.60 7.58 7.57 7.56 7.55 7.53 7.53 7.49 7.47
2.46
3j
1H NMR
(400 MHz,CDCl3)
0.90 1.91 0.97 0.98 0.98 1.00 2.99 1.00 3.00
ppm

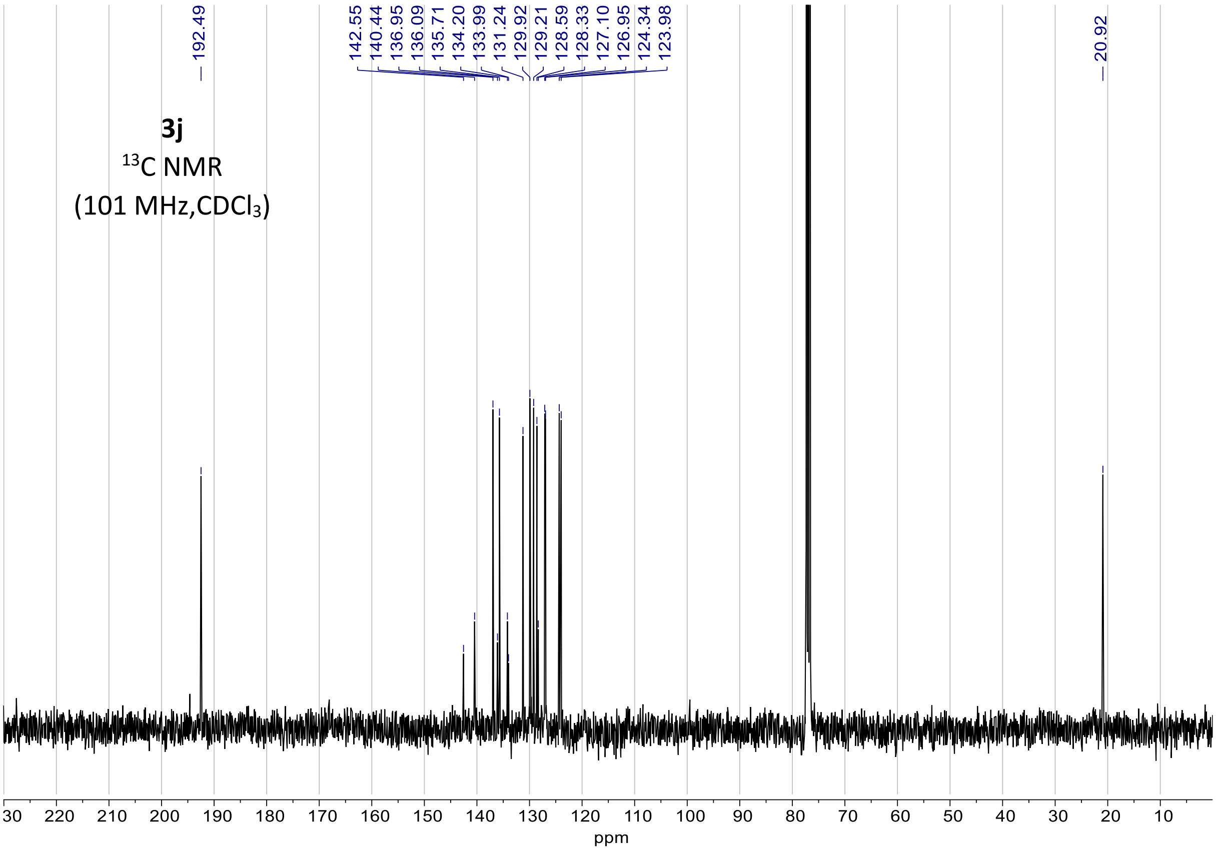
192.49
142.55 140.44 136.95 136.09 135.71 134.20 133.99 131.24 129.92 129.21 128.59 128.33 127.10 126.95 124.34 123.98
20.92
3j
13C NMR
(101 MHz,CDCl3)
ppm

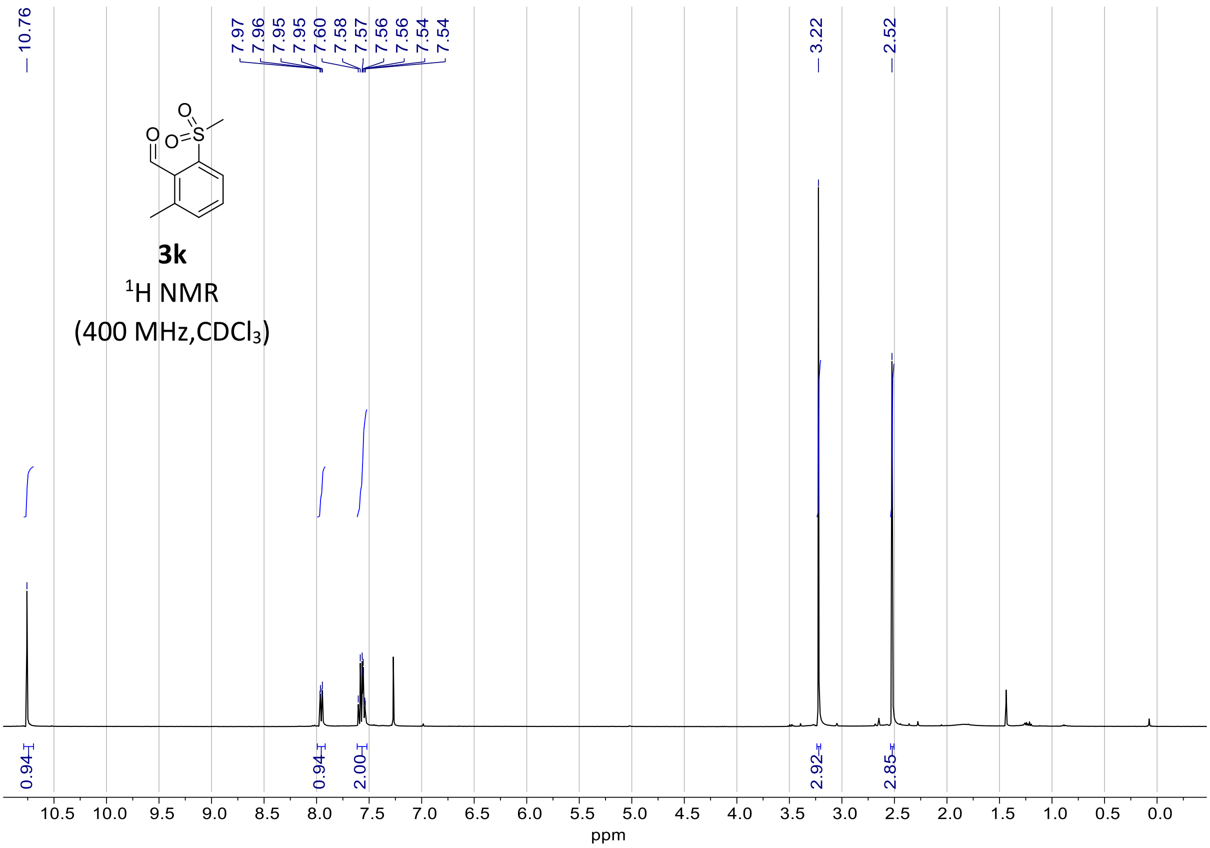

10.76
7.97 7.96 7.95 7.95 7.60 7.58 7.57 7.56 7.56 7.54 7.54
3.22
2.52
3k
1H NMR
(400 MHz,CDCl3)
0.94
0.94
2.00
2.92
2.85
10.5 10.0 9.5 9.0 8.5 8.0 7.5 7.0 6.5 6.0 5.5 5.0 4.5 4.0 3.5 3.0 2.5 2.0 1.5 1.0 0.5 0.0
ppm

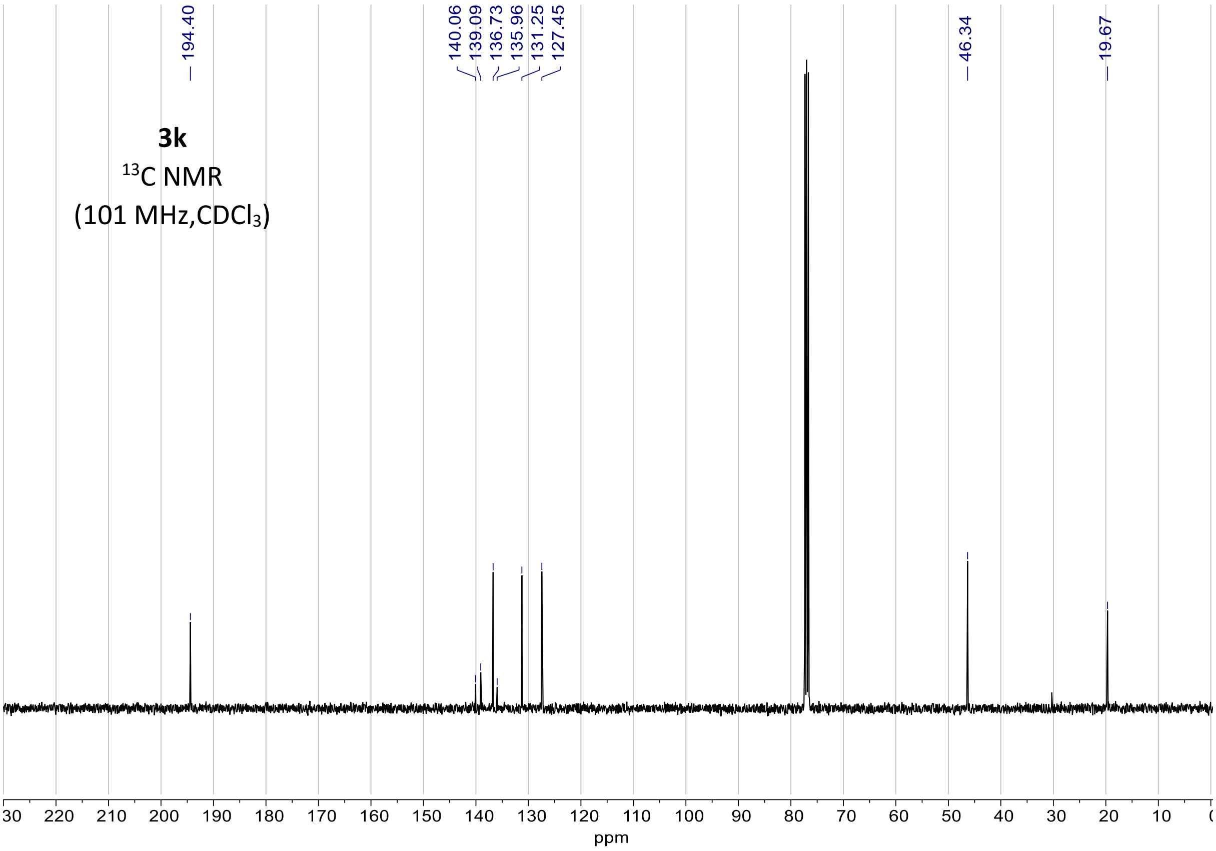

194.40
140.06 139.09 136.73 135.96 131.25 127.45
46.34
19.67
3k
13C NMR
(101 MHz,CDCl3)
30 220 210 200 190 180 170 160 150 140 130 120 110 100 90 80 70 60 50 40 30 20 10
ppm

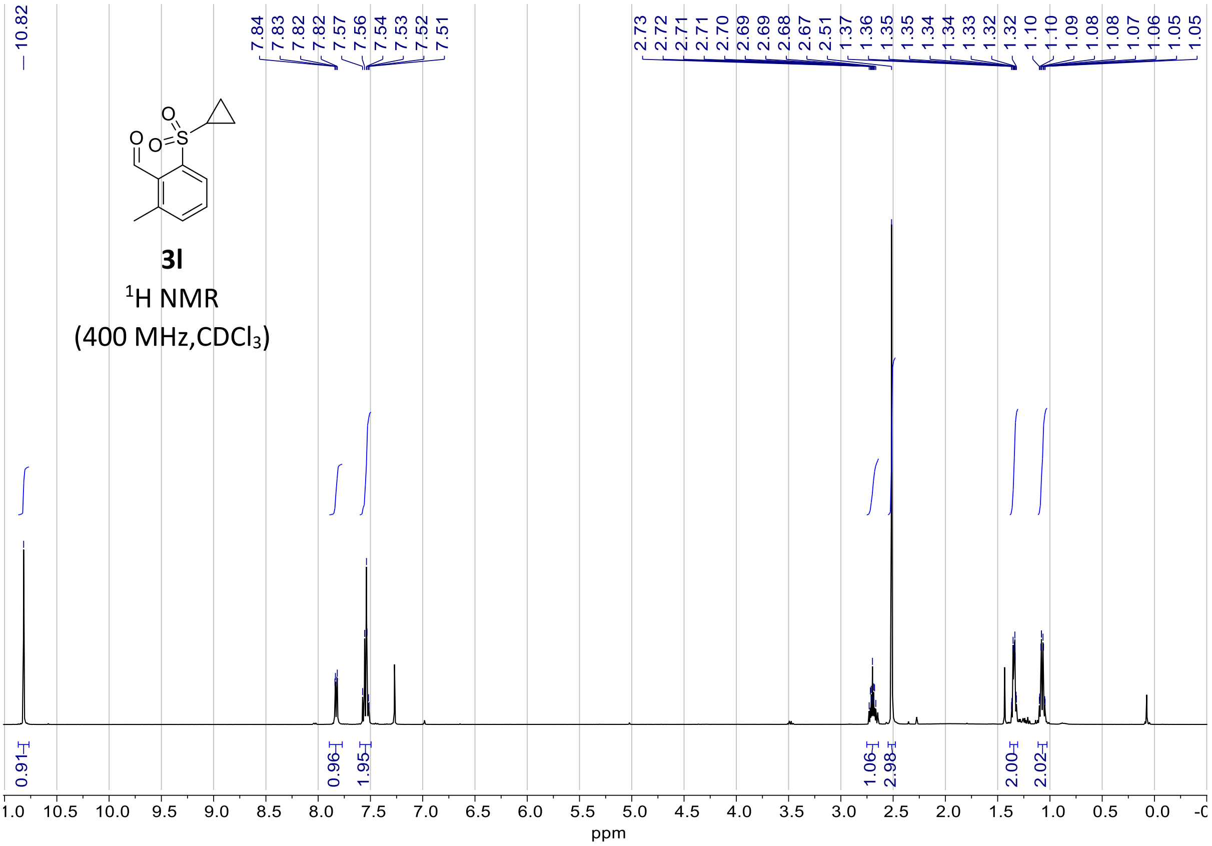
10.82
7.84 7.83 7.82 7.82 7.57 7.56 7.54 7.53 7.52 7.51
2.73 2.72 2.71 2.71 2.70 2.69 2.69 2.68 2.67 2.51 1.37 1.36 1.35 1.35 1.34 1.34 1.33 1.32 1.32 1.10 1.10 1.09 1.08 1.08 1.07 1.06 1.05 1.05
O
O
O
S
3l
^{1}H NMR
(400 MHz,CDCl$_3$)
0.91 0.96 1.95 1.06 2.98 2.00 2.02
ppm

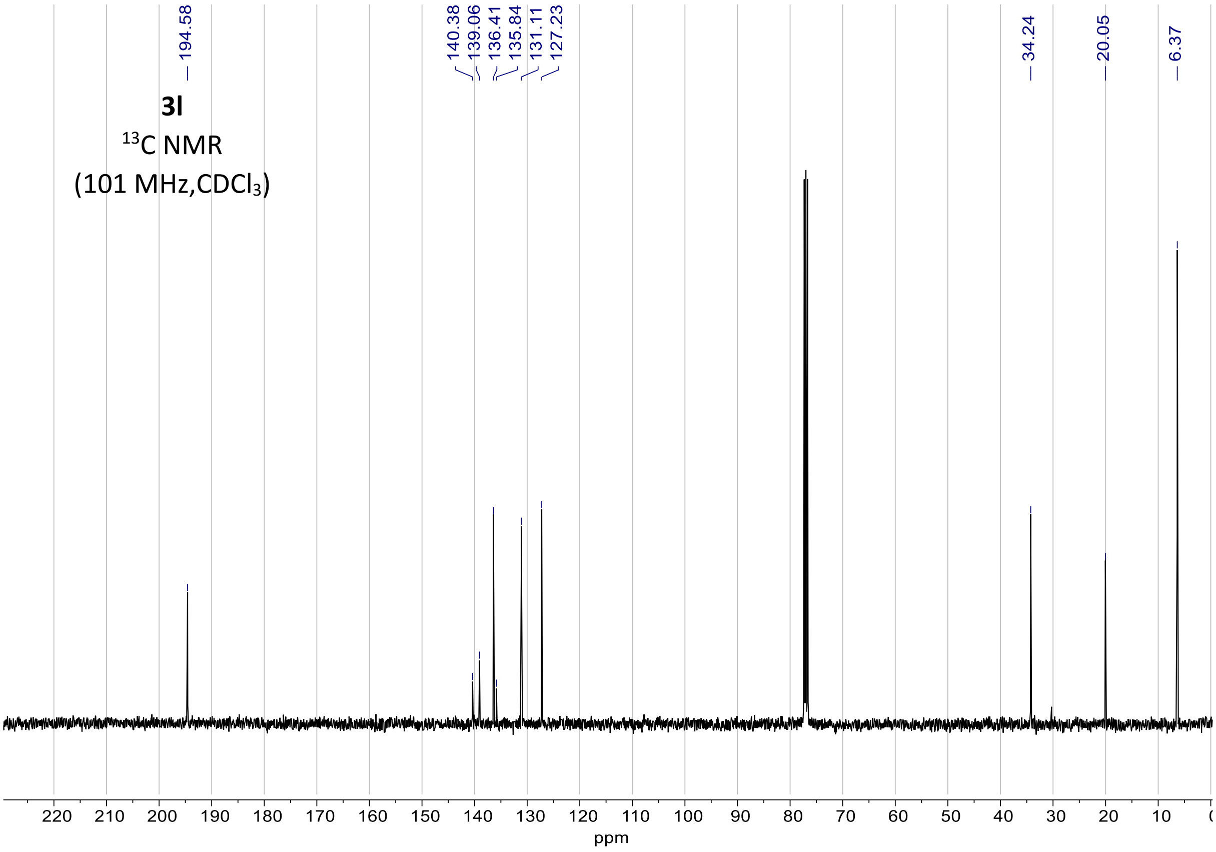
194.58
140.38 139.06 136.41 135.84 131.11 127.23
34.24 20.05 6.37
3l
^{13}C NMR
(101 MHz,CDCl$_3$)
ppm

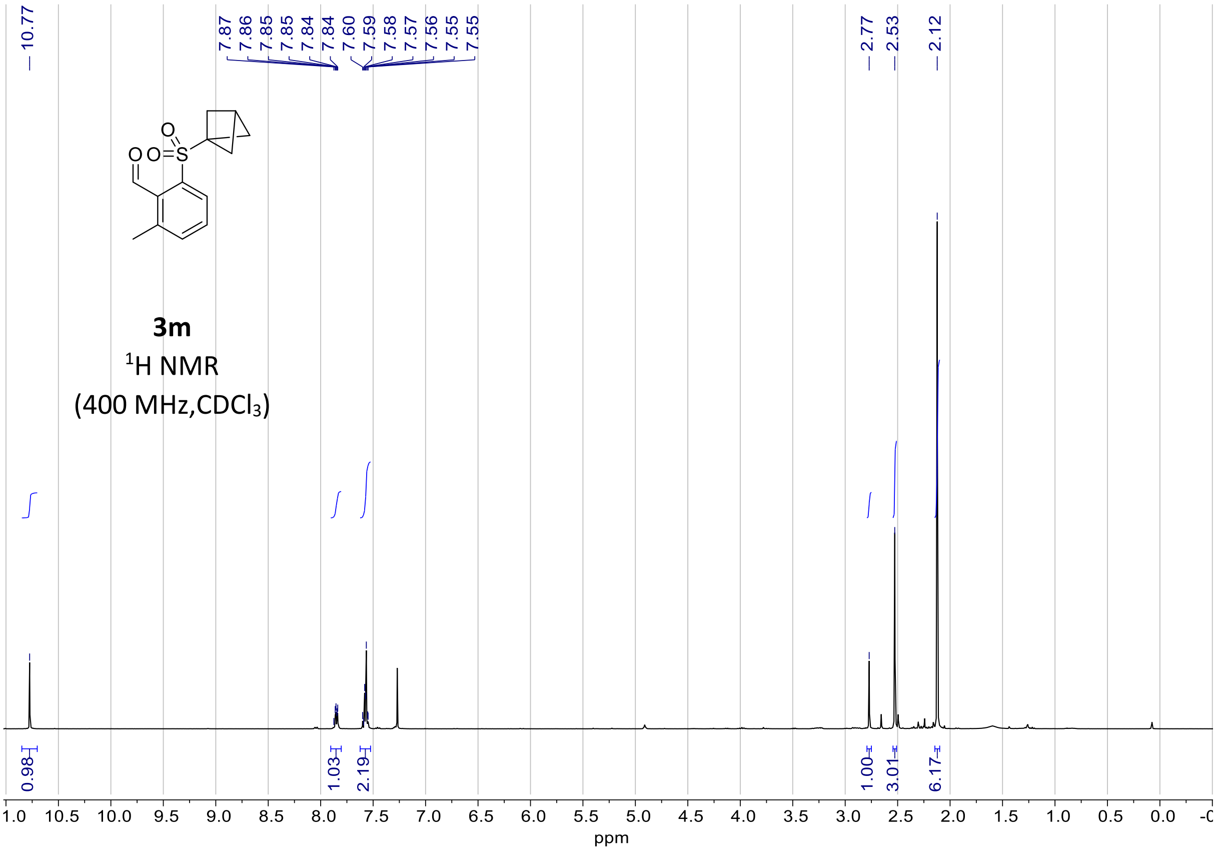
10.77
7.87
7.86
7.85
7.85
7.84
7.84
7.60
7.59
7.58
7.57
7.56
7.55
7.55
2.77
2.53
2.12
3m
¹H NMR
(400 MHz,CDCl₃)
0.98
1.03
2.19
1.00
3.01
6.17
ppm

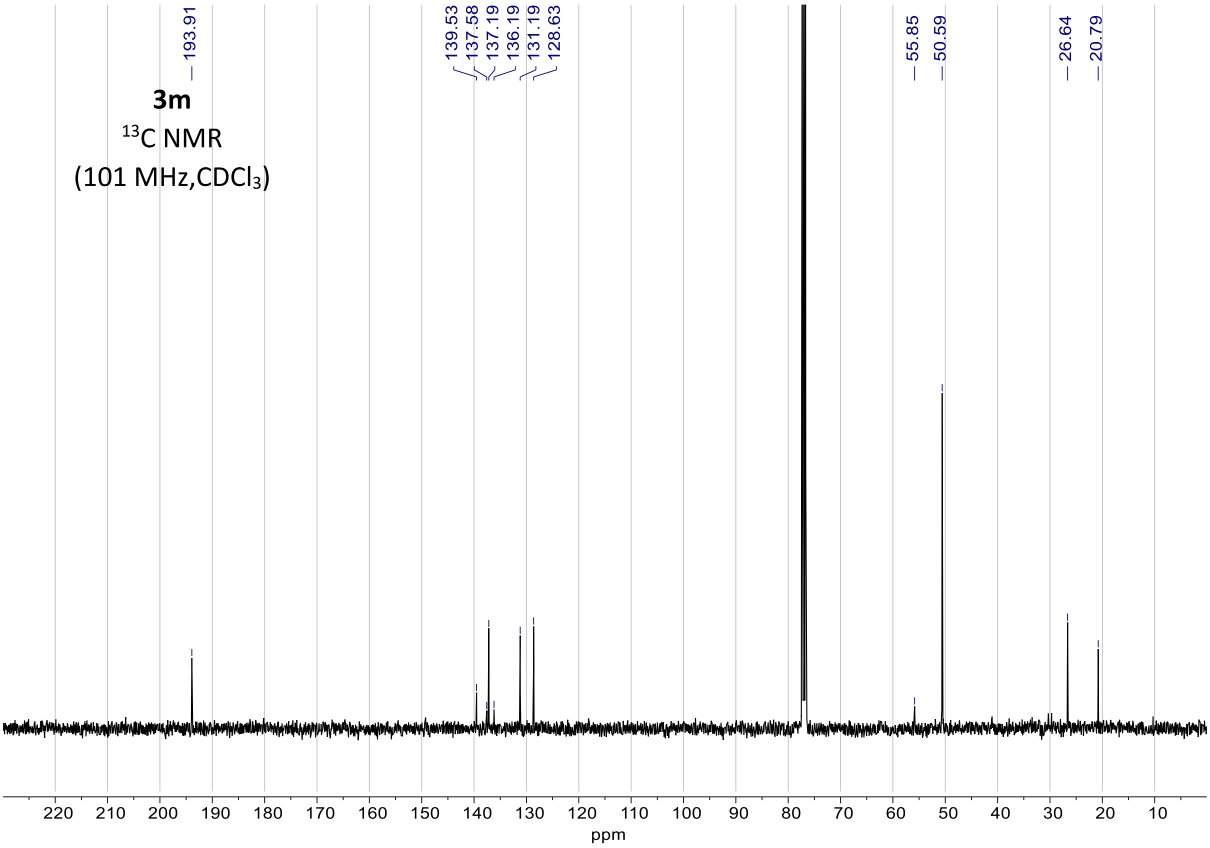
193.91
139.53
137.58
137.19
136.19
131.19
128.63
55.85
50.59
26.64
20.79
3m
¹³C NMR
(101 MHz,CDCl₃)
ppm

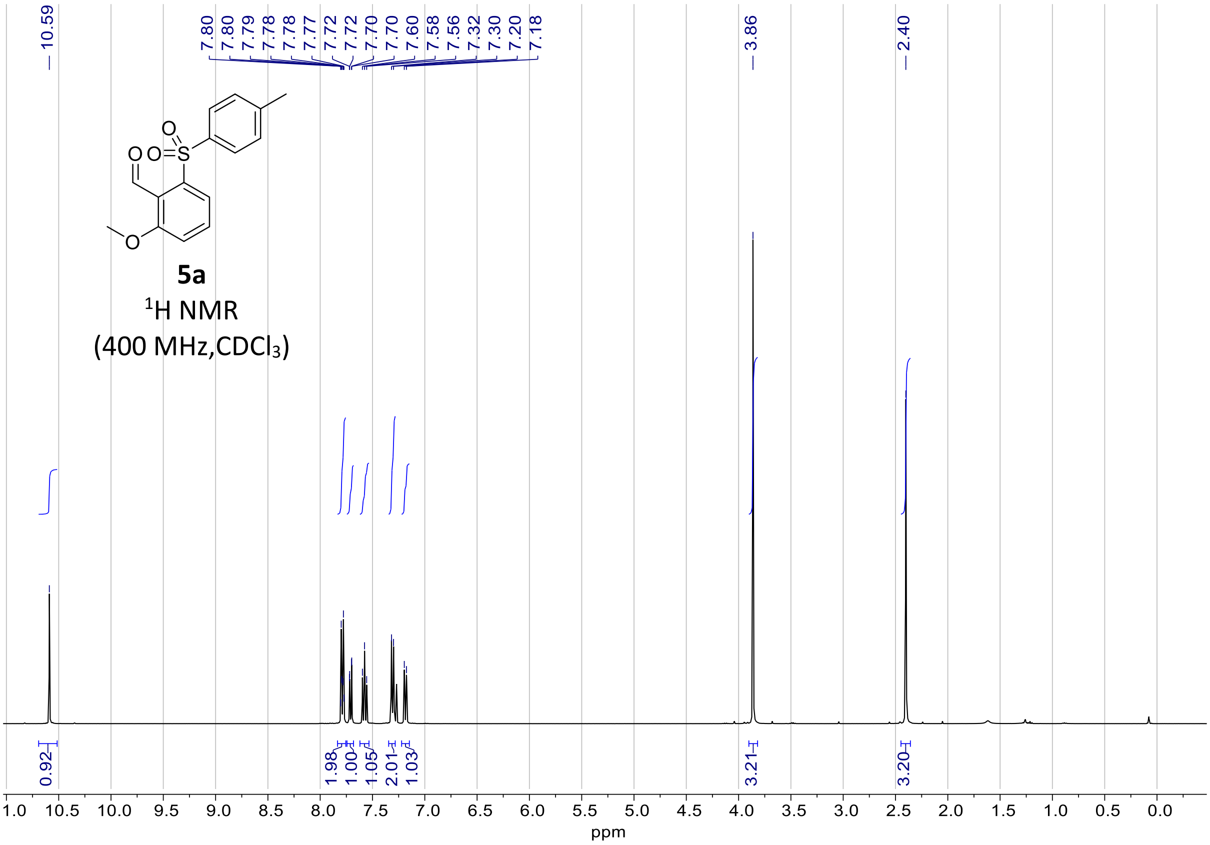
10.59
7.80
7.80
7.79
7.78
7.78
7.77
7.72
7.72
7.70
7.70
7.60
7.58
7.56
7.32
7.30
7.20
7.18
3.86
2.40
5a
1H NMR
(400 MHz,CDCl3)
0.92
1.98
1.00
1.05
2.01
1.03
3.21
3.20
ppm

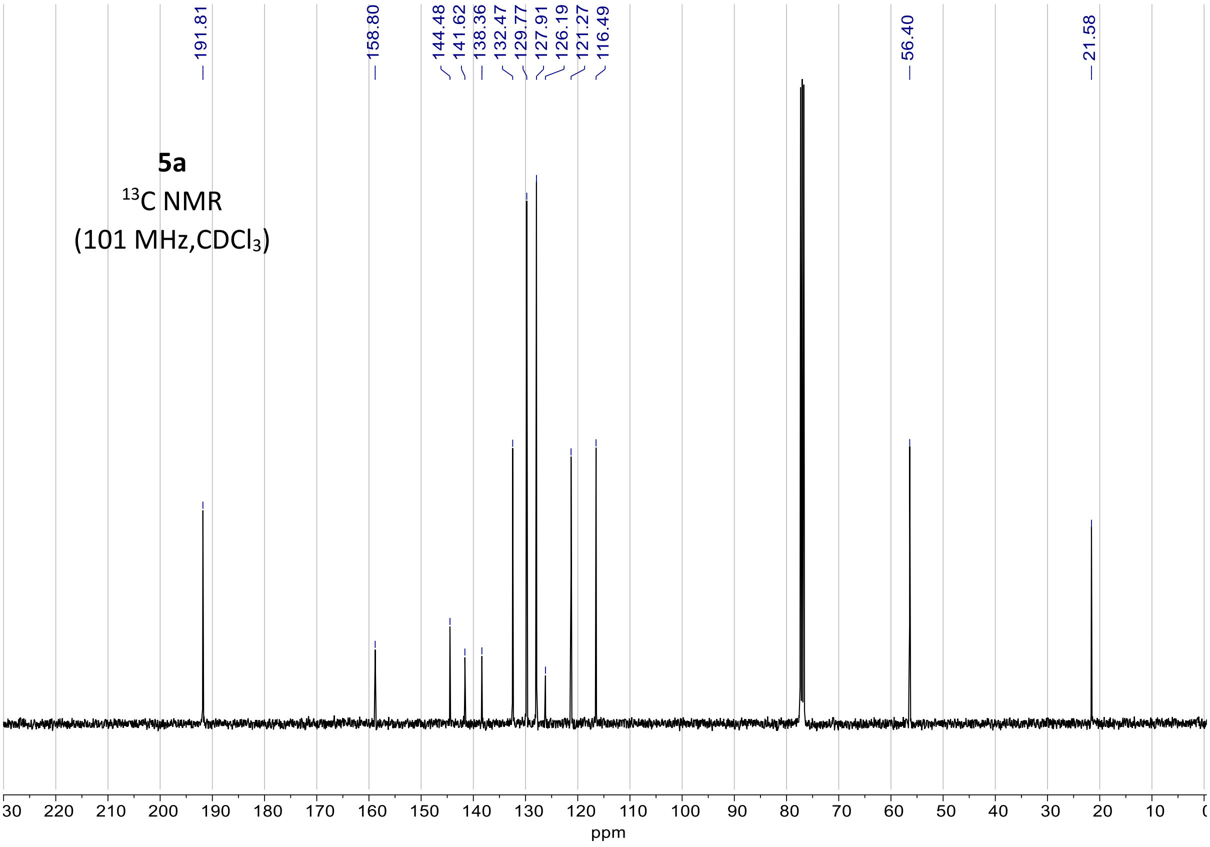
191.81
158.80
144.48
141.62
138.36
132.47
129.77
127.91
126.19
121.27
116.49
56.40
21.58
5a
13C NMR
(101 MHz,CDCl3)
ppm

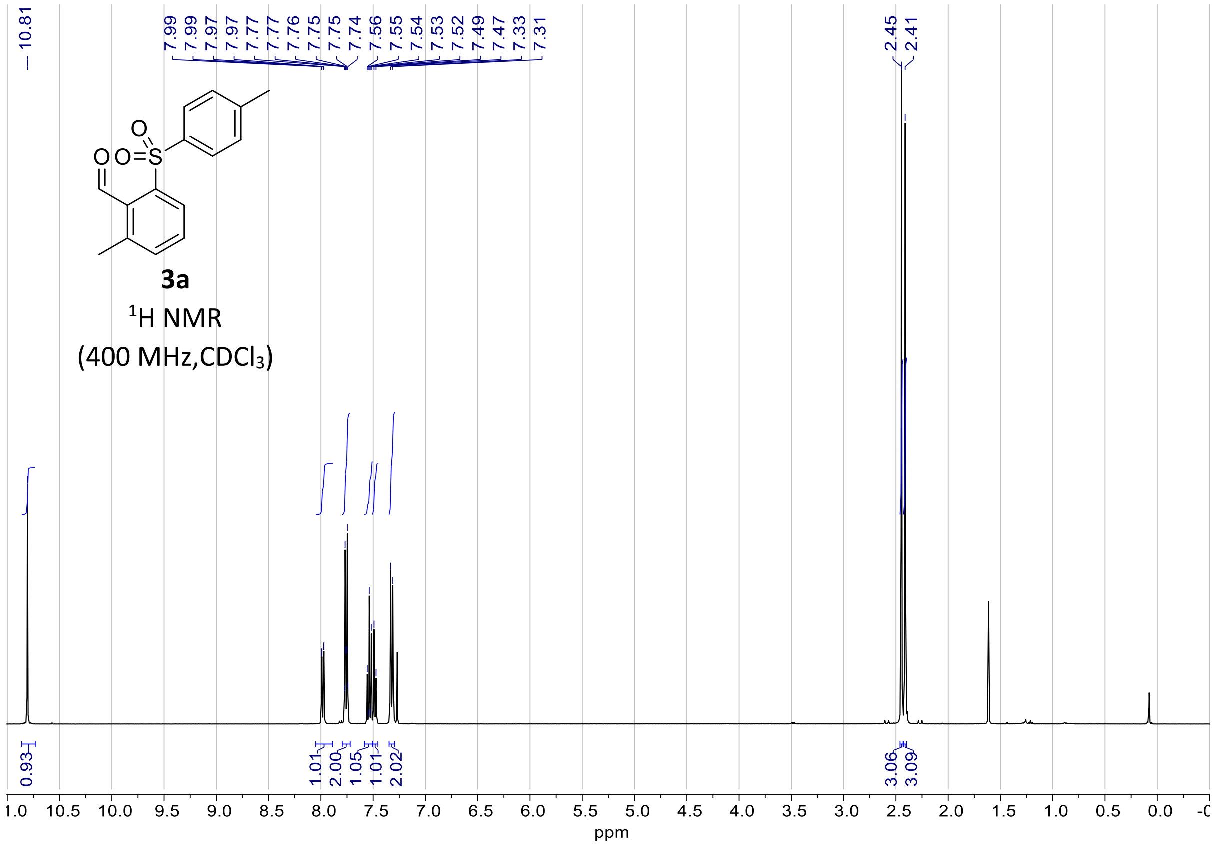
3a
^{1}H NMR
(400 MHz,CDCl$_3$)
ppm

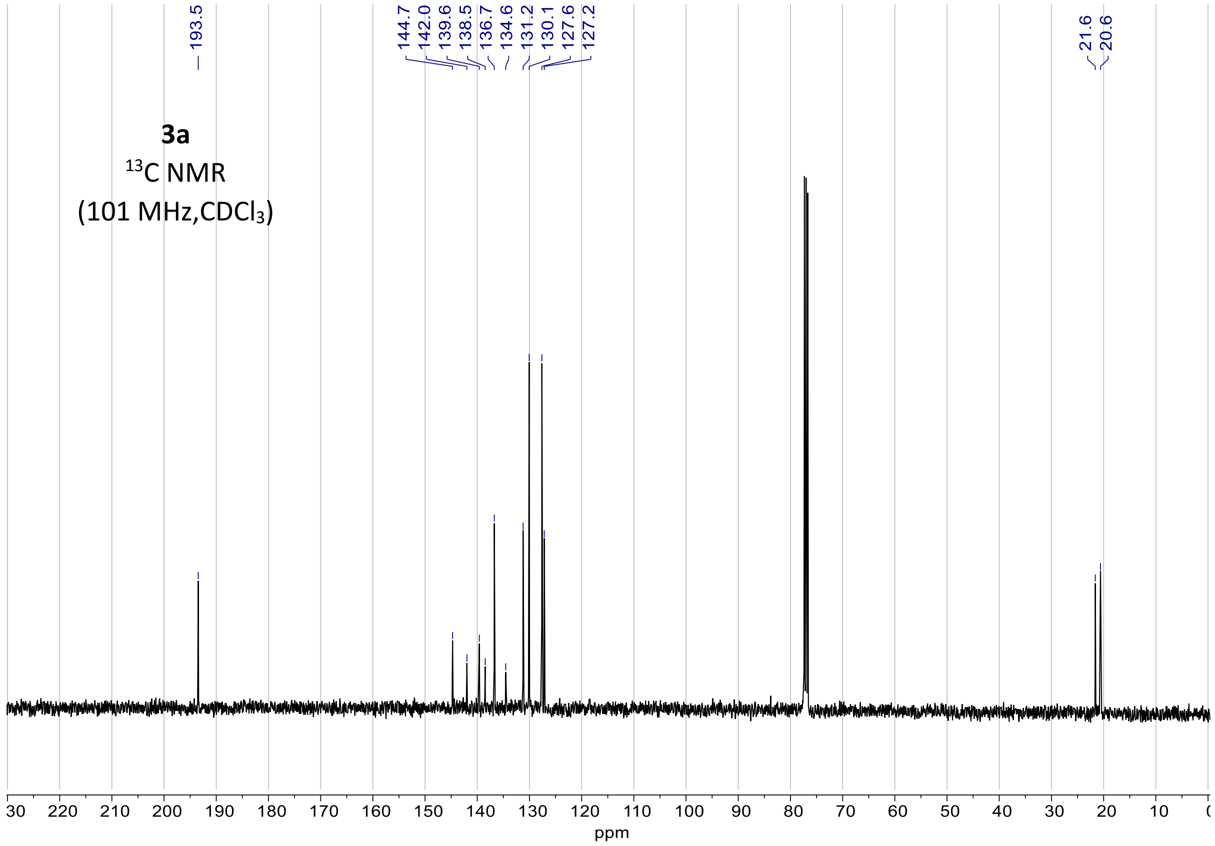
3a
^{13}C NMR
(101 MHz,CDCl$_3$)
ppm

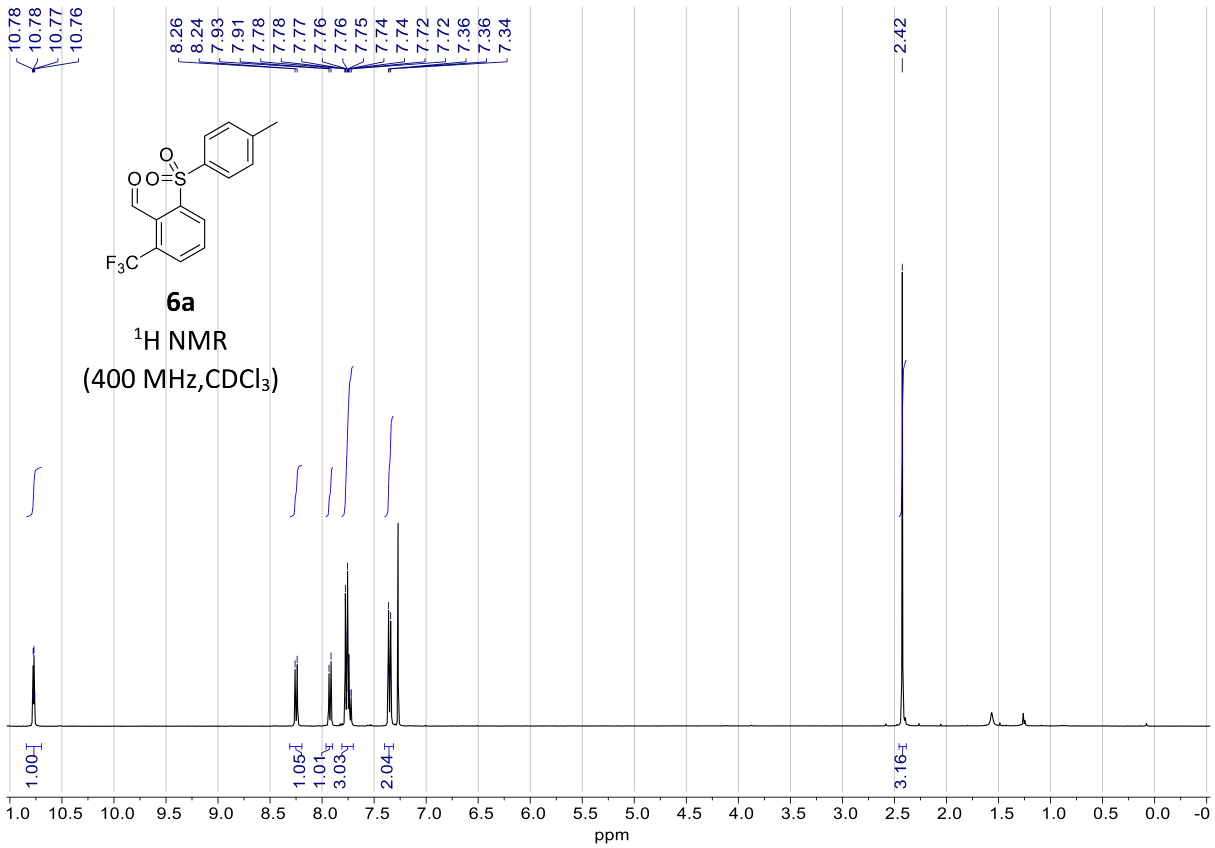
10.78
10.78
10.77
10.76
8.26
8.24
7.93
7.91
7.78
7.78
7.77
7.76
7.76
7.75
7.74
7.74
7.72
7.72
7.36
7.36
7.34
2.42
O
O
O=S
F3C
6a
1H NMR
(400 MHz,CDCl3)
1.00
1.05
1.01
3.03
2.04
3.16
ppm

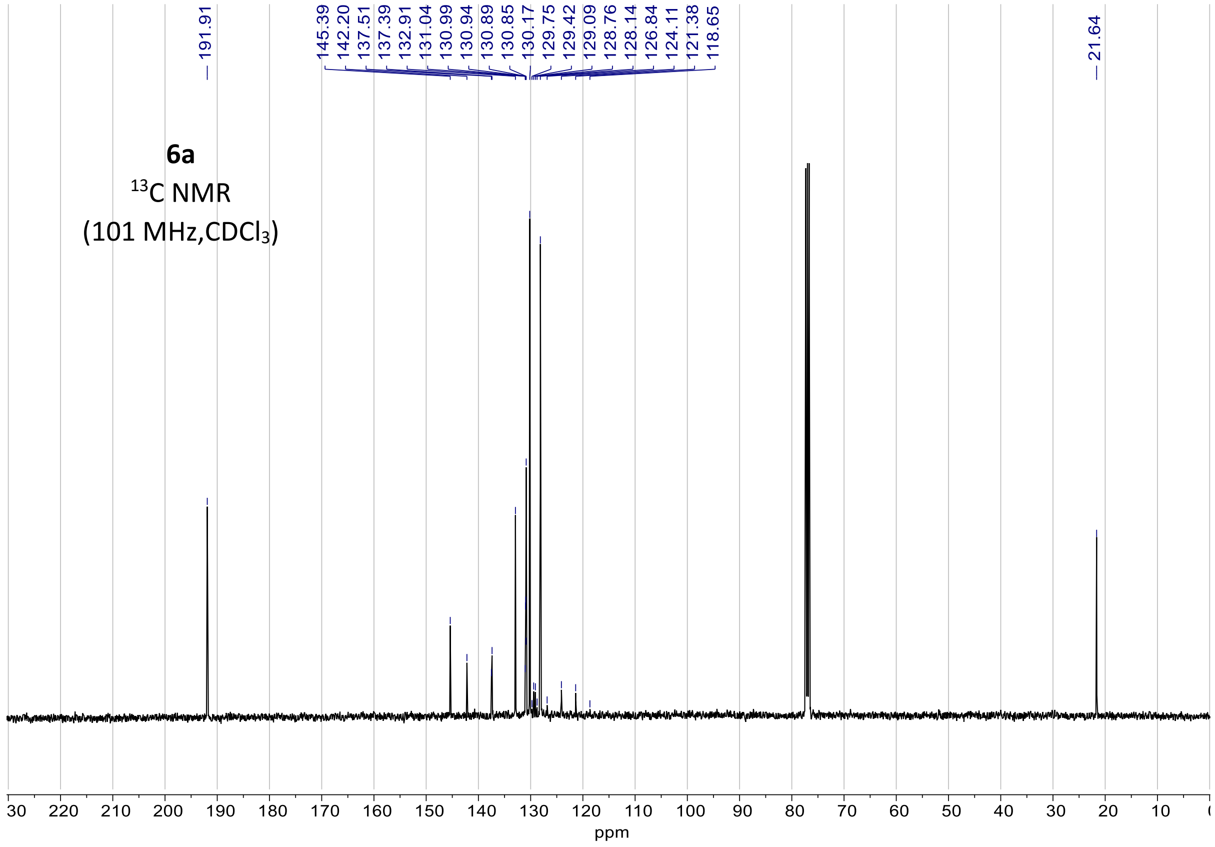
191.91
145.39
142.20
137.51
137.39
132.91
131.04
130.99
130.94
130.89
130.85
130.17
129.75
129.42
129.09
128.76
128.14
126.84
124.11
121.38
118.65
21.64
6a
13C NMR
(101 MHz,CDCl3)
ppm

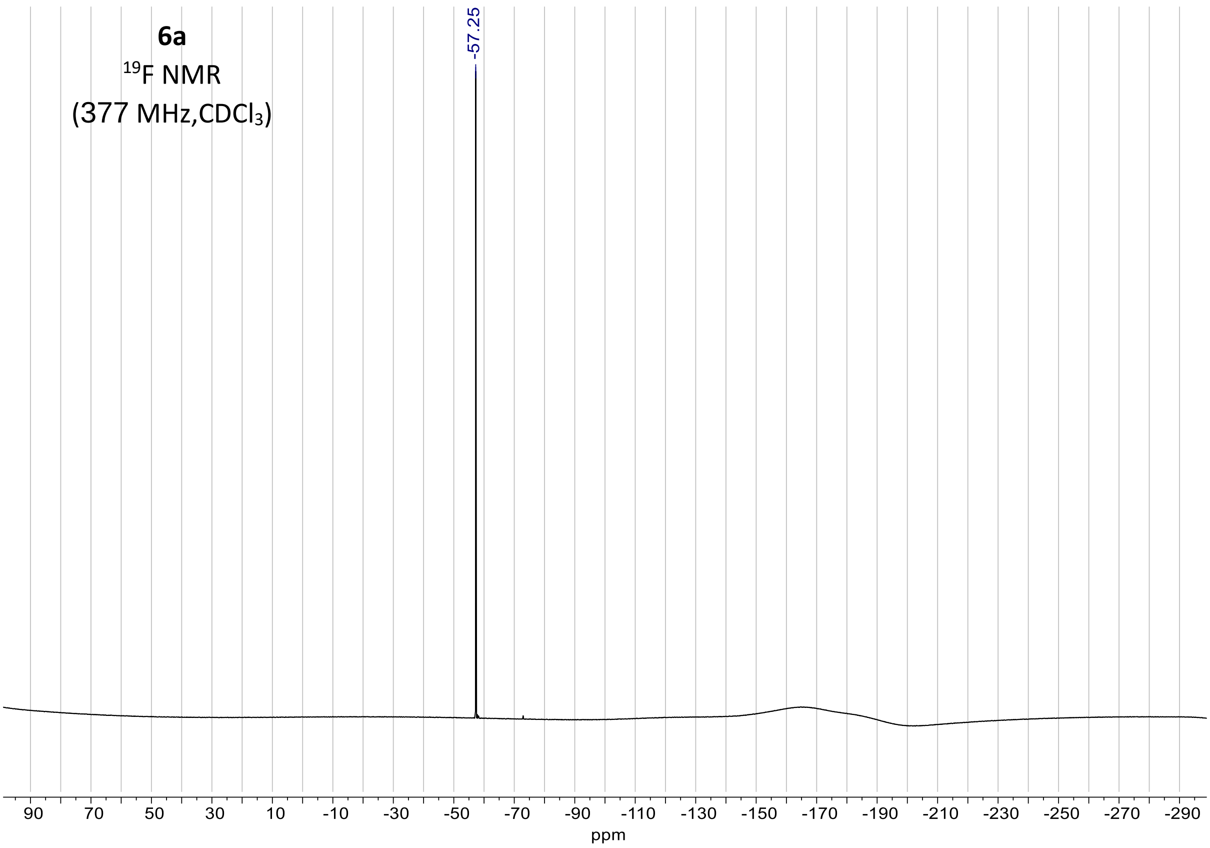
6a
19F NMR
(377 MHz,CDCl3)
-57.25
90 70 50 30 10 -10 -30 -50 -70 -90 -110 -130 -150 -170 -190 -210 -230 -250 -270 -290
ppm

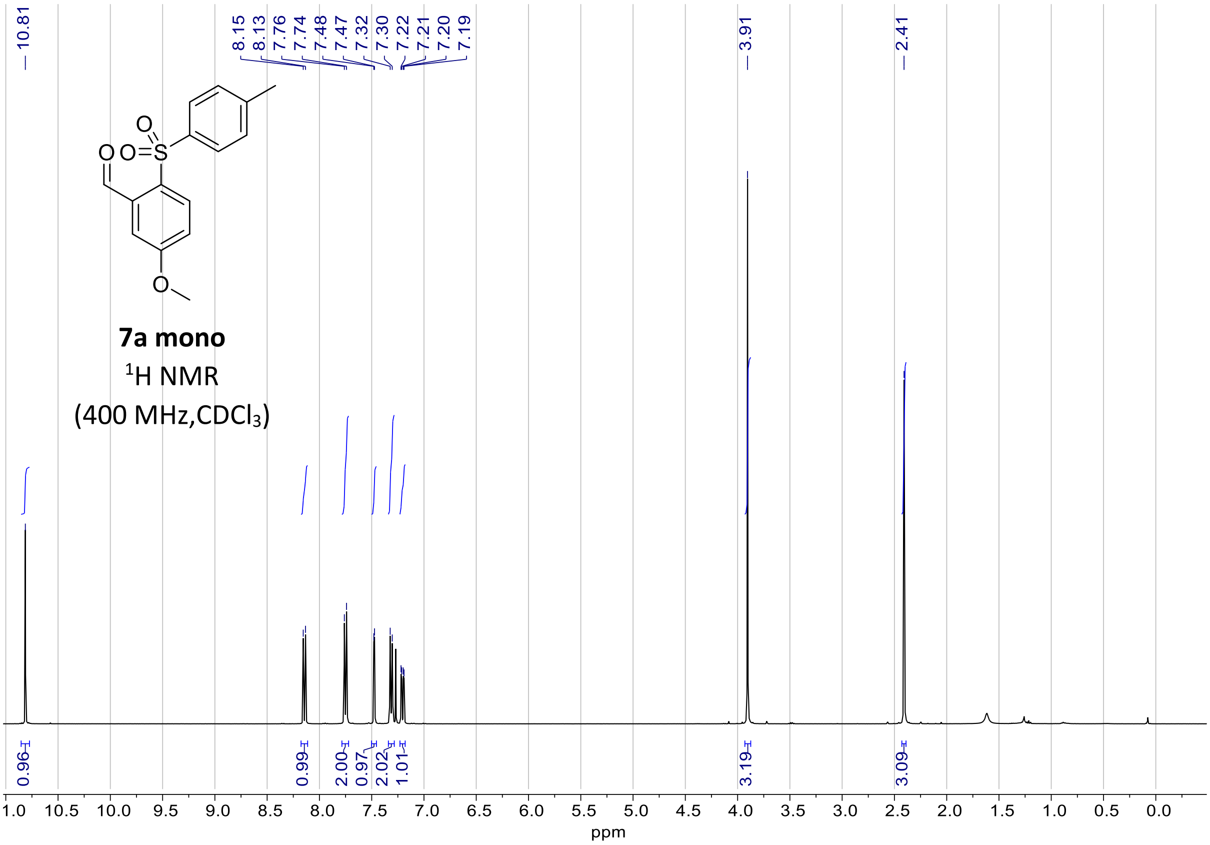
7a mono
1H NMR
(400 MHz,CDCl3)
ppm

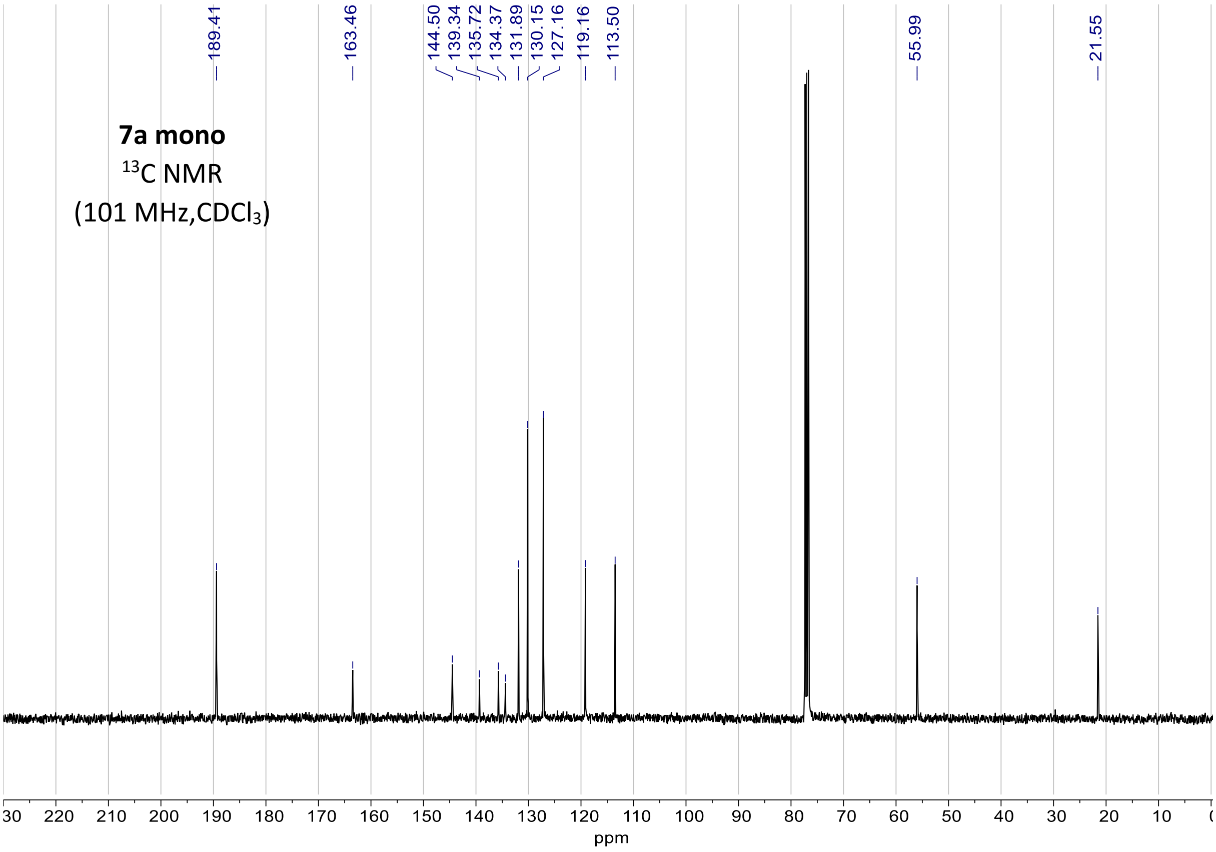
7a mono
13C NMR
(101 MHz,CDCl3)
ppm

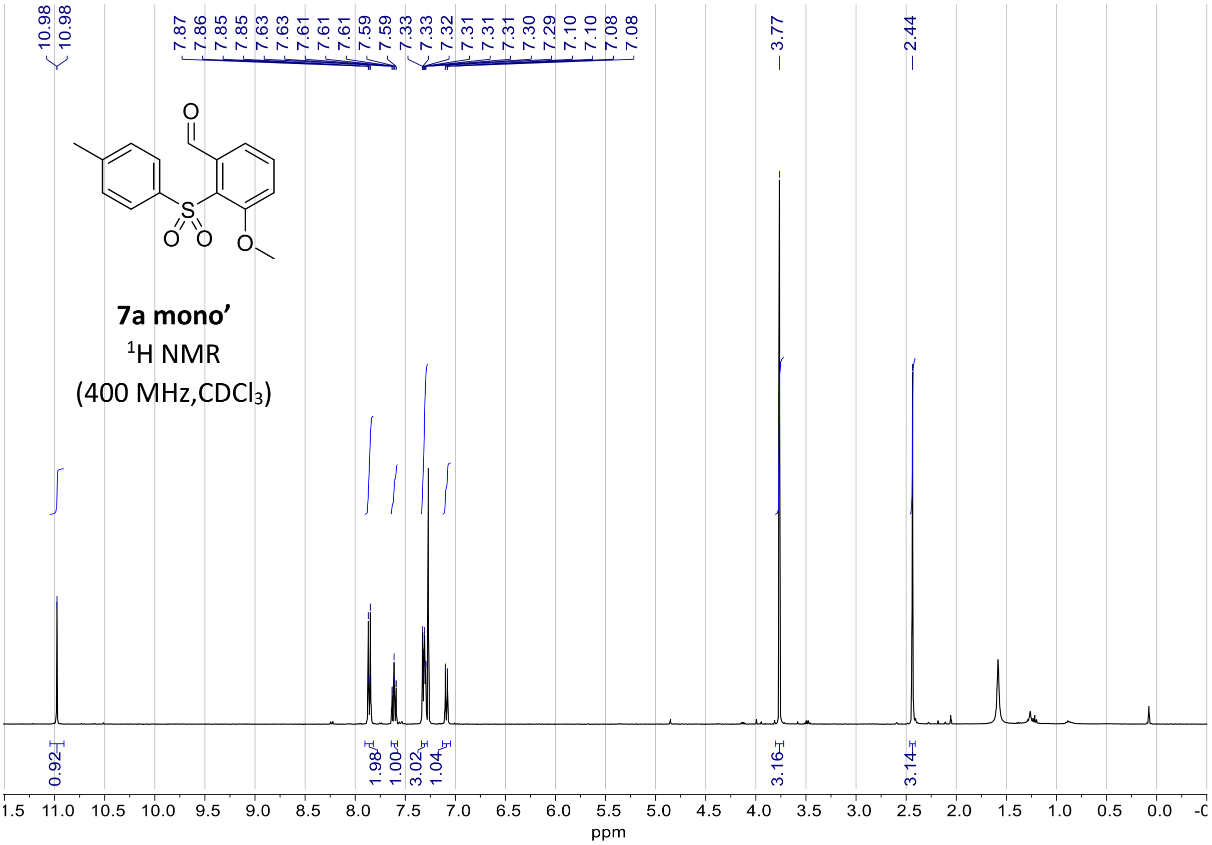
10.98
10.98
7.87
7.86
7.85
7.85
7.63
7.63
7.61
7.61
7.61
7.59
7.59
7.33
7.33
7.32
7.31
7.31
7.31
7.30
7.29
7.10
7.10
7.08
7.08
3.77
2.44
O
S
O O
O
7a mono'
1H NMR
(400 MHz,CDCl3)
0.92
1.98
1.00
3.02
1.04
3.16
3.14
ppm

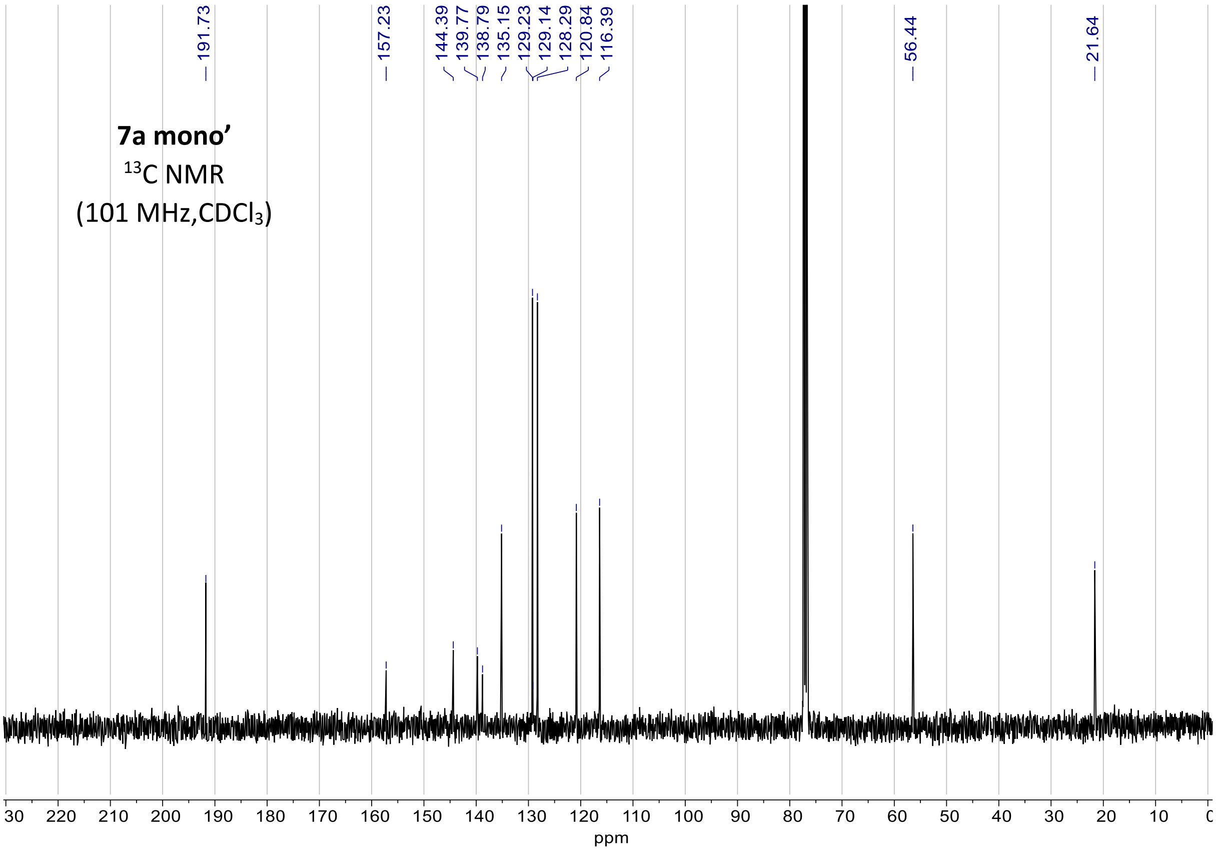
191.73
157.23
144.39
139.77
138.79
135.15
129.23
129.14
128.29
120.84
116.39
56.44
21.64
7a mono'
13C NMR
(101 MHz,CDCl3)
ppm

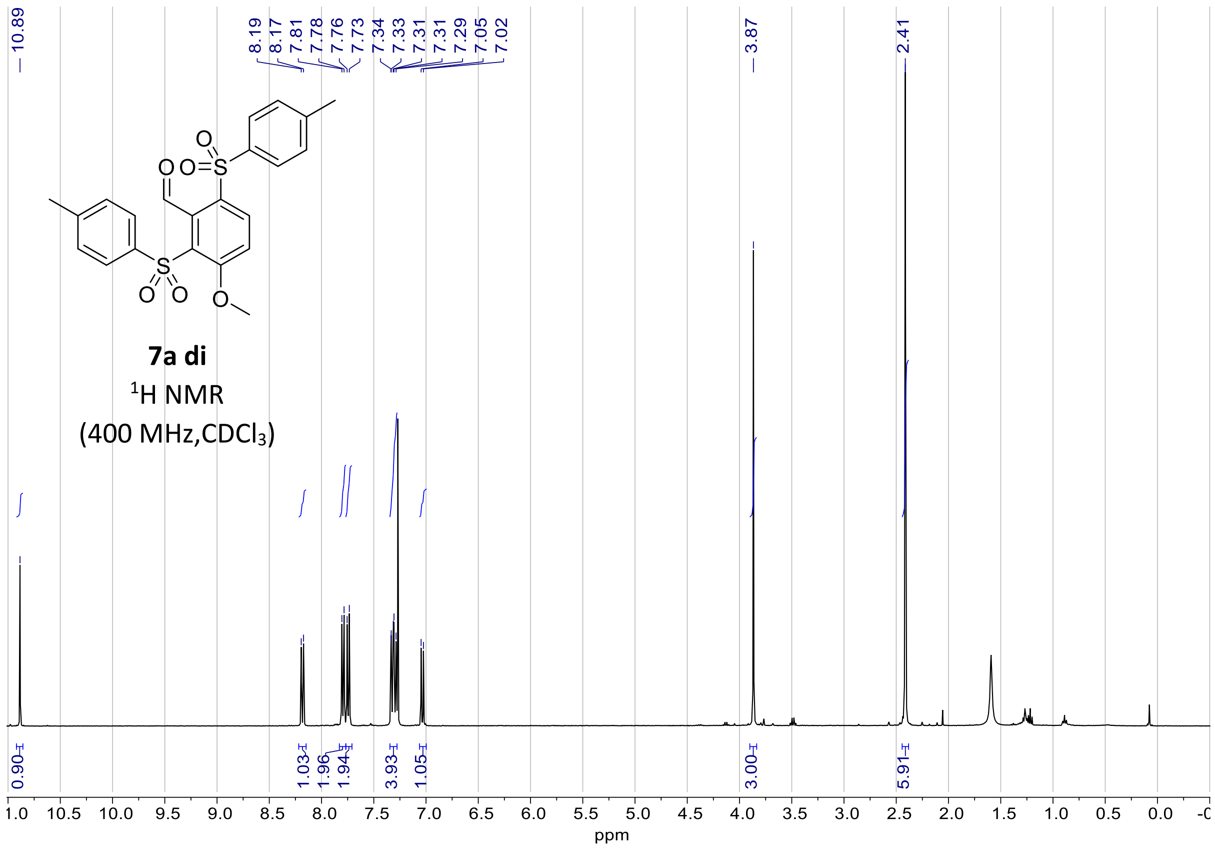
10.89
8.19
8.17
7.81
7.78
7.76
7.73
7.34
7.33
7.31
7.31
7.29
7.05
7.02
3.87
2.41
7a di
^{1}H NMR
(400 MHz,CDCl$_3$)
0.90
1.03
1.96
1.94
3.93
1.05
3.00
5.91
ppm

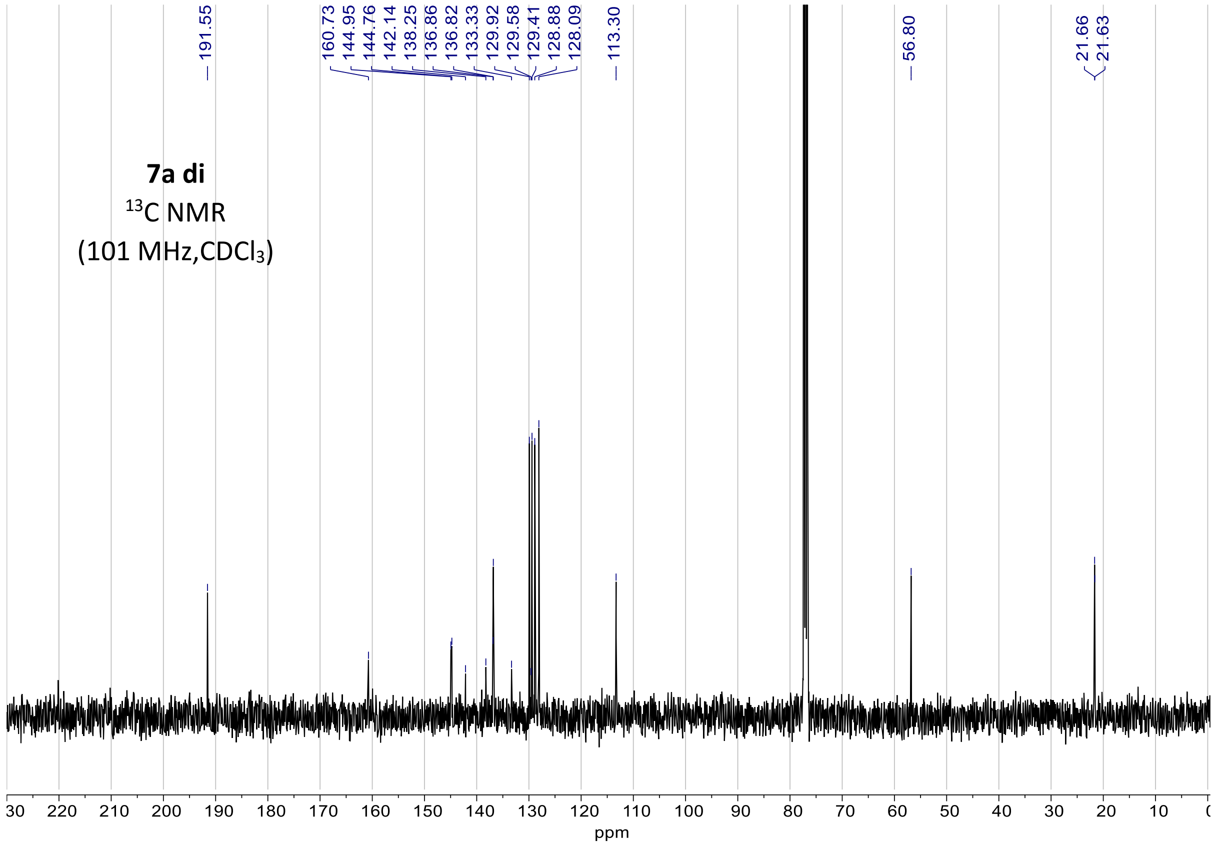
191.55
160.73
144.95
144.76
142.14
138.25
136.86
136.82
133.33
129.92
129.58
129.41
128.88
128.09
113.30
56.80
21.66
21.63
7a di
^{13}C NMR
(101 MHz,CDCl$_3$)
ppm

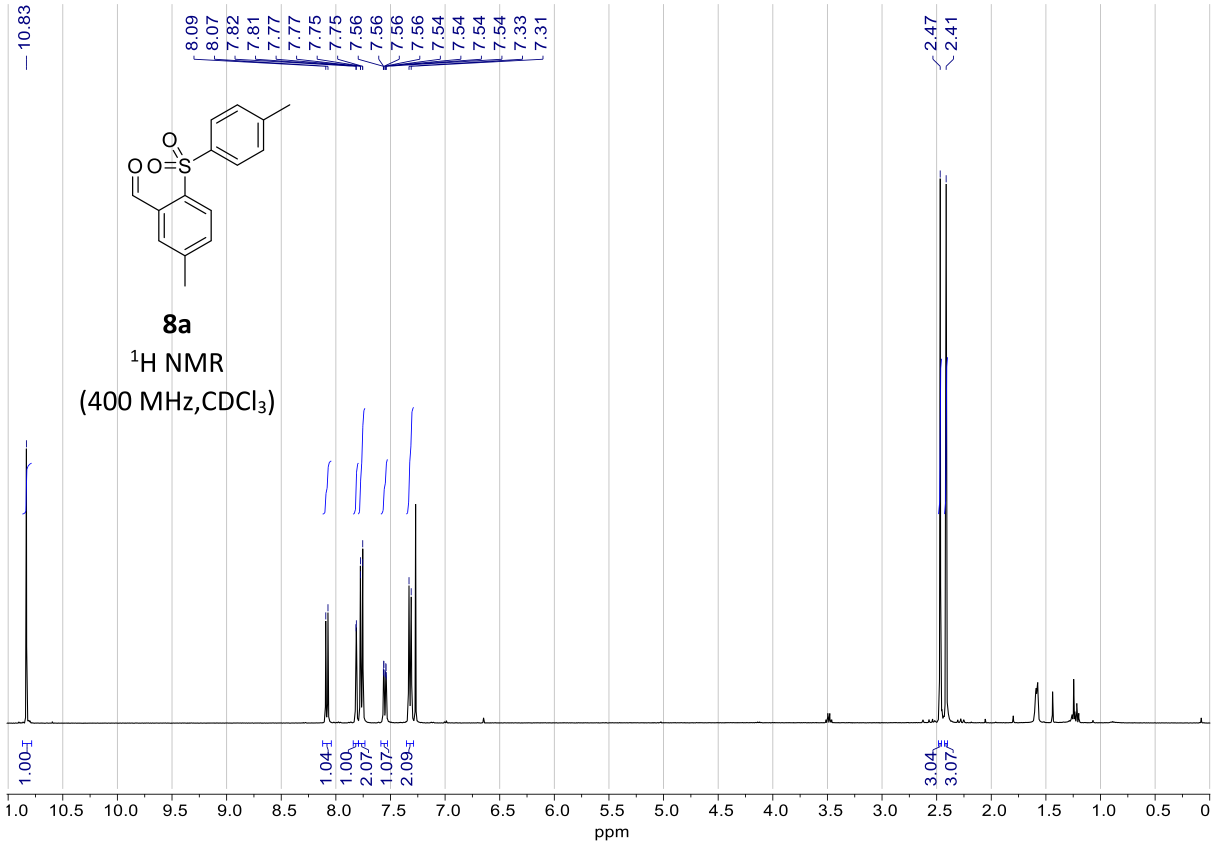
10.83
8.09
8.07
7.82
7.81
7.77
7.77
7.75
7.75
7.56
7.56
7.56
7.56
7.54
7.54
7.54
7.54
7.33
7.31
2.47
2.41
8a
^{1}H NMR
(400 MHz,CDCl$_3$)
1.00
1.04
1.00
2.07
1.07
2.09
3.04
3.07
ppm

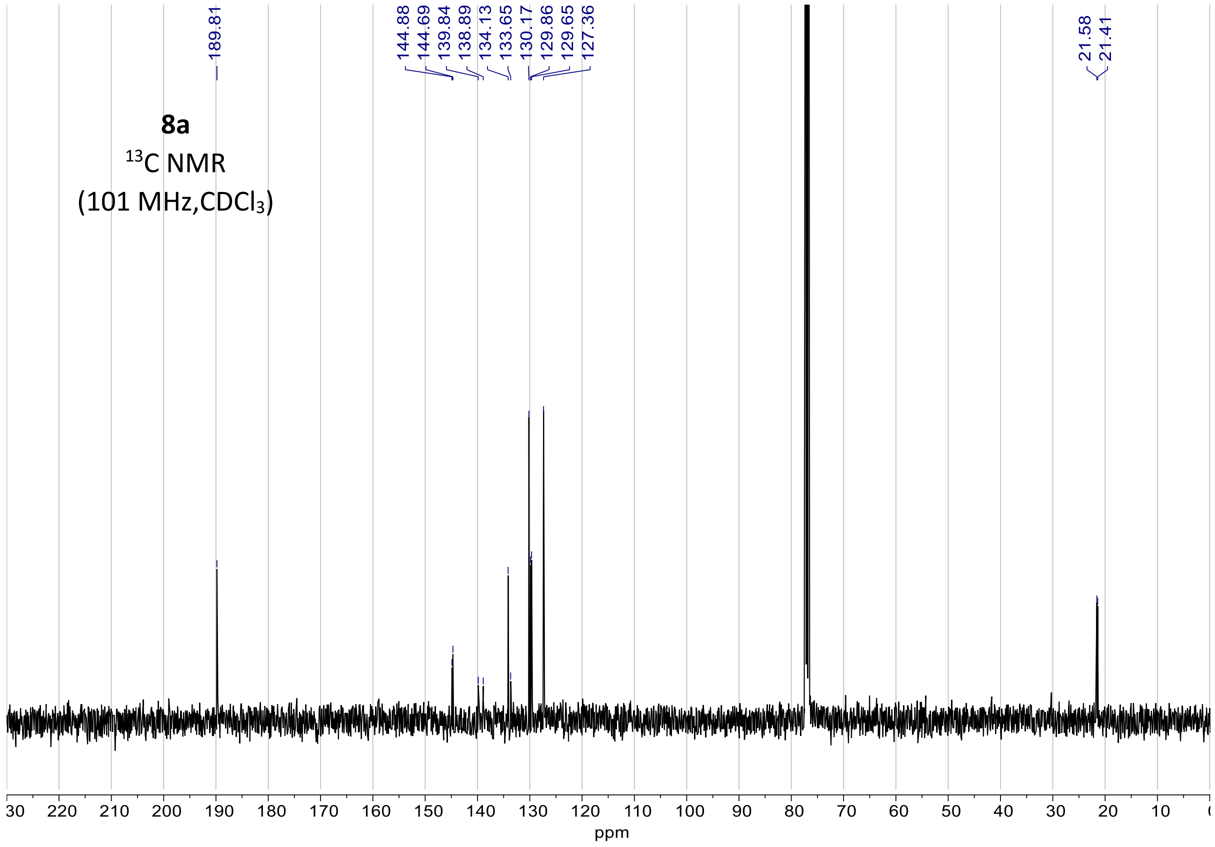
189.81
144.88
144.69
139.84
138.89
134.13
133.65
130.17
129.86
129.65
127.36
21.58
21.41
8a
^{13}C NMR
(101 MHz,CDCl$_3$)
ppm

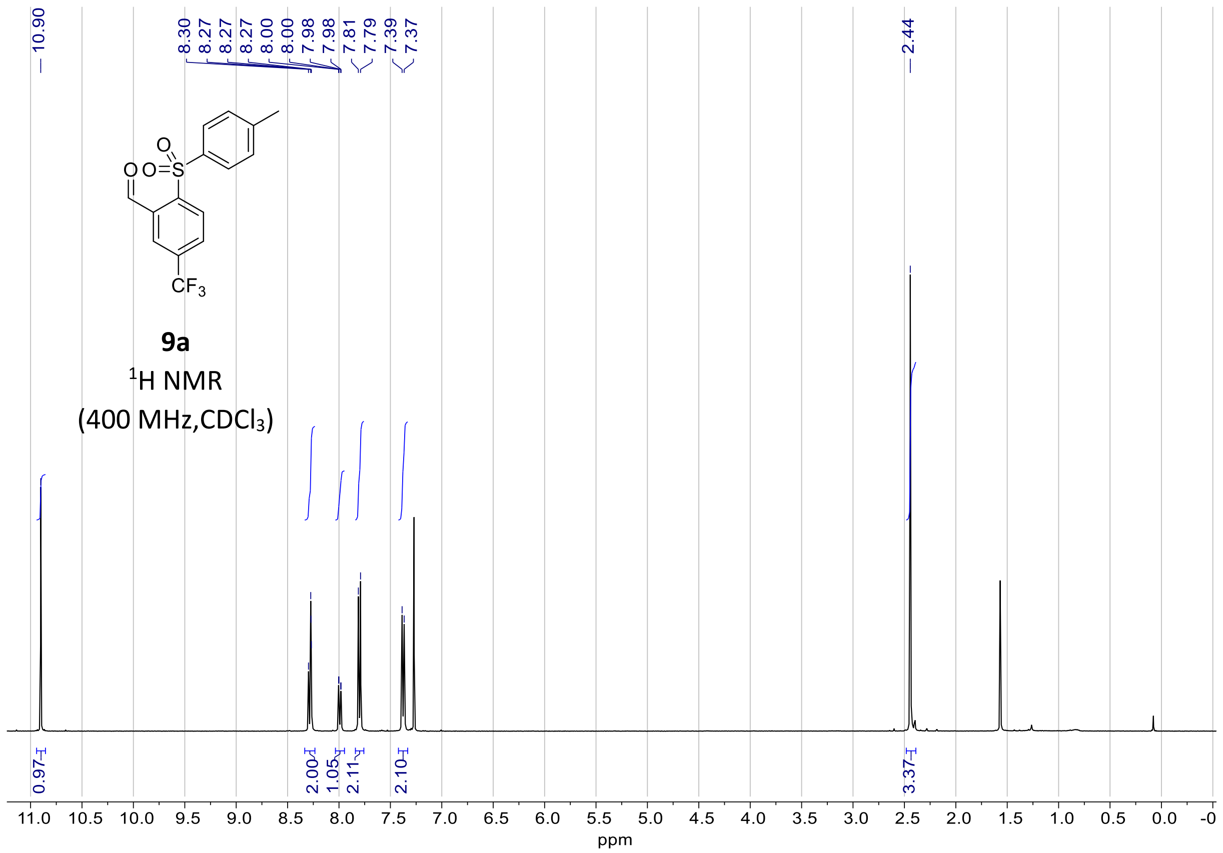
10.90
8.30
8.27
8.27
8.27
8.00
8.00
7.98
7.98
7.81
7.79
7.39
7.37
2.44
O
O
O=S
CF3
9a
1H NMR
(400 MHz,CDCl3)
0.97
2.00
1.05
2.11
2.10
3.37
ppm

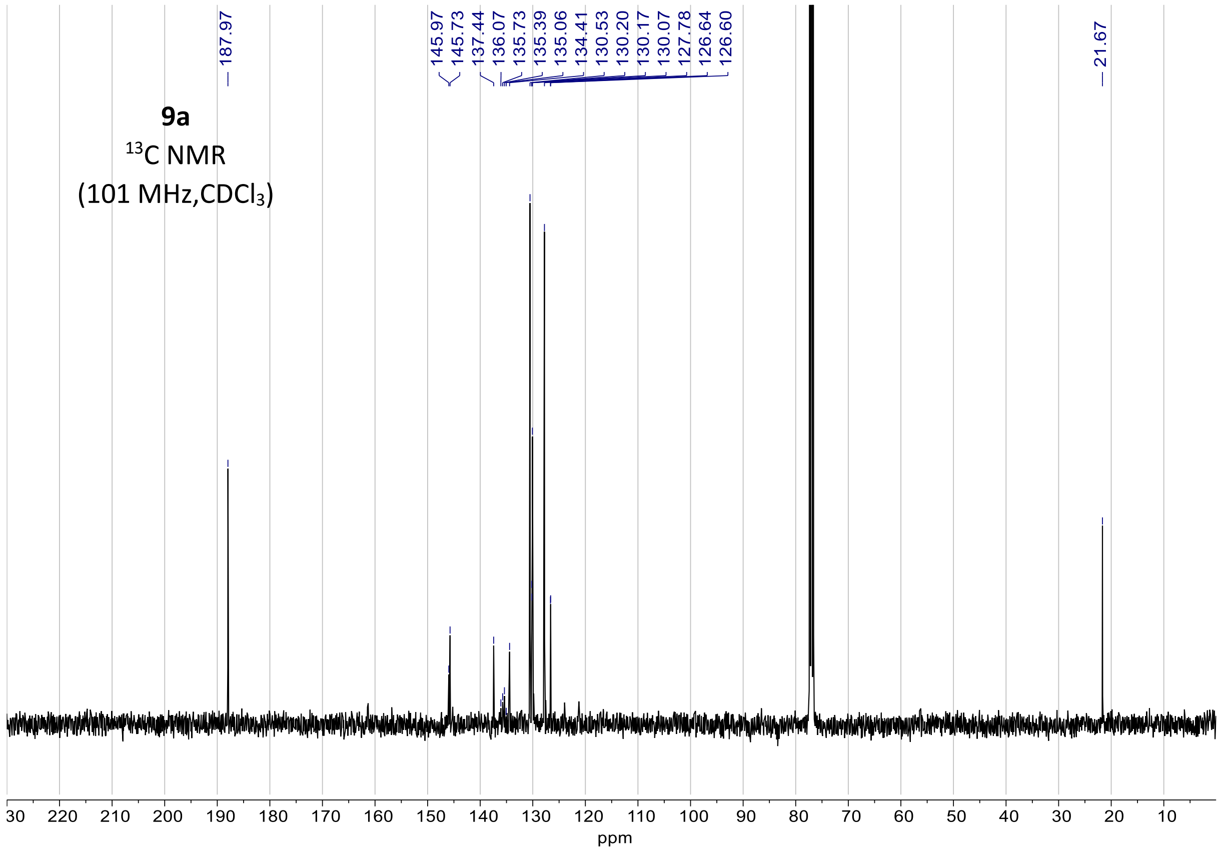
187.97
145.97
145.73
137.44
136.07
135.73
135.39
135.06
134.41
130.53
130.20
130.17
130.07
127.78
126.64
126.60
21.67
9a
13C NMR
(101 MHz,CDCl3)
ppm

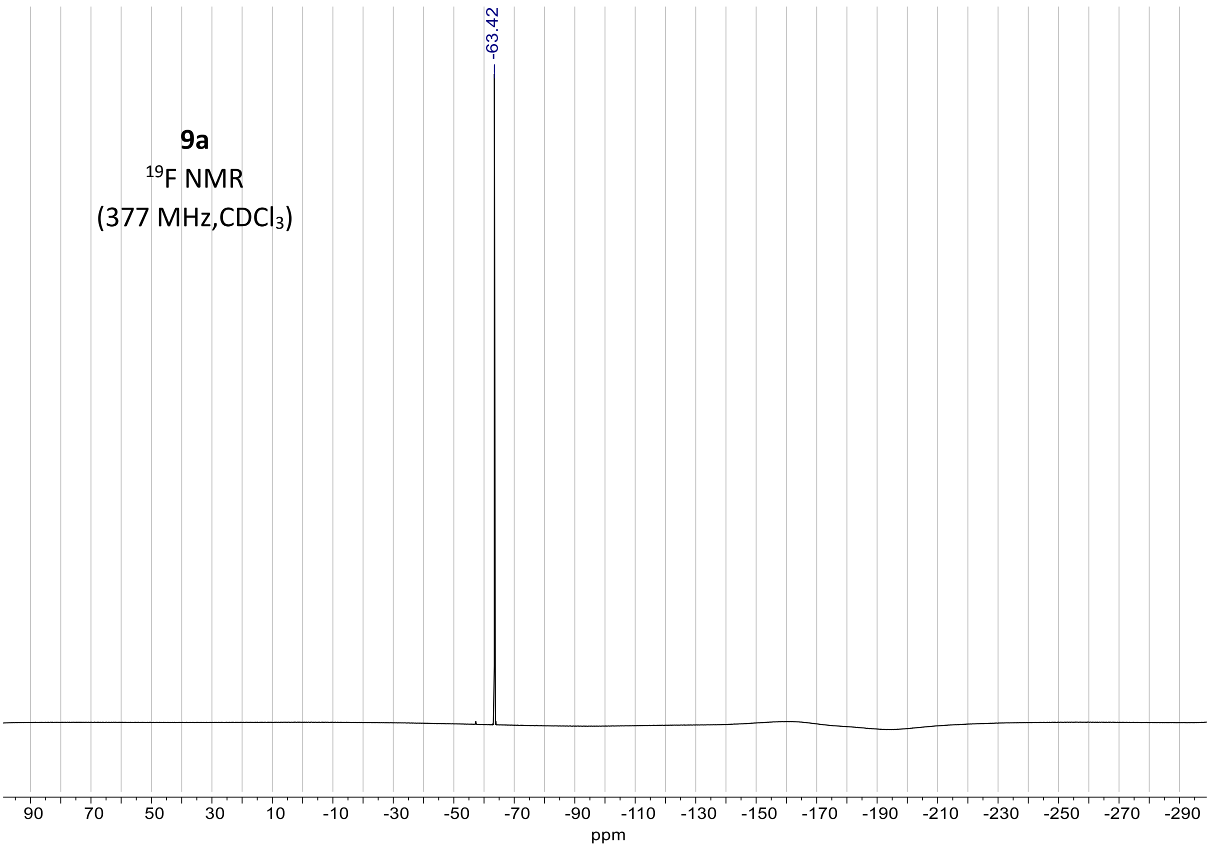

-63.42
9a
19F NMR
(377 MHz,CDCl3)
90 70 50 30 10 -10 -30 -50 -70 -90 -110 -130 -150 -170 -190 -210 -230 -250 -270 -290
ppm

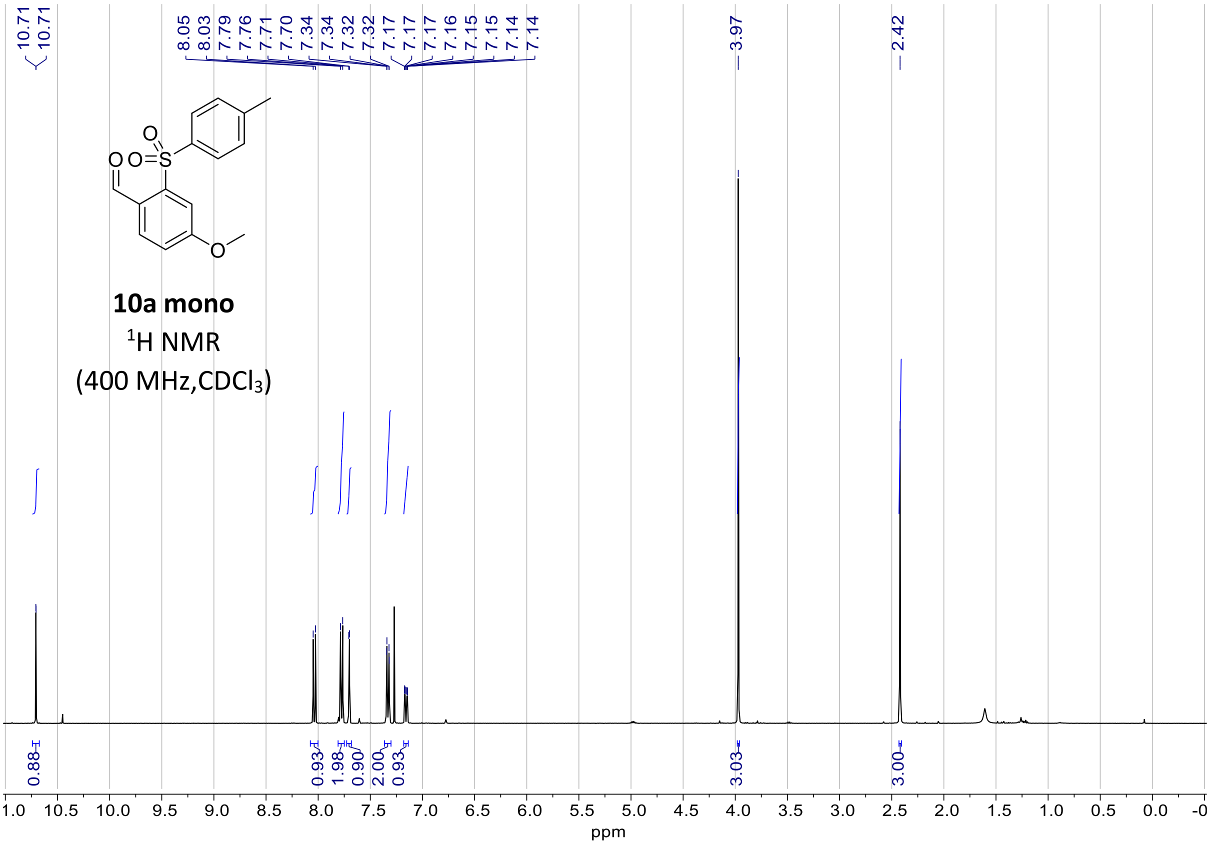
10a mono
1H NMR
(400 MHz,CDCl3)
ppm

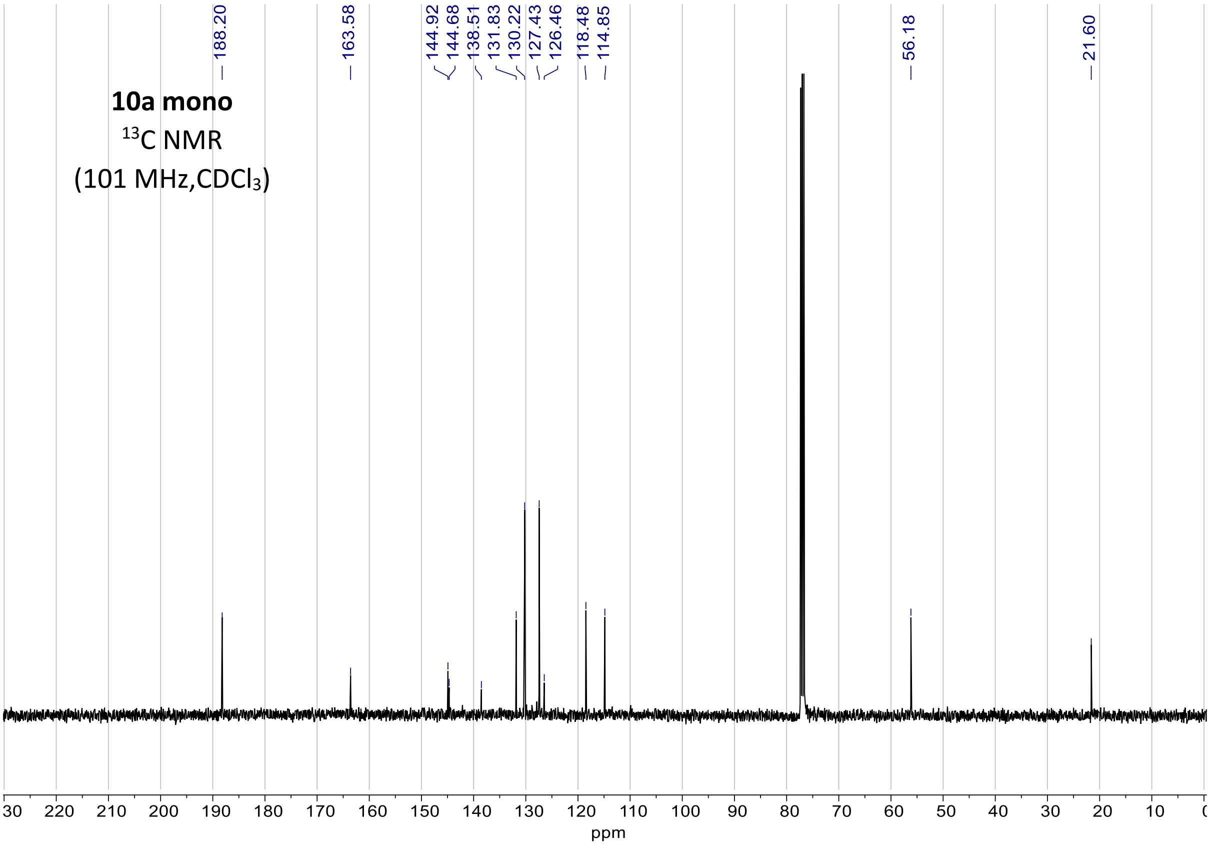
10a mono
13C NMR
(101 MHz,CDCl3)
ppm

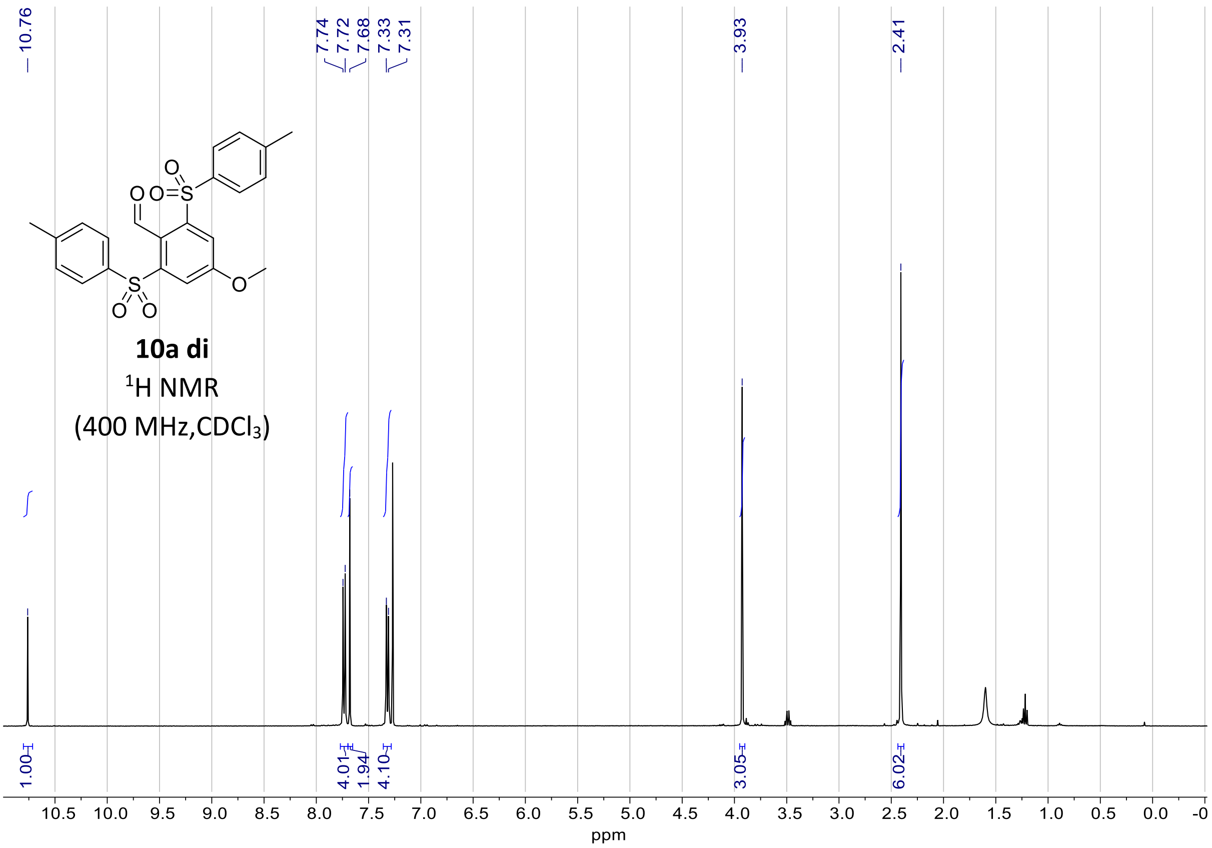
10a di
^{1}H NMR
(400 MHz,CDCl$_3$)
10.76
7.74
7.72
7.68
7.33
7.31
3.93
2.41
1.00
4.01
1.94
4.10
3.05
6.02
ppm

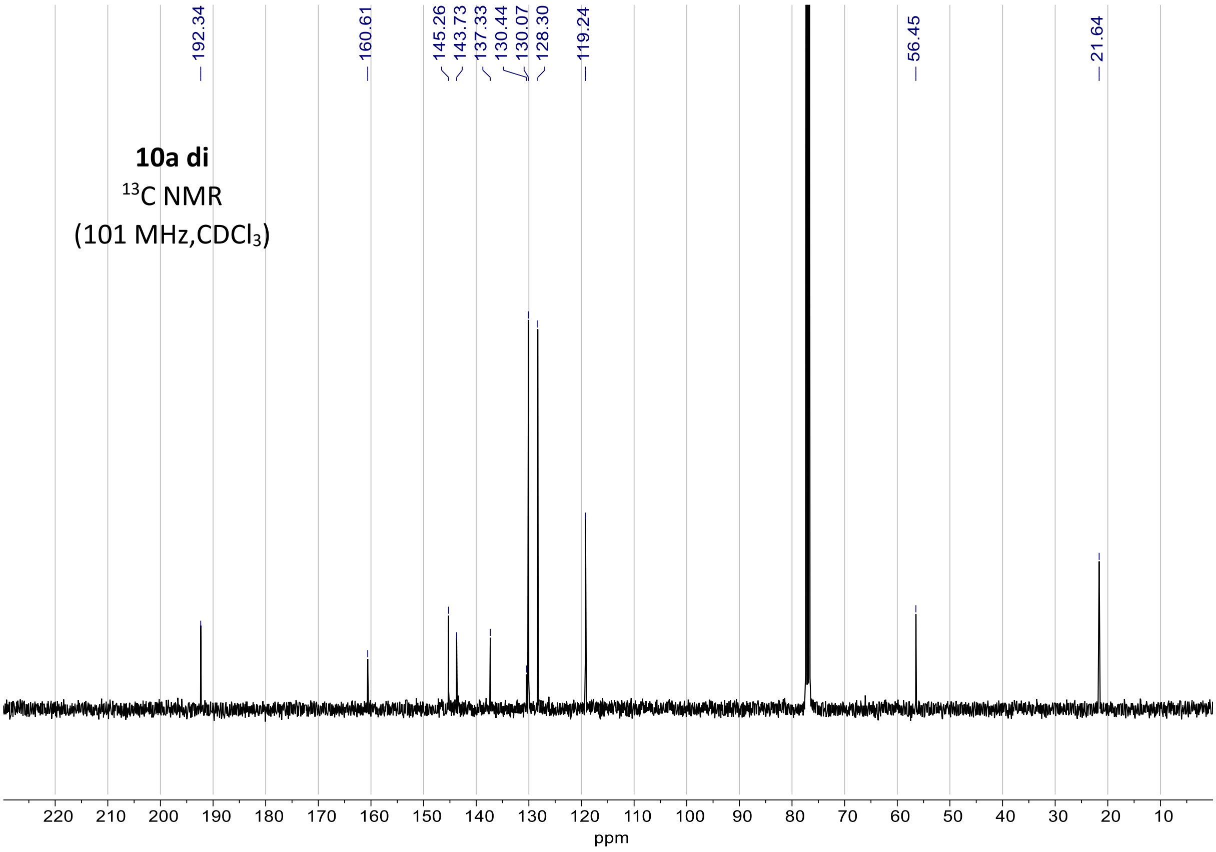
10a di
^{13}C NMR
(101 MHz,CDCl$_3$)
192.34
160.61
145.26
143.73
137.33
130.44
130.07
128.30
119.24
56.45
21.64
ppm

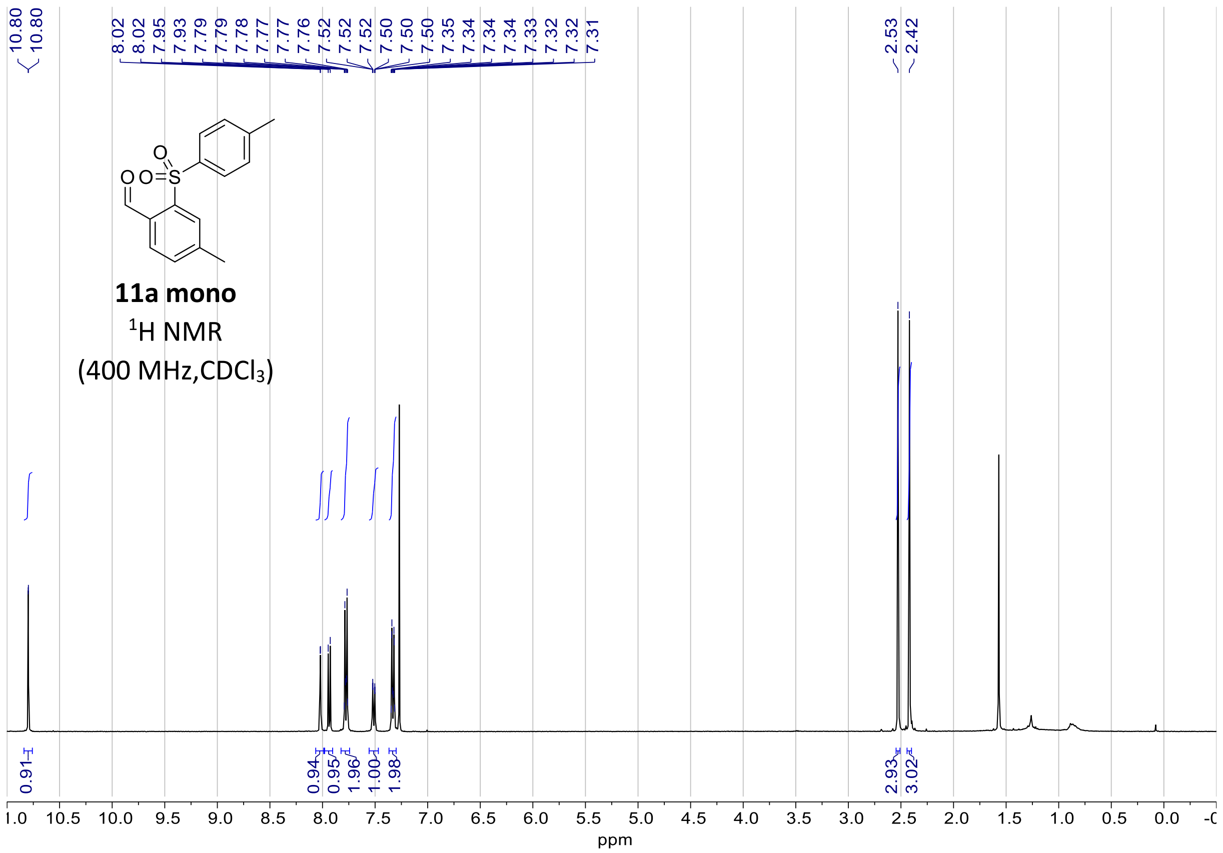
11a mono
^{1}H NMR
(400 MHz,CDCl$_3$)
ppm

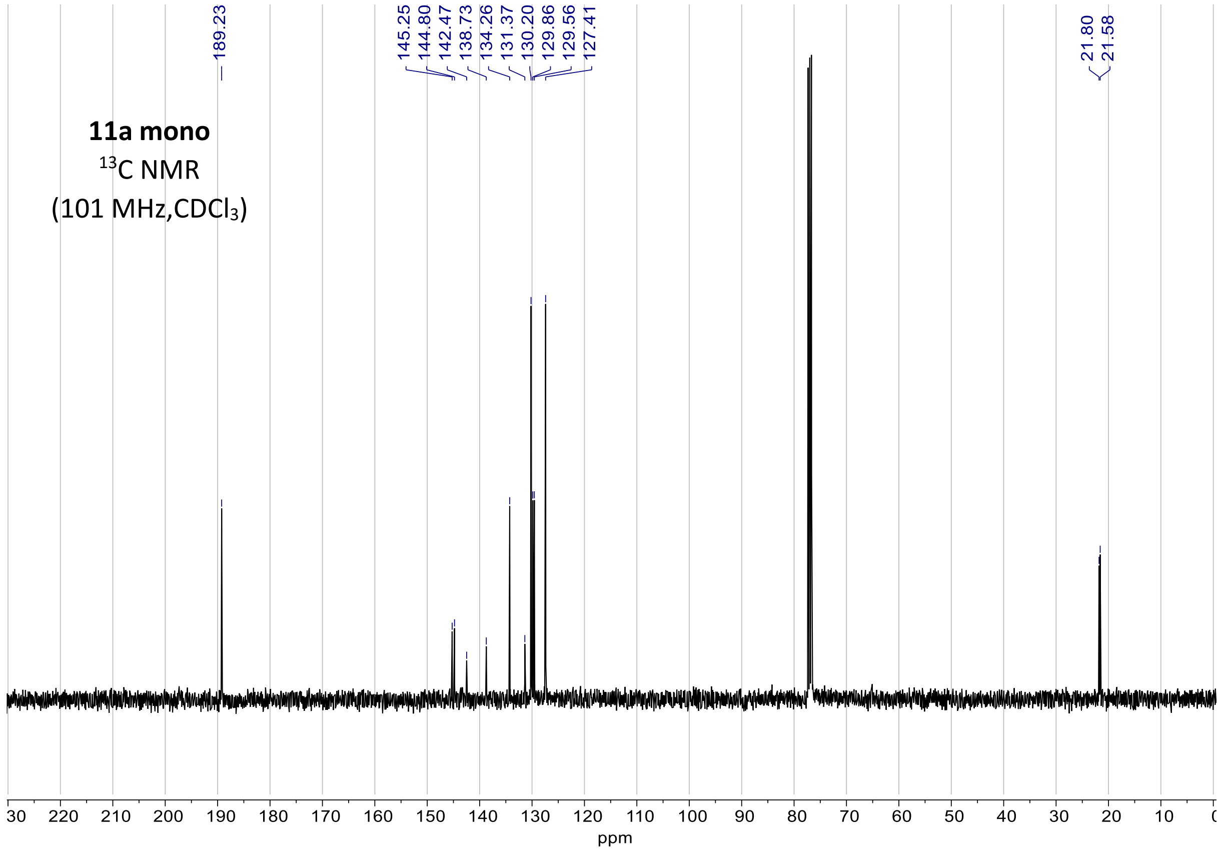
11a mono
^{13}C NMR
(101 MHz,CDCl$_3$)
ppm

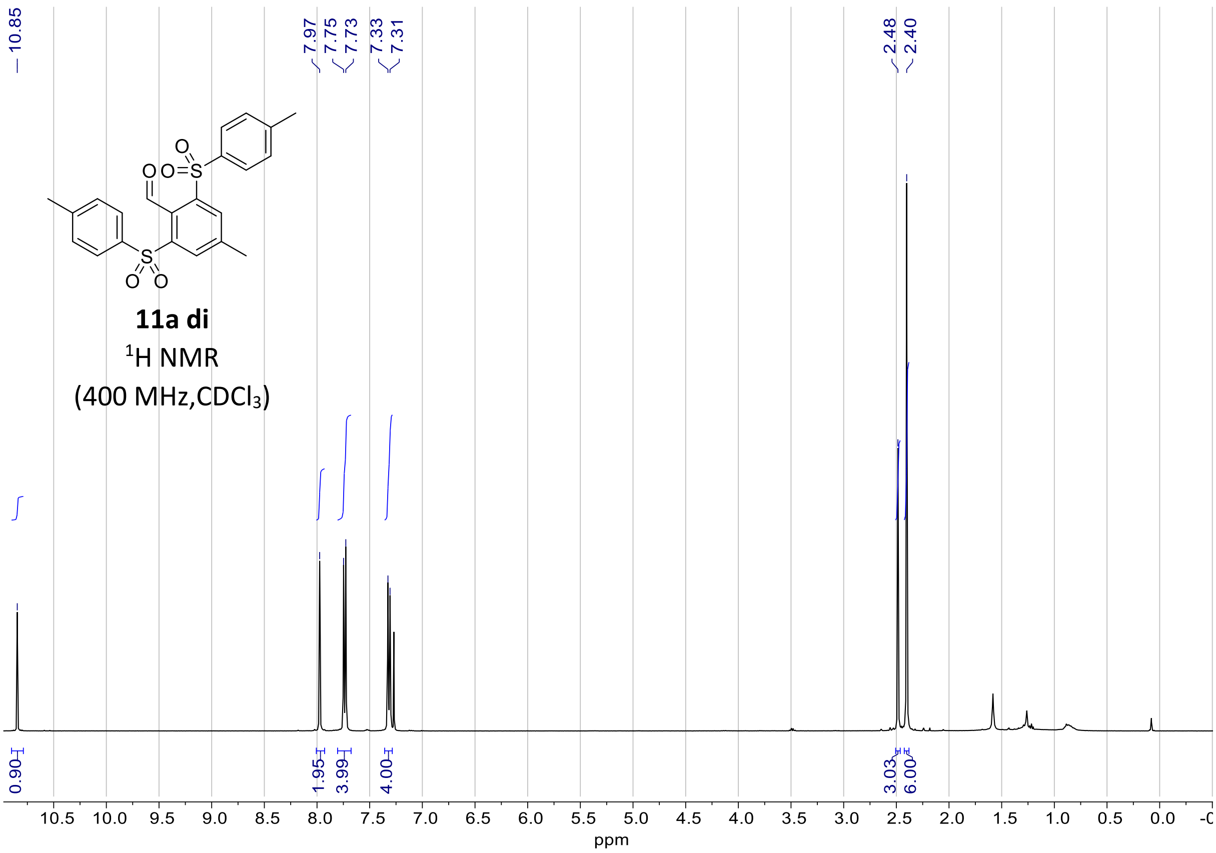
10.85
7.97
7.75
7.73
7.33
7.31
2.48
2.40
11a di
^{1}H NMR
(400 MHz,CDCl$_3$)
0.90
1.95
3.99
4.00
3.03
6.00
ppm

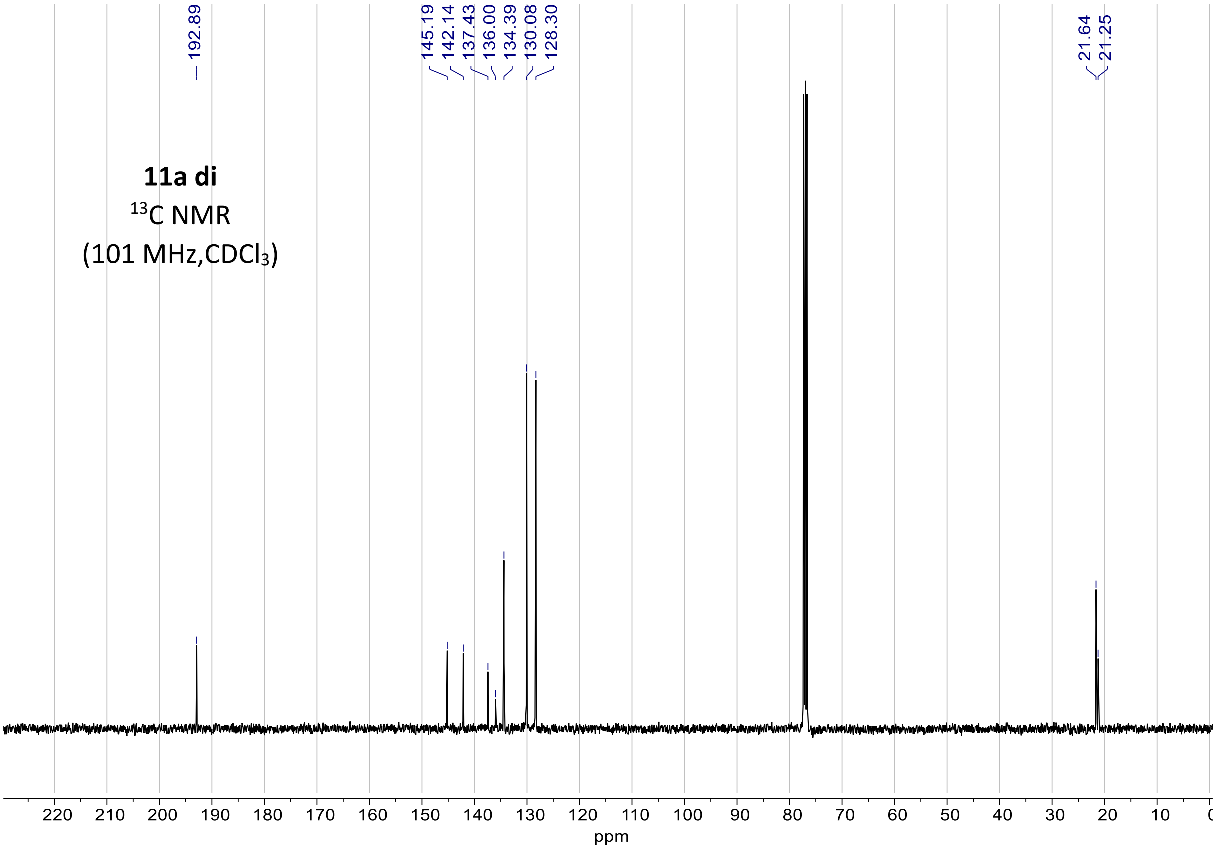
192.89
145.19
142.14
137.43
136.00
134.39
130.08
128.30
21.64
21.25
11a di
^{13}C NMR
(101 MHz,CDCl$_3$)
ppm

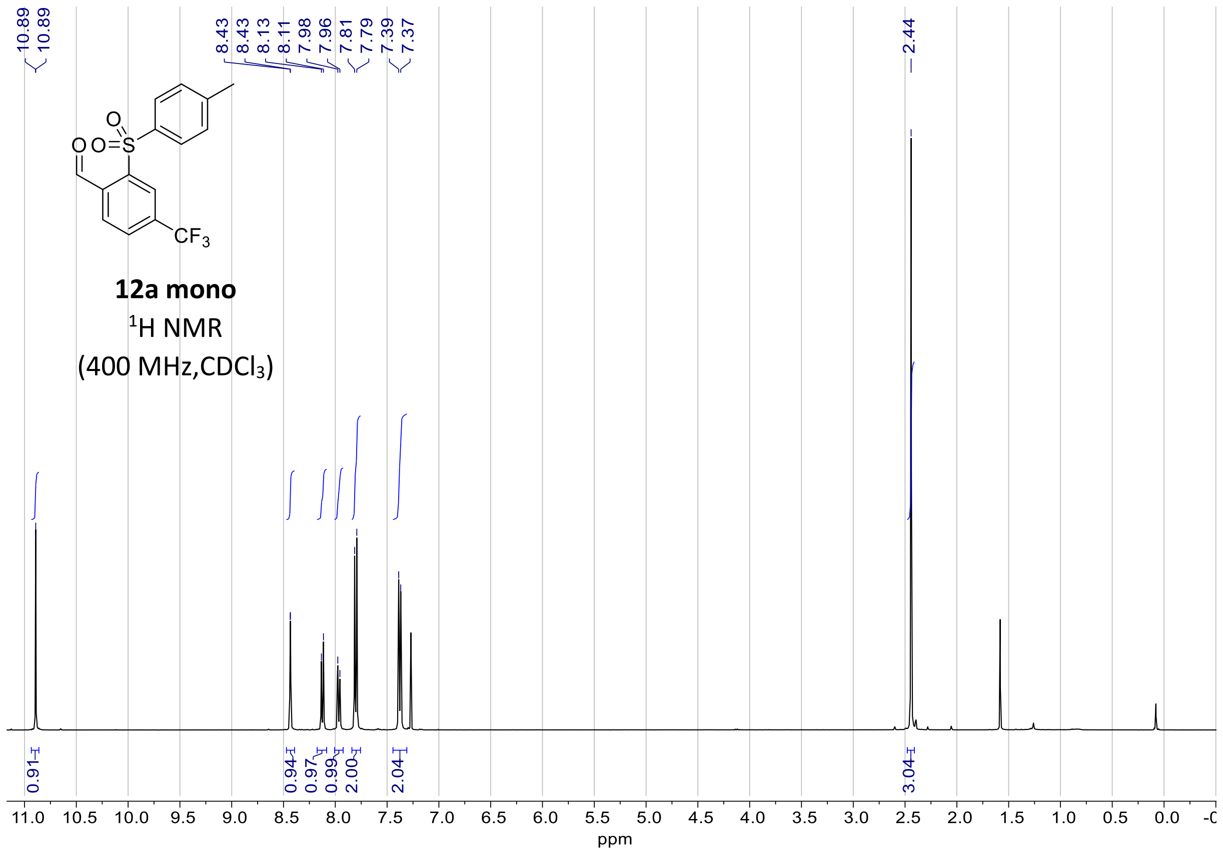
10.89
10.89
8.43
8.43
8.13
8.11
7.98
7.96
7.81
7.79
7.39
7.37
2.44
CF3
12a mono
1H NMR
(400 MHz,CDCl3)
0.91
0.94
0.97
0.99
2.00
2.04
3.04
ppm

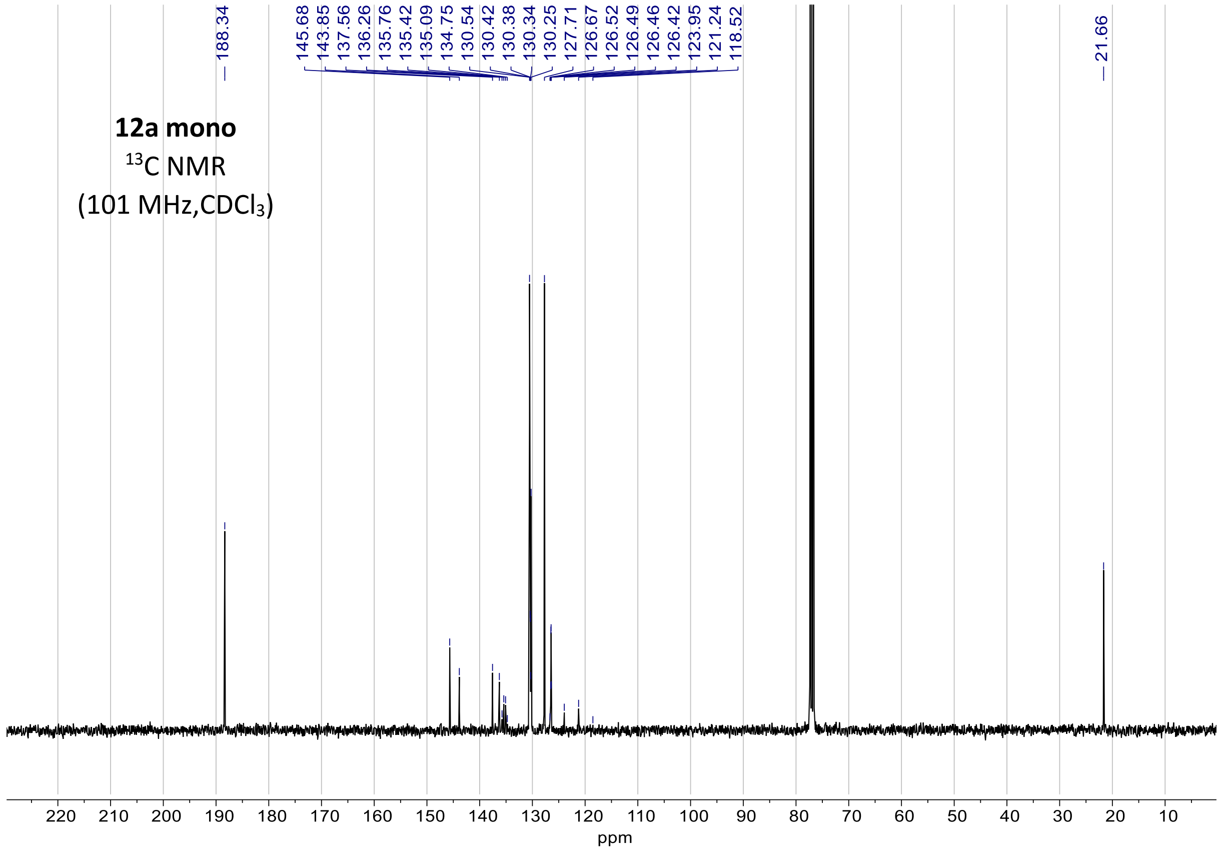
188.34
145.68
143.85
137.56
136.26
135.76
135.42
135.09
134.75
130.54
130.42
130.38
130.34
130.25
127.71
126.67
126.52
126.49
126.46
126.42
123.95
121.24
118.52
21.66
12a mono
13C NMR
(101 MHz,CDCl3)
ppm

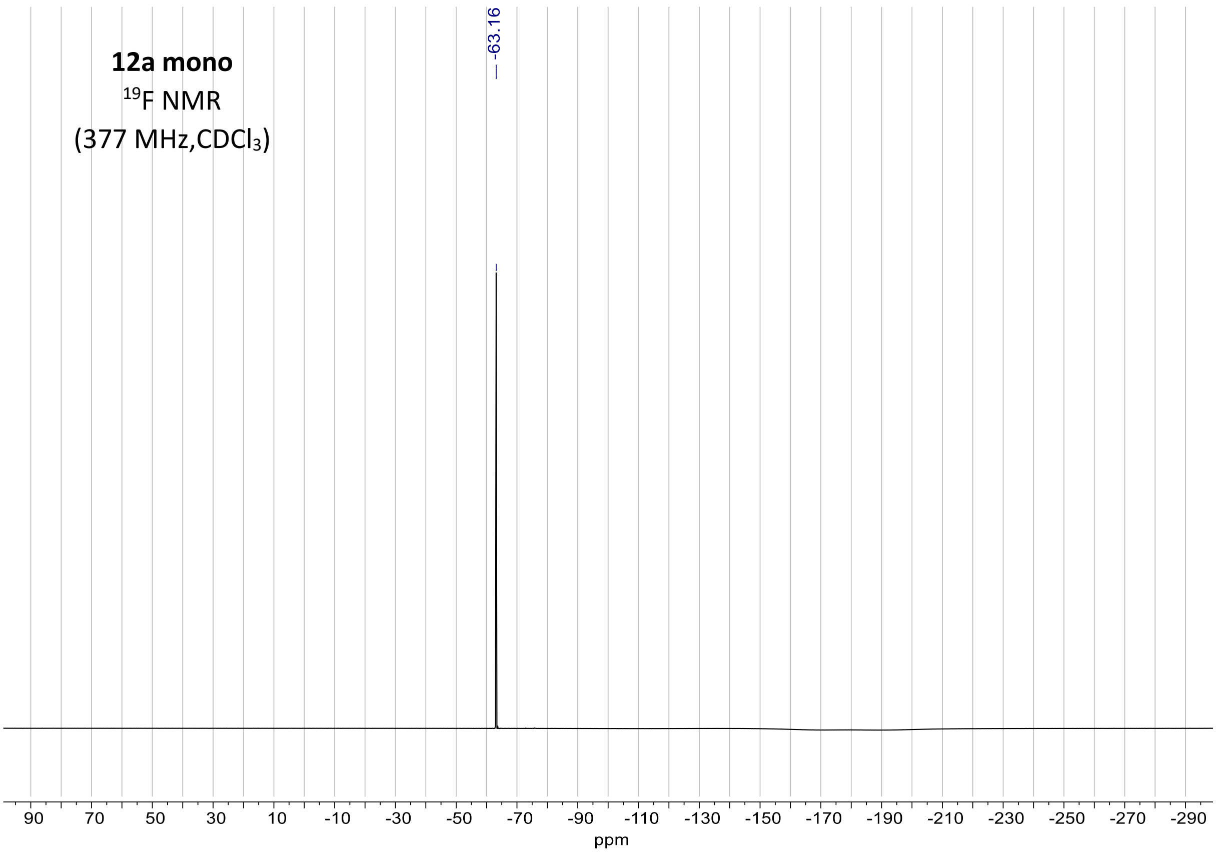
12a mono
^{19}F NMR
(377 MHz,CDCl$_3$)
-63.16
90 70 50 30 10 -10 -30 -50 -70 -90 -110 -130 -150 -170 -190 -210 -230 -250 -270 -290
ppm

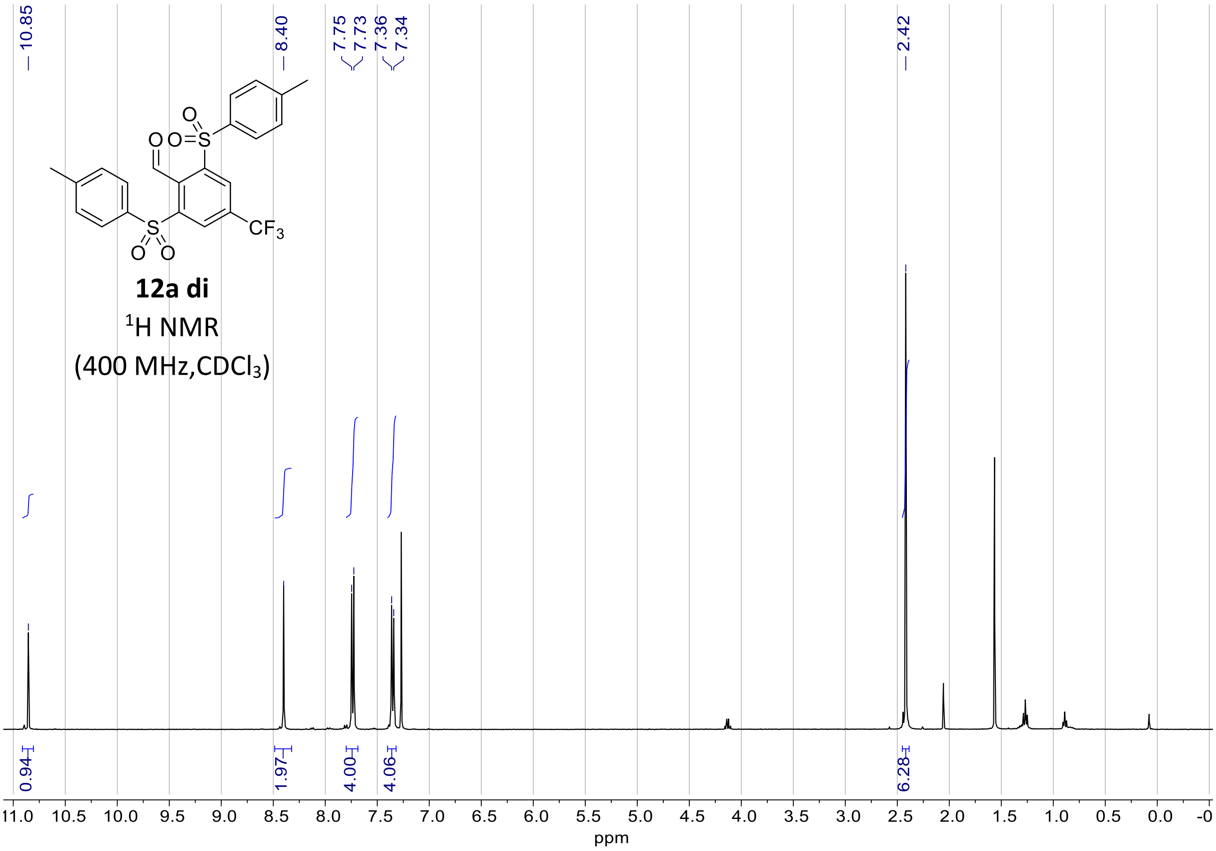

12a di
^{1}H NMR
(400 MHz,$CDCl_3$)

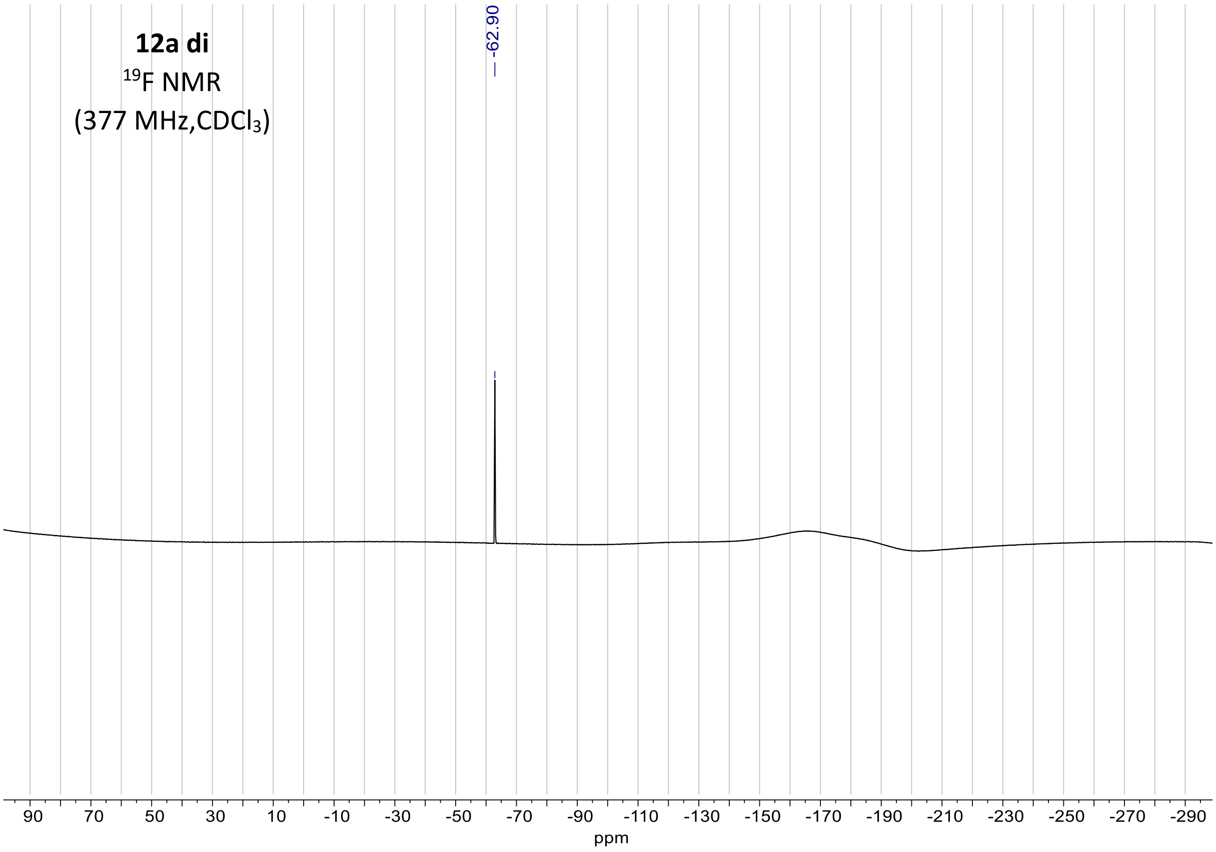

12a di
^{19}F NMR
(377 MHz,$CDCl_3$)

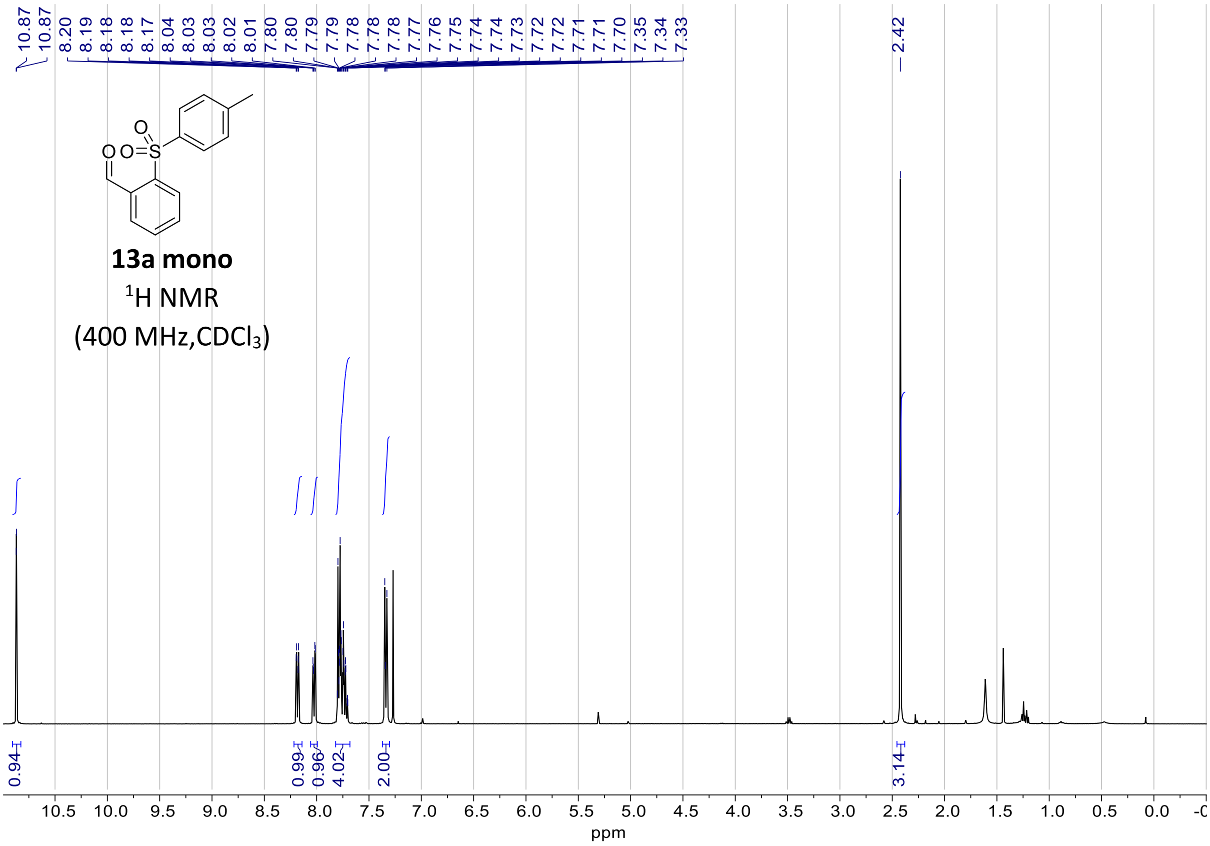
10.87 10.87 8.20 8.19 8.18 8.18 8.17 8.04 8.03 8.03 8.02 8.01 7.80 7.79 7.79 7.78 7.78 7.78 7.77 7.76 7.75 7.74 7.74 7.73 7.72 7.72 7.71 7.71 7.70 7.35 7.34 7.33
2.42
O
O=S
O
13a mono
^{1}H NMR
(400 MHz,$CDCl_3$)
0.94 0.99 0.96 4.02 2.00 3.14
10.5 10.0 9.5 9.0 8.5 8.0 7.5 7.0 6.5 6.0 5.5 5.0 4.5 4.0 3.5 3.0 2.5 2.0 1.5 1.0 0.5 0.0
ppm

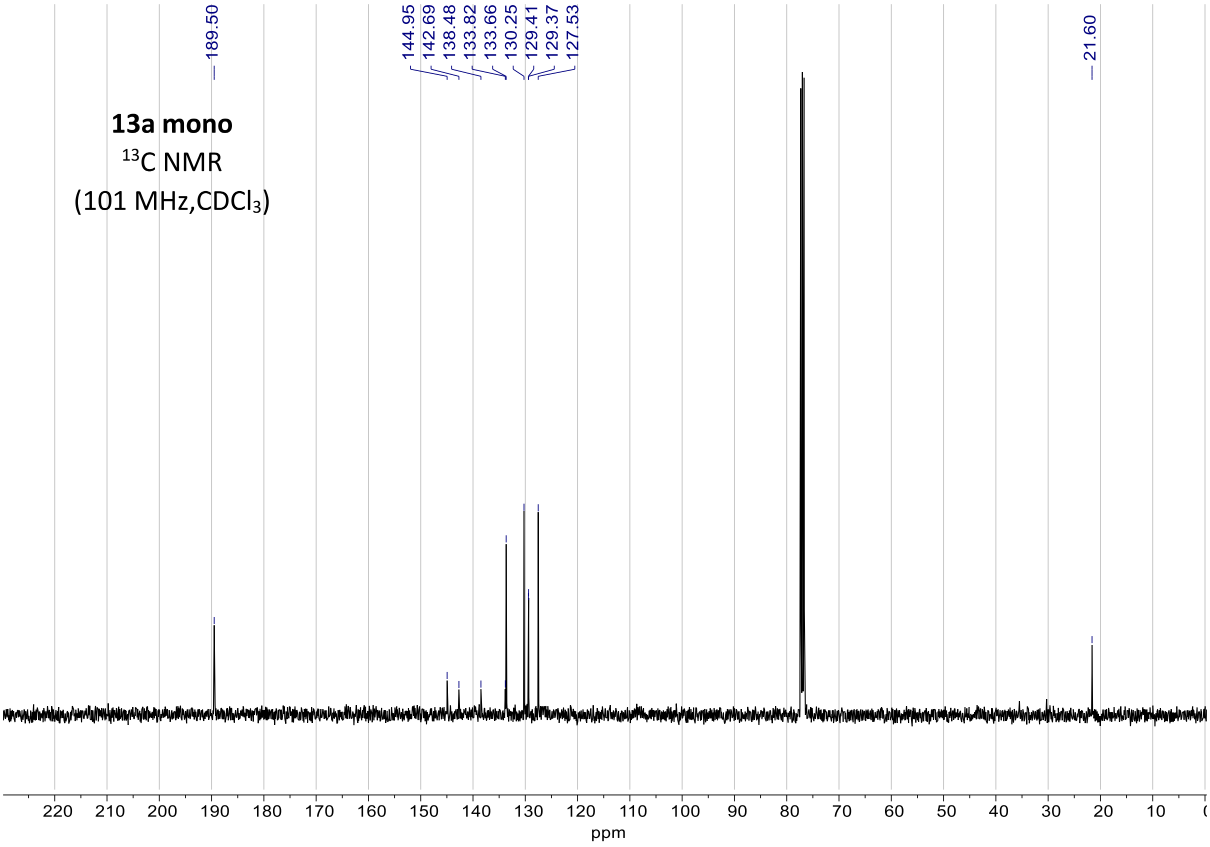
189.50
144.95 142.69 138.48 133.82 133.66 130.25 129.41 129.37 127.53
21.60
13a mono
^{13}C NMR
(101 MHz,$CDCl_3$)
220 210 200 190 180 170 160 150 140 130 120 110 100 90 80 70 60 50 40 30 20 10
ppm

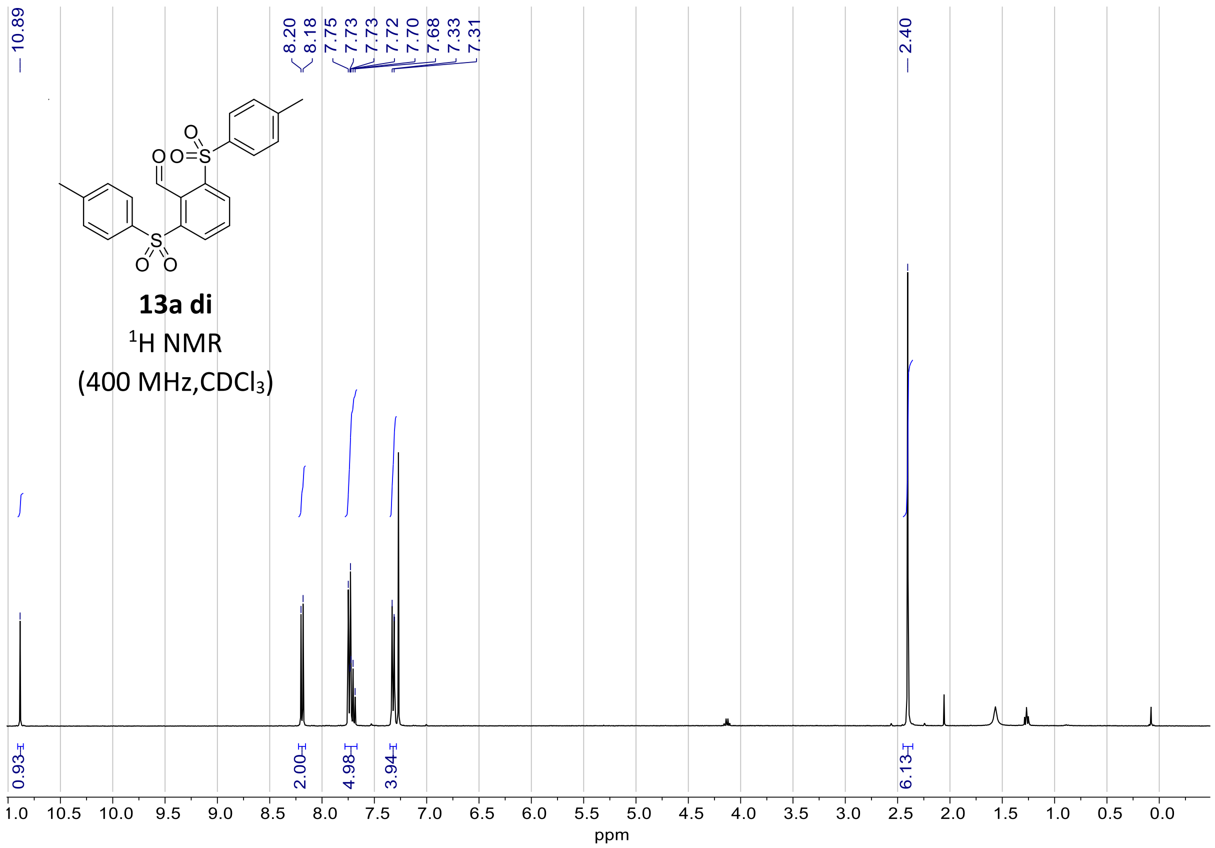
10.89
8.20
8.18
7.75
7.73
7.73
7.72
7.70
7.68
7.33
7.31
2.40
13a di
^{1}H NMR
(400 MHz,CDCl$_3$)
0.93
2.00
4.98
3.94
6.13
ppm

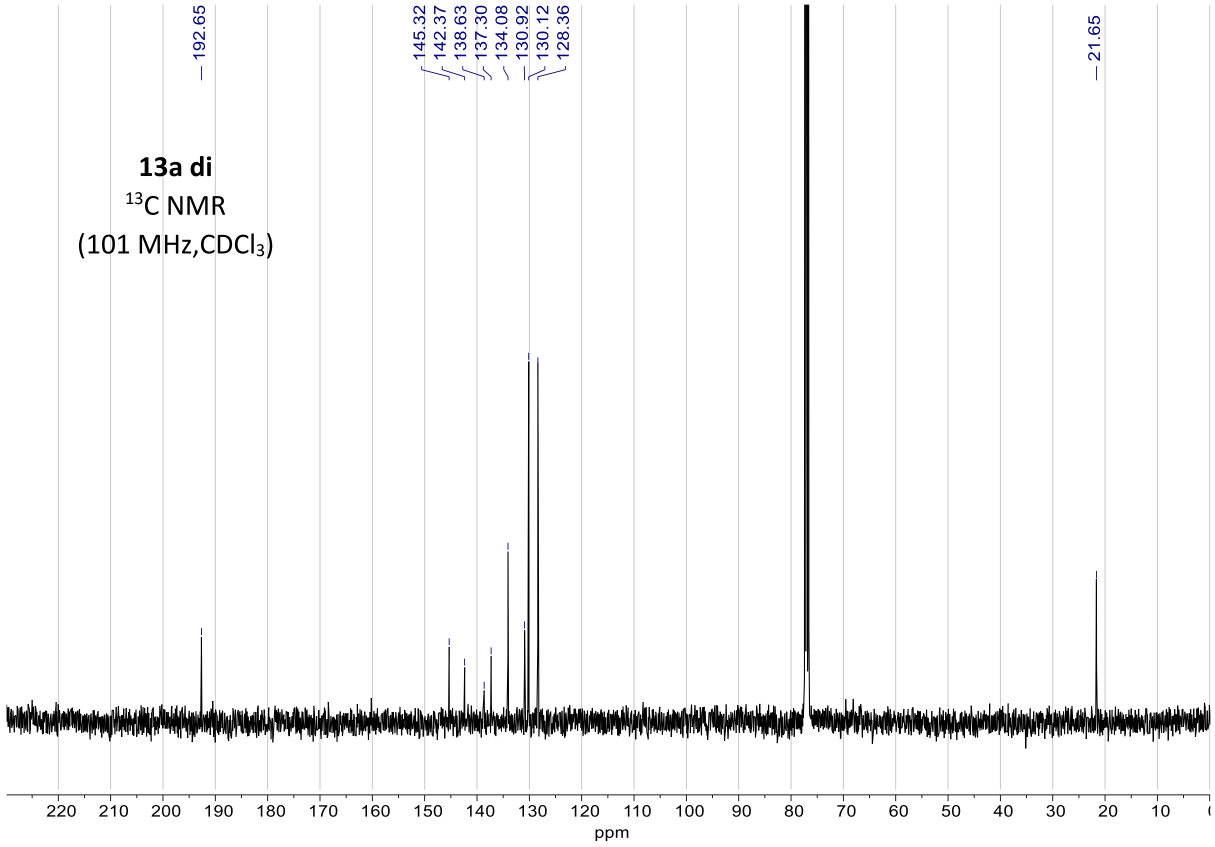
192.65
145.32
142.37
138.63
137.30
134.08
130.92
130.12
128.36
21.65
13a di
^{13}C NMR
(101 MHz,CDCl$_3$)
ppm

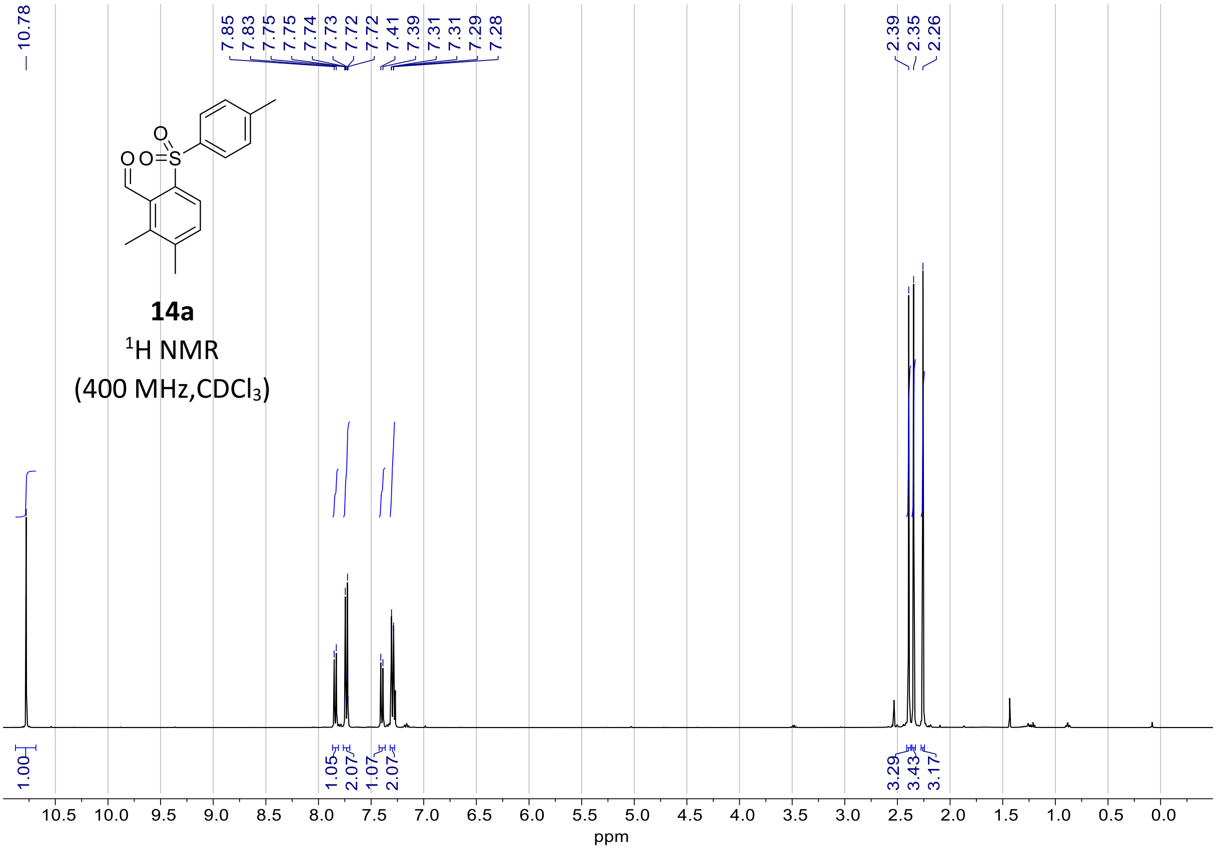

14a
^{1}H NMR
(400 MHz, $CDCl_3$)

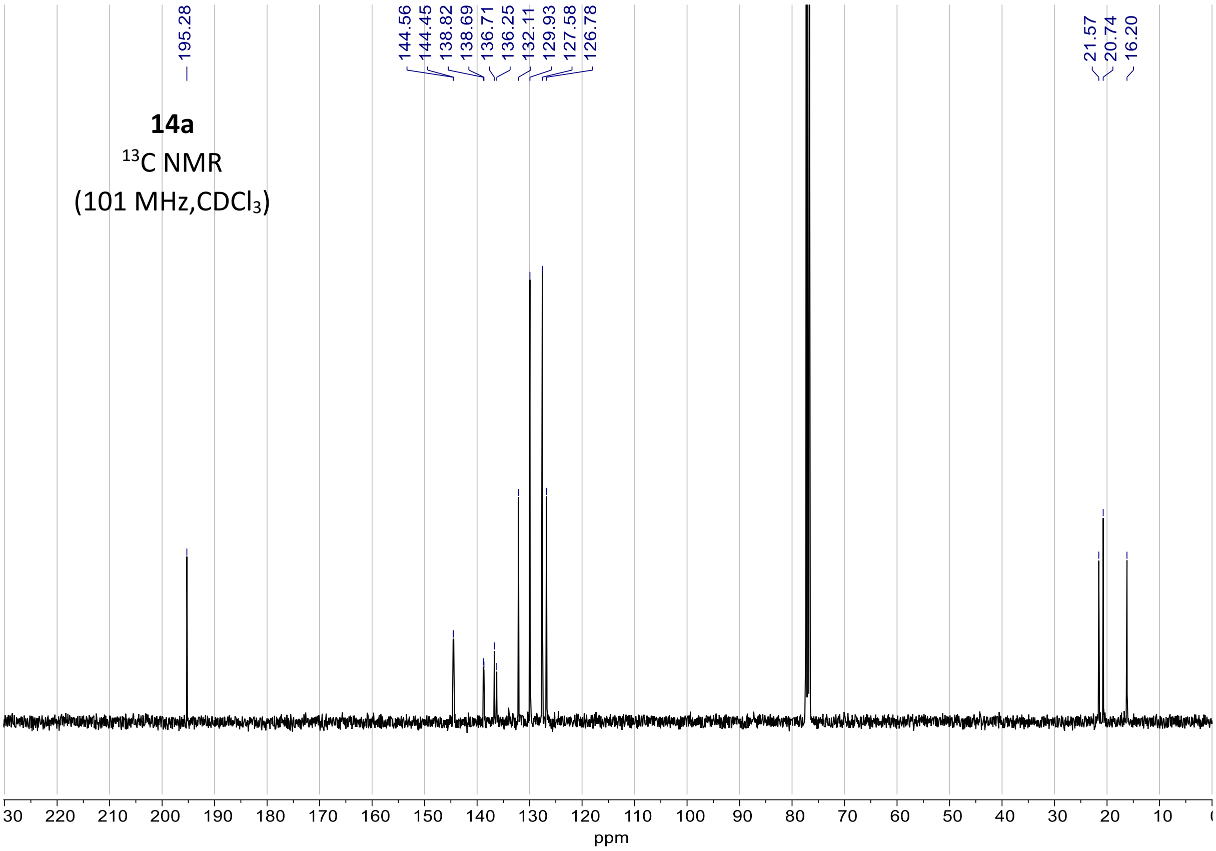

14a
^{13}C NMR
(101 MHz, $CDCl_3$)

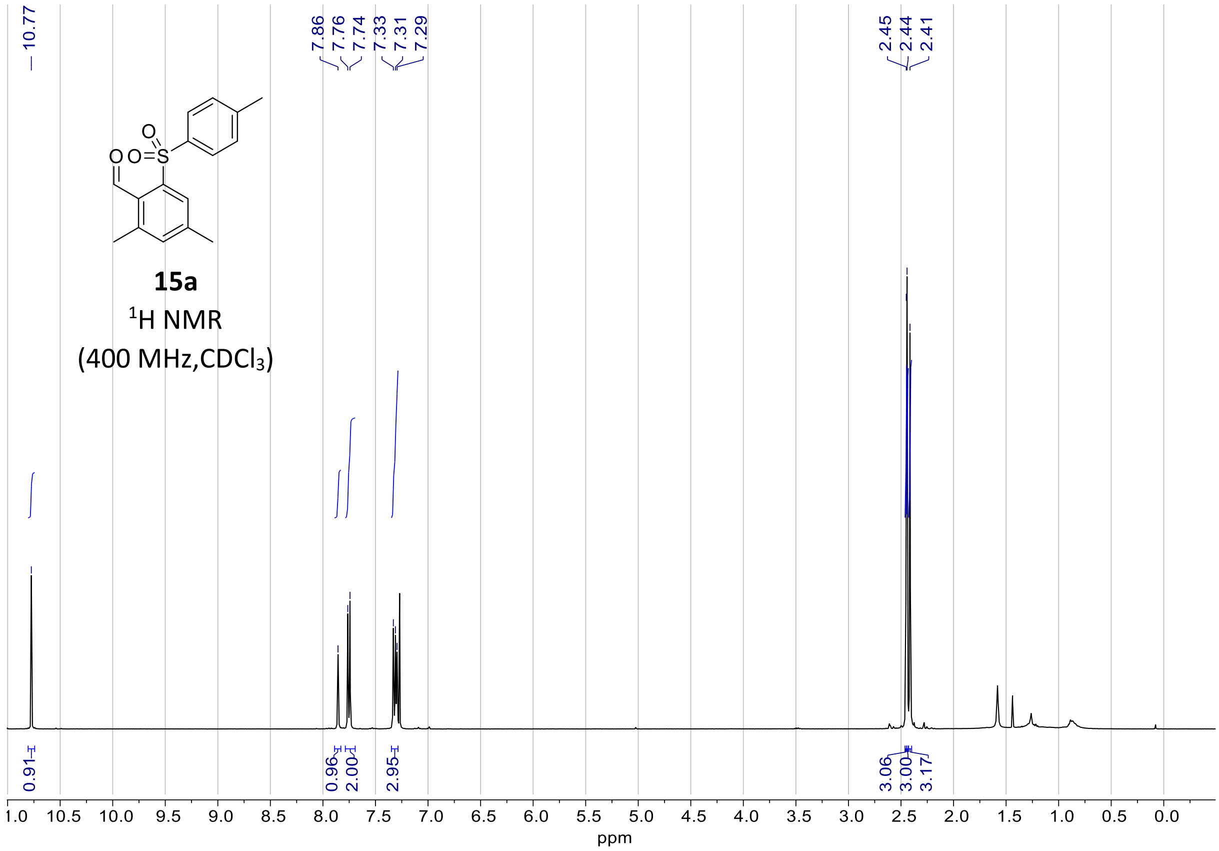
10.77
7.86
7.76
7.74
7.33
7.31
7.29
2.45
2.44
2.41
0.91
0.96
2.00
2.95
3.06
3.00
3.17
ppm
15a
^{1}H NMR
(400 MHz, $CDCl_3$)

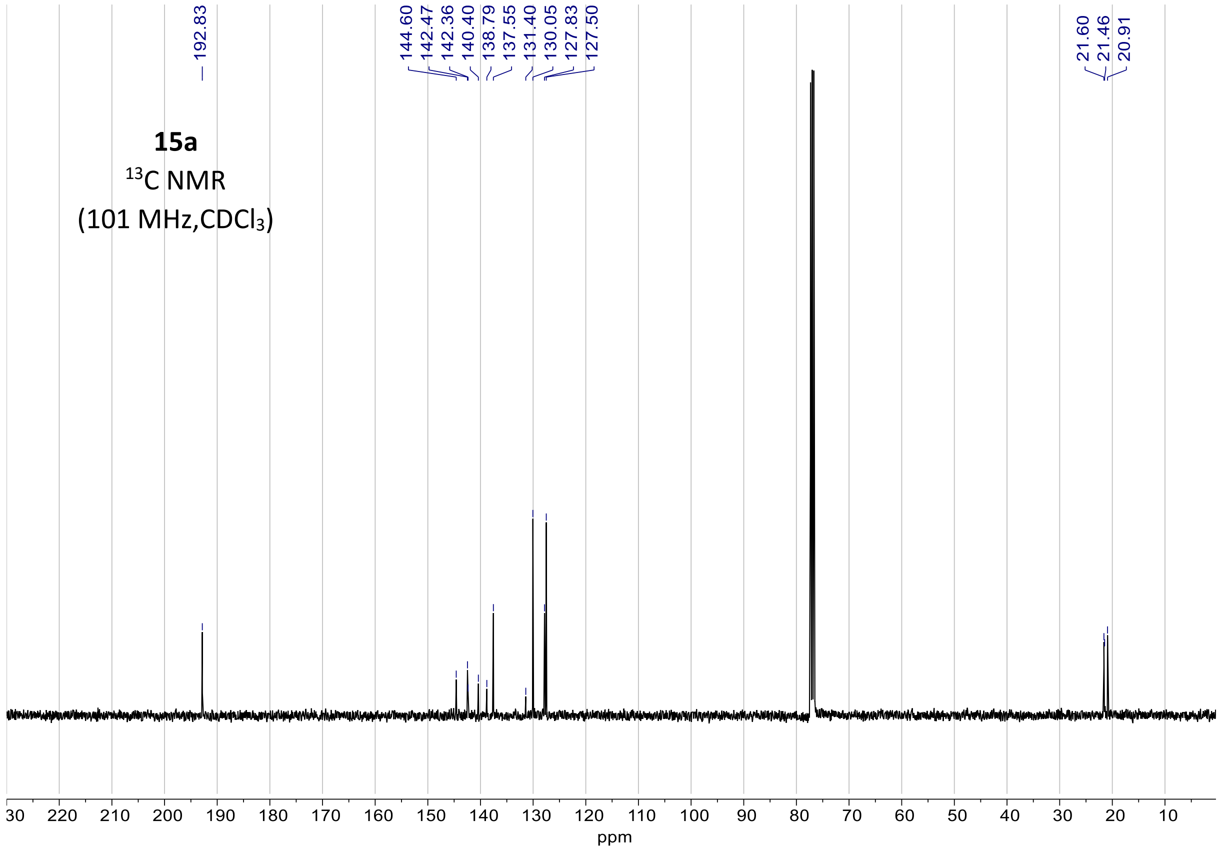
192.83
144.60
142.47
142.36
140.40
138.79
137.55
131.40
130.05
127.83
127.50
21.60
21.46
20.91
ppm
15a
^{13}C NMR
(101 MHz, $CDCl_3$)

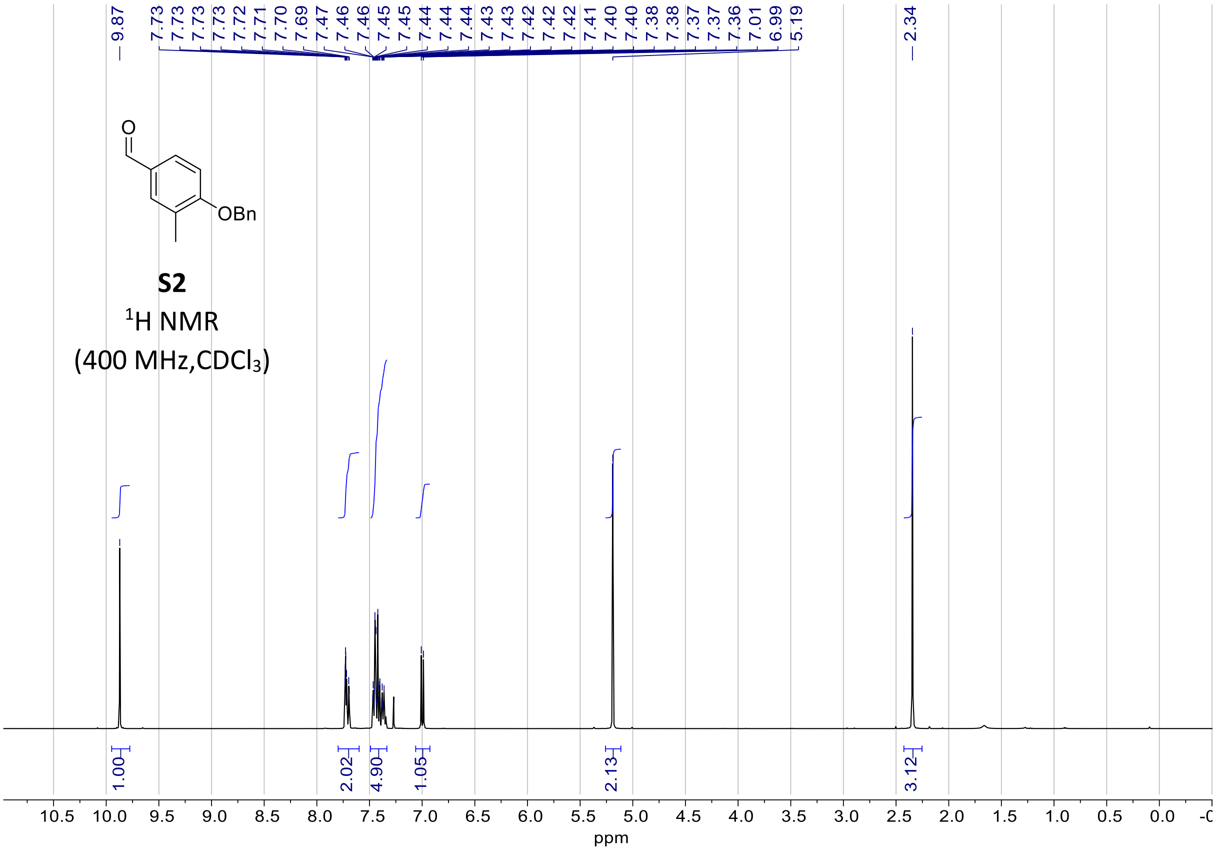
S2
¹H NMR
(400 MHz,CDCl₃)
OBn
O
9.87
7.73
7.73
7.73
7.73
7.72
7.71
7.70
7.69
7.47
7.46
7.46
7.45
7.45
7.44
7.44
7.44
7.43
7.43
7.42
7.42
7.42
7.41
7.40
7.40
7.38
7.38
7.37
7.37
7.36
7.01
6.99
5.19
2.34
1.00
2.02
4.90
1.05
2.13
3.12
ppm

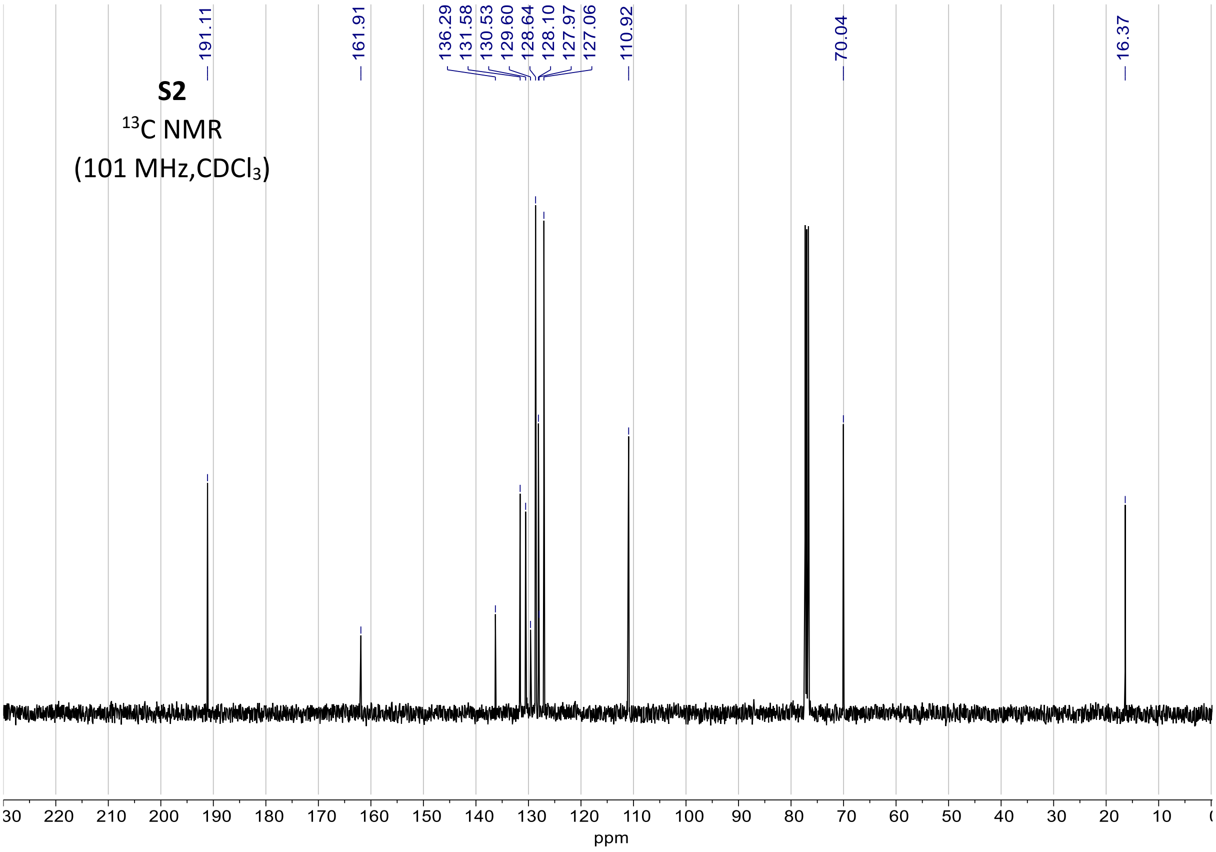
S2
¹³C NMR
(101 MHz,CDCl₃)
191.11
161.91
136.29
131.58
130.53
129.60
128.64
128.10
127.97
127.06
110.92
70.04
16.37
ppm

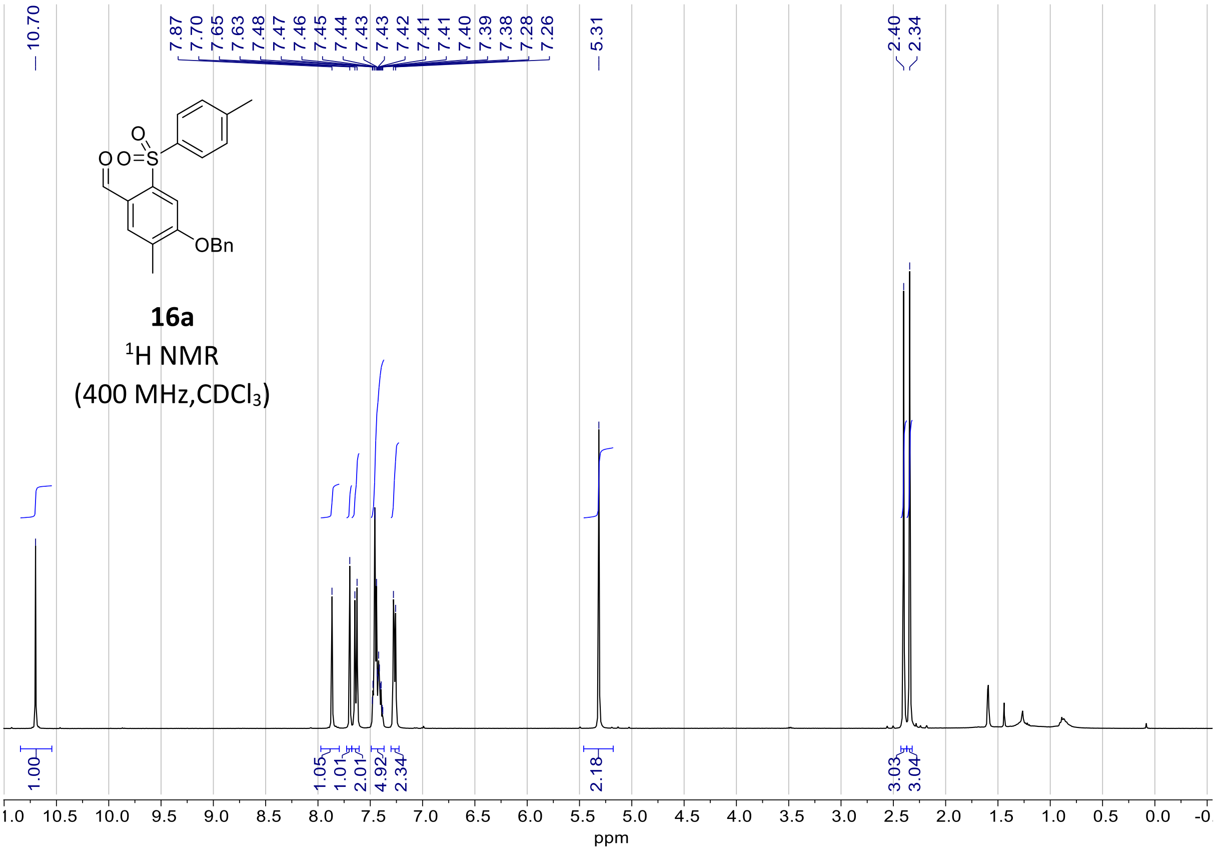
10.70
7.87 7.70 7.65 7.63 7.48 7.47 7.46 7.45 7.44 7.43 7.43 7.42 7.41 7.41 7.40 7.39 7.38 7.28 7.26
5.31
2.40 2.34
O O=S O
OBn
16a
1H NMR
(400 MHz,CDCl3)
1.00
1.05 1.01 2.01 4.92 2.34
2.18
3.03 3.04
ppm

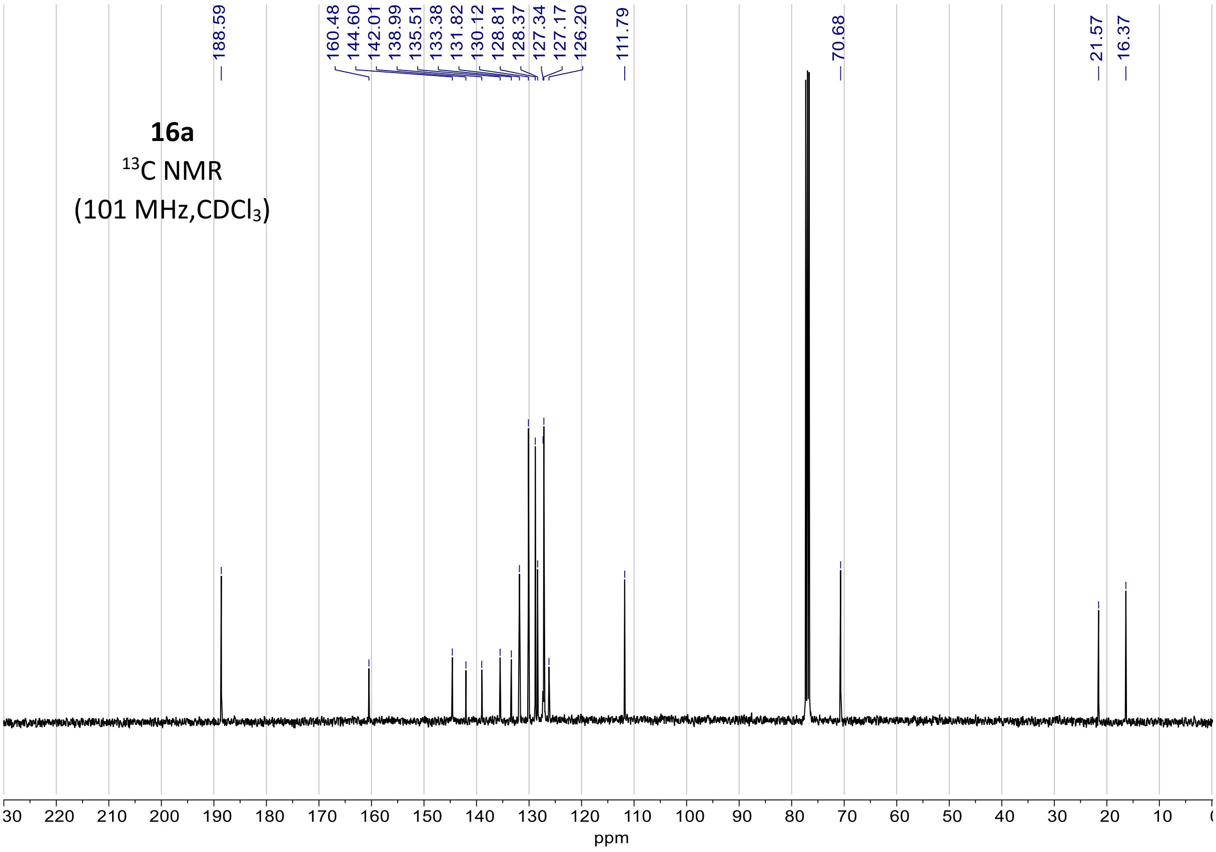
188.59
160.48 144.60 142.01 138.99 135.51 133.38 131.82 130.12 128.81 128.37 127.34 127.17 126.20
111.79
70.68
21.57 16.37
16a
13C NMR
(101 MHz,CDCl3)
ppm

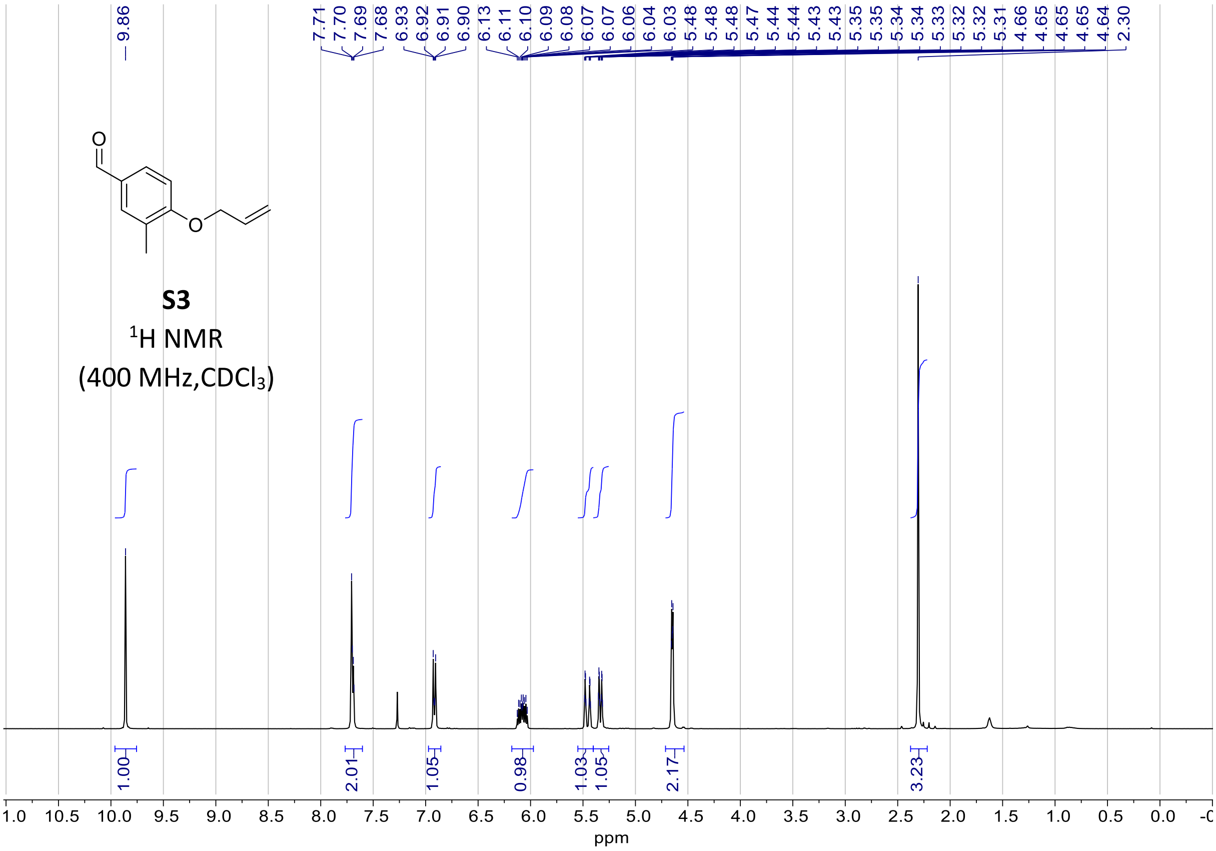

S3

^{1}H NMR

(400 MHz, $CDCl_3$)

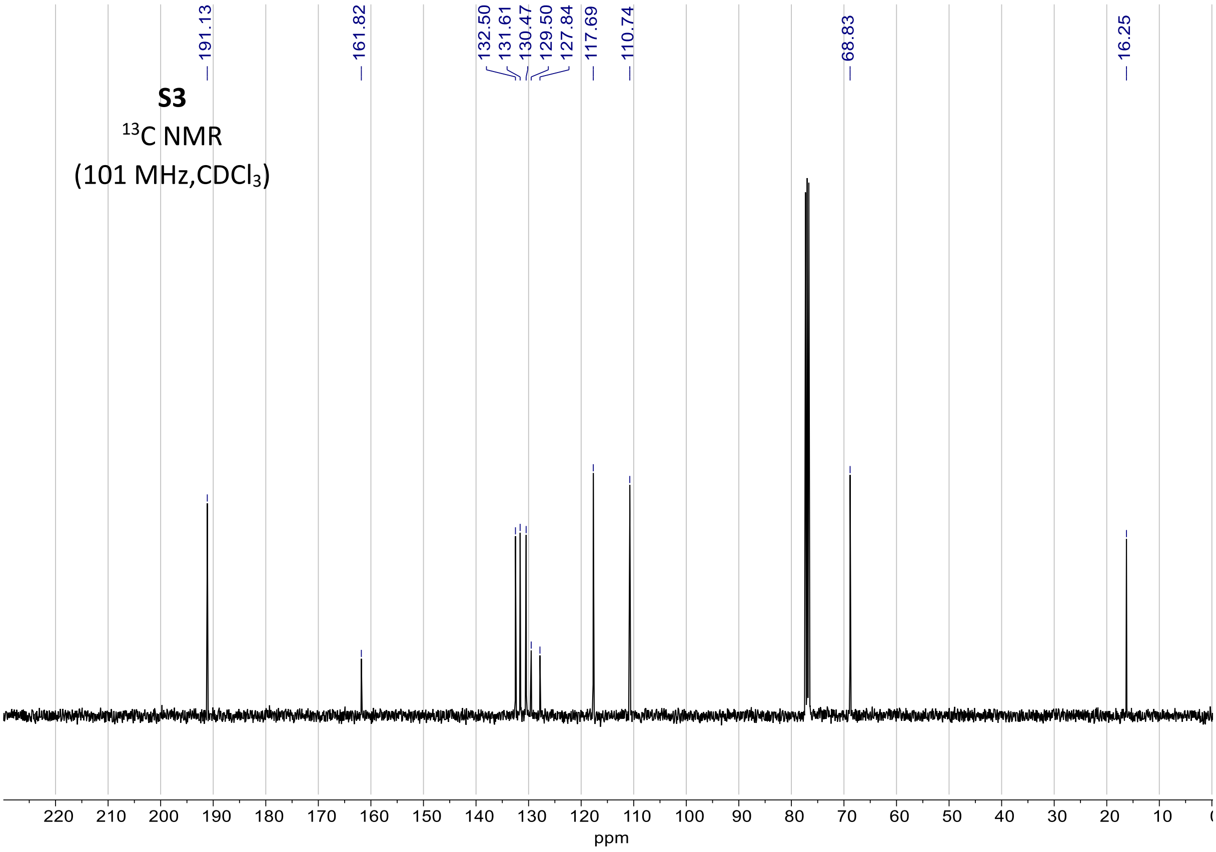

S3

^{13}C NMR

(101 MHz, $CDCl_3$)

17a

^{1}H NMR

(400 MHz,$CDCl_3$)

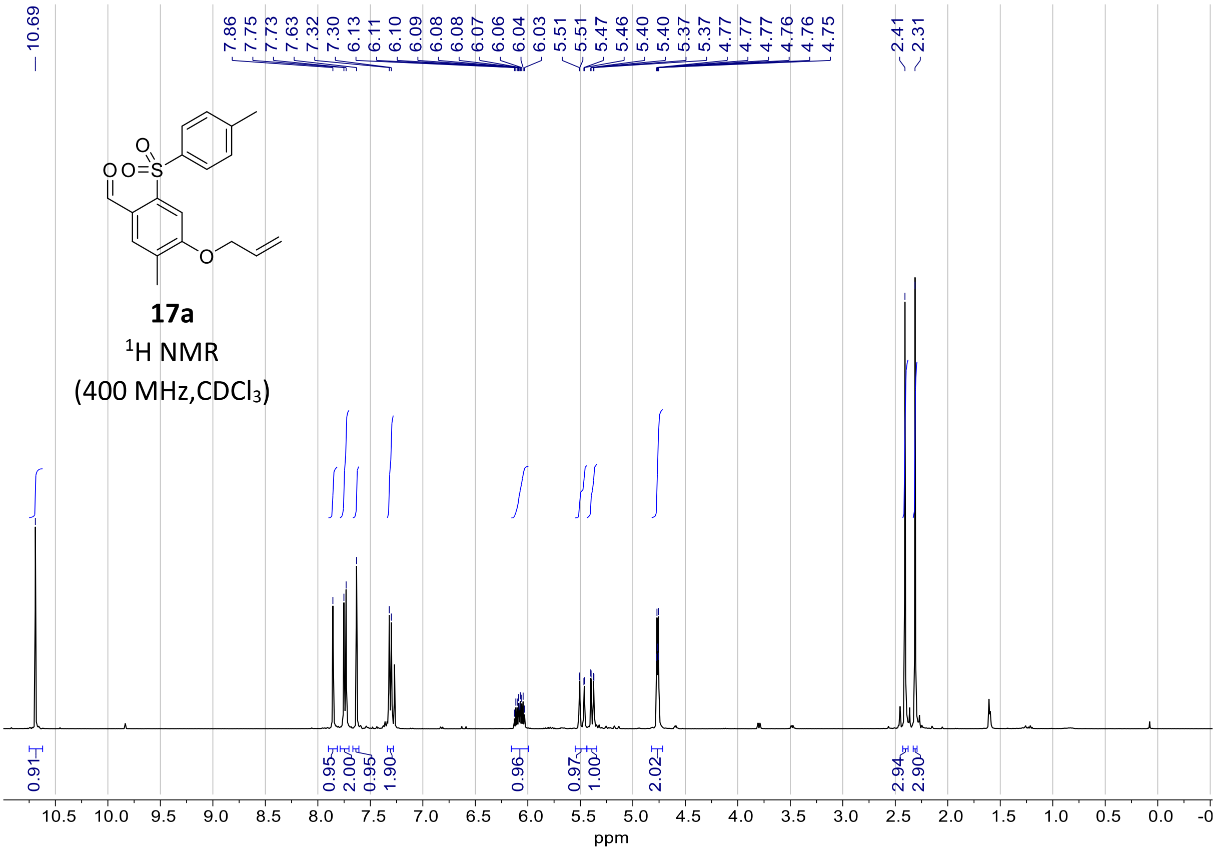

17a

^{13}C NMR

(101 MHz,$CDCl_3$)

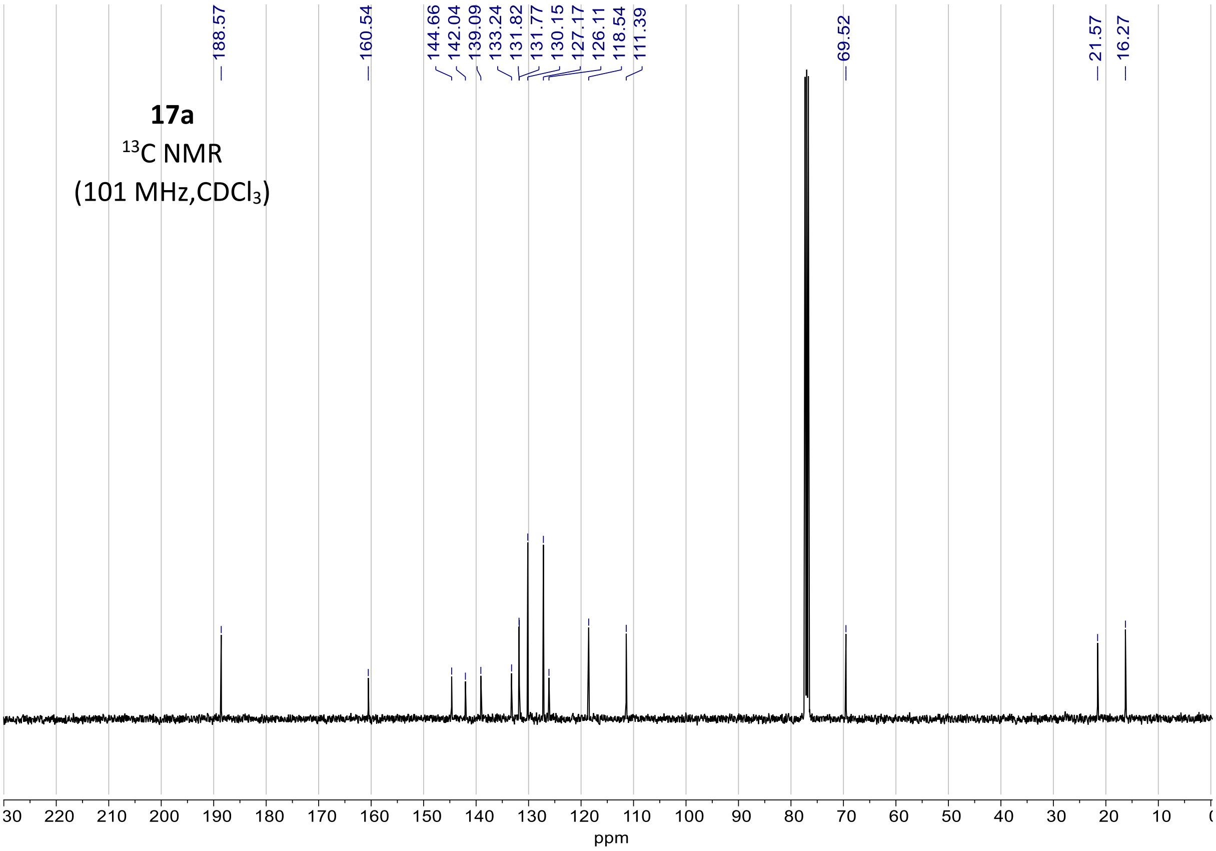

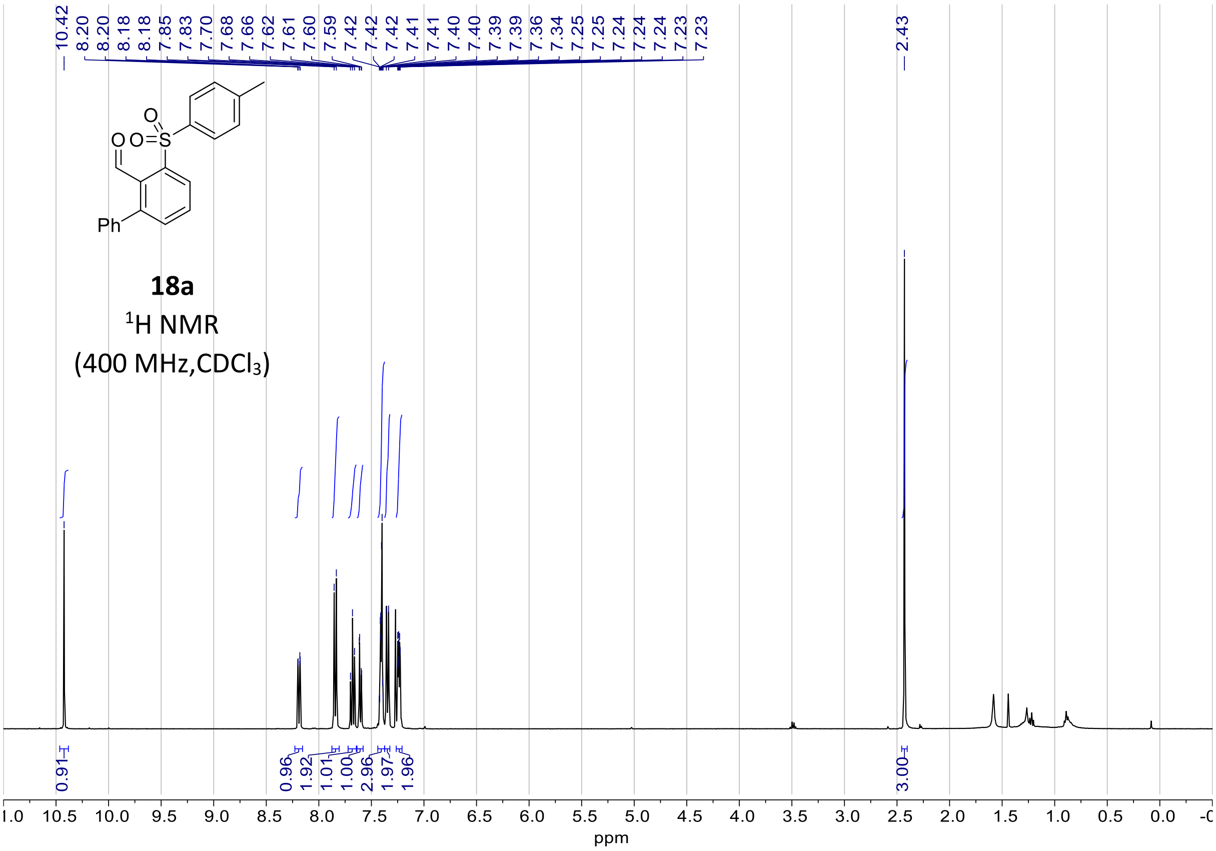
18a
^{1}H NMR
(400 MHz, CDCl$_3$)
10.42, 8.20, 8.20, 8.18, 8.18, 7.85, 7.83, 7.70, 7.68, 7.66, 7.62, 7.61, 7.60, 7.59, 7.42, 7.42, 7.42, 7.41, 7.41, 7.40, 7.40, 7.39, 7.39, 7.36, 7.34, 7.25, 7.24, 7.24, 7.24, 7.23, 7.23
2.43
0.91, 0.96, 1.92, 1.01, 1.00, 2.96, 1.97, 1.96, 3.00
ppm

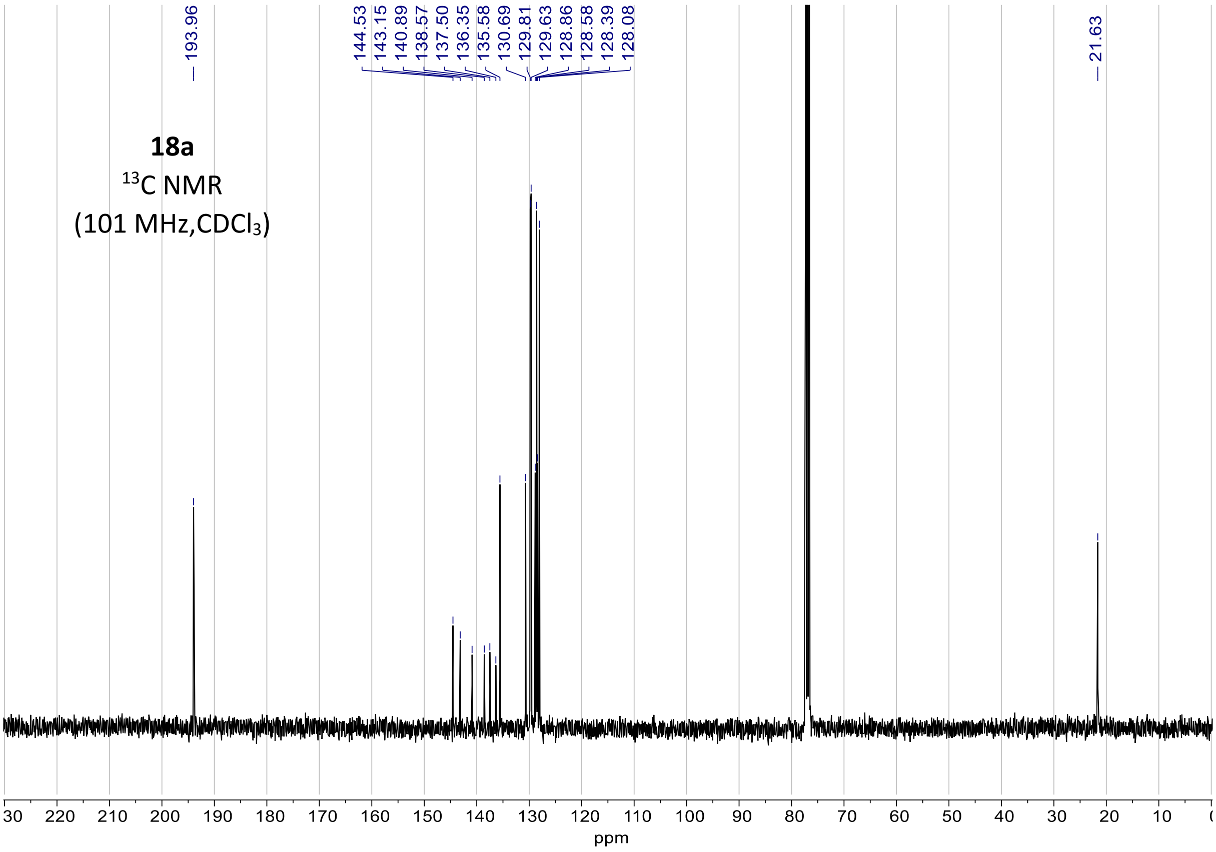
18a
^{13}C NMR
(101 MHz, CDCl$_3$)
193.96
144.53, 143.15, 140.89, 138.57, 137.50, 136.35, 135.58, 130.69, 129.81, 129.63, 128.86, 128.58, 128.39, 128.08
21.63
ppm

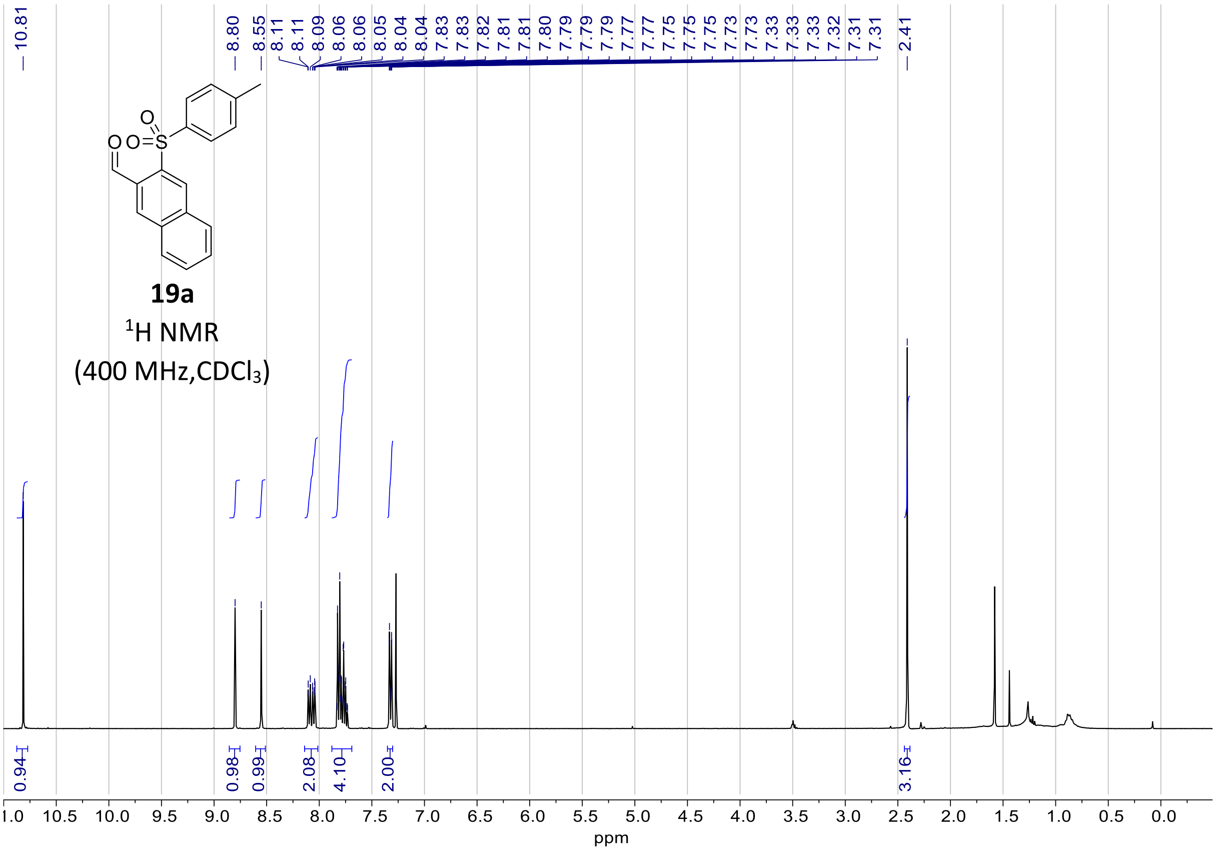
10.81
8.80
8.55
8.11
8.11
8.09
8.06
8.06
8.05
8.04
8.04
7.83
7.83
7.82
7.81
7.81
7.80
7.79
7.79
7.79
7.77
7.77
7.75
7.75
7.75
7.73
7.73
7.33
7.33
7.33
7.32
7.31
7.31
2.41
19a
^{1}H NMR
(400 MHz,CDCl$_3$)
0.94
0.98
0.99
2.08
4.10
2.00
3.16
ppm

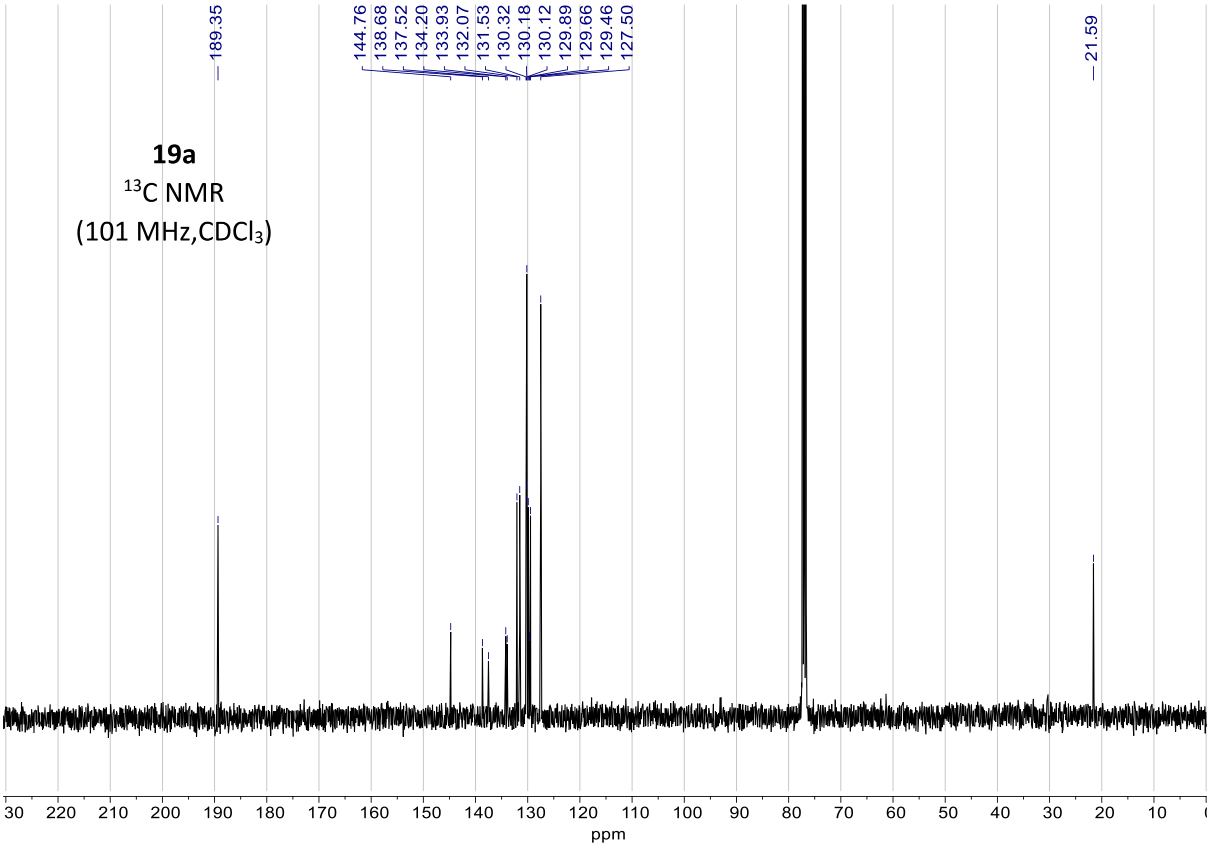
189.35
144.76
138.68
137.52
134.20
133.93
132.07
131.53
130.32
130.18
130.12
129.89
129.66
129.46
127.50
21.59
19a
^{13}C NMR
(101 MHz,CDCl$_3$)
ppm

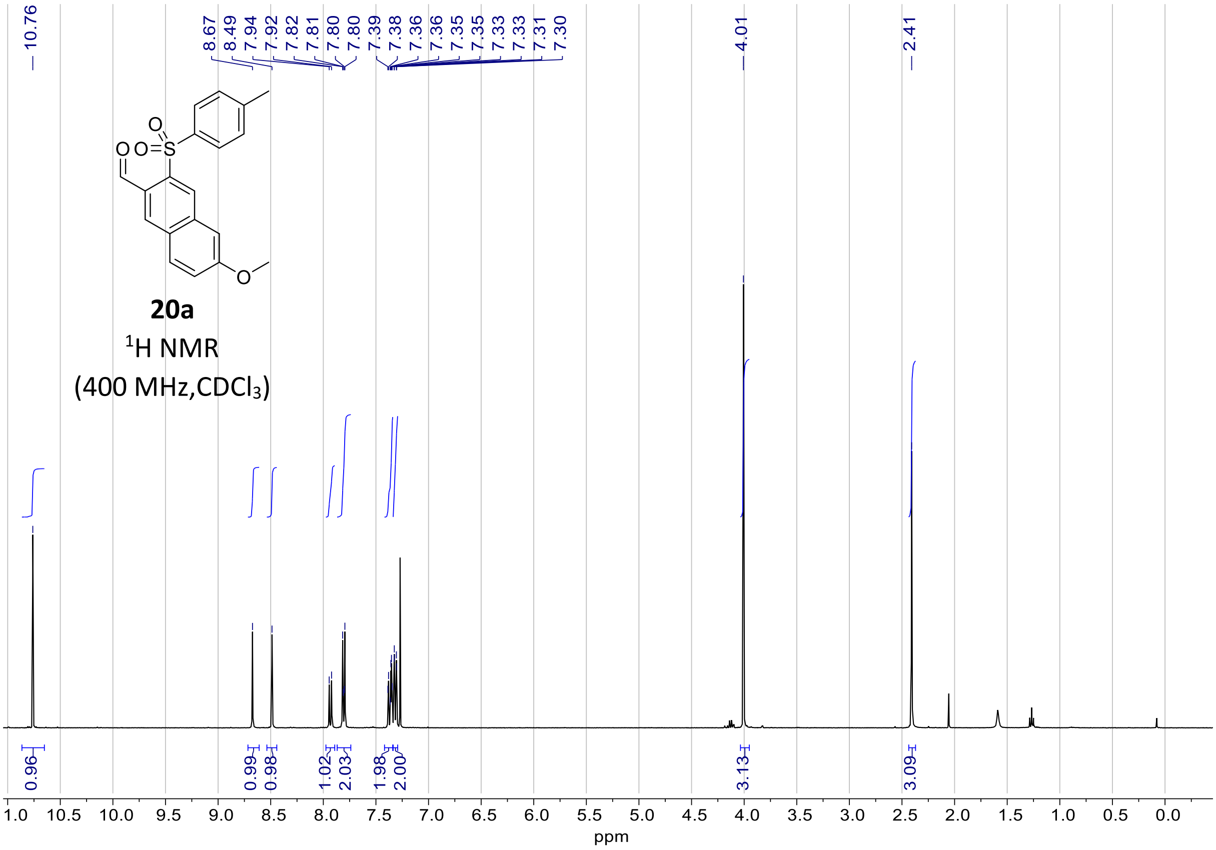

10.76
8.67
8.49
7.94
7.92
7.82
7.81
7.80
7.80
7.39
7.38
7.36
7.36
7.35
7.35
7.33
7.33
7.31
7.30
4.01
2.41
O
O
O=S
O
20a
^{1}H NMR
(400 MHz,CDCl$_3$)
0.96
0.99
0.98
1.02
2.03
1.98
2.00
3.13
3.09
ppm

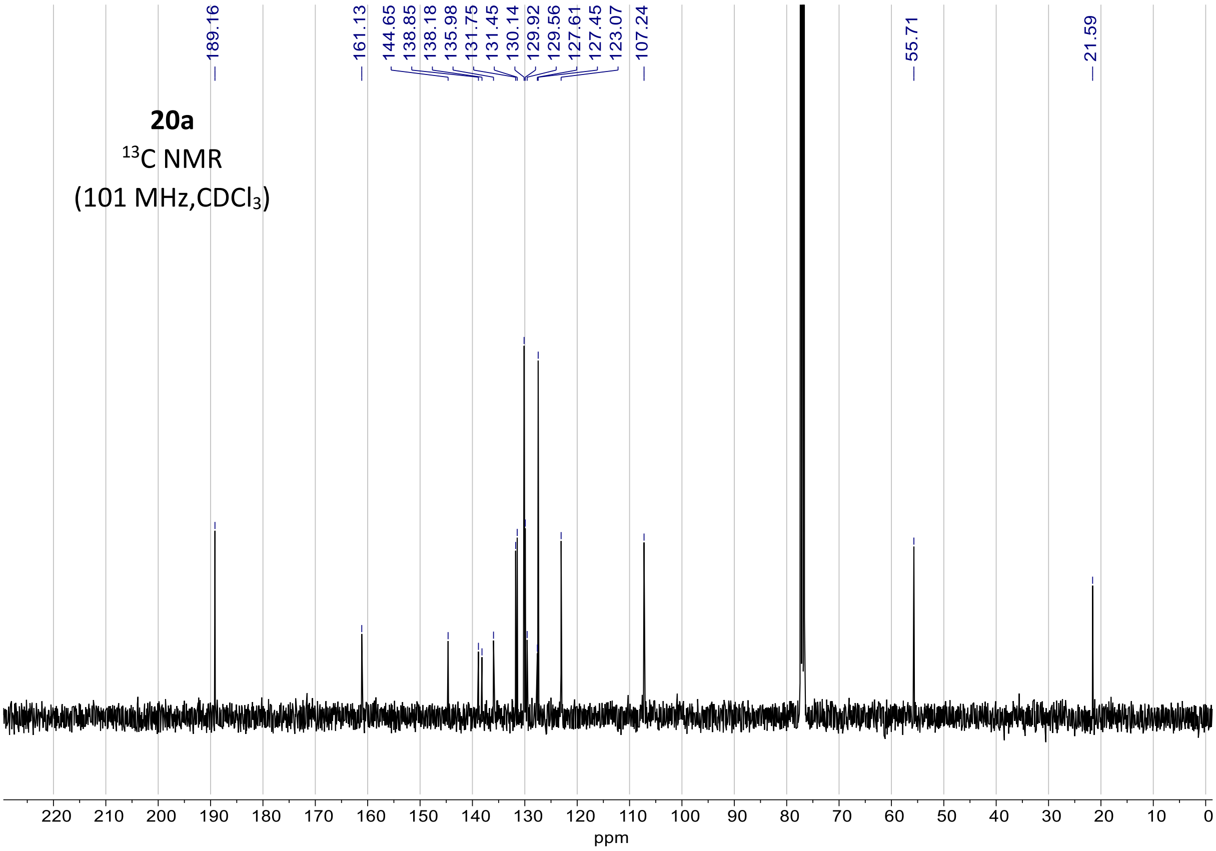

189.16
161.13
144.65
138.85
138.18
135.98
131.75
131.45
130.14
129.92
129.56
127.61
127.45
123.07
107.24
55.71
21.59
20a
^{13}C NMR
(101 MHz,CDCl$_3$)
ppm

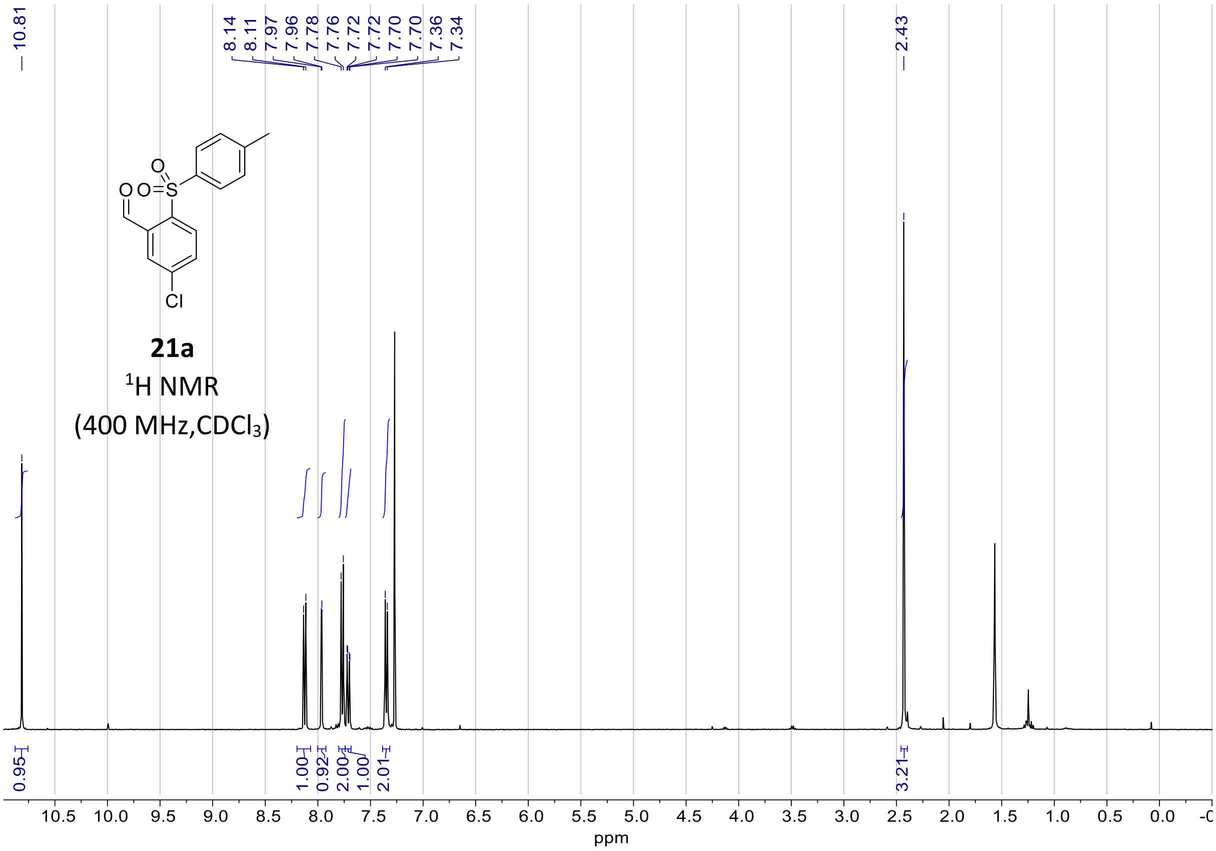

21a

^{1}H NMR

(400 MHz,$CDCl_3$)

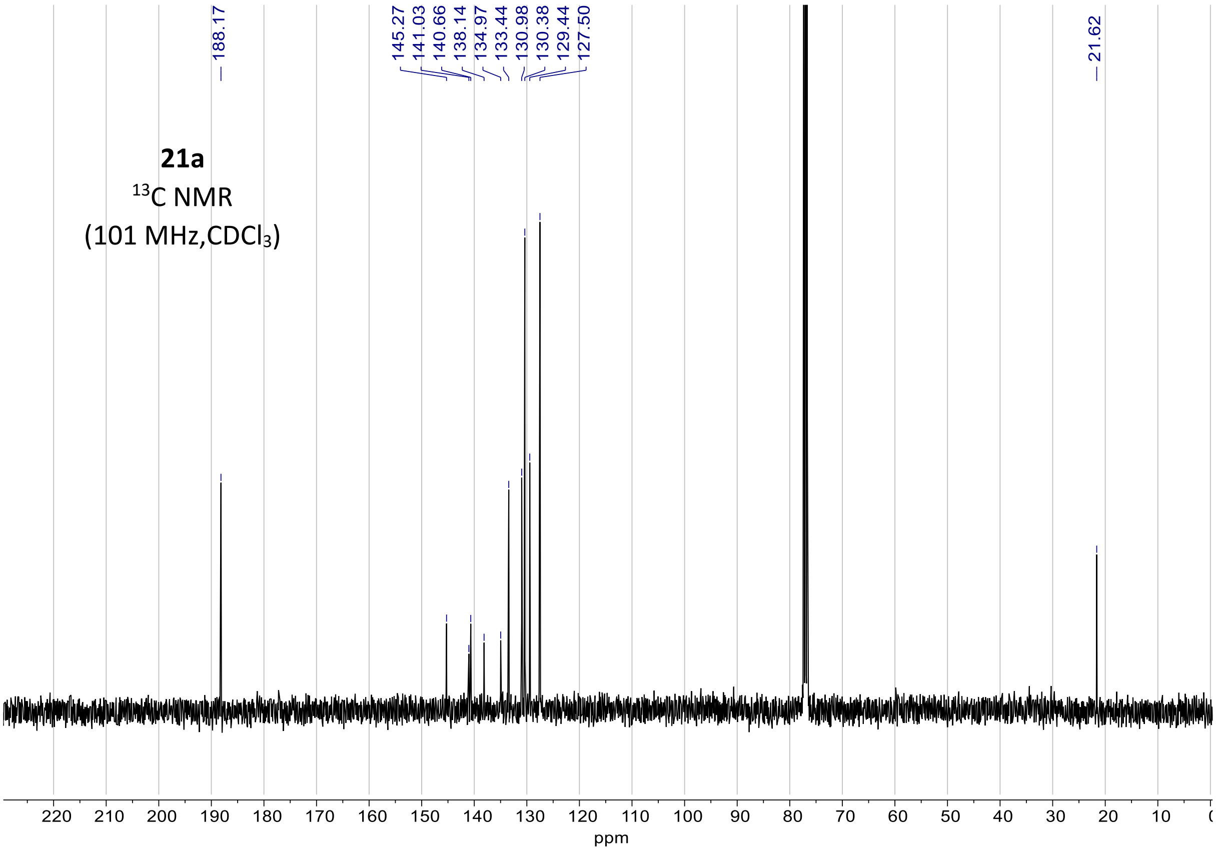

21a

^{13}C NMR

(101 MHz,$CDCl_3$)

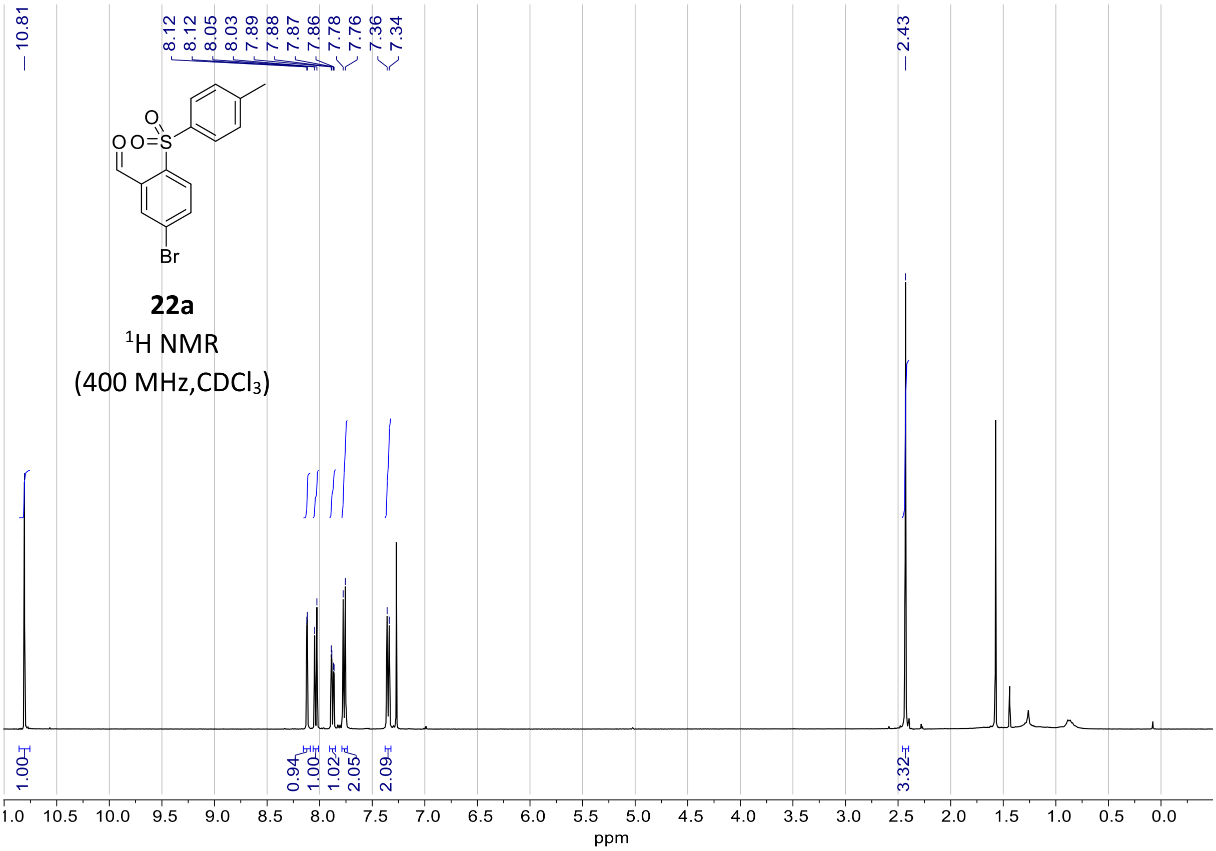
10.81
8.12
8.12
8.05
8.03
7.89
7.88
7.87
7.86
7.78
7.76
7.36
7.34
2.43
O
O=S
O
Br
22a
¹H NMR
(400 MHz,CDCl₃)
1.00
0.94
1.00
1.02
2.05
2.09
3.32
ppm

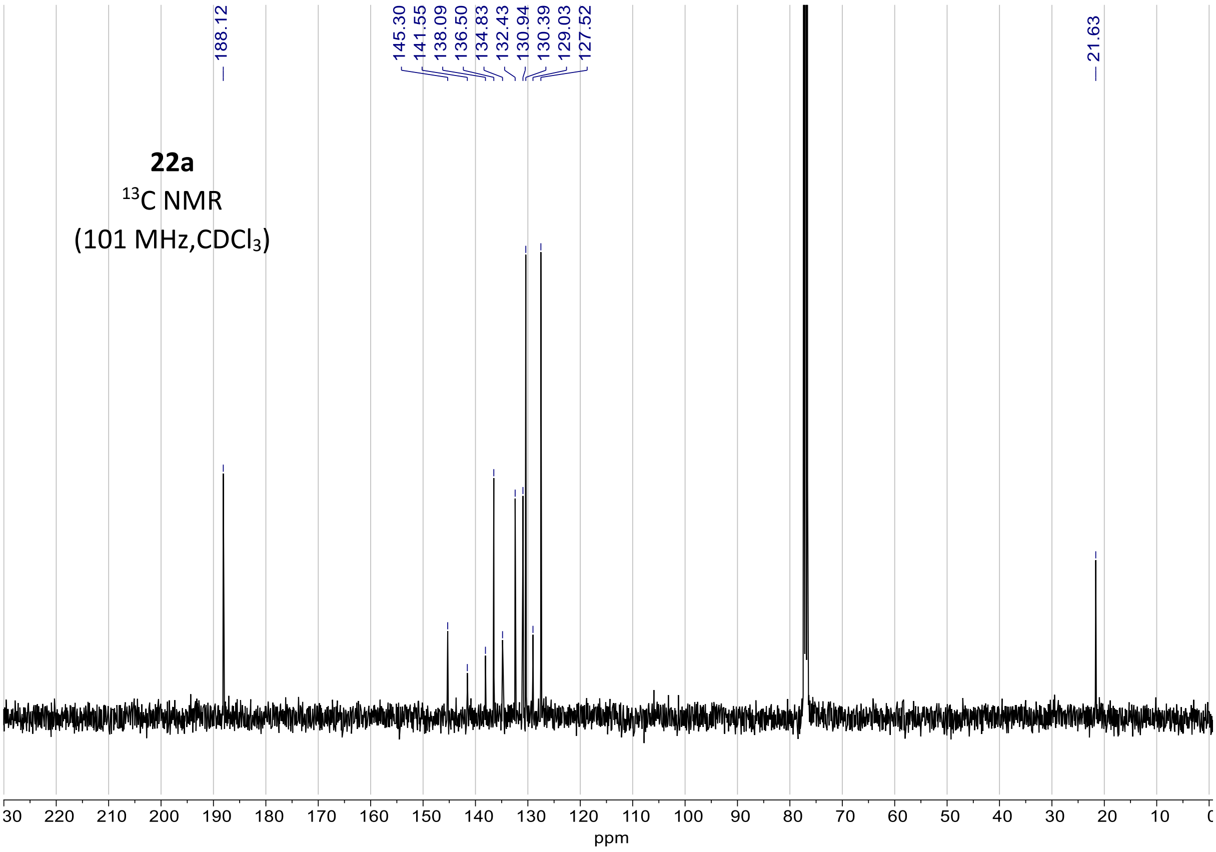
188.12
145.30
141.55
138.09
136.50
134.83
132.43
130.94
130.39
129.03
127.52
21.63
22a
¹³C NMR
(101 MHz,CDCl₃)
ppm

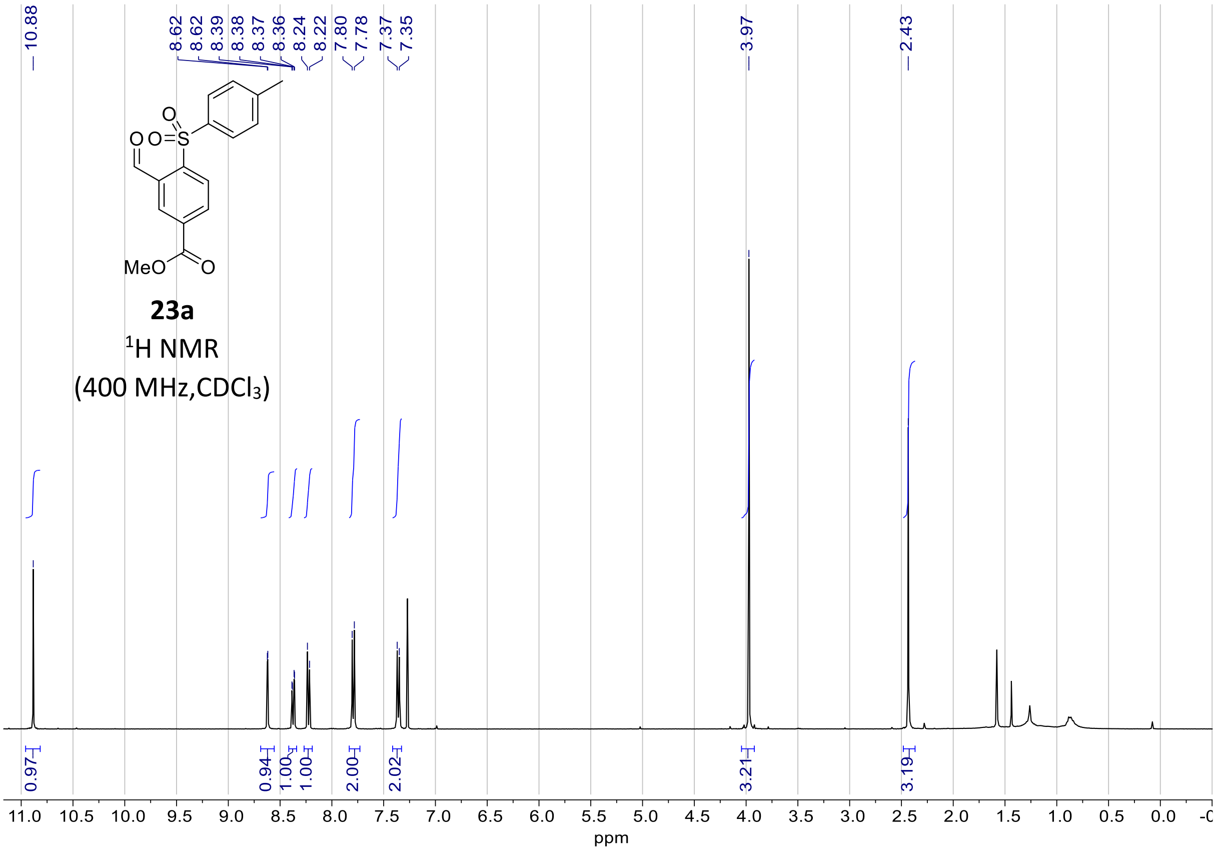
10.88
8.62
8.62
8.39
8.38
8.37
8.36
8.24
8.22
7.80
7.78
7.37
7.35
3.97
2.43
O
O=S
O
MeO
O
23a
1H NMR
(400 MHz,CDCl3)
0.97
0.94
1.00
1.00
2.00
2.02
3.21
3.19
11.0 10.5 10.0 9.5 9.0 8.5 8.0 7.5 7.0 6.5 6.0 5.5 5.0 4.5 4.0 3.5 3.0 2.5 2.0 1.5 1.0 0.5 0.0
ppm

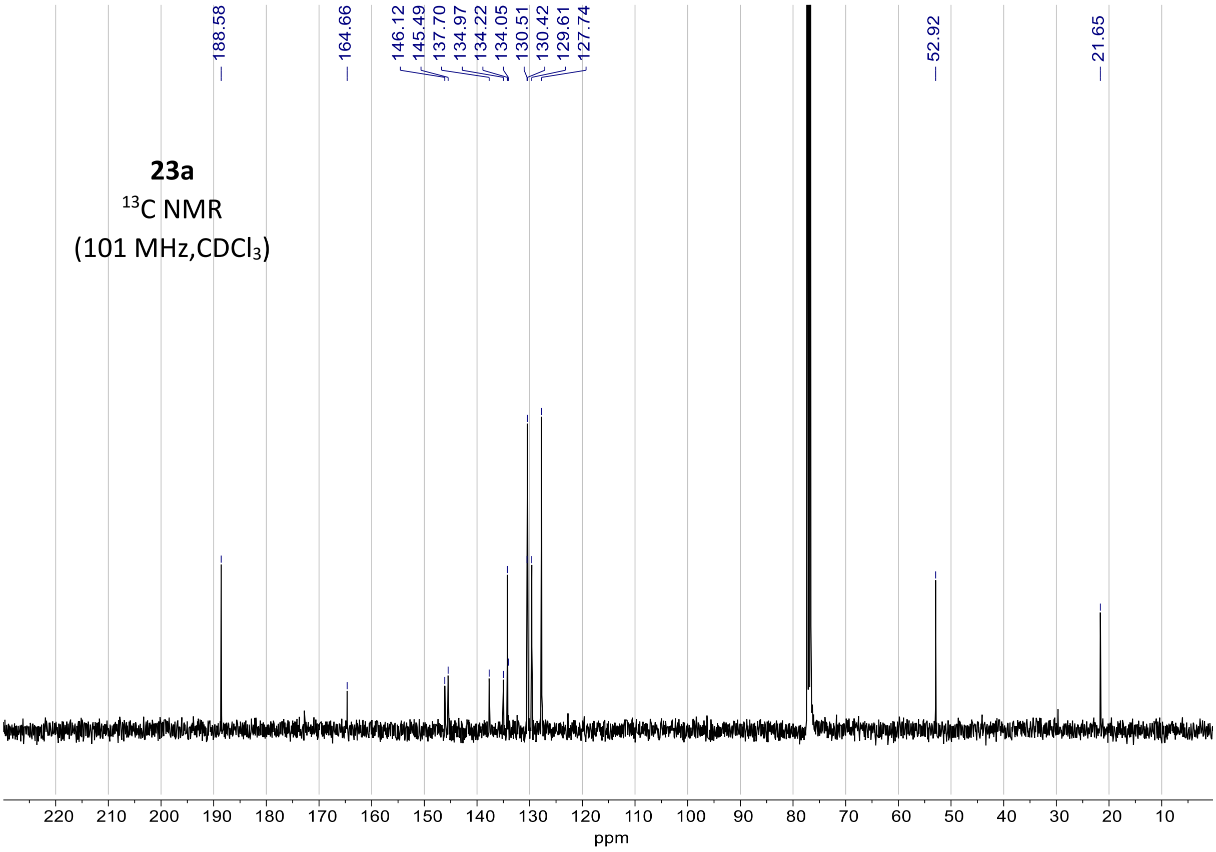
188.58
164.66
146.12
145.49
137.70
134.97
134.22
134.05
130.51
130.42
129.61
127.74
52.92
21.65
23a
13C NMR
(101 MHz,CDCl3)
220 210 200 190 180 170 160 150 140 130 120 110 100 90 80 70 60 50 40 30 20 10
ppm

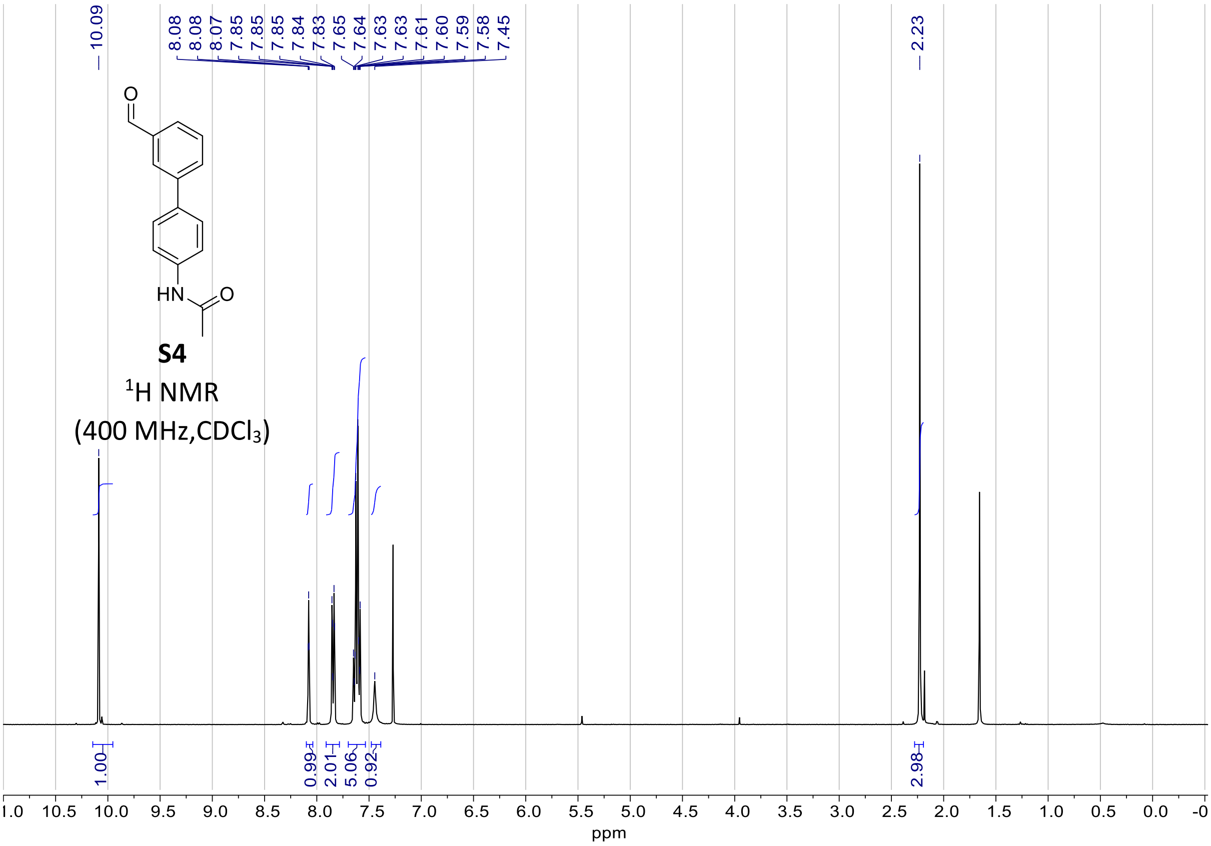
10.09
8.08
8.08
8.07
7.85
7.85
7.85
7.84
7.83
7.65
7.64
7.63
7.63
7.61
7.60
7.59
7.58
7.45
2.23
O
HN
O
S4
1H NMR
(400 MHz,CDCl3)
1.00
0.99
2.01
5.06
0.92
2.98
ppm

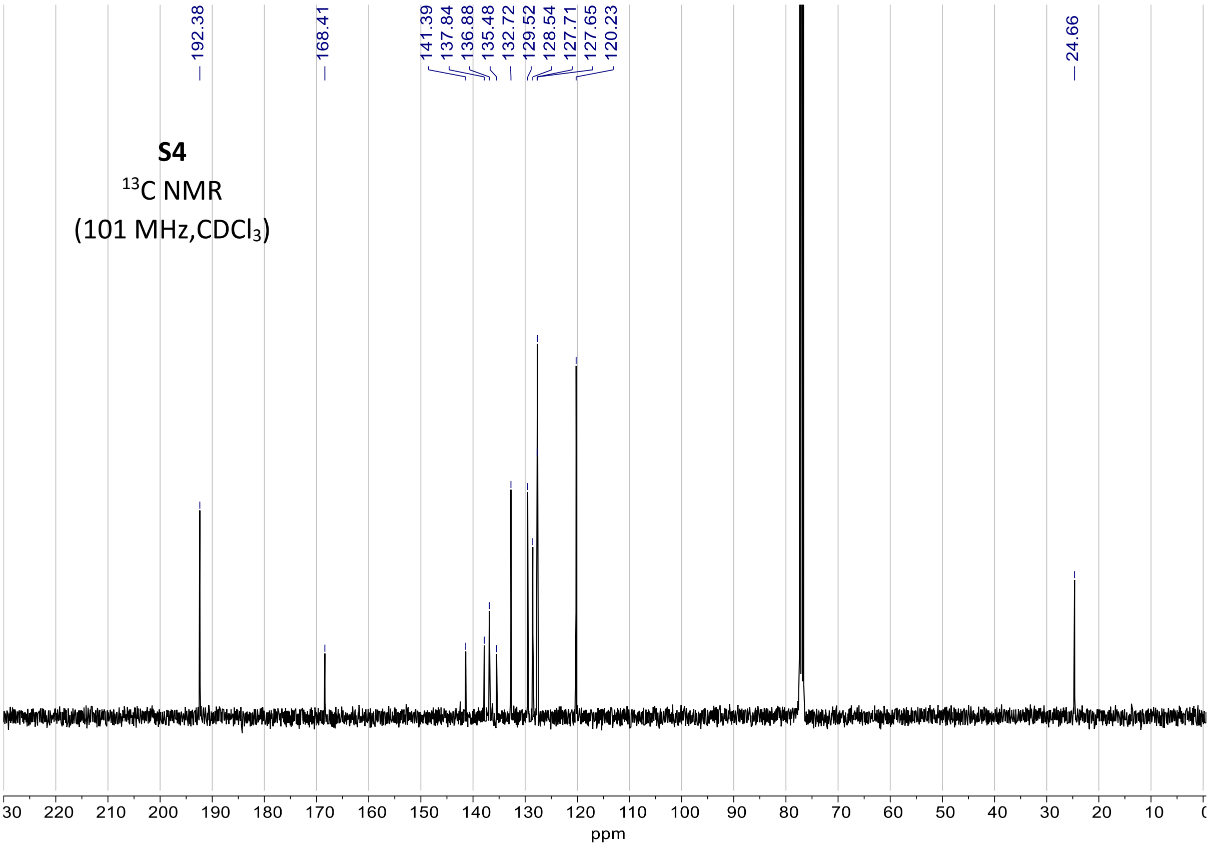
192.38
168.41
141.39
137.84
136.88
135.48
132.72
129.52
128.54
127.71
127.65
120.23
24.66
S4
13C NMR
(101 MHz,CDCl3)
ppm

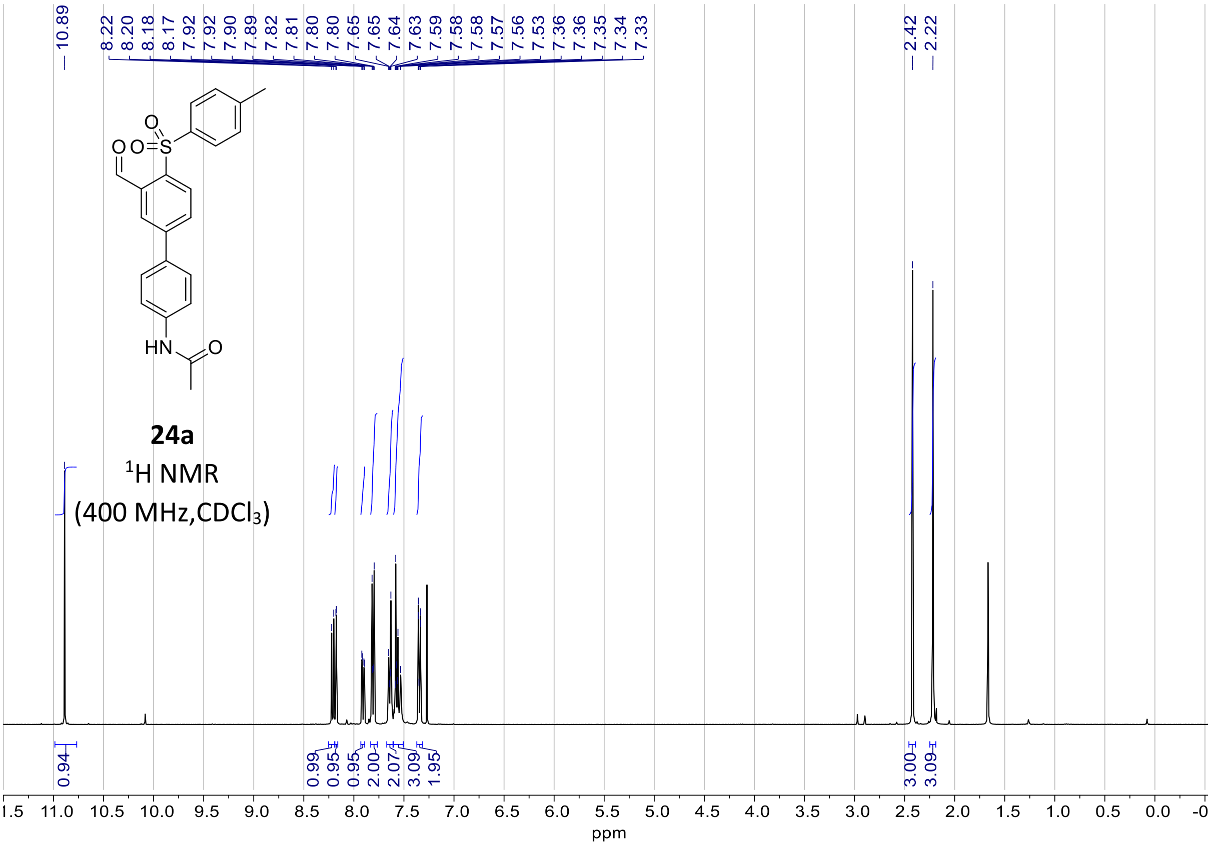
24a
^{1}H NMR
(400 MHz,CDCl$_3$)

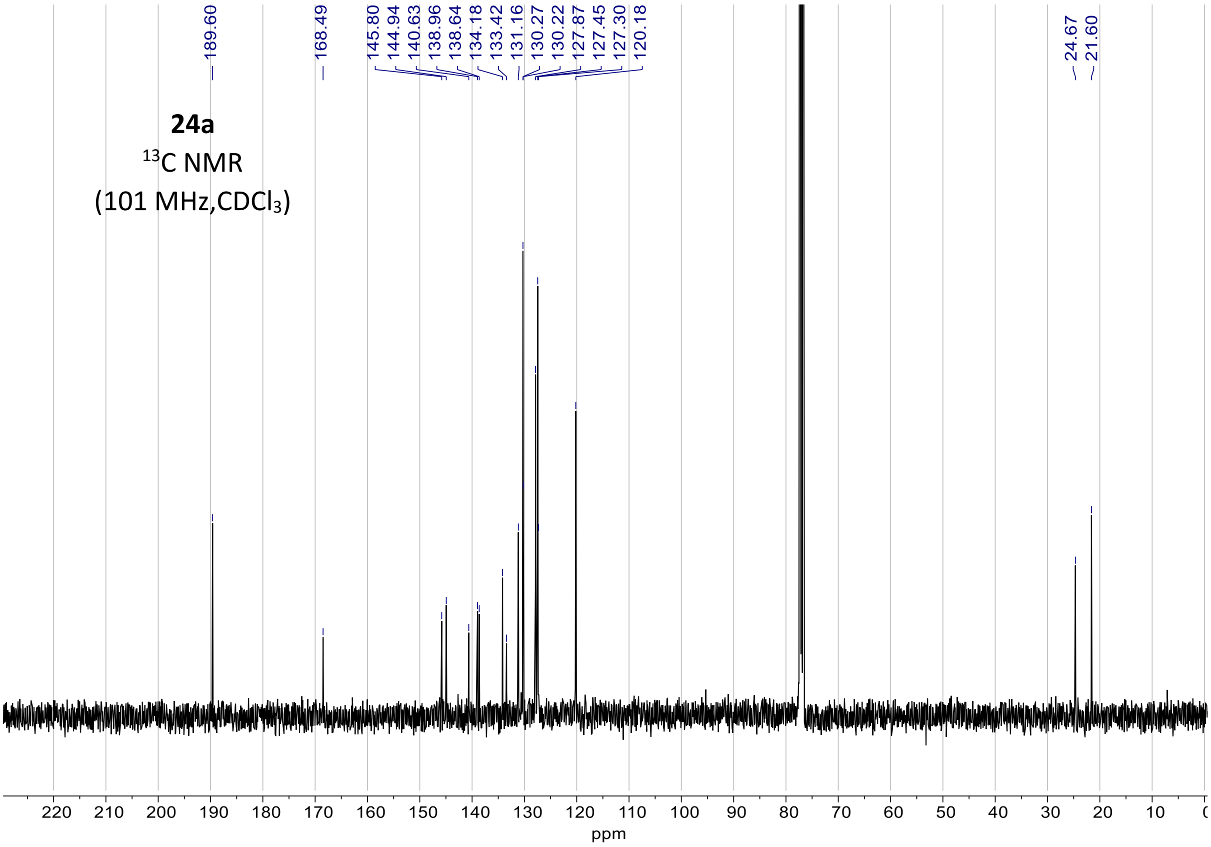
24a
^{13}C NMR
(101 MHz,CDCl$_3$)

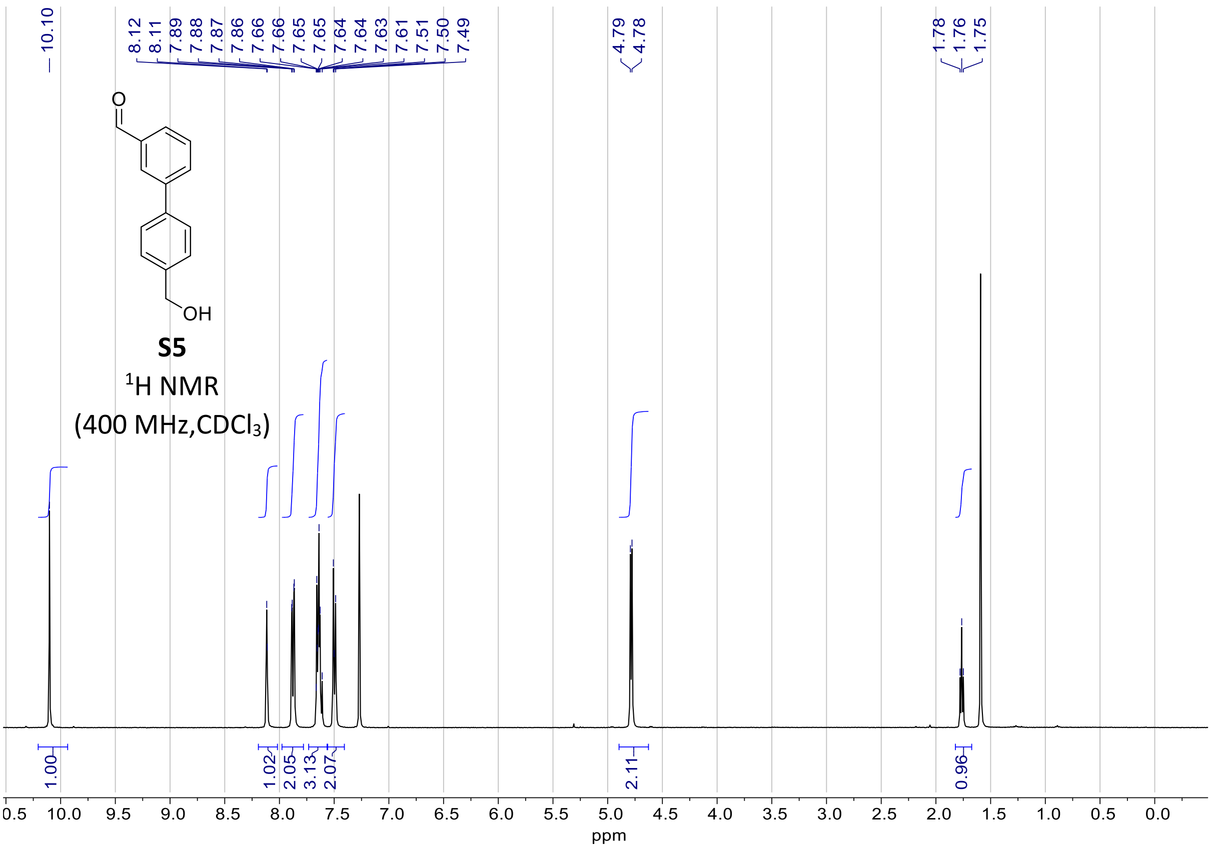
10.10
8.12
8.11
7.89
7.88
7.87
7.86
7.66
7.66
7.65
7.65
7.64
7.64
7.63
7.61
7.51
7.50
7.49
4.79
4.78
1.78
1.76
1.75
O
OH
S5
1H NMR
(400 MHz,CDCl3)
1.00
1.02
2.05
3.13
2.07
2.11
0.96
ppm

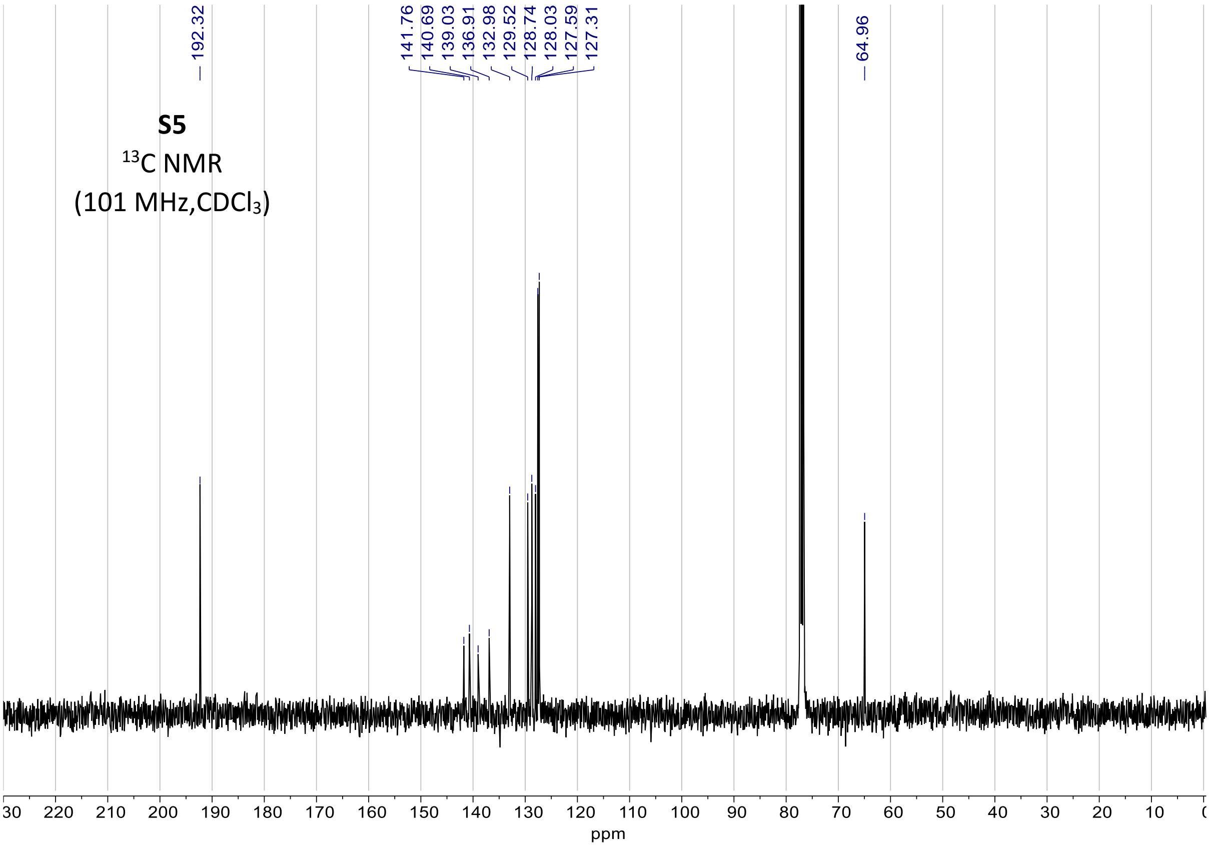
192.32
141.76
140.69
139.03
136.91
132.98
129.52
128.74
128.03
127.59
127.31
64.96
S5
13C NMR
(101 MHz,CDCl3)
ppm

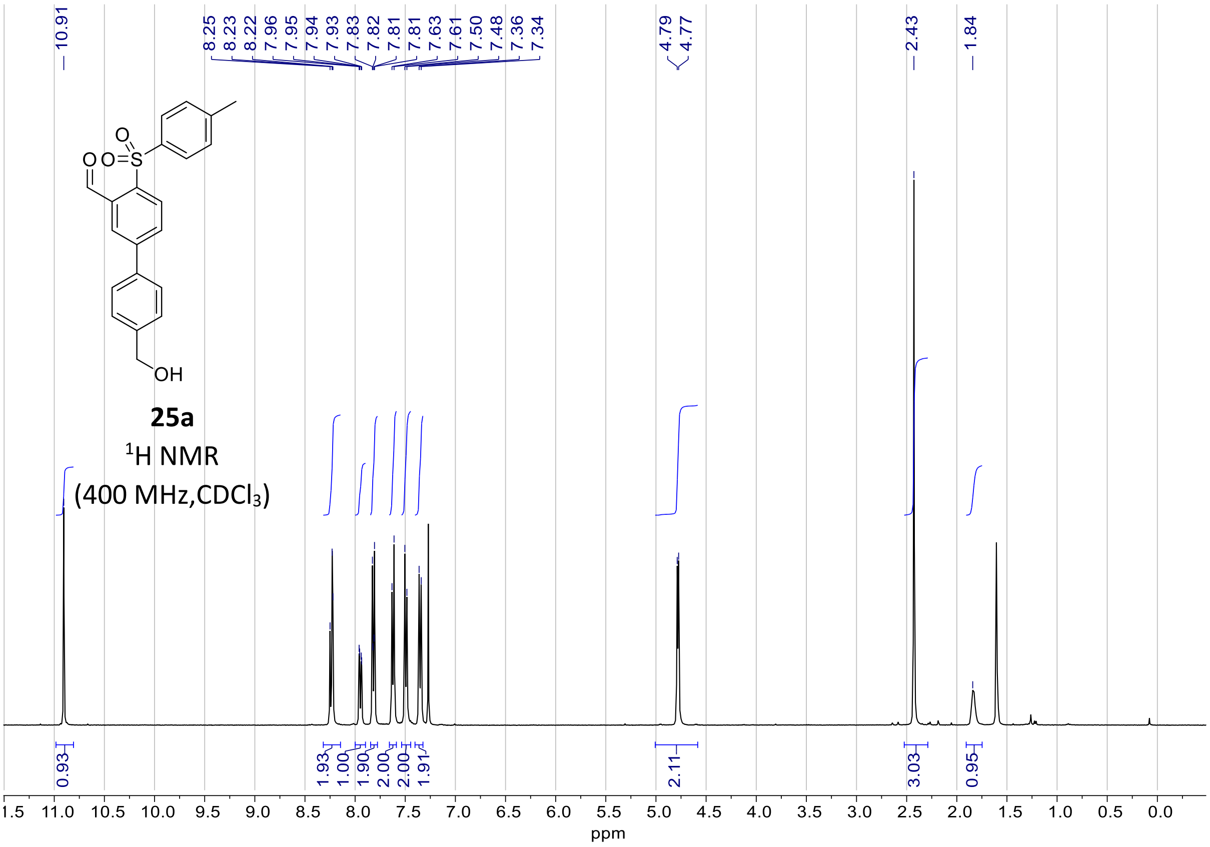

25a

^{1}H NMR

(400 MHz, $CDCl_3$)

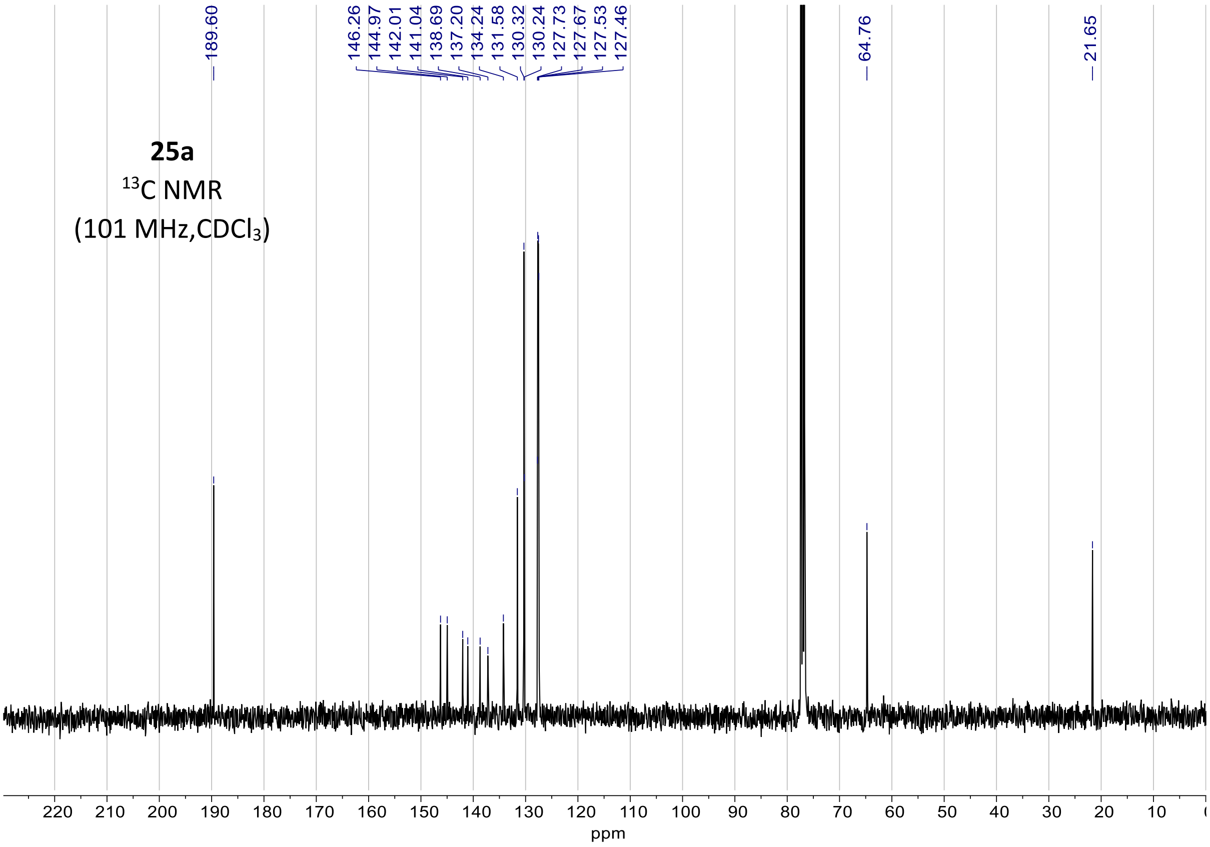

25a

^{13}C NMR

(101 MHz, $CDCl_3$)

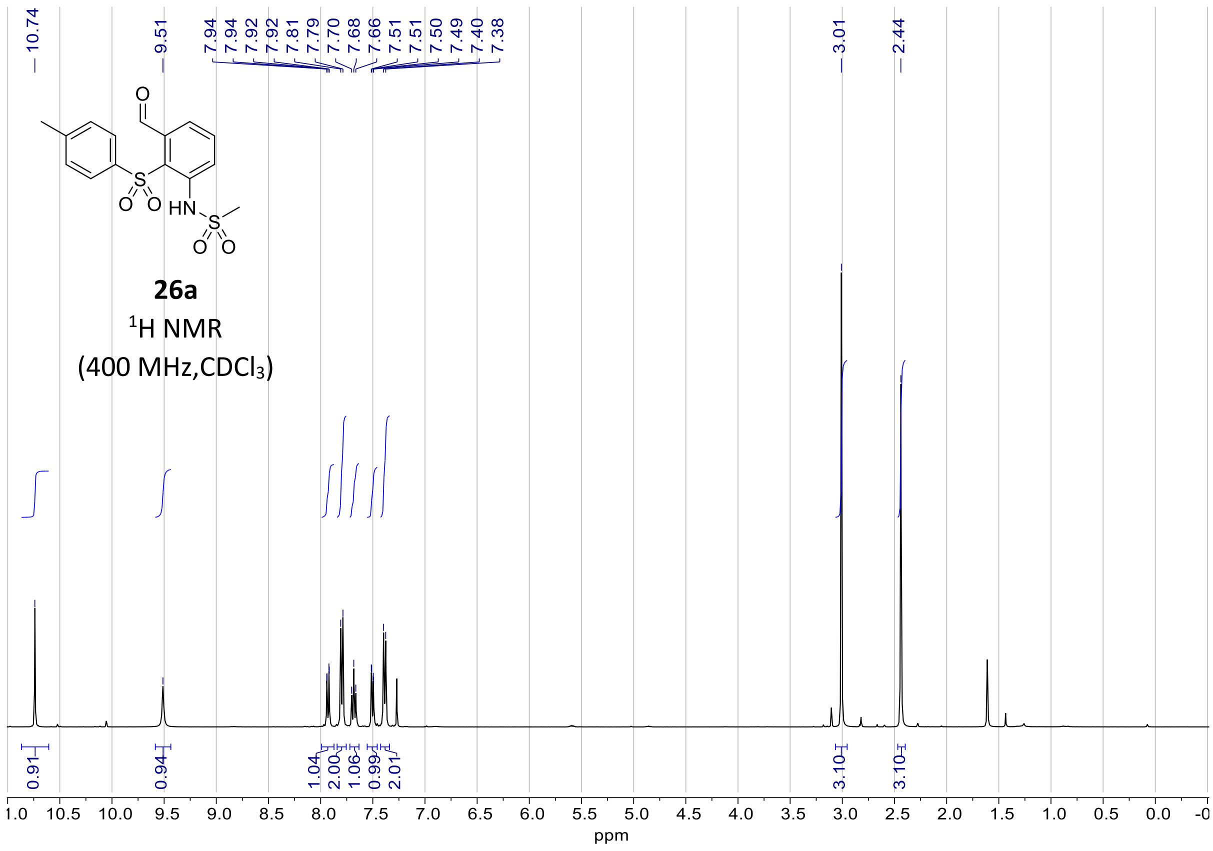
26a
^{1}H NMR
(400 MHz,CDCl$_3$)
ppm

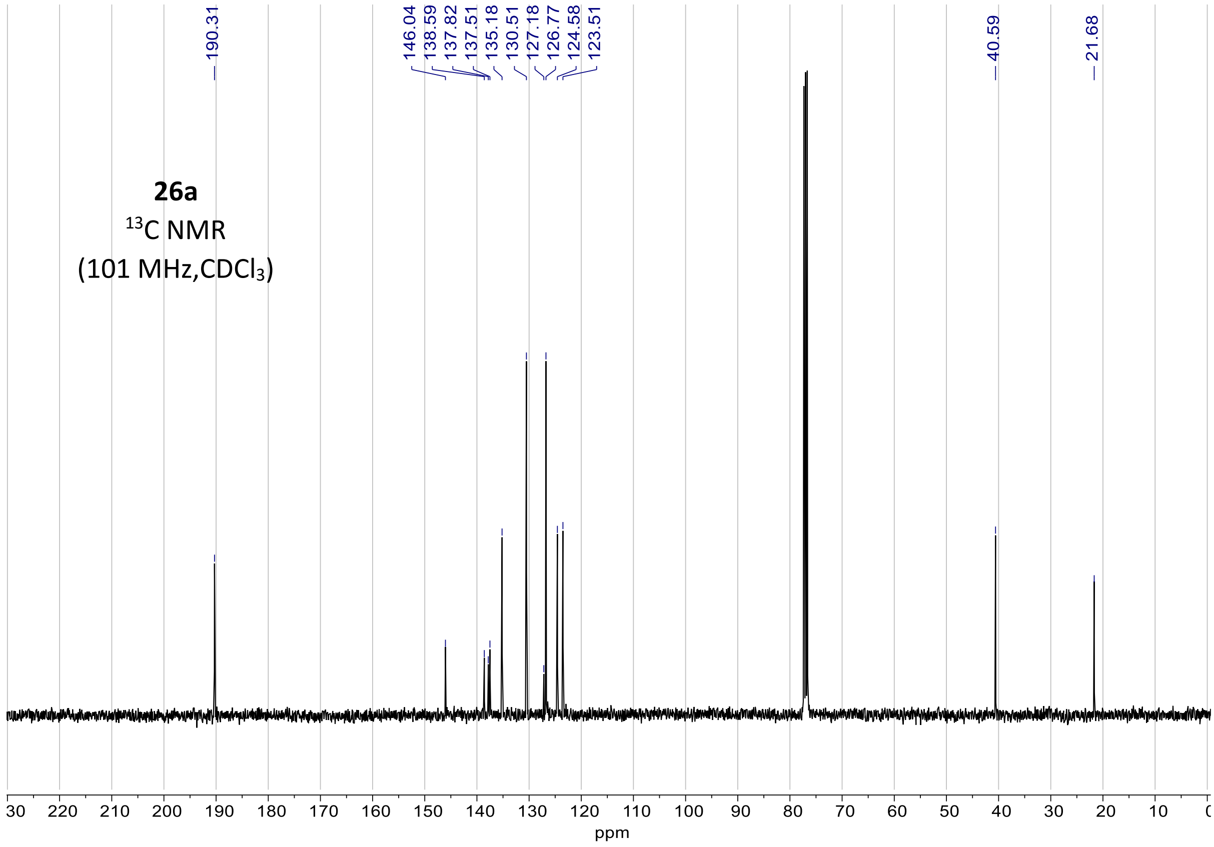
26a
^{13}C NMR
(101 MHz,CDCl$_3$)
ppm

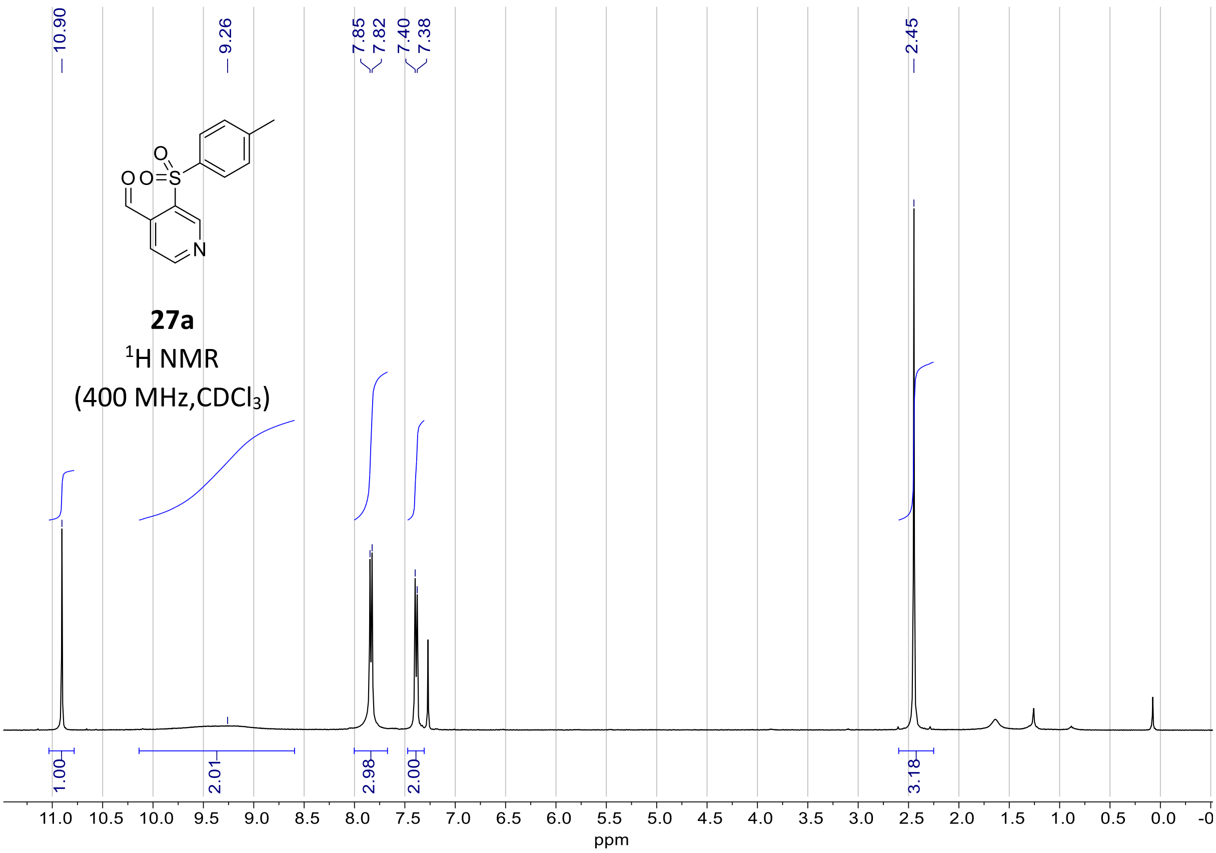

27a

^{1}H NMR

(400 MHz, $CDCl_3$)

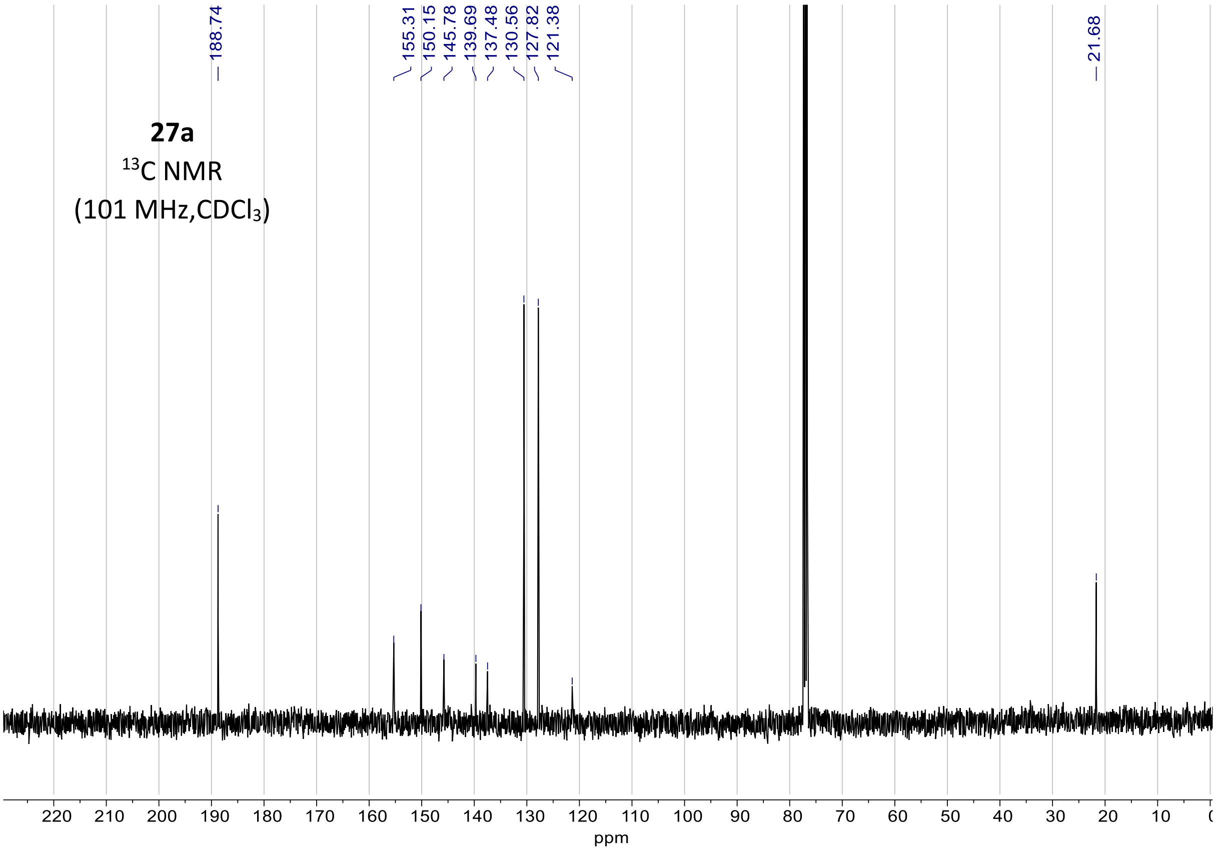

27a

^{13}C NMR

(101 MHz, $CDCl_3$)

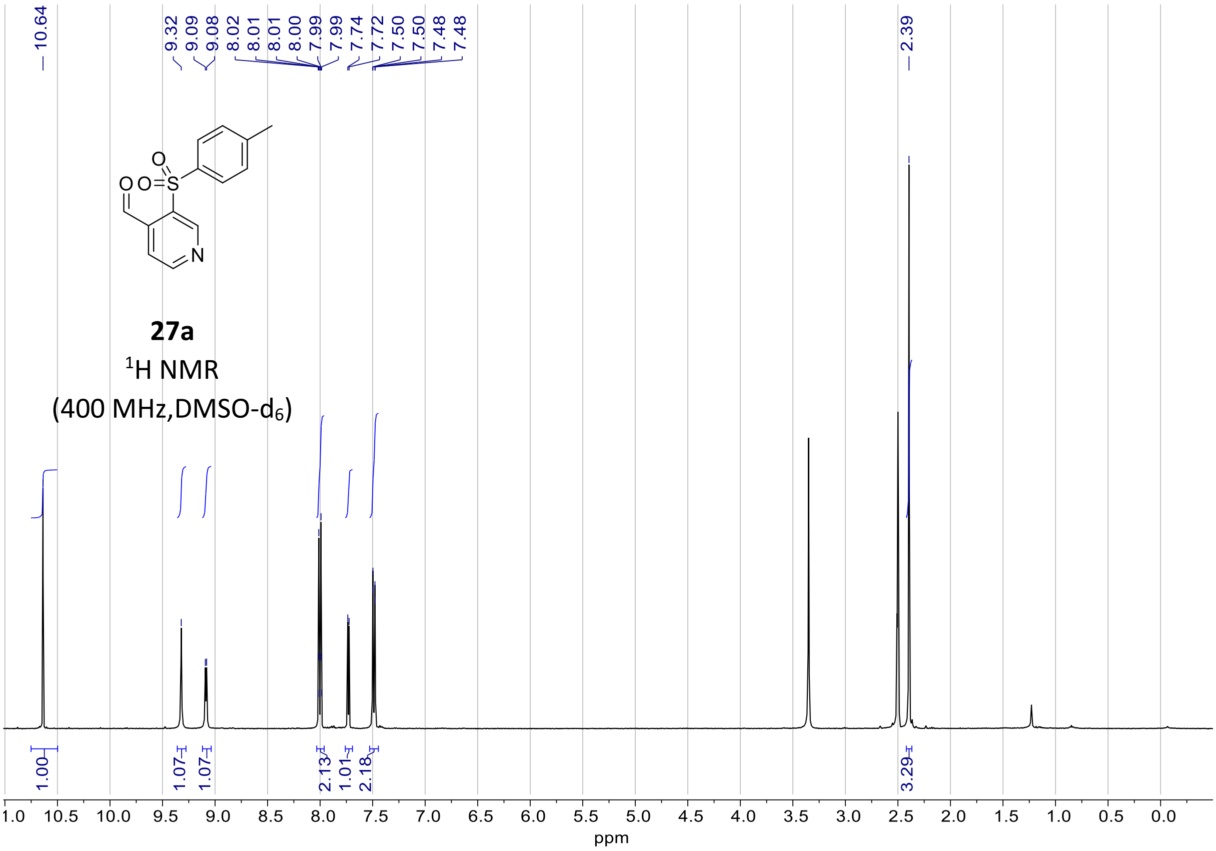

27a
^{1}H NMR
(400 MHz,DMSO-d_6)

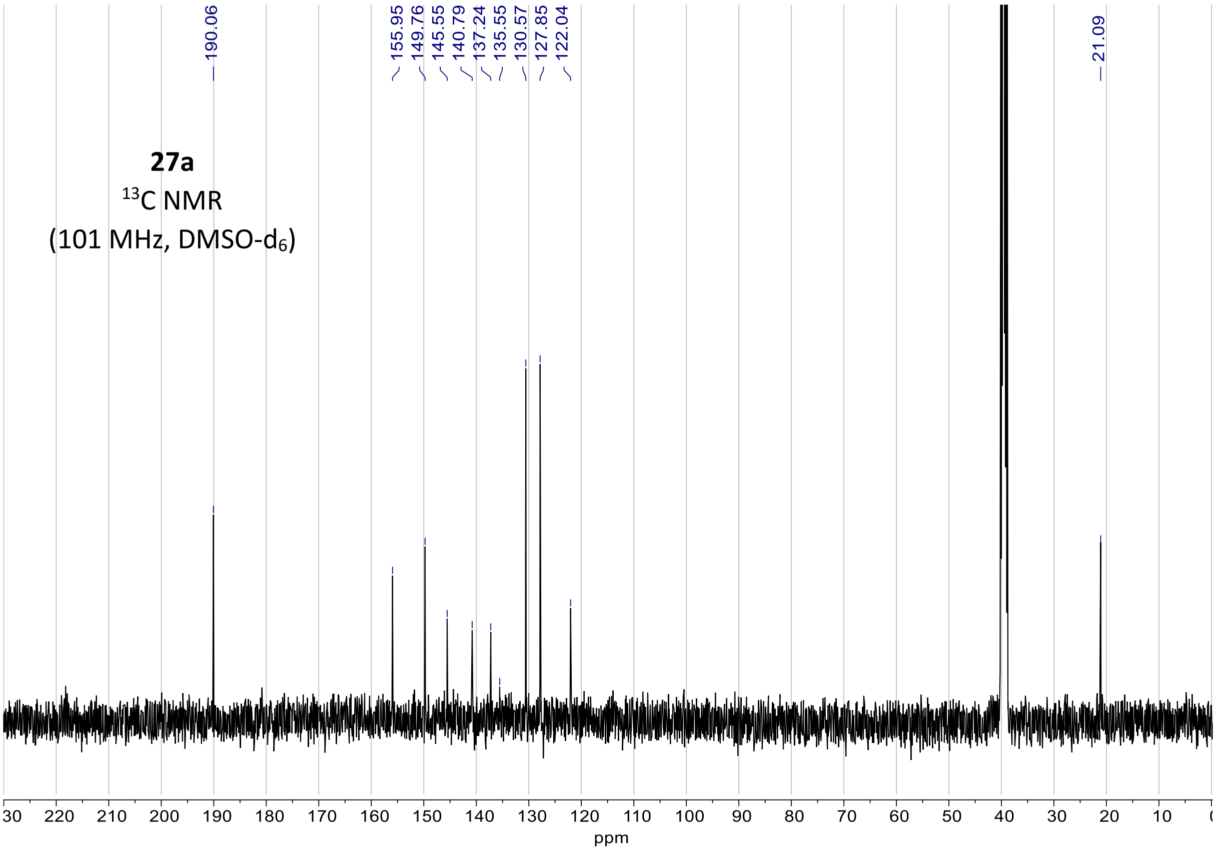

27a
^{13}C NMR
(101 MHz, DMSO-d_6)

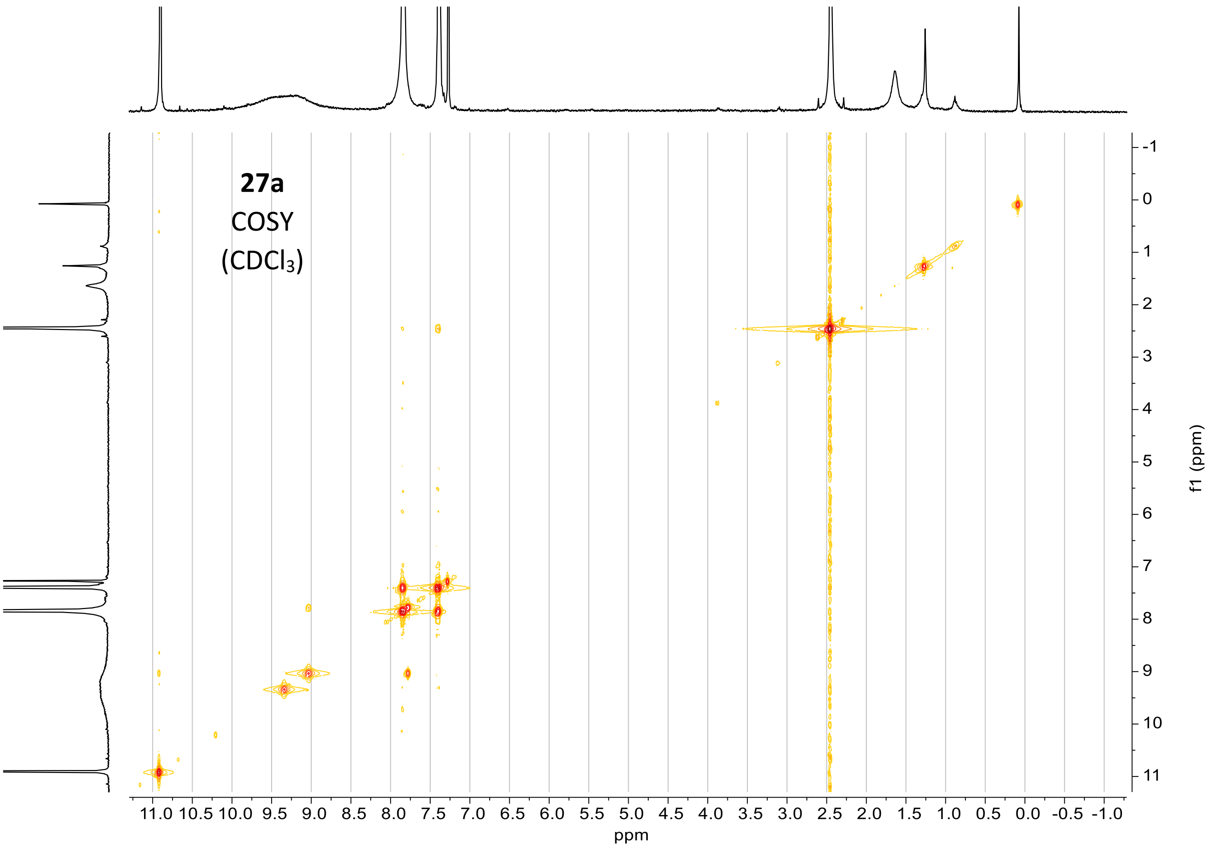
27a
COSY
(CDCl3)
ppm
f1 (ppm)

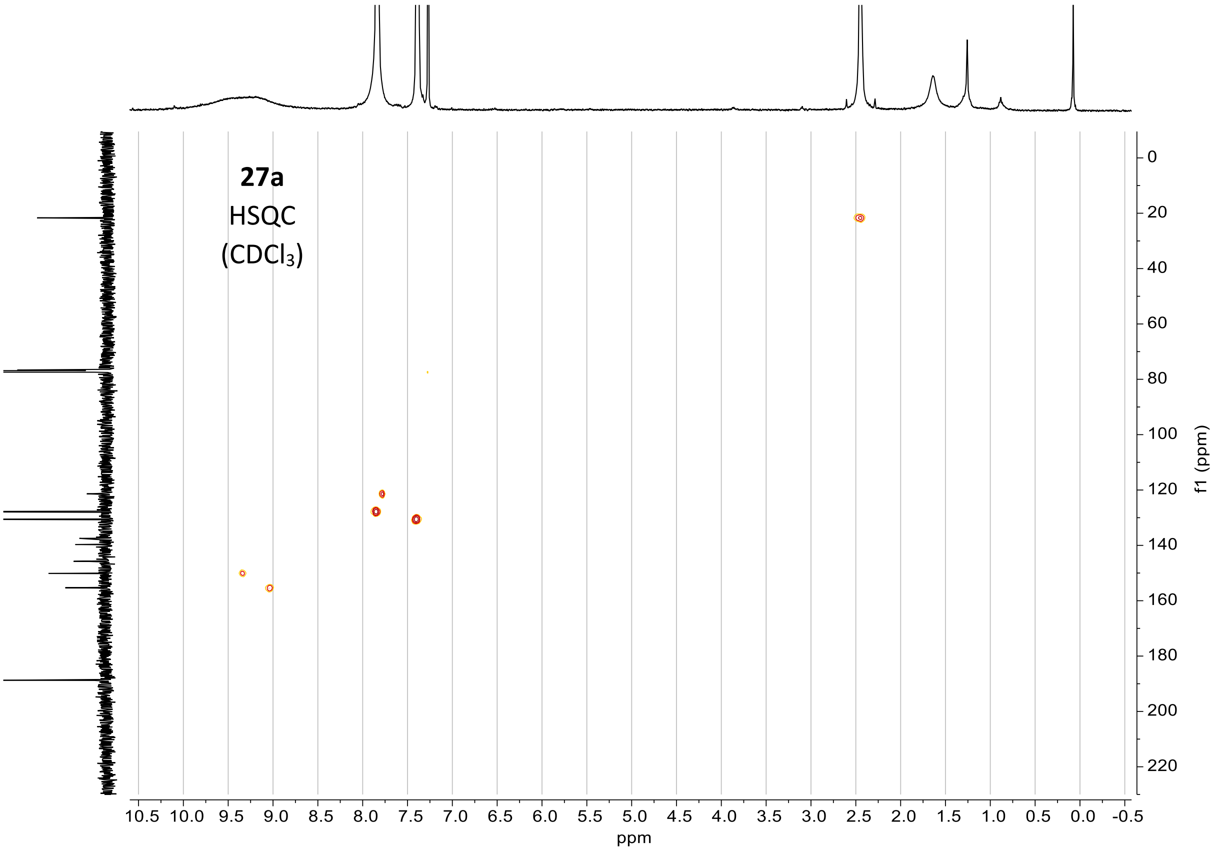
27a
HSQC
(CDCl3)
ppm
f1 (ppm)

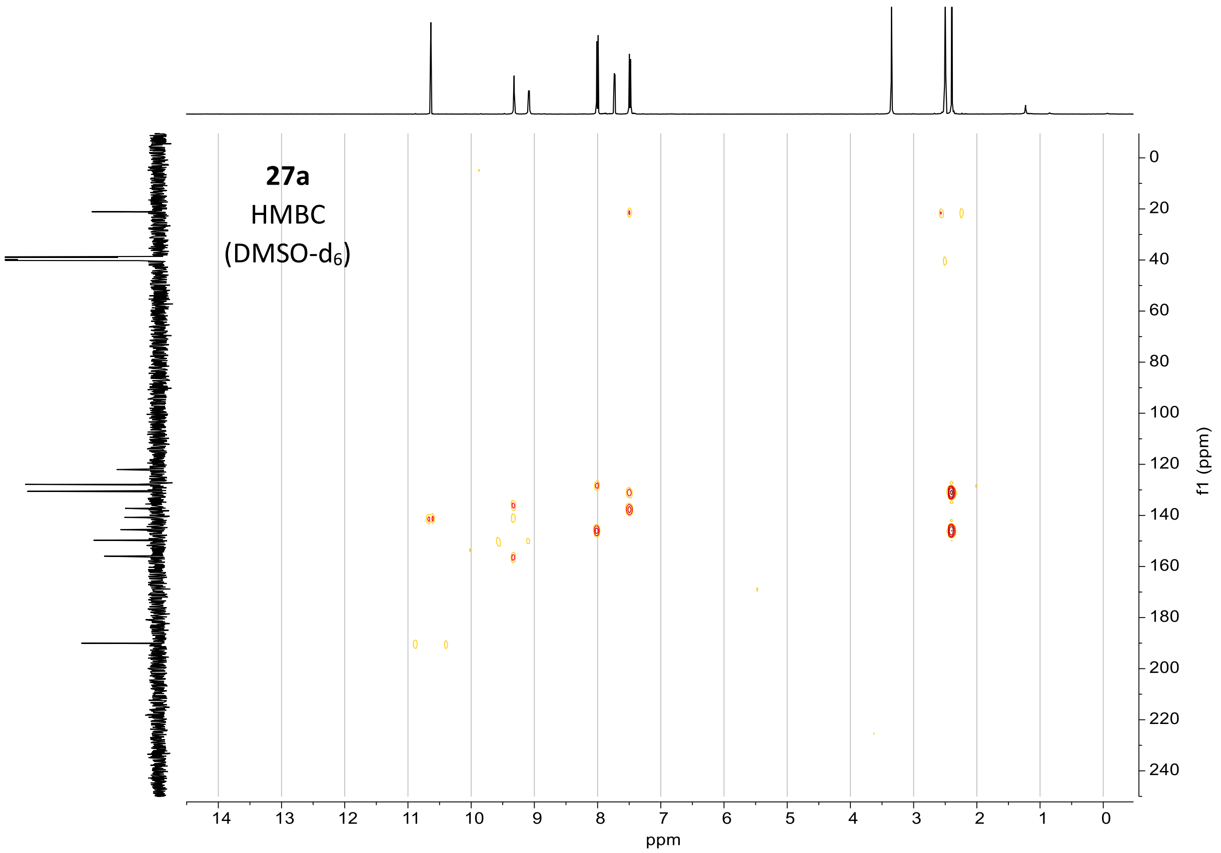
27a
HMBC
(DMSO-d6)
f1 (ppm)
ppm

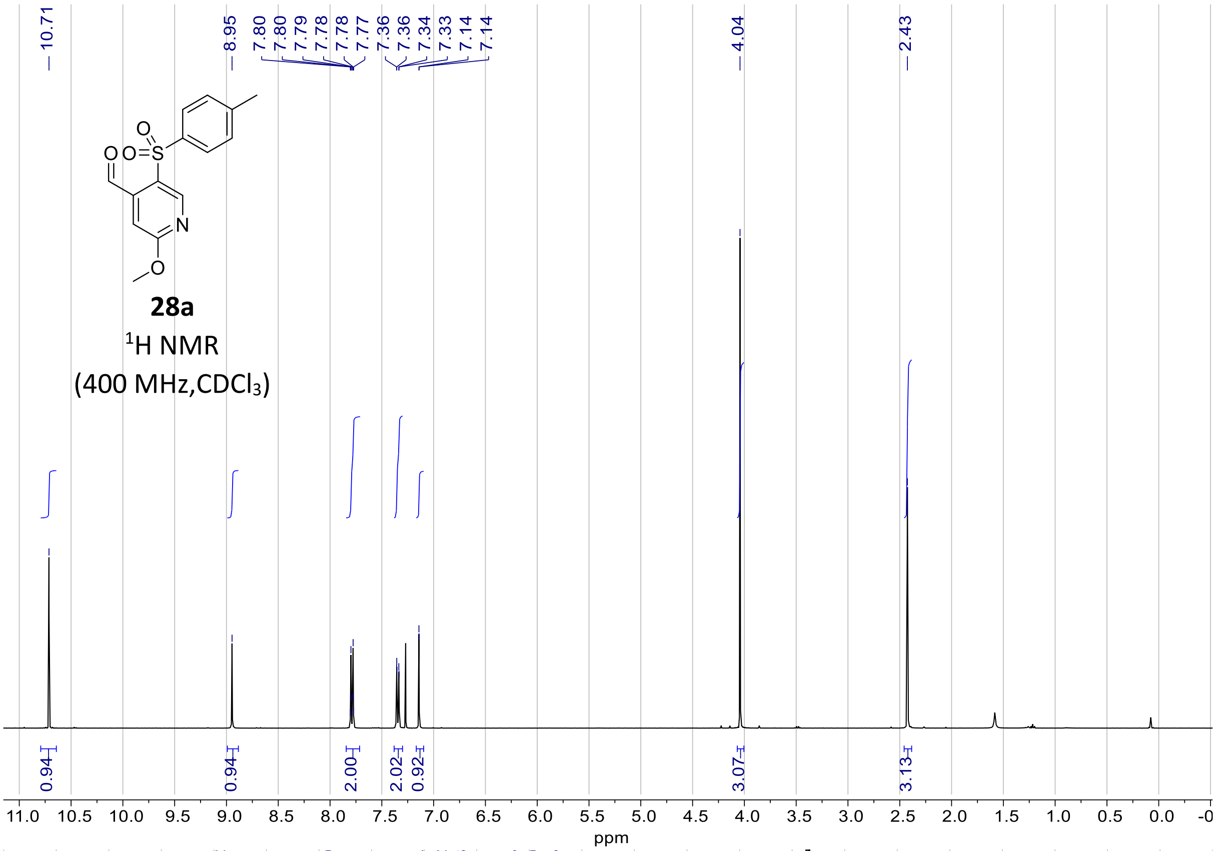
10.71
8.95
7.80
7.80
7.79
7.78
7.78
7.77
7.36
7.36
7.34
7.33
7.14
7.14
4.04
2.43
O
O=S
O
N
O
28a
^{1}H NMR
(400 MHz,CDCl$_3$)
0.94
0.94
2.00
2.02
0.92
3.07
3.13
ppm

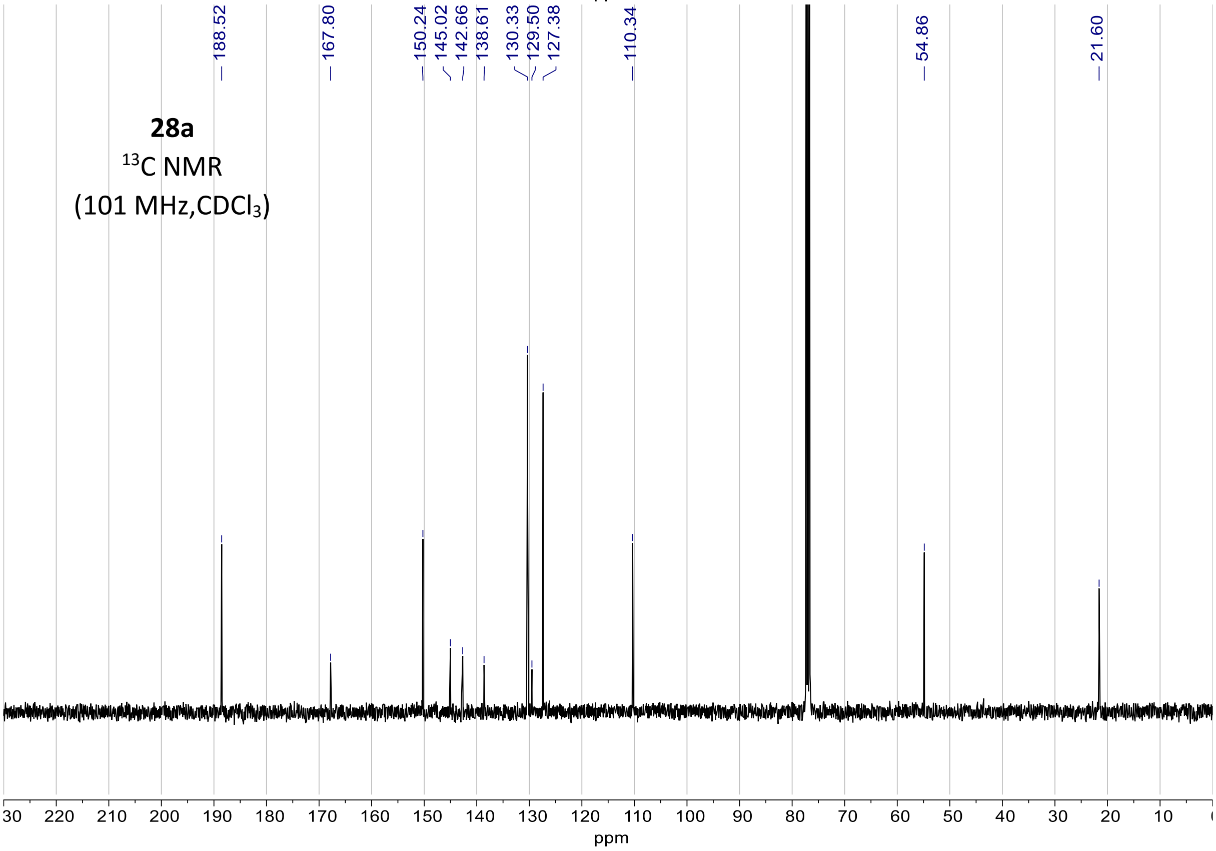
188.52
167.80
150.24
145.02
142.66
138.61
130.33
129.50
127.38
110.34
54.86
21.60
28a
^{13}C NMR
(101 MHz,CDCl$_3$)
ppm

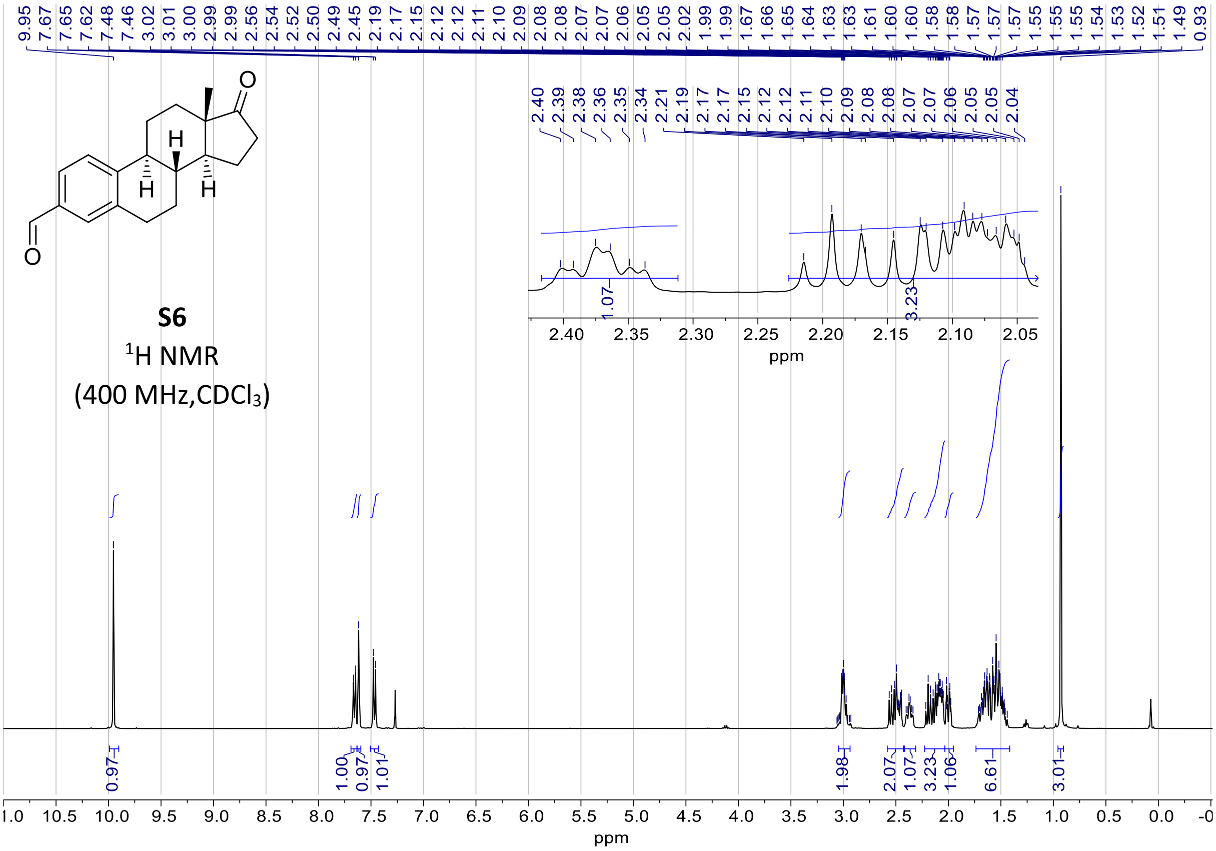
9.95 7.67 7.65 7.62 7.48 7.46 3.02 3.01 3.00 2.99 2.99 2.56 2.54 2.52 2.50 2.49 2.45 2.19 2.17 2.15 2.12 2.12 2.11 2.10 2.09 2.08 2.08 2.07 2.07 2.06 2.05 2.05 2.02 1.99 1.99 1.67 1.66 1.65 1.64 1.63 1.63 1.61 1.60 1.60 1.58 1.58 1.57 1.57 1.57 1.55 1.55 1.54 1.53 1.52 1.51 1.49 0.93
2.40 2.39 2.38 2.36 2.35 2.34 2.21 2.19 2.17 2.17 2.15 2.12 2.12 2.11 2.10 2.09 2.08 2.08 2.07 2.07 2.06 2.05 2.05 2.04
1.07
3.23
ppm
S6
^{1}H NMR
(400 MHz,CDCl$_3$)
0.97 1.00 0.97 1.01 1.98 2.07 1.07 3.23 1.06 6.61 3.01
ppm

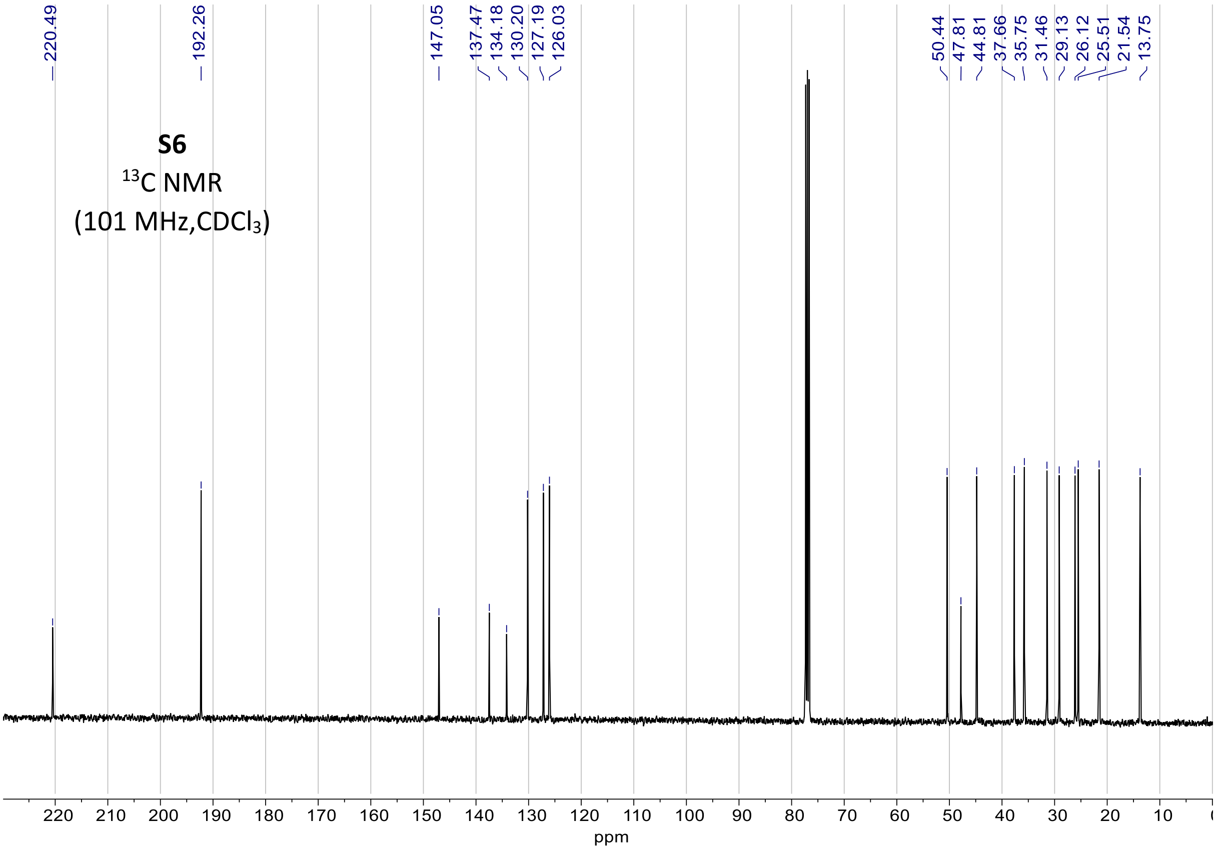
220.49 192.26 147.05 137.47 134.18 130.20 127.19 126.03 50.44 47.81 44.81 37.66 35.75 31.46 29.13 26.12 25.51 21.54 13.75
S6
^{13}C NMR
(101 MHz,CDCl$_3$)
ppm

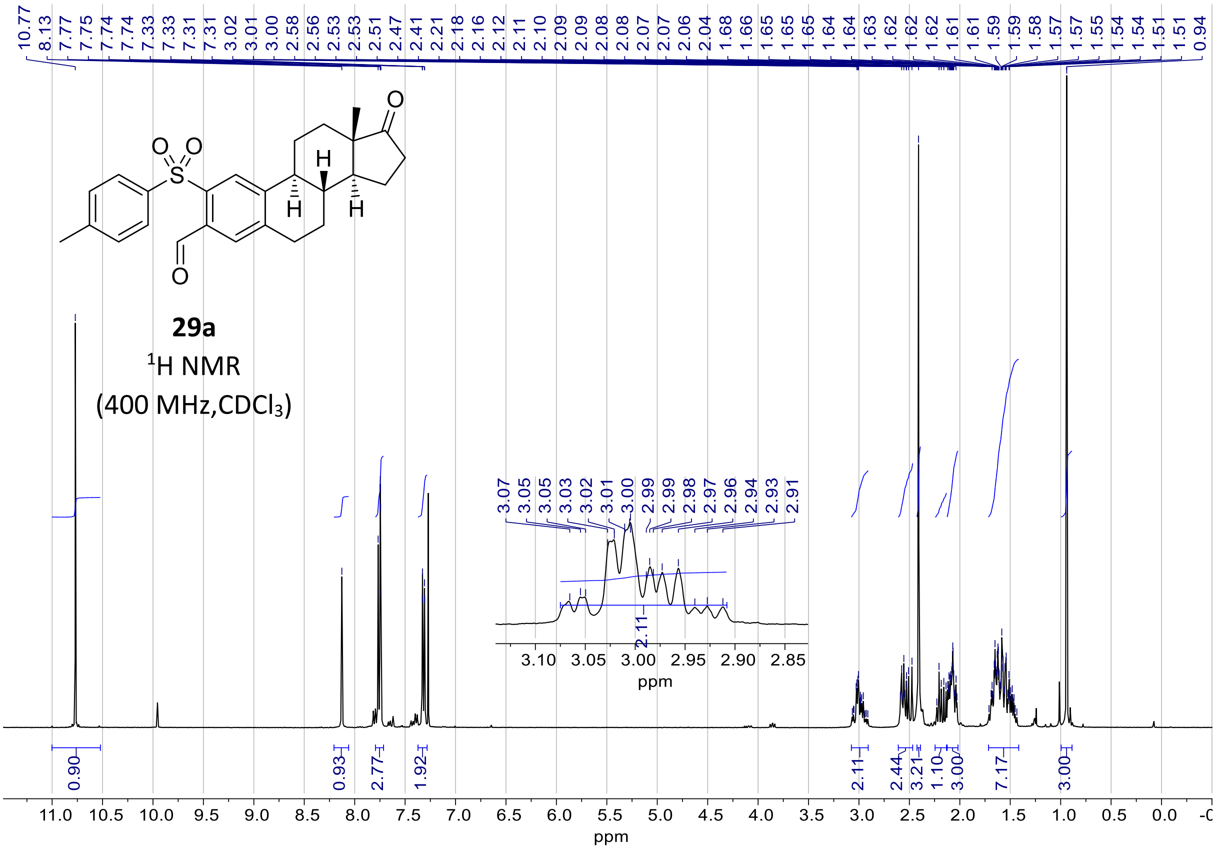
10.77 8.13 7.77 7.75 7.74 7.74 7.33 7.33 7.31 7.31 3.02 3.01 3.00 2.58 2.56 2.53 2.53 2.51 2.47 2.41 2.21 2.18 2.16 2.12 2.11 2.10 2.09 2.09 2.08 2.08 2.07 2.07 2.06 2.04 1.68 1.66 1.65 1.65 1.65 1.64 1.64 1.63 1.62 1.62 1.62 1.61 1.61 1.59 1.59 1.58 1.57 1.57 1.55 1.54 1.54 1.51 1.51 0.94
O
O
O
S
H
H
H
O
29a
1H NMR
(400 MHz,CDCl3)
3.07 3.05 3.05 3.03 3.02 3.01 3.00 2.99 2.99 2.98 2.97 2.96 2.94 2.93 2.91
2.11
3.10 3.05 3.00 2.95 2.90 2.85
ppm
0.90
0.93
2.77
1.92
2.11
2.44
3.21
1.10
3.00
7.17
3.00
11.0 10.5 10.0 9.5 9.0 8.5 8.0 7.5 7.0 6.5 6.0 5.5 5.0 4.5 4.0 3.5 3.0 2.5 2.0 1.5 1.0 0.5 0.0
ppm

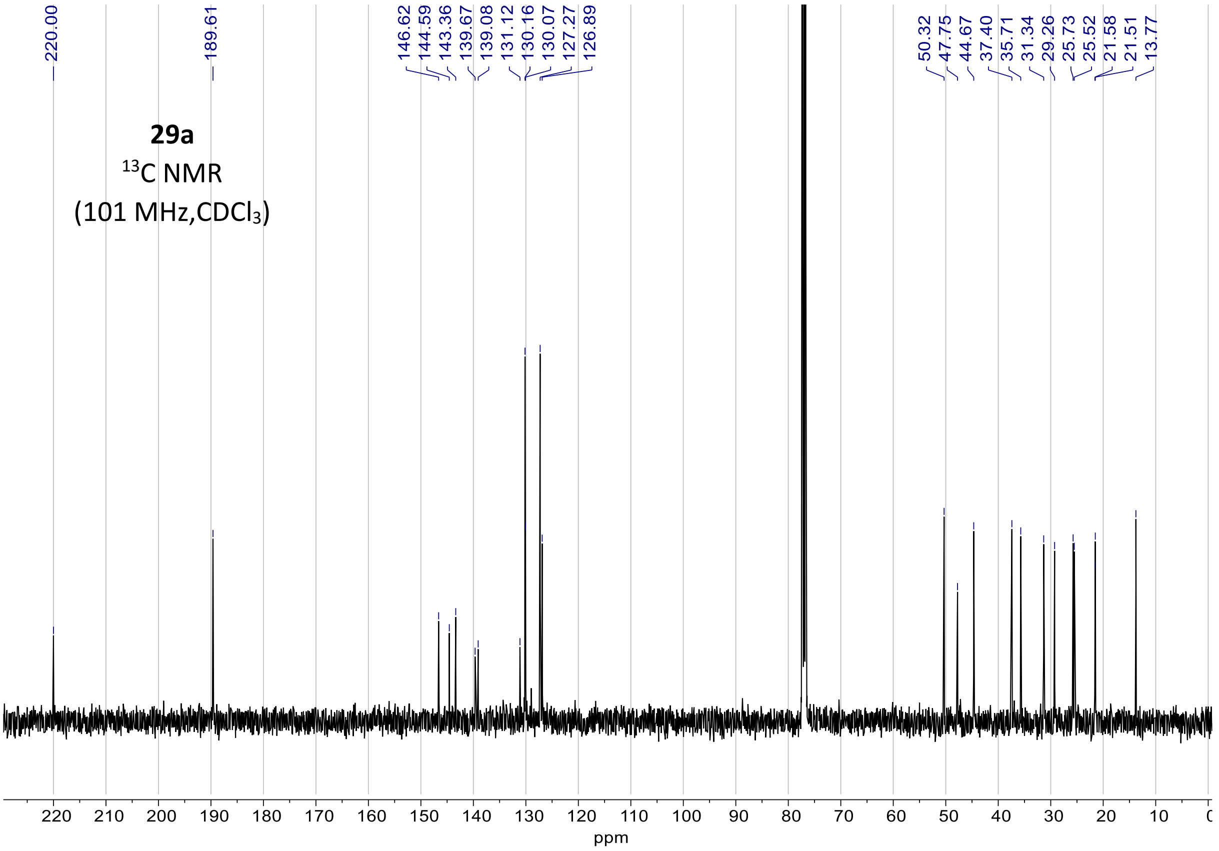
220.00
189.61
146.62 144.59 143.36 139.67 139.08 131.12 130.16 130.07 127.27 126.89
50.32 47.75 44.67 37.40 35.71 31.34 29.26 25.73 25.52 21.58 21.51 13.77
29a
13C NMR
(101 MHz,CDCl3)
220 210 200 190 180 170 160 150 140 130 120 110 100 90 80 70 60 50 40 30 20 10
ppm

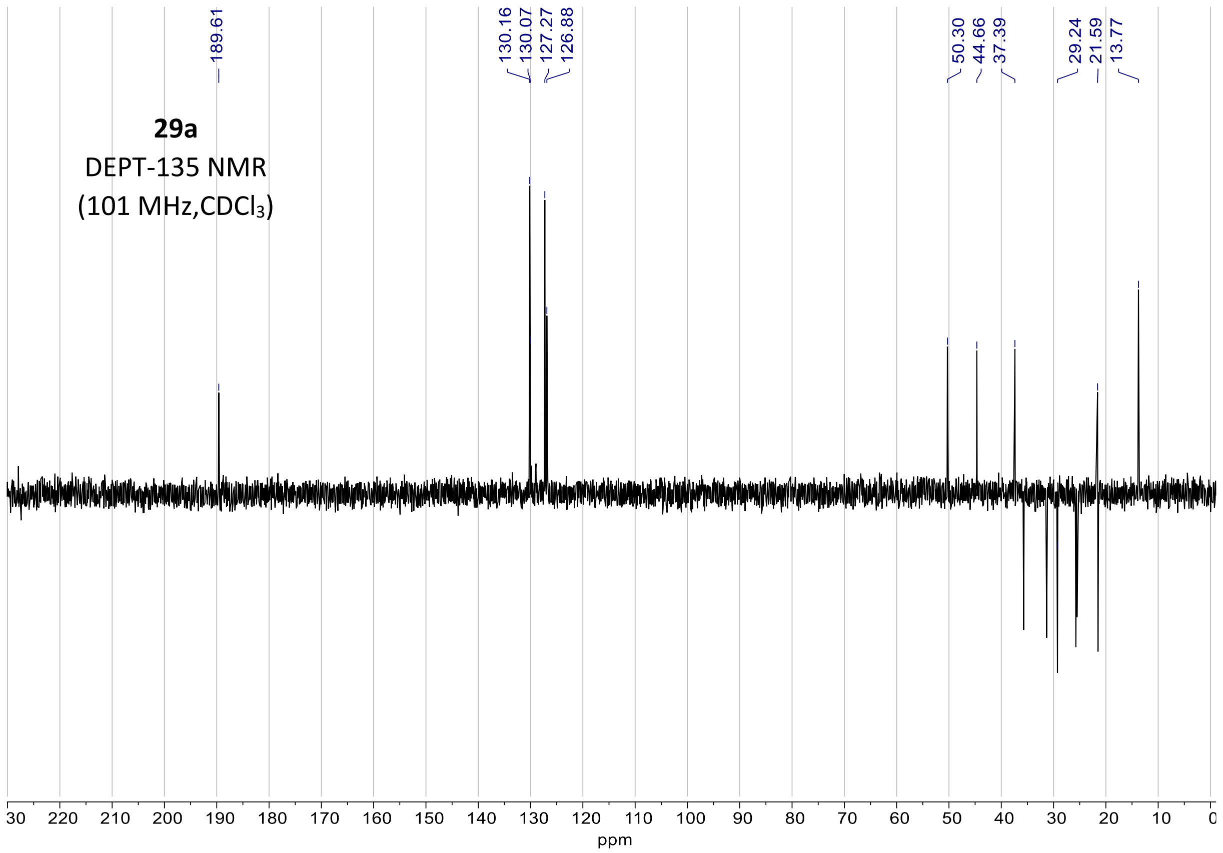

189.61
130.16
130.07
127.27
126.88
50.30
44.66
37.39
29.24
21.59
13.77
29a
DEPT-135 NMR
(101 MHz,CDCl3)
30
220
210
200
190
180
170
160
150
140
130
120
110
100
90
80
70
60
50
40
30
20
10
0
ppm

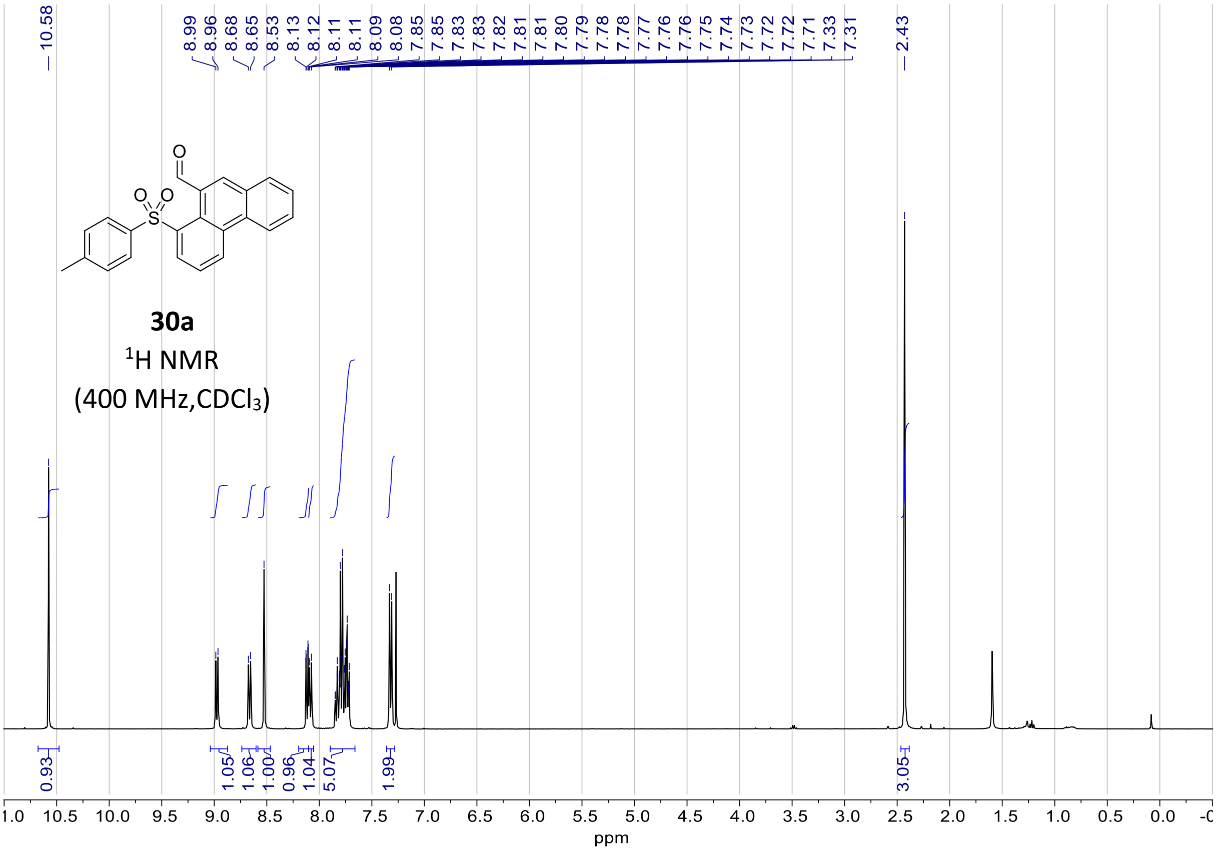
30a
^{1}H NMR
(400 MHz,CDCl$_3$)
ppm

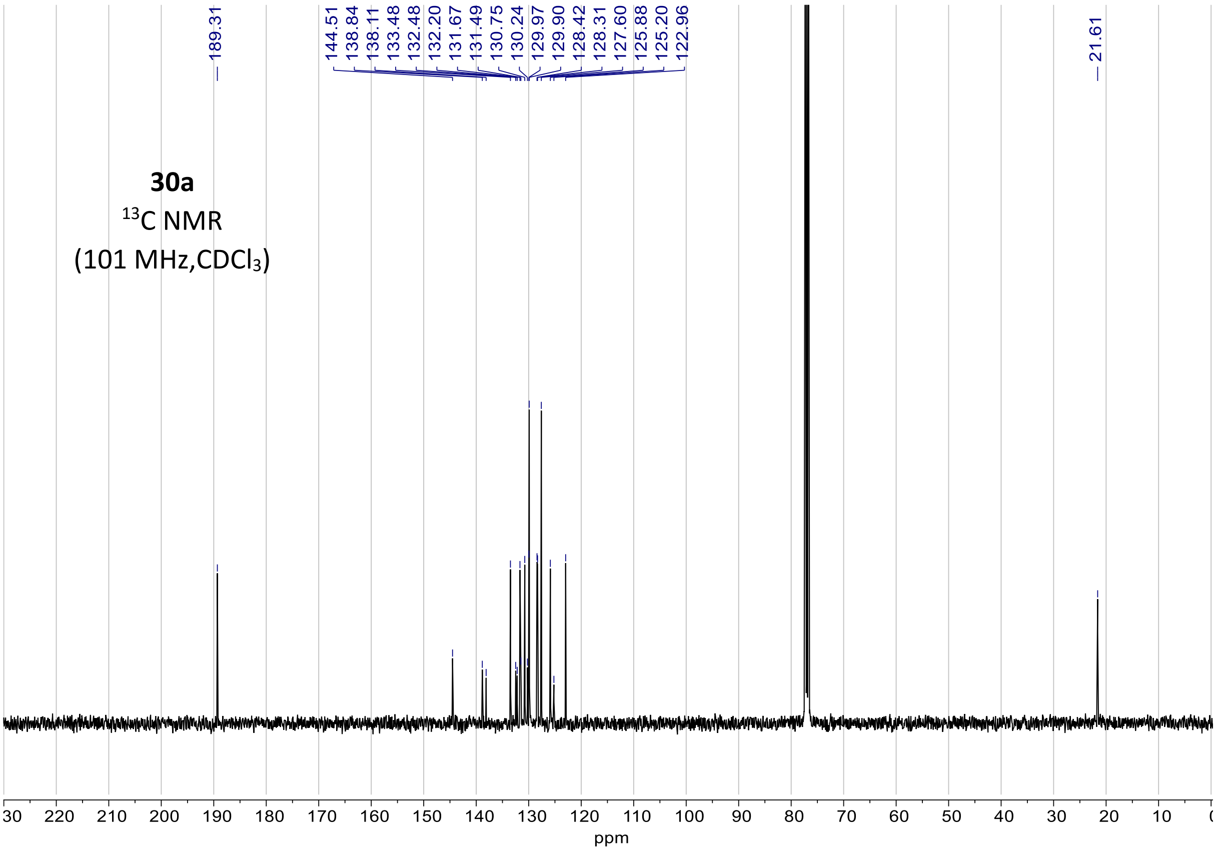
30a
^{13}C NMR
(101 MHz,CDCl$_3$)
ppm

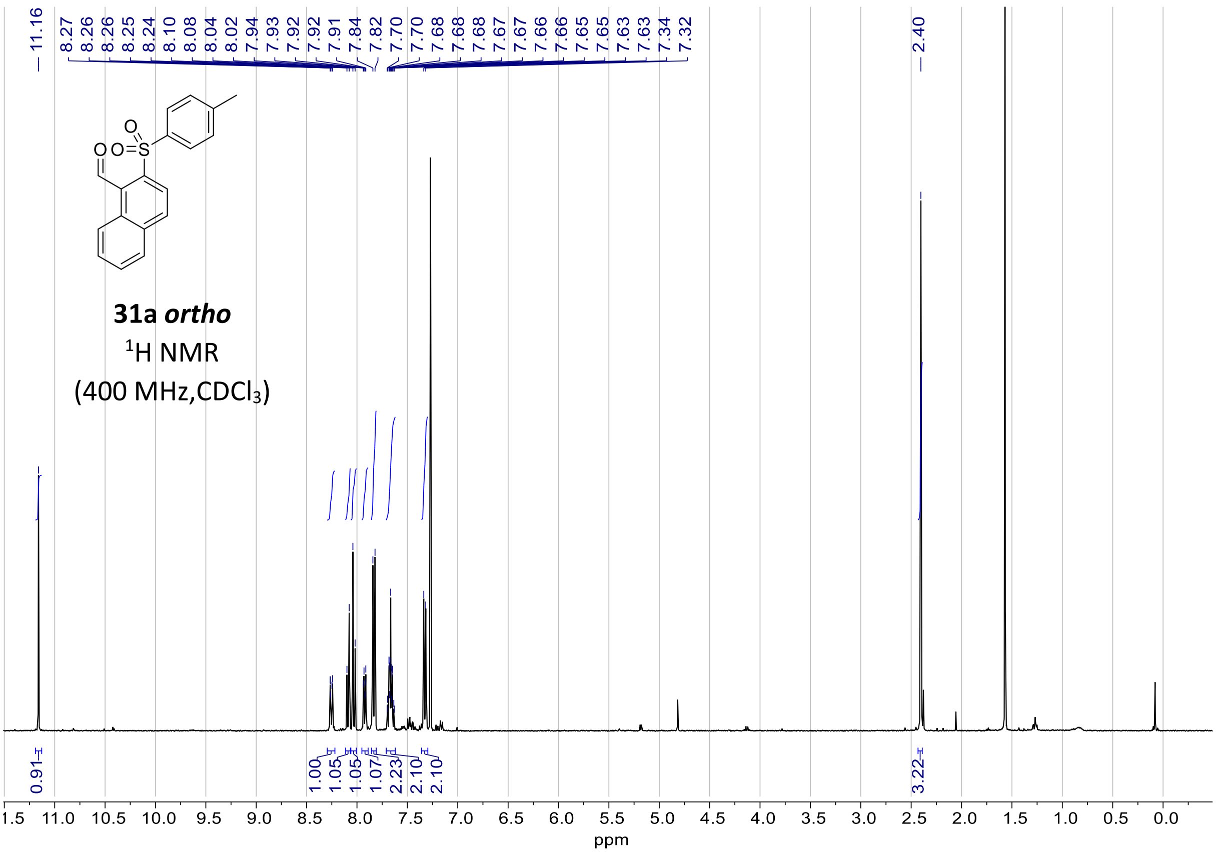
11.16
8.27
8.26
8.26
8.25
8.24
8.10
8.08
8.04
8.02
7.94
7.93
7.92
7.92
7.91
7.84
7.82
7.70
7.70
7.68
7.68
7.67
7.66
7.66
7.65
7.65
7.63
7.63
7.34
7.32
2.40
31a ortho
1H NMR
(400 MHz,CDCl3)
0.91
1.00
1.05
1.05
1.07
2.23
2.10
2.10
3.22
ppm

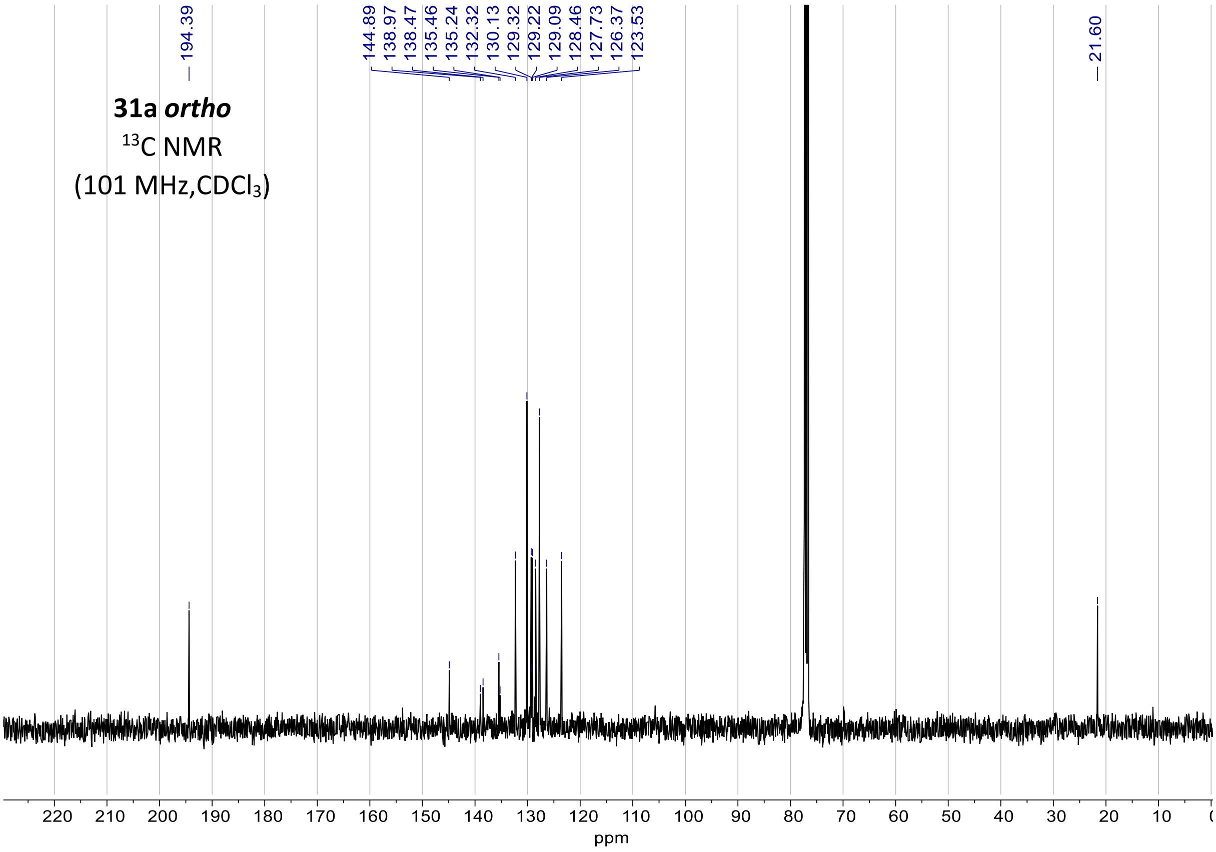
194.39
144.89
138.97
138.47
135.46
135.24
132.32
130.13
129.32
129.22
129.09
128.46
127.73
126.37
123.53
21.60
31a ortho
13C NMR
(101 MHz,CDCl3)
ppm

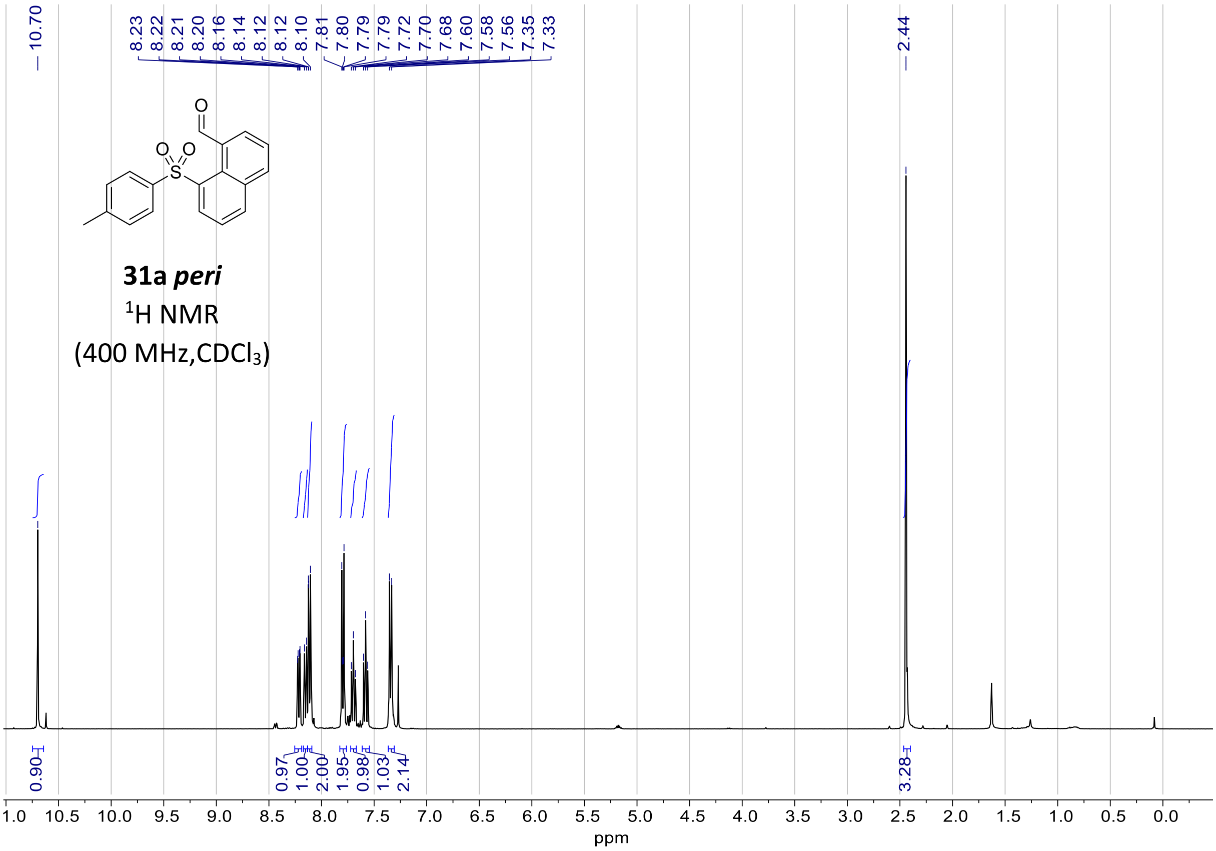
10.70
8.23 8.22 8.21 8.20 8.16 8.14 8.12 8.12 8.10 7.81 7.80 7.79 7.79 7.72 7.70 7.68 7.60 7.58 7.56 7.35 7.33
2.44
31a *peri*
^{1}H NMR
(400 MHz,$CDCl_3$)
0.90 0.97 1.00 2.00 1.95 0.98 1.03 2.14 3.28
ppm

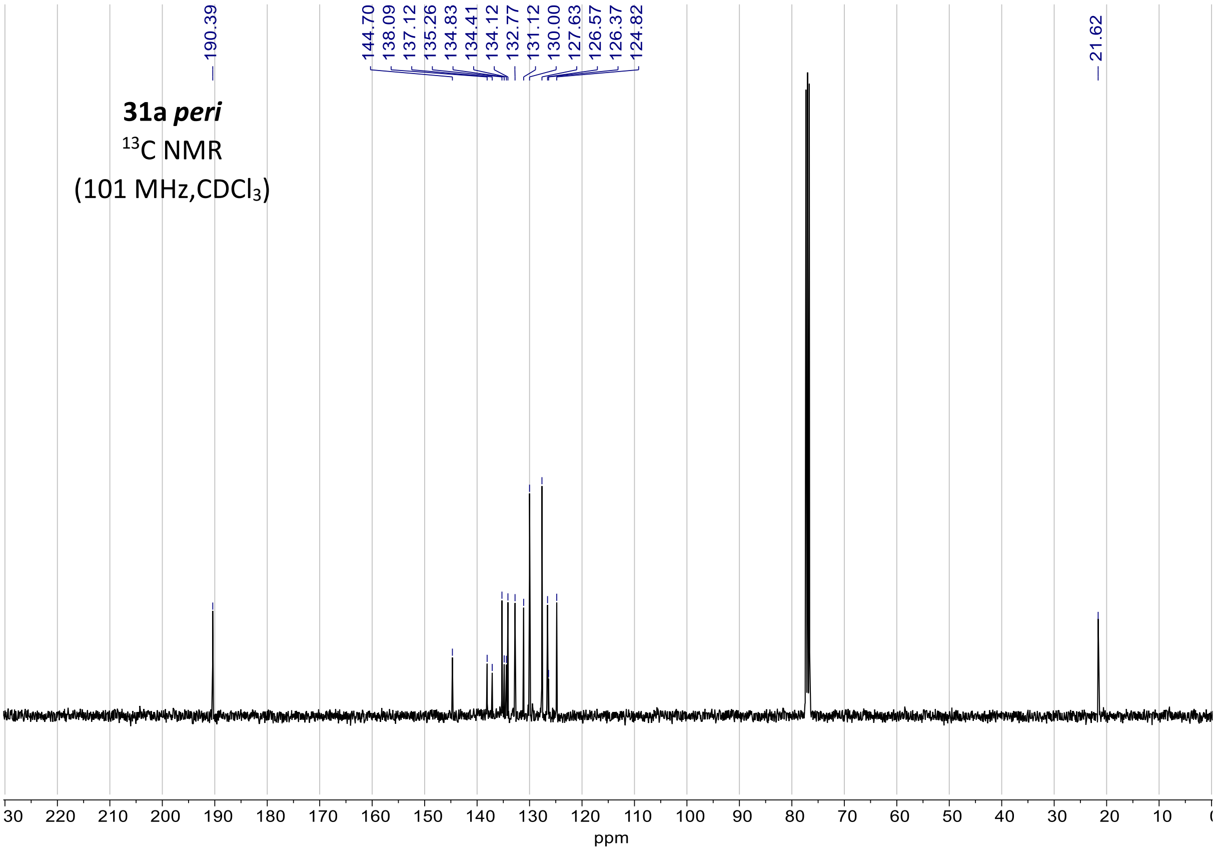
190.39
144.70 138.09 137.12 135.26 134.83 134.41 134.12 132.77 131.12 130.00 127.63 126.57 126.37 124.82
21.62
31a *peri*
^{13}C NMR
(101 MHz,$CDCl_3$)
ppm

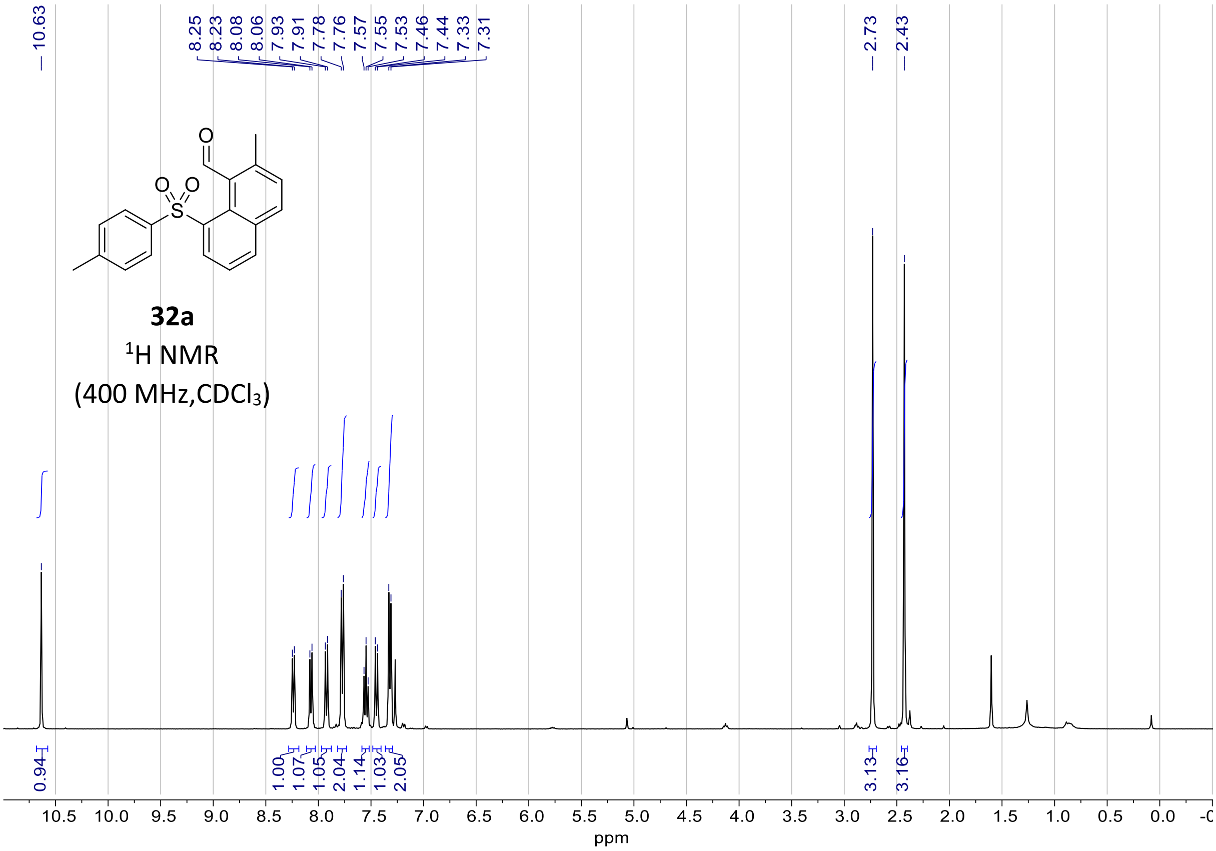

32a
^{1}H NMR
(400 MHz,$CDCl_3$)

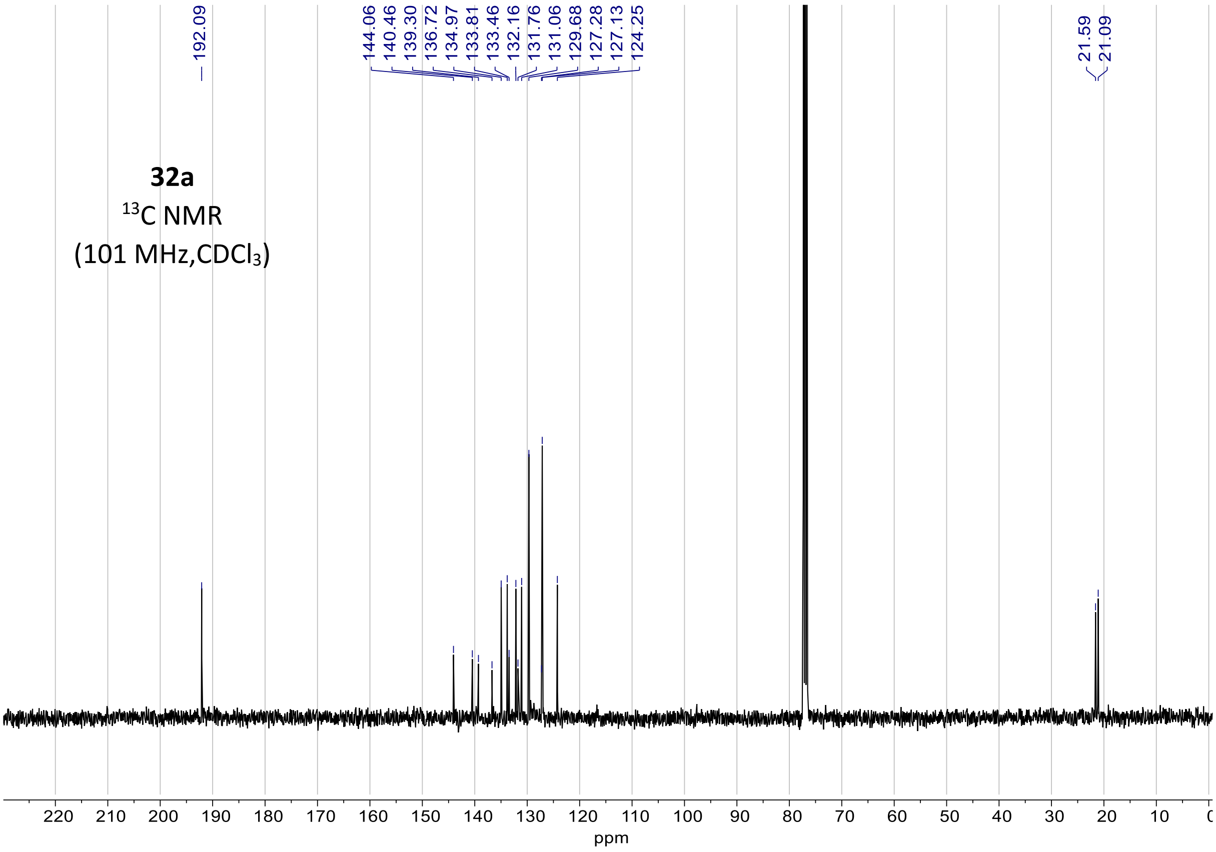

32a
^{13}C NMR
(101 MHz,$CDCl_3$)

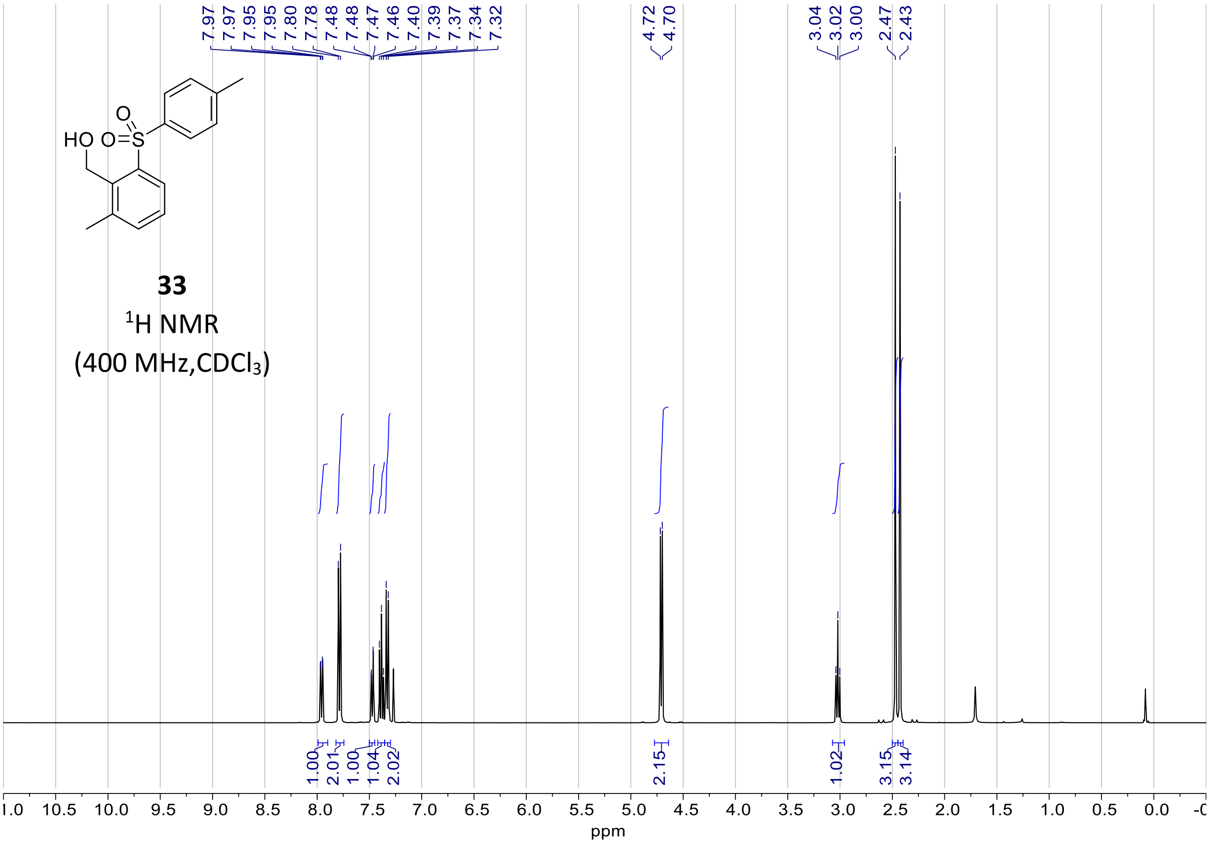
33
^{1}H NMR
(400 MHz,CDCl$_3$)
7.97 7.97 7.95 7.95 7.80 7.78 7.48 7.48 7.47 7.46 7.40 7.39 7.37 7.34 7.32
4.72 4.70
3.04 3.02 3.00 2.47 2.43
1.00 2.01 1.00 1.04 2.02 2.15 1.02 3.15 3.14
ppm

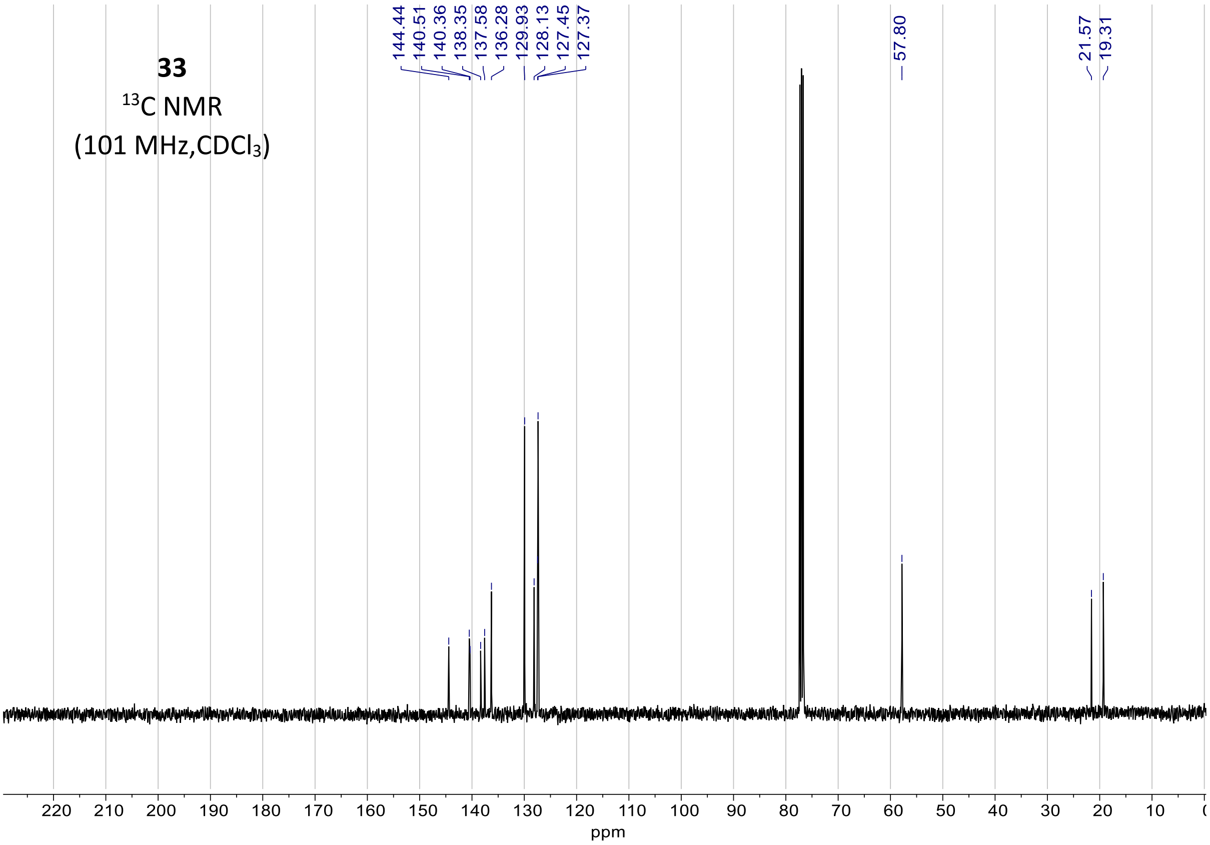
33
^{13}C NMR
(101 MHz,CDCl$_3$)
144.44 140.51 140.36 138.35 137.58 136.28 129.93 128.13 127.45 127.37
57.80
21.57 19.31
ppm

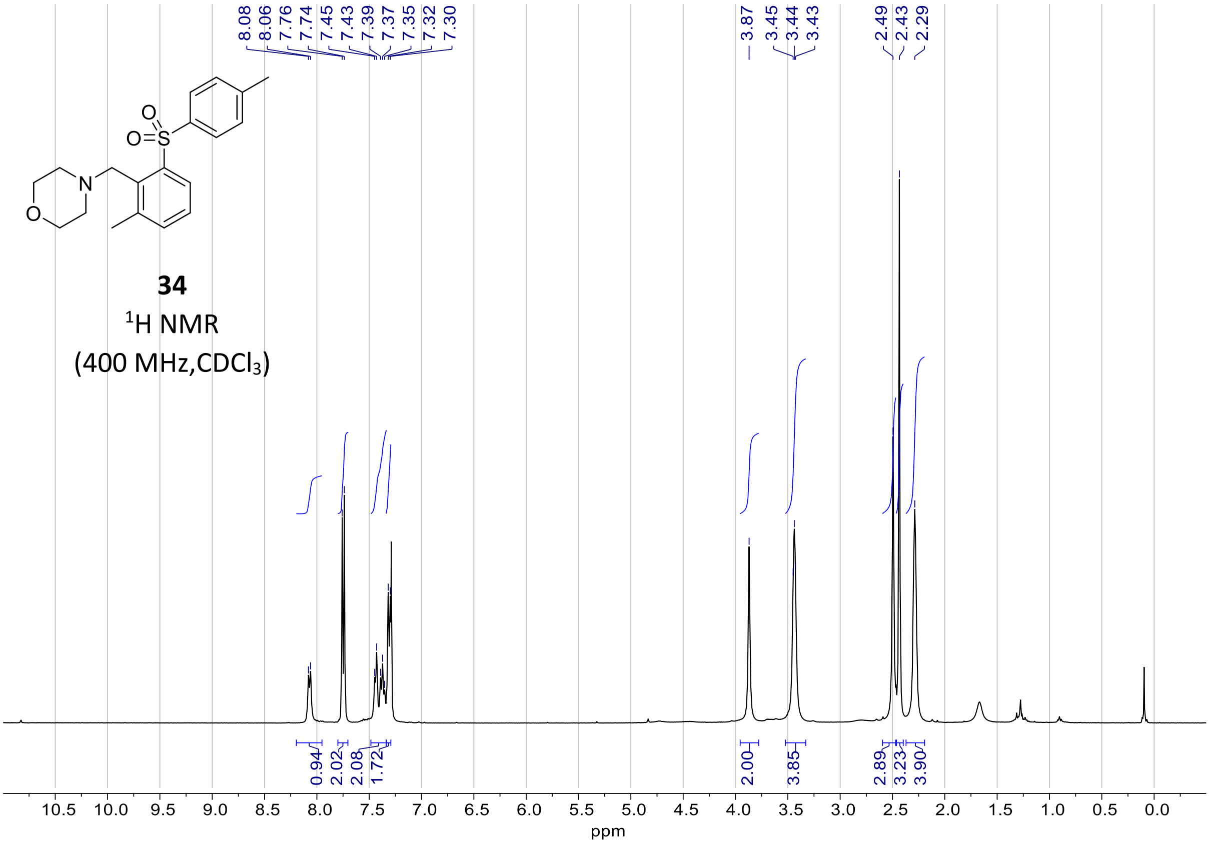
34
^{1}H NMR
(400 MHz, $CDCl_3$)
ppm

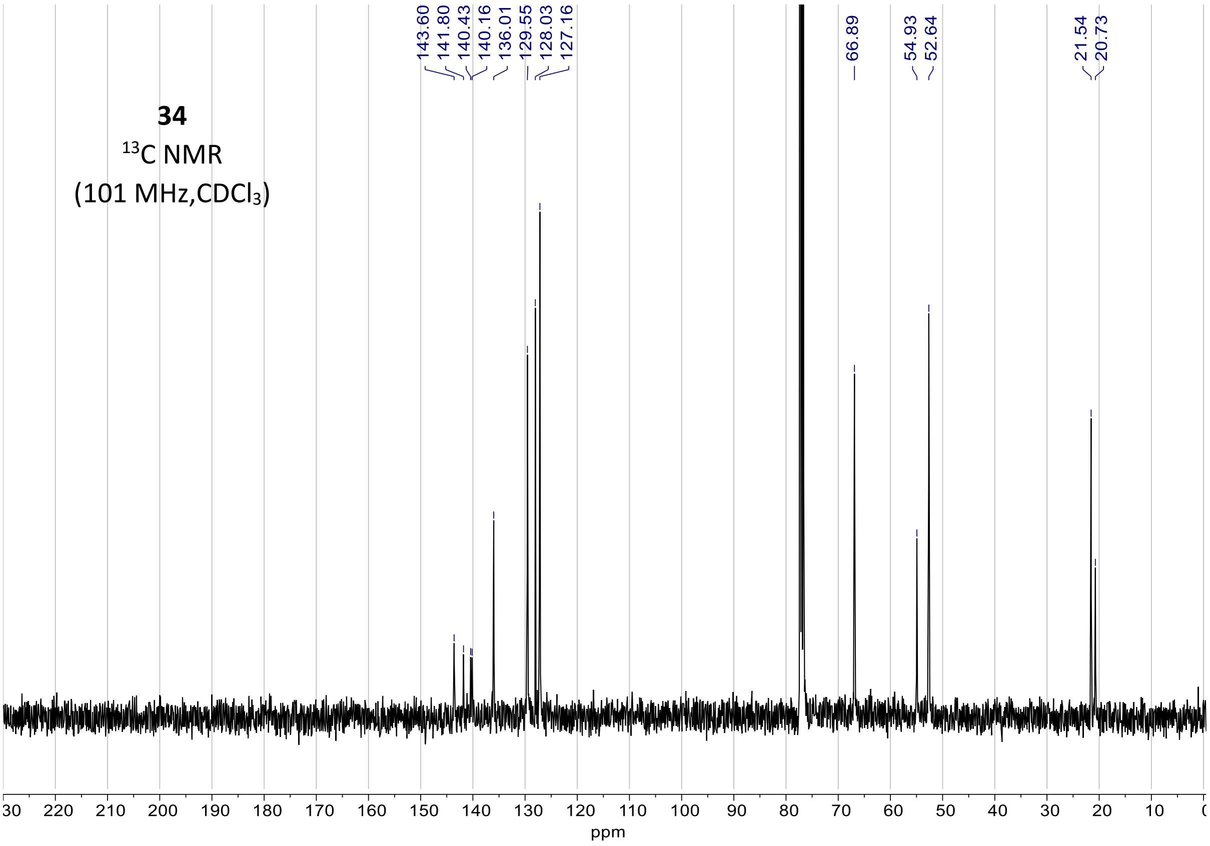
34
^{13}C NMR
(101 MHz, $CDCl_3$)
ppm

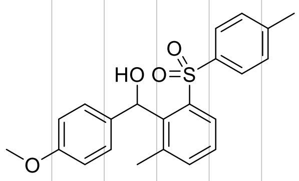

35

^{1}H NMR

(400 MHz, $CDCl_3$)

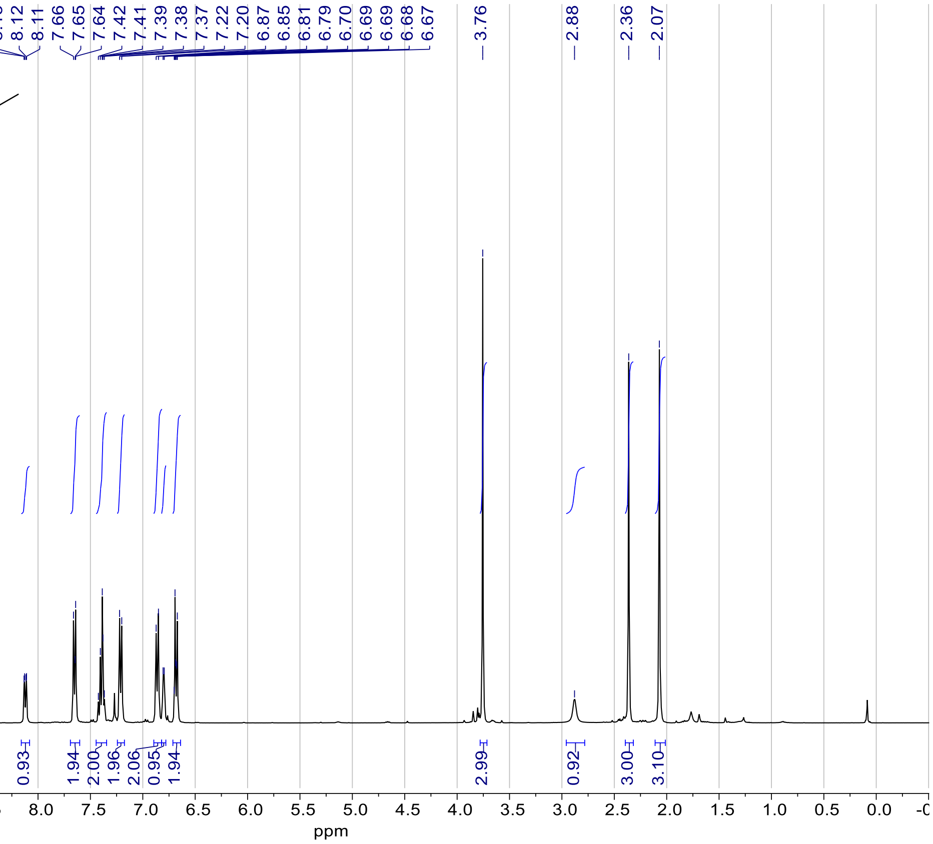

35

^{13}C NMR

(101 MHz, $CDCl_3$)

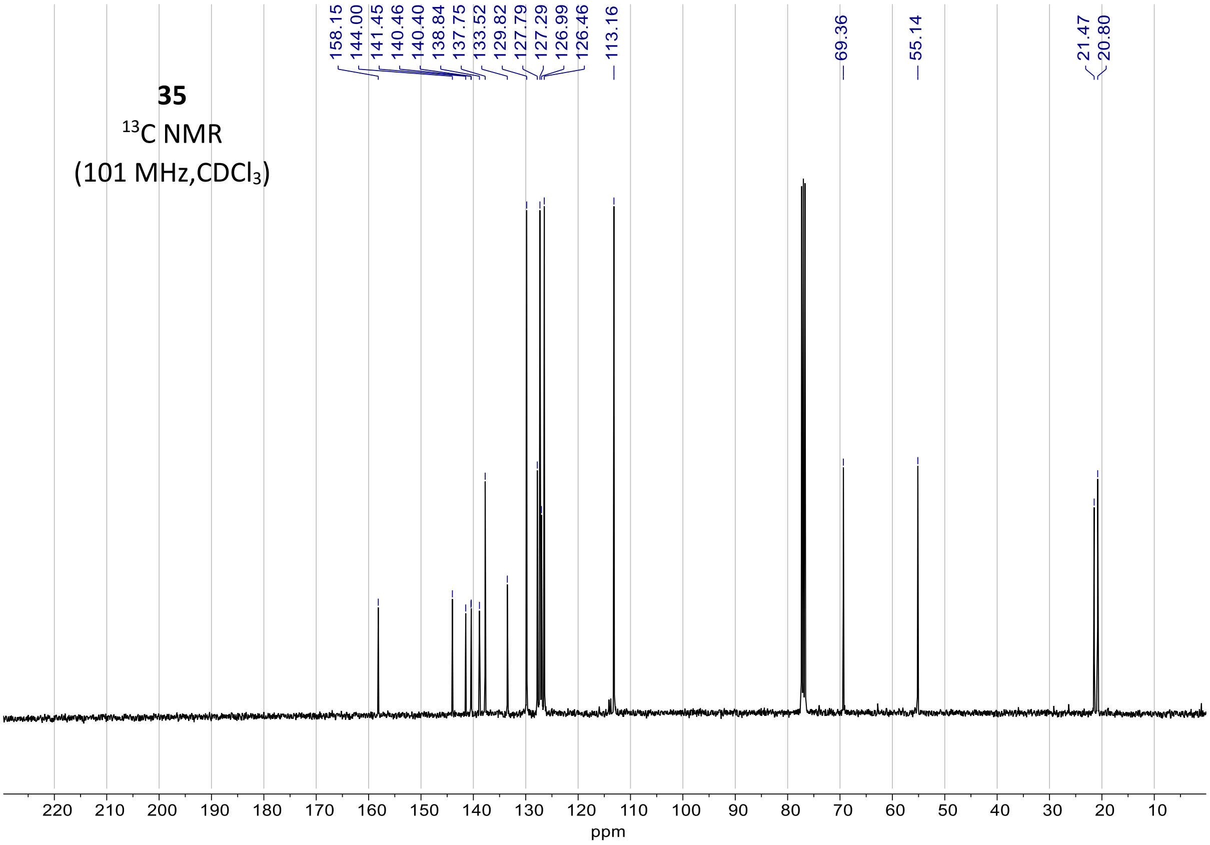

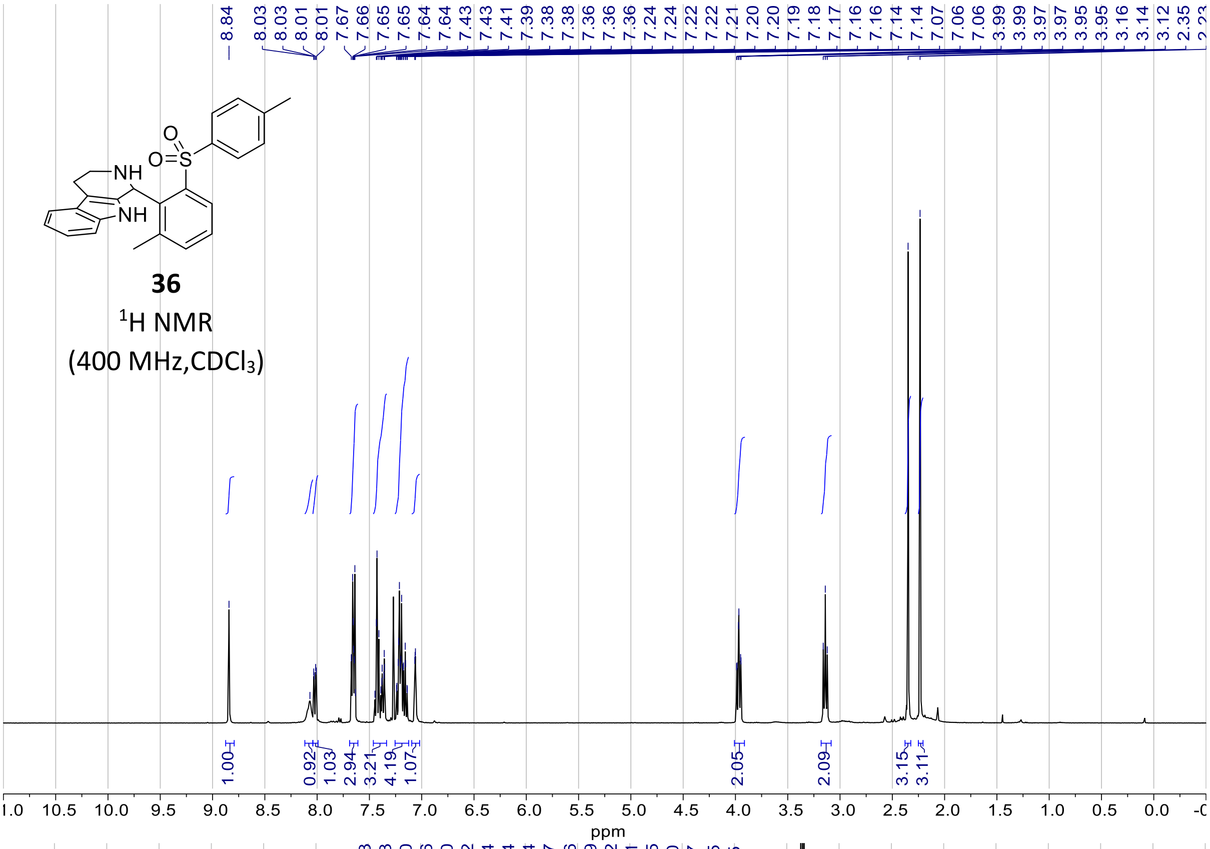
36
^{1}H NMR
(400 MHz, $CDCl_3$)
8.84
8.03 8.03 8.01 8.01 7.67 7.66 7.65 7.65 7.64 7.64 7.43 7.43 7.41 7.39 7.38 7.38 7.36 7.36 7.36 7.24 7.24 7.22 7.22 7.21 7.20 7.20 7.19 7.18 7.17 7.16 7.16 7.14 7.14 7.07 7.06 7.06 3.99 3.99 3.97 3.97 3.95 3.95 3.16 3.14 3.12 2.35
1.00 0.92 1.03 2.94 3.21 4.19 1.07 2.05 2.09 3.15 3.11
10.5 10.0 9.5 9.0 8.5 8.0 7.5 7.0 6.5 6.0 5.5 5.0 4.5 4.0 3.5 3.0 2.5 2.0 1.5 1.0 0.5 0.0
ppm

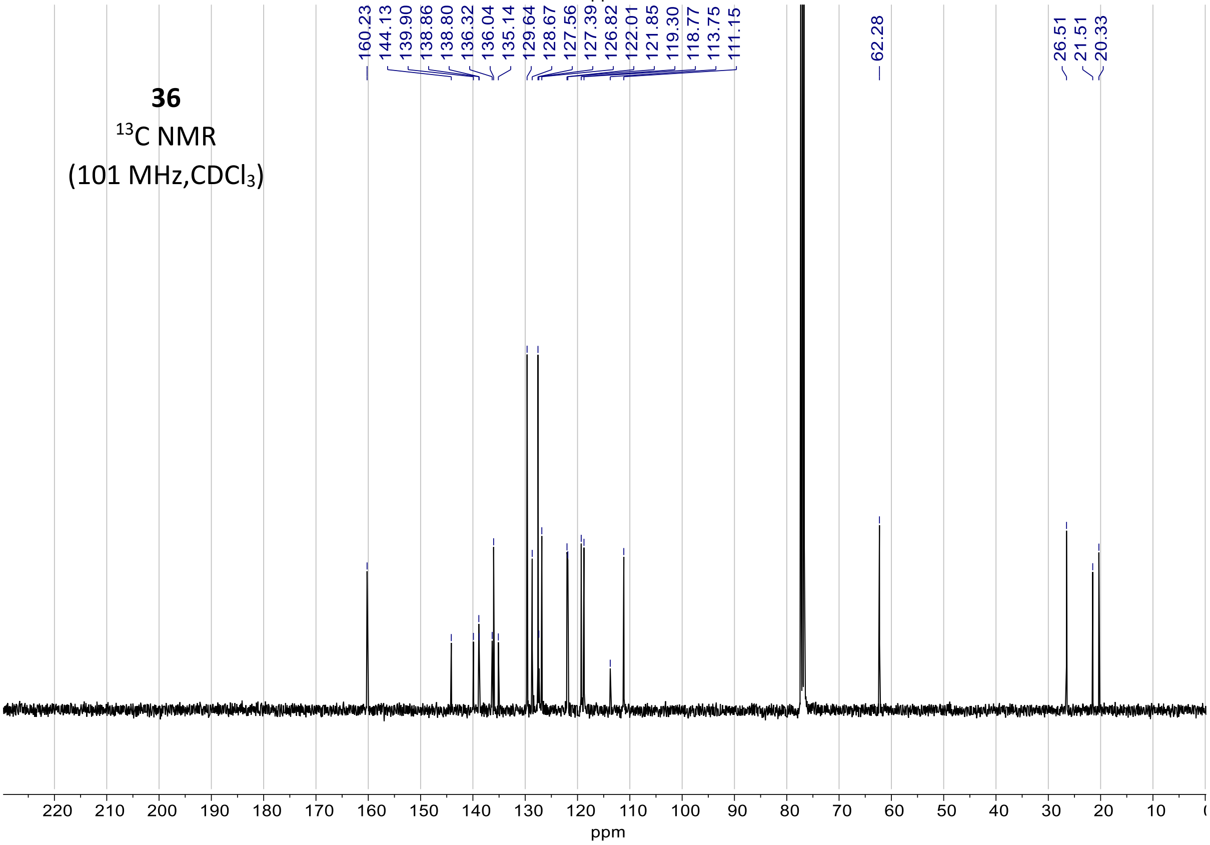
36
^{13}C NMR
(101 MHz, $CDCl_3$)
160.23 144.13 139.90 138.86 138.80 136.32 136.04 135.14 129.64 128.67 127.56 127.39 126.82 122.01 121.85 119.30 118.77 113.75 111.15
62.28
26.51 21.51 20.33
220 210 200 190 180 170 160 150 140 130 120 110 100 90 80 70 60 50 40 30 20 10
ppm

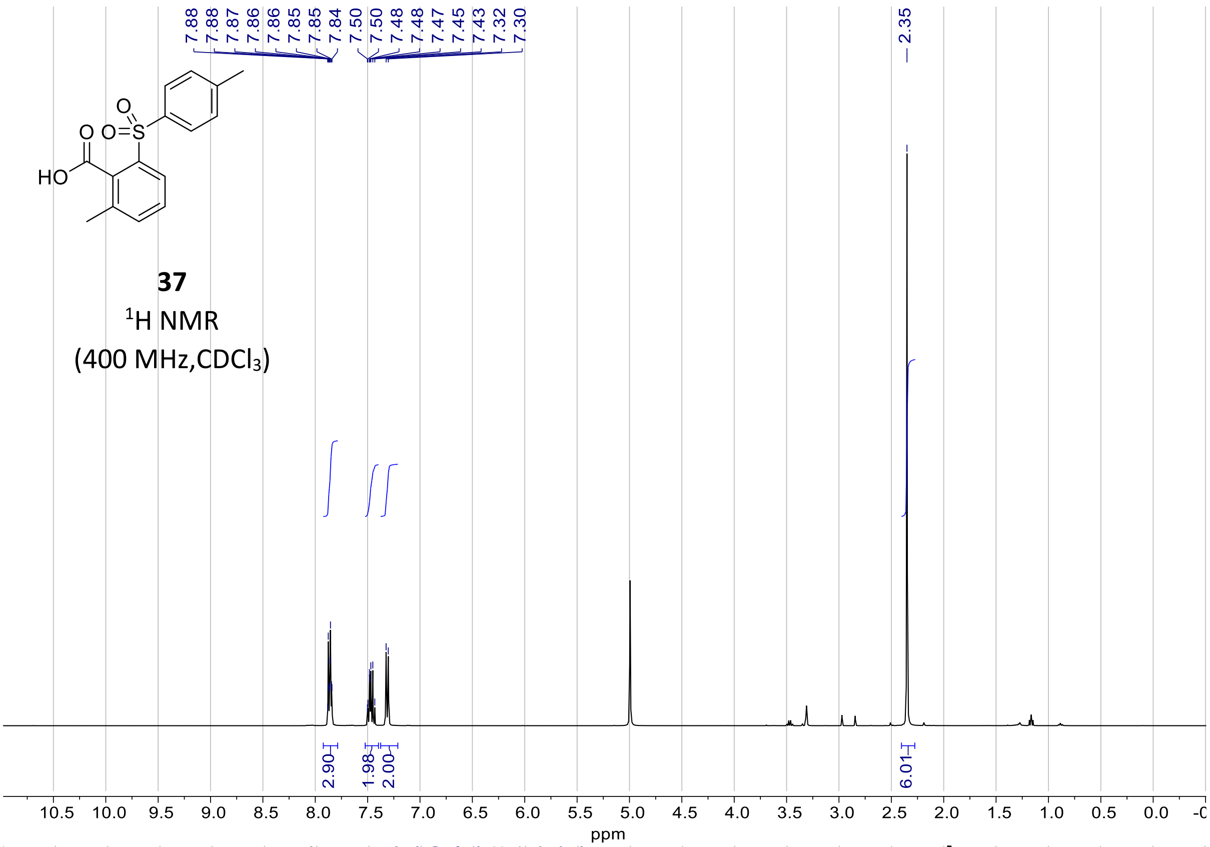
7.88
7.88
7.87
7.86
7.86
7.85
7.85
7.84
7.50
7.50
7.48
7.48
7.47
7.45
7.43
7.32
7.30
2.35
O
O=S
O
HO
37
^{1}H NMR
(400 MHz,CDCl$_3$)
2.90
1.98
2.00
6.01
10.5 10.0 9.5 9.0 8.5 8.0 7.5 7.0 6.5 6.0 5.5 5.0 4.5 4.0 3.5 3.0 2.5 2.0 1.5 1.0 0.5 0.0
ppm

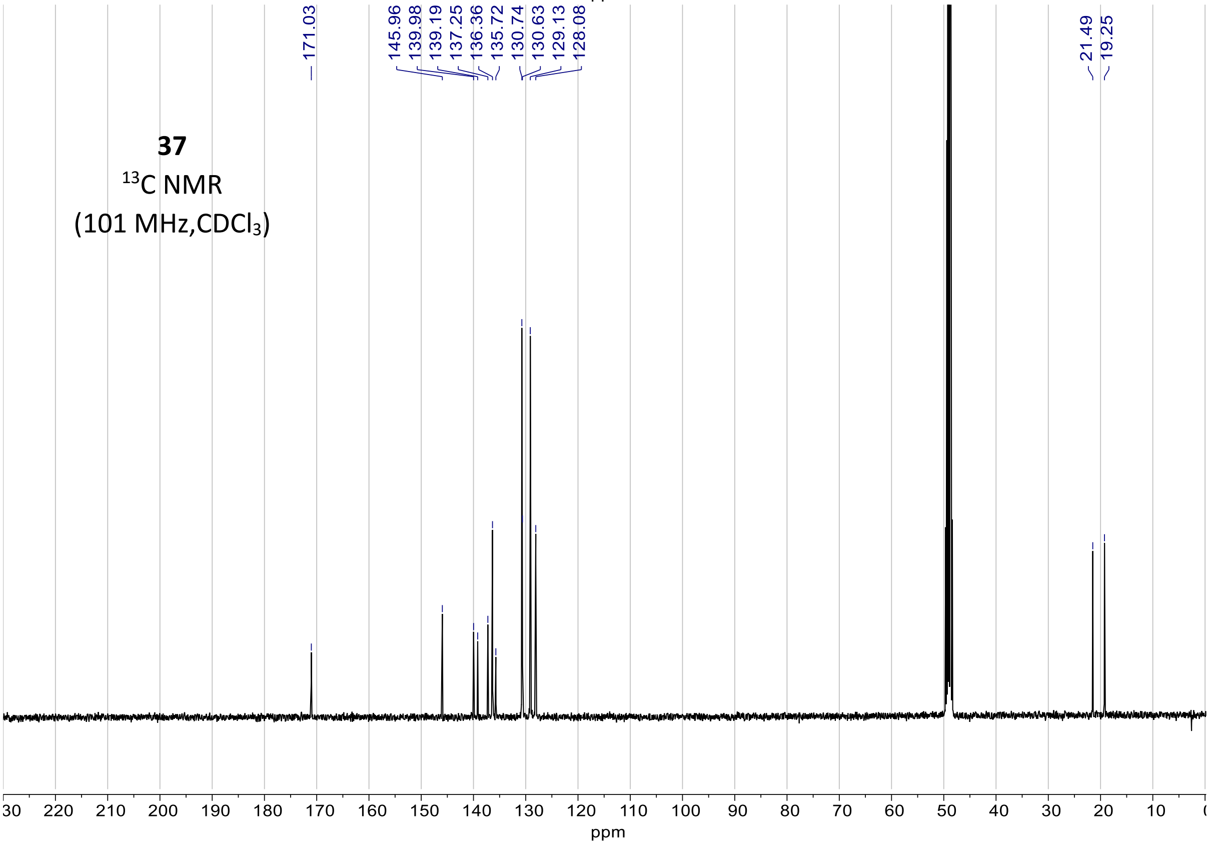
171.03
145.96
139.98
139.19
137.25
136.36
135.72
130.74
130.63
129.13
128.08
21.49
19.25
37
^{13}C NMR
(101 MHz,CDCl$_3$)
220 210 200 190 180 170 160 150 140 130 120 110 100 90 80 70 60 50 40 30 20 10
ppm

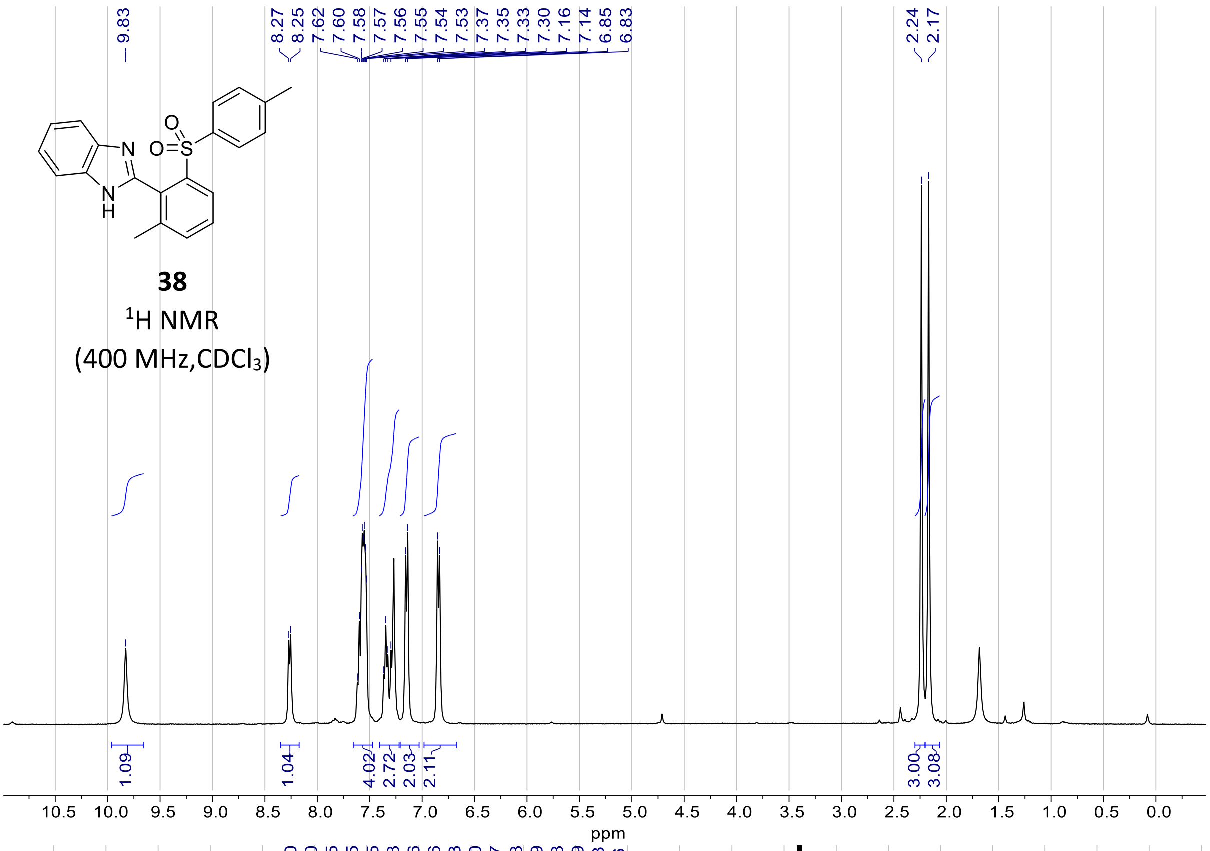

38
^{1}H NMR
(400 MHz, $CDCl_3$)

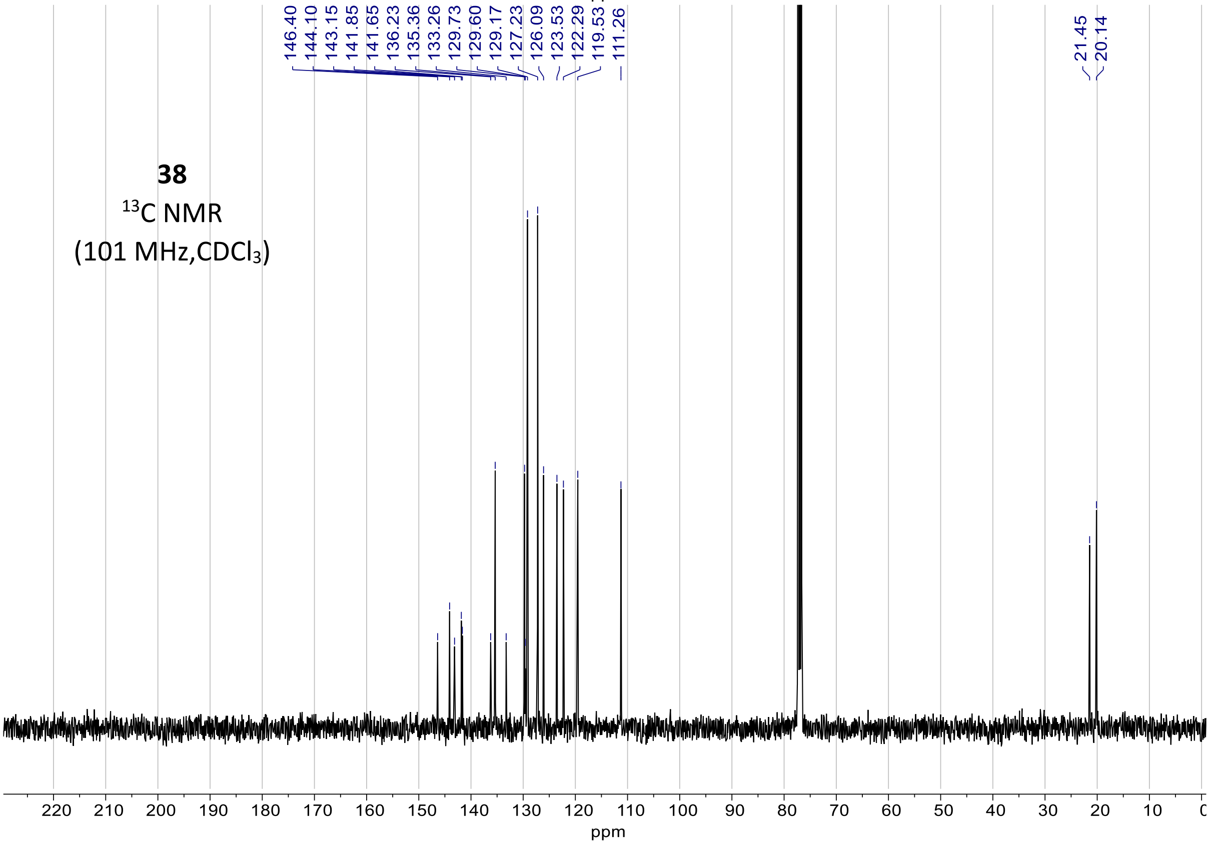

38
^{13}C NMR
(101 MHz, $CDCl_3$)

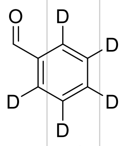

13-d$_5$
^{1}H NMR
(400 MHz,$CDCl_3$)

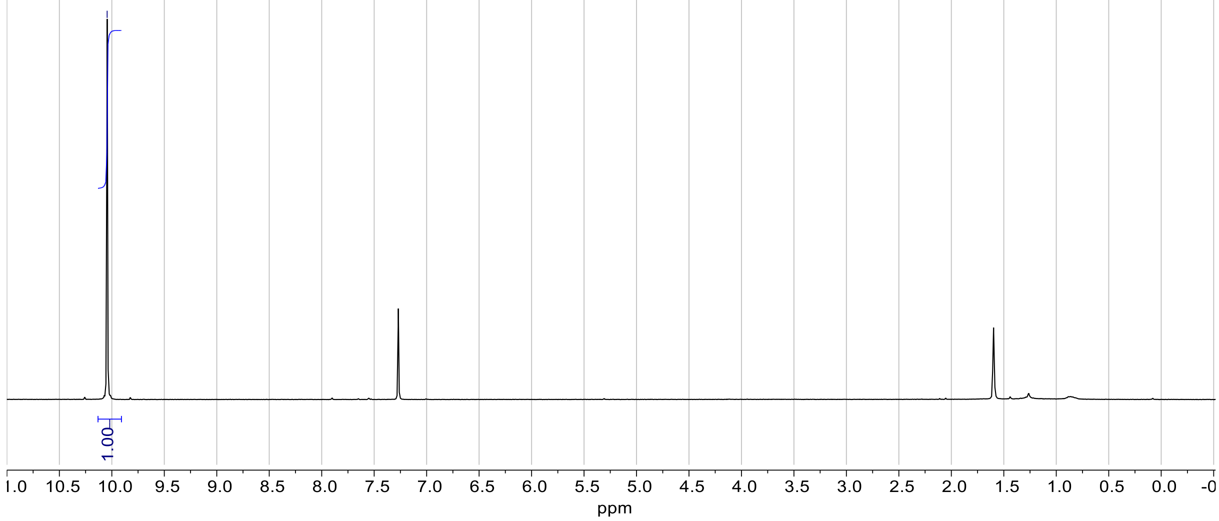

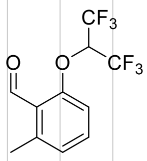

4
^{1}H NMR
(400 MHz,$CDCl_3$)

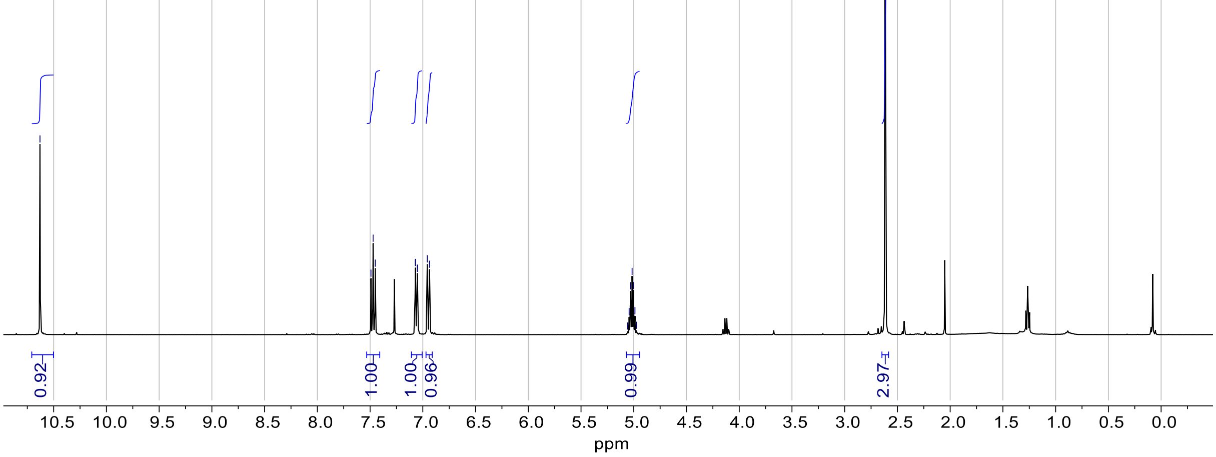

4
^{13}C NMR
(101 MHz,$CDCl_3$)

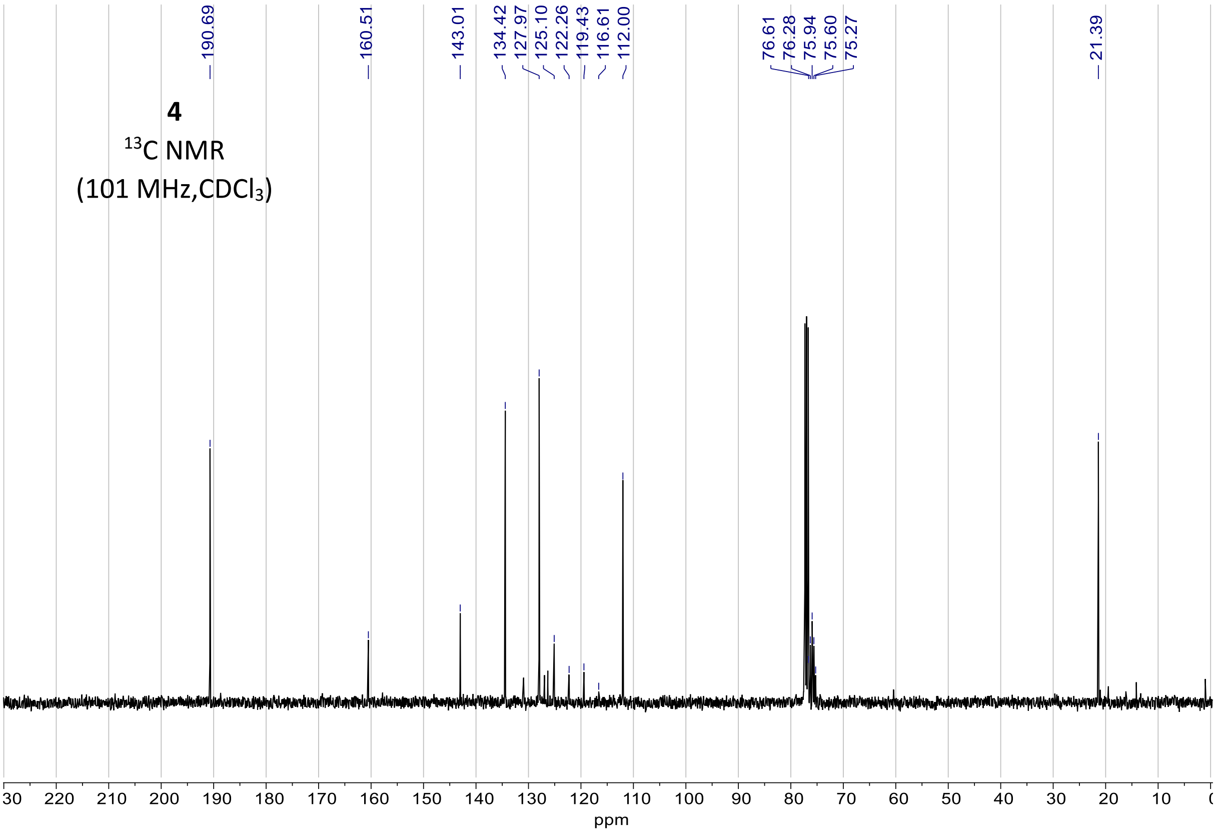

4

^{19}F NMR

(377 MHz, $CDCl_3$)

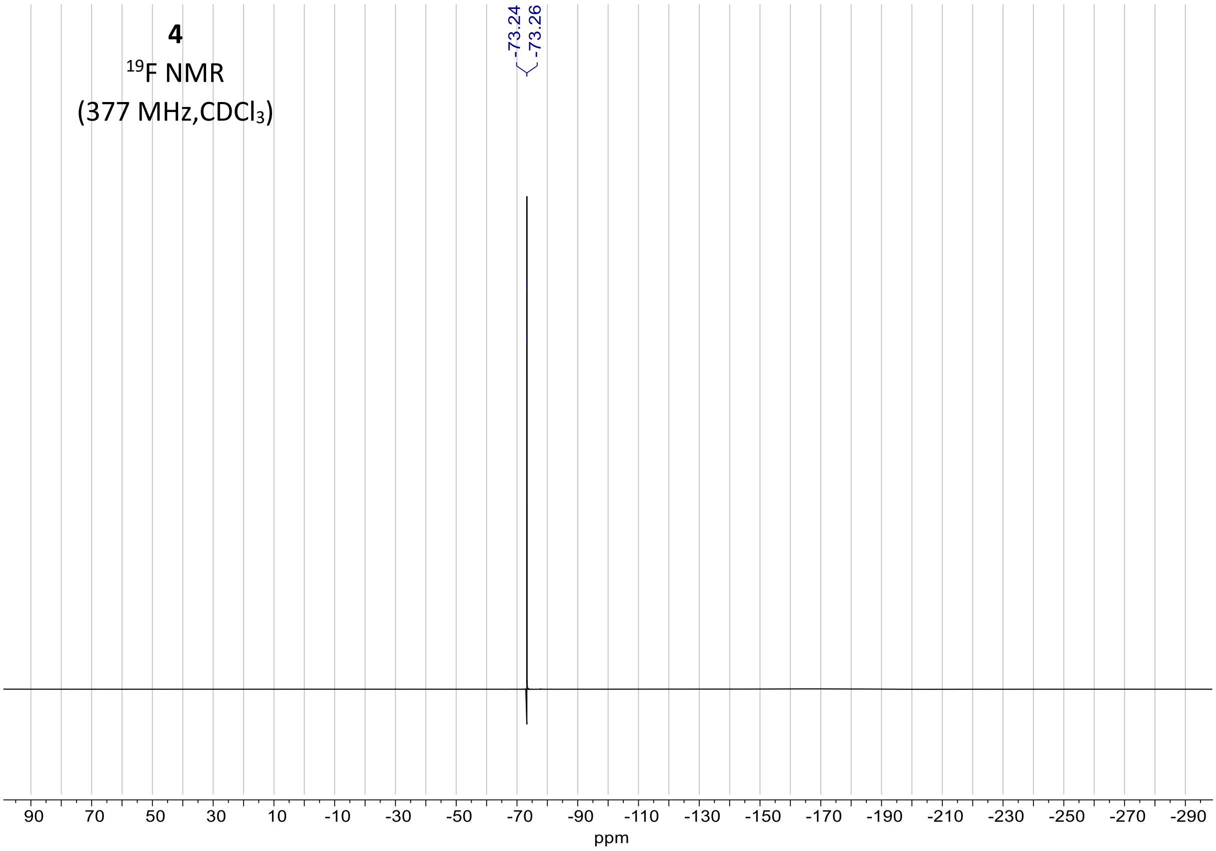

References

1 a) Y. Chen, M. C. Willis, *Chem. Sci.* **2017**, *8*, 3249–3253. b) Nandi, G. C. An Efficient Cu-Catalyzed Microwave-Assisted Synthesis of Diaryl Sulfones. *Synth. Commun.* **2017**, *47*, 319–323.

2 (a) Liu, J.; Yu, L.; Zhuang, S.; Gui, Q.; Chen, X.; Wang, W.; Tan, Z. Copper-Mediated Ortho C-H Sulfonylation of Benzoic Acid Derivatives with Sodium Sulfinates. *Chem. Commun*. **2015**, *51*, 6418–6421. (b) Rao, W. H.; Shi, B. F. Copper(II)-Catalyzed Direct Sulfonylation of C(sp2)-H Bonds with Sodium Sulfinates. *Org. Lett.* **2015**, *17,* 2784–2787. (c) Liang, S.; Liu, N. W.; Manolikakes, G. Copper-Mediated Sulfonylation of Aryl C(sp^2)-H Bonds with Sodium and Lithium Sulfinates. *Adv. Synth. Catal.* **2016**, *358*, 159–163.

3 Pitzer, L.; Schäfers, F.; Glorius, F. Rapid Assessment of the Reaction-Condition-Based Sensitivity of Chemical Transformations. *Angew. Chem. Int. Ed.* **2019**, *58*, 8572–8576.

4 (a) Blackmond, D. G. Reaction Progress Kinetic Analysis: A Powerful Methodology for Mechanistic Studies of Complex Catalytic Reactions. *Angew. Chem. Int. Ed.* **2005**, *44*, 4302–4320. (b) Baxter, R. D.; Sale, D.; Engle, K. M.; Yu, J. Q.; Blackmond, D. G. Mechanistic Rationalization of Unusual Kinetics in Pd-Catalyzed C-H Olefination. *J. Am. Chem. Soc.* **2012**, *134*, 4600–4606.

5 (a) Burés, J. A Simple Graphical Method to Determine the Order in Catalyst. *Angew. Chem. Int. Ed.* **2016**, *55*, 2028–2031. (b) Burés, J. Variable Time Normalization Analysis: General Graphical Elucidation of Reaction Orders from Concentration Profiles. *Angew. Chem. Int. Ed.* **2016**, *55*, 16084–16087. c) Nielsen, C. D. T.; Burés, J. Visual Kinetic Analysis. *Chem. Sci.* **2019**, *10*, 348–353.

6 Higham, J. I.; Bull, J. A. Copper Catalysed Oxidative α-Sulfonylation of Branched Aldehydes Using the Acid Enhanced Reactivity of Manganese(IV) Oxide. *Chem. Commun.* **2020**, *56*, 4587–4590.

7 Bär, R. M.; Gross, P. J.; Nieger, M.; Bräse, S. Sodium Bicyclo[1.1.1]Pentanesulfinate: A Bench-Stable Precursor for Bicyclo[1.1.1]Pentylsulfones and Bicyclo- [1.1.1]Pentanesulfonamides. *Chem. Eur. J.* **2020**, *26*, 4242–4245.

8 Sparks, S. M.; Aquino, C.; Banker, P.; Collins, J. L.; Cowan, D.; Diaz, C.; Dock, S. T.; Hertzog, D. L.; Liang, X.; Swiger, E. D.; et al. Exploration of Phenylpropanoic Acids as Agonists of the Free Fatty Acid Receptor 4 (FFA4): Identification of an Orally Efficacious FFA4 Agonist. *Bioorganic Med. Chem. Lett.* **2017**, *27*, 1278–1283.

9 Cheng, X. Q.; Chen, X.; Hughes, R. A.; Williams, S. J.; Woodman, O. L. Understanding the Cardioprotective Effects of Flavonols: Discovery of Relaxant Flavonols without Antioxidant Activity. *J. Med. Chem.* **2008**, *51*, 1874–1884.

10 Sarkar, D.; Ghosh, M. K.; Rout, N. Phenyl Trimethyl Ammonium Tribromide Mediated Robust One-Pot Synthesis of Spiro-Oxacycles-an Economic Route-Stereoselective Synthesis of Oxaspirohexacyclodieneones. *Org. Biomol. Chem.* **2016**, *14*, 7883–7898.

11 Zhang, M.; Li, N.; Tao, X.; Ruzi, R.; Yu, S.; Zhu, C. Selective Reduction of Carboxylic Acids to Aldehydes with Hydrosilane: Via Photoredox Catalysis. *Chem. Commun.* **2017**, *53*, 10228–10231.

12 Mohr, L. M.; Bauer, A.; Jandl, C.; Bach, T. Visible Light-Mediated Intermolecular [2 + 2] Photocycloaddition of 1-Aryl-2-Nitroethenes and Olefins. *Org. Biomol. Chem.* **2019**, *17*, 7192–7203.

13 Zhou, J.; Liu, D.; Bai, C.; Bao, A.; Muschin, T.; Baiyin, M.; Bao, Y.-S. Transient Directing Groups Controlled Regiodivergent C(Sp^3)-H and C(Sp^2)-H Polyfluoroalkoxylation of Aromatic Aldehydes. *Org. Chem. Front.* **2021**, *8*, 5975–5981.
